# Projecting the long-term economic benefits of reducing *Shigella*-attributable linear growth faltering with a potential vaccine: a modelling study

**DOI:** 10.1016/S2214-109X(23)00050-5

**Published:** 2023-05-16

**Authors:** Chloe Puett, John D Anderson, Karoun H Bagamian, Farzana Muhib, Suzanne Scheele, William P Hausdorff, Clint Pecenka

**Affiliations:** a Department of Family, Population & Preventive Medicine, Program in Public Health, Health Sciences Center, Stony Brook University, Stony Brook, NY, USA; b Bagamian Scientific Consulting, Gainesville, FL, USA; c Department of Environmental and Global Health, University of Florida, Gainesville, FL, USA; d PATH, Washington, DC, USA; e Faculty of Medicine, Université Libre de Bruxelles, Brussels, Belgium; f PATH, Seattle, WA, USA

## Abstract

**Background:**

Linear growth is an important outcome of child development with implications for economic productivity. Enteric infections, particularly *Shigella*, have been linked to linear growth faltering (LGF). However, benefits from potential reductions in LGF are rarely included in economic analyses of enteric infections. We aimed to quantify the economic benefits of vaccination related to reduced *Shigella*-attributable disease and associated LGF compared with the net costs of a vaccine programme.

**Methods:**

In this benefit–cost analysis, we modelled productivity benefits in 102 low-income and middle-income countries that had recent stunting estimates available, at least one *Shigella*-attributable death annually, and available economic data, particularly on gross national income and growth rate projections. We modelled benefits strictly related to linear growth improvements and no other benefits associated with reducing diarrhoeal burden. The effect size in each country was calculated as shifts in height-for-age Z score (HAZ), representing population average changes for preventing *Shigella*-attributable less-severe diarrhoea and moderate-to-severe diarrhoea separately for children younger than 5 years. Benefits data were calculated per country and combined with estimated net costs of the vaccine programme in the form of benefit–cost ratios (BCRs); BCRs above parity, or $1 in benefits per $1 in costs (with a 10% margin representing borderline results: 1·10:1), were considered cost-beneficial. Countries were aggregated for analysis by WHO region, World Bank income category, and eligibility for support from Gavi, the Vaccine Alliance.

**Findings:**

In the base-case scenario, all regions exhibited cost-beneficial results, with the South-East Asia region and Gavi-eligible countries exhibiting the highest BCRs (21·67 for the South-East Asia region and 14·45 for Gavi-eligible countries), and the Eastern Mediterranean region yielding the lowest BCRs (2·90). All regions exhibited cost-beneficial results from vaccination, except in more conservative scenarios (eg, those assuming early retirement ages and higher discount rates). Our findings were sensitive to assumed returns for increased height, assumptions about vaccine efficacy against linear growth detriments, the anticipated shift in HAZ, and discount rate. Incorporating the productivity benefits of LGF reduction into existing cost-effectiveness estimates resulted in longer-term cost-savings in nearly all regions.

**Interpretation:**

LGF is a secondary outcome of *Shigella* infection and reduction in LGF is not often quantified as a health or economic benefit of vaccination. However, even under conservative assumptions, a *Shigella* vaccine only moderately effective against LGF could pay for itself from productivity gains alone in some regions. We recommend that LGF be considered in future models assessing the economic and health impacts of interventions preventing enteric infections. Further research is needed on vaccine efficacy against LGF to inform such models.

**Funding:**

Bill & Melinda Gates Foundation, Wellcome Trust.

## Introduction

Linear growth faltering (LGF) is an important public health concern, with more than 300 million children worldwide having at least mildly stunted growth.^1^ Child growth is linked to cognitive development.^2^ Moreover, suboptimal growth is associated with an increased mortality risk for children younger than 5 years.^3^

LGF can be caused by several factors, including poor diet quality, inadequate caring and feeding practices, suboptimal hygiene, and increased disease burden.^4^ Some enteric infections have been linked to LGF in young children;^5^ although the mechanism is not fully understood, this association is hypothesised to result from infection-triggered gut inflammation, known as environmental enteric dysfunction.^6^ One such example is the enteric bacterial pathogen *Shigella*: studies have repeatedly identified *Shigella*-attributable illness or subclinical infections as being associated with suboptimal growth in young children.^7^, ^8^

In the longer term, suboptimal linear growth can impair economic productivity. A wide body of literature examines the linkages between increased height and improved income, also known as the height premium. This literature reports increases in income per additional cm in adult height, ranging from 0·1% to 5·0% on average, depending on study context and methods used.^9^

**Research in context**

**Evidence before this study**

Recent studies in the field of nutrition have quantified the future economic productivity benefits of improving child linear growth with a package of nutrition interventions, and compared these benefits with the programmatic and societal costs of such interventions. This benefit–cost evidence aids in cross-sectoral priority setting to maximise anticipated benefits when investing scarce resources. For the present study we adapted methods outlined in several studies from the nutrition field, such as those by Hoddinott and colleagues (2013), Alderman and colleagues (2017), and Galasso and Wagstaff (2019), that quantified productivity benefits from improving child linear growth, and applied these methods to an economic evaluation of a vaccine intervention.

**Added value of this study**

To our knowledge, this is the first study to estimate productivity benefits of vaccine-reduced linear growth faltering attributable to *Shigella* infection. This expanded scope provides policy-relevant data quantifying the potential longer-term economic benefits of reducing *Shigella* burden, which have not been calculated previously.

**Implications of all the available evidence**

Improving linear growth is only one potential benefit of reducing *Shigella* and related diarrhoeal disease burden, and yet the benefits appear to be substantial for some regions. This finding suggests that the economic value of *Shigella* vaccination might be much greater than previously estimated. These results complement findings from recent cost-effectiveness analyses of *Shigella* vaccines, which suggest that *Shigella* vaccines could be cost-effective, and even cost-saving, in some regions when including productivity benefits in the calculations. Future economic analyses of interventions preventing enteric infections should include benefits of reducing or preventing diarrhoea-related stunting or growth faltering to value a broader set of benefits.

Studies in the nutrition field have used these findings and empirical data from longitudinal trials measuring child growth and adult productivity to quantify the economic benefits of improving growth via a package of nutrition interventions.^10^, ^11^, ^12^ Pairing these data on benefits with intervention costs enables benefit–cost analysis.

Although there is increasing interest in both the nutrition and vaccine fields to include broader societal benefits in economic evaluations,^13^ fewer than half of vaccine economic evaluations do so,^14^ resulting in an undervaluation of vaccines.^15^ Evaluations that include broader societal benefits do not commonly include productivity benefits related to longer-term outcomes,^16^ although it is recommended to include later-life benefits linked with improving young children's nutrition and health, particularly their height.^17^, ^18^

To date, the benefits of improving child growth have not been estimated regularly in fields beyond nutrition. The present study quantifies the economic benefits of vaccination related to reduced *Shigella*-attributable disease and associated LGF compared with the net costs of vaccine programmes. Although several *Shigella* vaccine candidates are in advanced clinical development, it remains unclear whether the potential health and economic benefits of *Shigella* vaccination, as traditionally assessed,^19^ would be considered sufficient for its prioritisation over competing public health interventions by global and national recommending bodies. By quantifying the potential productivity benefits from vaccine-induced reductions in LGF, this economic analysis also directly addresses a key recommendation by the WHO Burden of Enteric Diseases Morbidity Working Group^20^ and thereby should promote a better understanding of the true public health value of *Shigella* vaccination.

## Methods

### Analytical strategy and effect size estimation

We modelled the future productivity benefits of a vaccine that reduces *Shigella*-attributable disease and associated LGF. This analysis was conducted alongside a complementary impact and cost-effectiveness study using similar assumptions but focused on the short-term impacts on *Shigella*-attributable health and economic burden during childhood.^19^ In this study, we modelled benefits strictly related to linear growth improvements and no other benefits associated with reducing diarrhoeal burden. Inclusion criteria for study countries were as follows: World Bank categorisation as low-income or middle-income; having at least one *Shigella*-attributable death annually according to 2019 Global Burden of Disease Study estimates; and availability of economic data, including gross national income (GNI) and economic growth rate projections. The final model included 102 countries (appendix p 2). Since no individual data were used for this analysis, and only publicly available data were used, ethics committee approval was not required or sought.

The effect size in each country was calculated as shifts in height-for-age Z score (HAZ), representing population average changes for preventing *Shigella-*attributable less-severe diarrhoea (LSD) and moderate-to-severe diarrhoea (MSD) separately for children younger than 5 years.^19^ *Shigella-*attributable HAZ shifts were estimated by the product of annual *Shigella-*attributable MSD and LSD episode rates during the first 5 years of life^19^ and the HAZ shifts associated with episode severity.^21^ Although other studies have also linked subclinical *Shigella* infections to growth faltering,^7^ we took the conservative approach of only including the contribution of symptomatic *Shigella* infections to growth faltering, because global data on asymptomatic infection are scarce at this time. The proportions of the HAZ shifts that were prevented by vaccination were extrapolated from assumptions derived from WHO's Preferred Product Characteristics for *Shigella* vaccines, namely 40% efficacy in preventing *Shigella-*attributable LSD episodes and 60% efficacy in preventing *Shigella*-attributable MSD episodes caused by vaccine serotypes, multiplied by the projected vaccine coverage in each country.^22^ Separate LSD and MSD shifts were summed to estimate the full average effect per country. We assessed the sensitivity of our findings to a lower efficacy estimate and to include only MSD-associated burden.

Benefits data were calculated per country, using the HAZ shifts described above and the model procedures outlined below, and combined with estimated net costs of the vaccine programme from our companion study,^19^ to perform benefit–cost analysis. Countries were aggregated for analysis by WHO region, World Bank income category, and eligibility for support from Gavi, the Vaccine Alliance.

Benefit–cost ratios (BCRs) provide information on the extent to which the economic benefits of reducing LGF with *Shigella* vaccination, presented in monetary terms, outweighed the vaccine programme costs for particular country groupings. BCRs above parity, or $1 in benefits per $1 in costs (with a 10% margin representing borderline results: 1·10:1), were considered cost-beneficial. Higher ratios indicate higher returns for particular country groupings.

### Model structure

**Figure 1:**
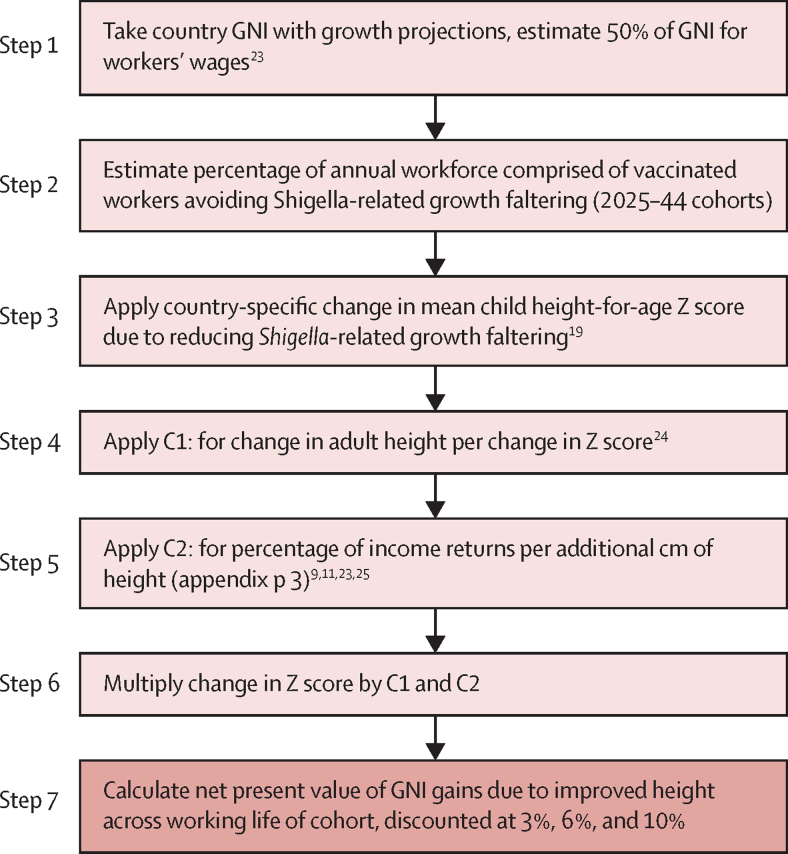
Analytical flowchart of the economic model Data sources for steps 1 and 2 included the World Bank and the IMF. C1=coefficient 1. C2=coefficient 2. GNI=gross national income. IMF=International Monetary Fund.

Our general modelling approach for estimating economic benefits is described below and depicted in figure 1. The vaccine programme was modelled to last 20 years. Benefits were calculated annually, starting when the first vaccinated cohort entered the workforce through to retirement of the final vaccinated cohort, assuming the Organisation for Economic Development and Co-operation (OECD) standard working age of 15–64 years. We estimated the annual percentage of the workforce having received the vaccine on the basis of the total years of working age included in the labour force, adjusted for country-specific unemployment rates.

For each year, the country-specific HAZ shift was multiplied by a coefficient for the change in adult height per change in Z score,^24^ following previous work.^10^ This process yielded an average estimate of additional cm grown per country, which was then multiplied by a height premium estimate.

Benefit estimation was based on each country's 2019 GNI, adjusted for a conservative wage share of 50%.^23^ Projected annual growth rates were applied up to and including 2026, and thereafter we applied an average of the latest 5-year growth-rate projections minus 1%, as a conservative measure.

Because benefits accrued far in the future, we projected each country's per-capita GNI (where available) and compared these to projected World Bank income category cutoffs adjusted for 2% inflation. Values for the height premium estimate and the economic multiplier were assigned on the basis of country income category, with new values applied in the year of any projected category changes.

Finally, we calculated the present value of expected changes in children's future lifetime earnings, which is sensitive to the chosen discount rate. There is no consensus on which rate is appropriate to use.^26^ We assessed sensitivity to two rates common in the nutrition literature (3% and 6%)^10^, ^11^ and to a more conservative scenario (with a rate of 10%) in the sensitivity analysis.

We used vaccine programme net costs from the health-care system perspective from the companion analysis (appendix p 24), which have been described elsewhere.^19^ The *Shigella* vaccine was assumed to be an immediately effective two-dose vaccine given by the age of 6 months. Second-dose and third-dose coverage of the diphtheria, tetanus, and pertussis combination vaccine were used as proxies for the two doses of *Shigella* vaccine, as the timing for *Shigella* vaccination is not yet confirmed. Partial coverage was not included in the analysis. Administrative costs varied by country income category, and vaccine prices were estimated on the basis of recent rotavirus and pneumococcal vaccine prices by income group and Gavi eligibility (appendix p 24). For all Gavi-eligible countries, vaccine price was estimated at $2 ($1–3; appendix p 24) and reflected a lower but full Gavi-negotiated vaccine price and not just the country's copay (ie, the portion of the price the country is responsible for). Vaccine prices for Gavi-ineligible countries were $7·10 ($3·55–10·65) in lower-middle-income countries (LMICs), $9·73 ($4·87–14·60) in upper-middle-income countries (UMICs), and $6·75 ($3·73–10·12) in countries in the region of the Americas (LMICs and UMICs). We did not have an equivalent estimate for low-income countries (LICs) because there were no Gavi-ineligible LICs. Wastage was assumed to be 10% of vaccine costs. We estimated medical costs averted by vaccination on the basis of an analysis of rotavirus direct inpatient and outpatient costs,^27^ and 20-year vaccine programme costs were adjusted for inflation at 2%.

Analyses were conducted in Microsoft Excel. We present the net present value of costs and benefits in 2019 USD.

### Scenario analyses

We estimated the results in several sets of scenarios. We constructed two retirement scenarios with the same underlying assumptions and costs except for retirement age, which was 64 years for the average retirement scenario and 50 years for the early retirement scenario (table 1). Results for each retirement scenario are presented as two sets of effects estimates: the income effect and the multiplier effect.

**Table 1:** Model scenario parameters and definitions

	**Working age (years)**	Multiplier*	**Income categories**	**Discount rate**	**Burden envelope**	**Z score shift**†	**Net costs**†
Average retirement scenario	15–64 (OECD)	LIC: 0·73; LMIC: 0·73; UMIC: 0·46‡	LIC, LMIC, and UMIC	3%, 6%, and 10%§	LSD and MSD	Average estimate per country	Average estimate per country
Early retirement scenario	15–50	LIC: 0·73; LMIC: 0·73; UMIC: 0·46‡	LIC, LMIC, and UMIC	3%, 6%, and 10%§	LSD and MSD	Average estimate per country	Average estimate per country
Base-case scenario	15–64	None	LIC and LMIC only (for sensitivity analysis)¶	3%	LSD and MSD	Average estimate per country	Average estimate per country
Conservative scenario	15–50	None	LIC and LMIC only (for sensitivity analysis)¶	3%	MSD only	Average estimate per country	Average estimate per country

The height premium corresponded to an increase in income per cm of adult height gained. Estimates are medians from a literature review conducted for this study (appendix): LIC: 0·87% (median of 2 LIC studies, 6 LMIC); LMIC: 0·83% (median of 6 LMIC studies); UMIC: 0·87% (median of 6 LMIC studies, 2 UMIC); HIC: 0·26% (median of 13 HIC studies). LIC=low-income country. LMIC=lower-middle-income country. LSD=less-severe diarrhoea. MSD=moderate-to-severe diarrhoea. OECD=Organisation for Economic Co-operation and Development. UMIC=upper-middle income country.

* Multipliers represent the broader societal effects of increased productivity, which differ by income class.

† Full results, including point estimates and uncertainty intervals for the Z score shift and net cost parameters are included in the appendix.

‡ The average retirement and early retirement scenarios are presented with both income effect only and multiplier effects.

§ Results for 10% discount scenario are presented in the appendix.

¶ Results for the base-case scenario are presented for both income category groups (with and without UMICs). For the sensitivity analysis, results are compared with the base case for LIC and LMIC only.

The income effect represents the economic benefits of improved income due to average height increases per country. To calculate this effect, we applied median height premium estimates per country income category from a literature review, ranging from an increase of 0·83% to 0·87% in income per cm gained (appendix p 3). Given the sparse data for LICs and UMICs for these categories (two studies for each category), we used the median, including LMIC estimates for which data were more available (six studies) than for LICs and UMICs (table 1). During the model timeframe, a few UMICs would transition to high-income countries (HICs). We used a conservative HIC estimate from our literature review in all relevant scenarios (13 studies). We included an optimistic scenario in the appendix to present returns using a larger height premium estimate than the one included in the results presented in this Article, from empirical findings from Latin America.

The multiplier effect additionally incorporates an economic multiplier, representing the broader societal effects of increased productivity. Economic multipliers are based on the marginal propensity to consume (MPC) in different contexts. For example, an MPC of 0·7 is interpreted as a population consuming 70% and saving 30% of additional income. Individuals in such a country would spend 70% of any additional income on goods and services, further increasing GNI. The multiplier is calculated as 1/(1 – MPC). From a literature review, we calculated average MPC estimates for LMICs and UMICs (table 1; appendix p 4). As no published estimates were found for LICs, we applied LMIC estimates for these countries. Inclusion of multiplier effects yields optimistic estimates of the vaccine's full economic benefits; as a conservative measure, multiplier effects were not included in the base-case and conservative scenario analyses.

The base-case scenario was defined as the average retirement income effect scenario at 3% discounting; for sensitivity analyses, the base case refers to results from LICs and LMICs only.

### Combining long-term and short-term vaccination benefits

We combined long-term vaccination benefits (ie, future productivity gains) from this study and short-term vaccination benefits (ie, averted disability-adjusted life years [DALYs] and related costs) from our companion study^19^ to examine the lifelong impact of vaccination (appendix p 24). To do this, we calculated incremental cost-effectiveness ratios (ICERs) combining the net costs of vaccination with two long-term benefit scenarios outlined in table 1—the base-case scenario (average retirement income effect, including both LSD and MSD) and a conservative scenario (early retirement income effect, including MSD only). The conservative scenario assumes that the vaccine is effective in reducing only *Shigella* MSD-attributable morbidity (ie, episodes and stunting); total mortality (ie, deaths from acute *Shigella* episodes and from other infections due to *Shigella*-attributable stunting); and medical costs. The base-case scenario assumes the vaccine is effective in reducing *Shigella* LSD-attributable and MSD-attributable morbidity, total mortality, and medical costs*.*

We calculated short-term ICERs^19^ by dividing incremental vaccine programme net costs by DALYs averted, aggregated by region, income class, and Gavi eligibility (appendix p 24). The comparator scenario was no vaccination. We calculated ICERs of long-term benefits by subtracting the productivity gains from the net costs and then dividing this by the relevant scenario-specific DALYs (appendix p 24). ICERs were estimated using probabilistic methods, and 95% uncertainty intervals are presented from 1000 model simulations (appendix p 24).

### Sensitivity analyses

We did univariate sensitivity analyses (appendix pp 16–22) to understand the relative impact on BCRs of a plausible range of model parameter values, summarised in tornado diagrams for key country groupings. These analyses explored sensitivity relative to the base case (average retirement income effect scenario at 3% discounting for LICs and LMICs). We assessed the following variables and ranges in sensitivity analyses: HAZ shift (lower and upper uncertainty interval; appendix pp 26–34 and 43–51); vaccine programme net costs (lower and upper uncertainty interval; appendix pp 60–69); height premium (worst case: 0·55%,^10^, ^23^ best case: 1·3%^11^); 10% discounting (compared with 3% in the base case); only MSD costs and effects (compared with MSD and LSD in the base case; appendix pp 43–52 and 69–78); and low vaccine efficacy of 10% against all diarrhoea and growth faltering (compared with base-case values of 40% [LSD] and 60% [MSD]; appendix pp 34–43, 52–60).

### Role of the funding source

The funders of the study had no role in study design, data collection, data analysis, data interpretation, or writing of the report.

## Results

Under the average retirement income effect scenario, BCRs were above parity at 3% discounting for all regions, remaining above parity at 6% discounting for all except for the Eastern Mediterranean region (table 2). Under the early retirement income effect scenario, BCRs were above parity for all regions at 3% discounting and for most at 6% discounting (table 2A).

**Table 2:** Base-case benefit–cost ratios including productivity benefits of preventing *Shigella*-attributable growth faltering through vaccination

	**Average retirement scenario**				**Early retirement scenario**			
	Income effect		Multiplier effect		Income effect		Multiplier effect	
	Discounting 3%	Discounting 6%	Discounting 3%%	Discounting 6%	Discounting 3%%	Discounting 6%	Discounting 3%%	Discounting 6%
**LICs and LMICs only**								
African region	8·52	2·63	23·00	7·62	4·98	1·95	14·61	5·99
Region of the Americas	3·32	1·18	12·62	4·49	2·38	1·00*	9·06	3·79
Eastern Mediterranean region	2·90	0·97*	8·01	2·73	1·92	0·77*	5·40	2·22
European region	4·06	1·36	15·43	5·15	2·70	1·09*	10·27	4·15
South-East Asia region	21·67	5·95	40·51	11·14	11·00	3·97	20·61	7·45
Western Pacific region	6·56	1·92	12·35	3·63	3·66	1·37	6·95	2·61
Gavi-eligible countries	14·45	4·11	30·42	8·95	7·63	2·83	16·78	6·38
Overall	11·60	3·34	24·55	7·31	6·23	2·32	13·78	5·27
**LICs, LMICs, and UMICs**								
African region	8·11	2·53	21·59	7·18	4·81	1·89	13·80	5·67
Region of the Americas	10·85	3·97	20·85	7·62	8·02	3·41	15·40	6·54
Eastern Mediterranean region	2·69	0·91*	7·06	2·42	1·80	0·73*	4·79	1·98
European region	4·65	1·61	10·54	3·61	3·23	1·33	7·27	2·98
South-East Asia region	15·26	4·27	28·52	8·00	7·97	2·91	14·92	5·46
Western Pacific region	5·68	1·73	10·60	3·22	3·38	1·29	6·31	2·41
Gavi-eligible countries	14·45	4·11	30·42	8·95	7·63	2·83	16·78	6·38
Overall	8·53	2·58	17·26	5·31	4·94	1·90	10·22	3·97

Average retirement age was defined as 64 years as per the OECD, whereas early retirement age was defined as 50 years. LIC=low-income country. LMIC=lower-middle-income country. OECD=Organisation for Economic Co-operation and Development. UMIC=upper-middle-income country.

* Results less than or equal to 1·10, representing ratios that are below parity or borderline.

**Figure 2:**
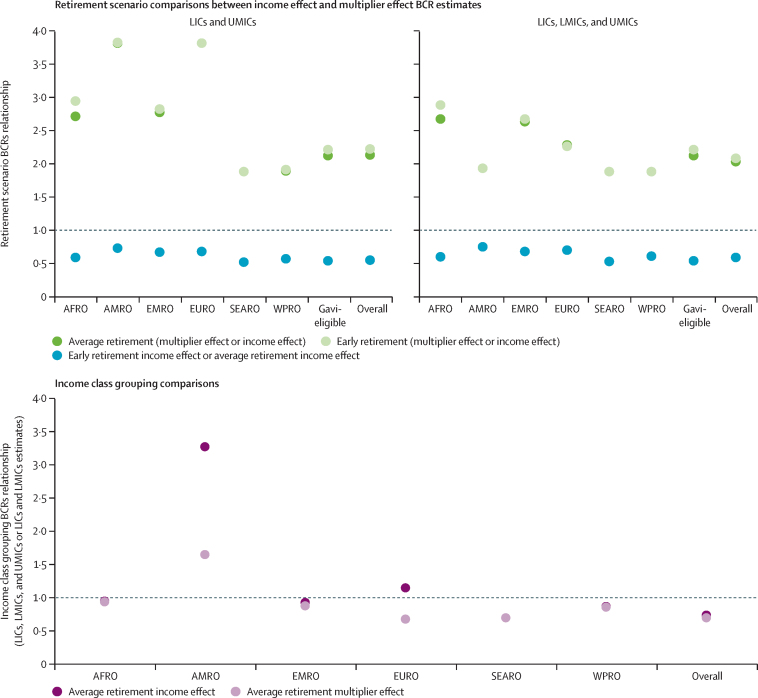
Comparing the magnitude of change between BCRs in different scenarios The y-axis represent the magnitude of change between two BCRs when comparing them by dividing the first BCR by the second. (A) Comparisons of BCRs between (1) average retirement (multiplier effect divided by income effect), (2) early retirement (multiplier effect divided by income effect), and (3) early retirement income effect divided by average retirement income effect. (B) Comparisons of BCRs for LICs, LMICs, and UMICs, and BCRs for estimates excluding UMICs (LICs and LMICs only) in each region and overall. Comparisons were made between (1) average retirement income effect and (2) average retirement multiplier effect estimates. Panel B excludes estimates for Gavi-eligible countries because there are no Gavi-eligible UMICs among the 102 countries included in this model. AFRO=African region. AMRO=region of the Americas. BCR=benefit–cost ratio. EMRO=Eastern Mediterranean region. EURO=European region. LIC=low-income country. LMIC=lower-middle-income country. SEARO=South-East Asia region. UMIC=upper-middle-income country. WPRO=Western Pacific region.

Within both retirement scenarios, inclusion of the multiplier effect substantially increased cost-savings, doubling or tripling BCRs for most regions or groupings at both discount rates (table 2A), and quadrupling them for the region of the Americas and the European region (LICs and LMICs only; figure 2A). In addition, including the multiplier effect resulted in all regions reaching parity at the more conservative 6% discount rate under both retirement scenarios.

Under the base-case scenario (average retirement income effect at 3% discounting), the South-East Asia region and Gavi-eligible countries had the highest BCRs, which were 21·67 for the South-East Asia region and 14·45 for Gavi-eligible countries (table 2A). The BCRs for the South-East Asia region were consistently the highest for all scenario and effect combinations. The BCRs for the Eastern Mediterranean region were the lowest among regions and were below parity (or borderline) for several scenario and effect combinations (table 2).

When UMICs were included in the analysis, the BCRs for the region of the Americas were 3 times higher for the average retirement income effect and 1·7 times higher for the average retirement multiplier effect scenario than when UMICs were not included (figure 2B). In the European region, BCRs were 1·2 times higher for the average retirement income effect and 0·7 times lower for the average retirement multiplier effect scenario when UMICs were included than when they were not. BCRs of all other regions and groupings remained largely the same, with the African region experiencing the least BCR change, due to only four of 42 countries being a UMIC (table 2).

For most regions, costs or benefits (or both) were influenced by a few dominant countries. For example, in the base case, India drove overall economic trends in the South-East Asia region (51% of costs and 81% of benefits), as China did in the Western Pacific region (81% of costs and 80% of benefits; appendix pp 8–15). The African region comprises more LICs and LMICs than other regions in our analysis, with no single economy influencing results.

Vaccination under the average retirement income effect scenario was cost-saving in all regions and income groupings (ICERs ≤0; table 3). Vaccination under the early retirement income effect and MSD scenario was cost-saving (ICERs ≤0) in all regions except the region of the Americas ($1 per DALY averted) and the Eastern Mediterranean region ($37 per DALY averted) in the analysis restricted only to LICs and LMICs. When UMICs were included in the analysis, all regions were cost-saving except the Eastern Mediterranean region ($50 per DALY averted).

**Table 3:** Short-term net costs of the vaccination programme, long-term productivity benefits of vaccination, and ICERs

	**Short-term net costs of vaccination with LSD and MSD burden**	**Long-term average retirement income effect scenario with LSD and MSD burden**	**Total net costs with LSD and MSD burden, average retirement income effect scenario**	**Short-term net costs of vaccination with MSD only burden**	**Long-term early retirement income effect scenario with MSD only burden**	**Total net costs with MSD only burden, early retirement income effect scenario**	**Short-term ICERs of vaccination with LSD and MSD burden**	**Total ICERs of vaccination with LSD and MSD burden**	**Short-term ICERs of vaccination with MSD only burden**	**Total ICERs of vaccination with MSD only burden**
**LICs and LMICs only**										
African region	$2 128 410 503	$18 190 037 895	−$16 061 627 393	$3 168 081 088	$4 448 008 377	−$1 279 927 289	$148	Cost-saving	$253	Cost-saving
Region of the Americas	$116 458 342	$387 380 874	−$270 922 531	$139 774 342	$118 090 266	$21 684 076	$1468	Cost-saving	$2076	$1
Eastern Mediterranean region	$1 444 441 843	$4 199 309 262	−$2 754 867 419	$1 705 034 748	$1 170 111 530	$534 923 218	$962	Cost-saving	$1366	$37
European region	$169 596 359	$689 762 644	−$520 166 285	$188 993 601	$194 381 649	−$5 388 048	$6258	Cost-saving	$8568	Cost-saving
South-East Asia Region	$2 558 955 923	$55 541 011 504	−$52 982 055 580	$2 854 852 733	$11 883 800 004	−$9 028 947 271	$3679	Cost-saving	$5207	Cost-saving
Western Pacific region	$876 685 851	$5 763 024 543	−$4 886 338 692	$918 655 357	$1 354 206 683	−$435 551 327	$7167	Cost-saving	$9085	Cost-saving
Gavi-eligible countries	$5 175 827 295	$74 975 327 035	−$69 799 499 741	$6 586 343 447	$16 654 760 176	−$10 068 416 730	$308	Cost-saving	$458	Cost-saving
Global	$7 294 548 821	$84 770 526 722	−$77 475 977 900	$8 975 391 870	$19 168 598 510	−$10 193 206 640	$414	Cost-saving	$596	Cost-saving
**LICs, LMICs, and UMICs**										
African region	$2 342 074 114	$19 054 584 092	−$16 712 509 979	$3 498 838 733	$4 725 160 835	−$1 226 322 102	$161	Cost-saving	$276	Cost-saving
Region of the Americas	$1 172 321 009	$12 749 072 001	−$11 576 750 992	$1 593 900 789	$4 004 414 220	−$2 410 513 431	$4237	Cost-saving	$6913	Cost-saving
Eastern Mediterranean region	$1 844 211 327	$4 977 018 137	−$3 132 806 809	$2 133 757 615	$1 404 033 854	$729 723 761	$1210	Cost-saving	$1687	$50
European region	$711 481 873	$3 317 730 095	−$2 606 248 221	$794 126 013	$975 865 603	−$181 739 590	$14 840	Cost-saving	$20 507	Cost-saving
South-East Asia region	$4 073 465 298	$62 269 412 660	−$58 195 947 362	$4 439 808 108	$13 710 812 232	−$9 271 004 123	$4893	Cost-saving	$6701	Cost-saving
Western Pacific region	$5 507 077 021	$31 336 480 661	−$25 829 403 640	$5 858 927 274	$7 837 815 440	−$1 978 888 166	$22 990	Cost-saving	$30 111	Cost-saving
Gavi-eligible countries	$5 175 827 295	$74 975 327 035	−$69 799 499 741	$6 586 343 447	$16 654 760 176	−$10 068 416 730	$308	Cost-saving	$458	Cost-saving
Global	$15 650 630 642	$133 704 297 646	−$118 053 667 004	$18 319 358 533	$32 658 102 184	−$14 338 743 651	$849	Cost-saving	$1170	Cost-saving

Average retirement age was defined as 64 years as per the OECD, whereas early retirement age was defined as 50 years. ICERs are presented from the companion cost-effectiveness analysis along with new ICERs calculated by combining short-term and long-term benefits; they are shown as 2019 US$ per DALYs averted. Cost-saving indicates that net costs are negative, indicating the costs of vaccination are fully offset by the benefits of vaccination. DALY=disability-adjusted life year. ICER=incremental cost-effectiveness ratio. LSD=less-severe disease. MSD=moderate-to-severe disease. OECD=Organisation for Economic Development and Co-operation.

**Figure 3:**
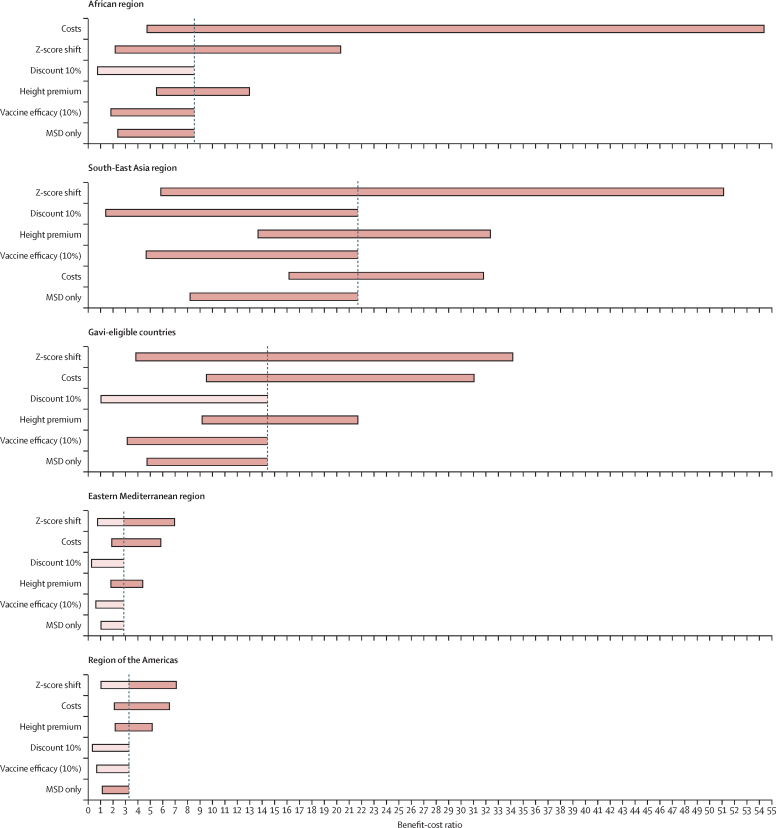
Tornado diagrams showing sensitivity of BCRs to changes in input parameter values for select country groupings The x-axis represents BCR values, with results centred on the base-case BCR for each country grouping. The figure presents sensitivity results compared with results from the average retirement income effect scenario, at 3% discounting, including results from only low-income and lower-middle-income countries. Light coloured bars indicate that the sensitivity range for the variable included results below borderline parity (1·10). BCR=benefit–cost ratio. MSD=moderate-to-severe disease.

Across most groupings, varying the HAZ shift had the largest impact on BCRs, with upper-bound estimates more than doubling base-case results. The lower-bound estimate resulted in BCRs below parity for the Eastern Mediterranean region and the region of the Americas even at 3% discounting (figure 3; appendix p 21). For the African region, vaccine programme net costs were the most influential parameter—the lower-bound estimate resulted in a BCR that was 6·4 times higher than base-case results. Variation in net costs resulted from a combination of wide or skewed distributions of diarrhoeal episodes, vaccine price, vaccine efficacy, and *Shigella*-attributed morbidity fractions from the companion analysis.^19^ Using increased discount rates (10%) resulted in base-case results dropping below parity for all regions except the South-East Asia region (with borderline results for Gavi-eligible countries).

Applying a low (10%) vaccine efficacy scenario lowered base-case BCRs for all regions and brought results below parity in the region of the Americas, the Eastern Mediterranean region, and the European region at 3% discounting, and for all regions except the South-East Asia region (LICs and LMICs only) at 6% discounting. Even at highly conservative vaccine efficacies, BCRs remained above parity for several groups: the African region, South-East Asia region, Western Pacific region, Gavi-eligible countries, and overall (appendix pp 18–19).

When comparing a conservative scenario with vaccination effective only against *Shigella* MSD-attributable disease to the broader base case (effective against LSD and MSD), BCRs in the Eastern Mediterranean region dropped closer to parity. However, BCRs were above parity in all other regions, with benefits more than double the costs in the African, South-East Asia, and Western Pacific regions, and in Gavi-eligible countries (appendix pp 17–18).

## Discussion

This analysis has shown that preventing LGF—a secondary potential outcome of shigellosis not often quantified when examining the health or economic benefits of vaccination—can have substantial returns. Even under conservative assumptions, preventing the long-term economic consequences of *Shigella*-attributable LGF by itself might pay for the vaccine due to future productivity gains in some regions. This finding is solely due to the potential economic benefits of averting growth faltering and does not include other positive health consequences of averting *Shigella* infections. The inclusion of those factors could potentially augment these observed benefits even further.

This benefit–cost analysis complements and extends the results of cost-effectiveness analyses for *Shigella* vaccines. Previous studies have suggested that vaccines might be cost-effective in some countries.^28^ Updated analyses that expanded the burden envelope to include LSD and associated stunting confirmed these results and further suggested that efficacy against stunting could substantially improve vaccine cost-effectiveness in some regions.^19^ The inclusion of potential future productivity benefits in this benefit–cost analysis arising from plausible vaccine-associated reductions in *Shigella*-attributable LGF resulted in vaccination being cost-saving for most regional and country groups examined.

Our complementary economic analyses use different approaches to assess the potential lifelong impact of *Shigella* vaccination, offering complementary information that should be interpreted jointly. Benefits estimates were sensitive to countries’ projected growth rates and the base GNI to which these growth rates were applied. Regions with higher BCRs tended to include countries with larger economies, higher growth rates (appendix pp 12–15), and higher LGF rates. The high BCRs for Gavi-eligible countries resulted from their high growth rates and high total income relative to the regional country groupings. Both studies identified the South-East Asia region as more likely to benefit economically from vaccination than other regions, due in part to its high regional income, high economic growth rates, and high LGF rates.

The importance of economic growth for the present model introduces differences between the findings of this benefit–cost analysis and the companion cost-effectiveness analysis.^19^ For example, although the Eastern Mediterranean region was one of the more cost-effective regions in the companion study, it was the only region that dipped below parity in several conservative scenarios in this study, despite the base-case scenario having a respectable $3 in benefits per $1 in costs. The Eastern Mediterranean region was estimated to have a high diarrhoeal burden and high rates of LGF,^19^ both of which were influential variables in the cost-effectiveness analysis, representing a strong need for intervention. However, on average, countries in the Eastern Mediterranean region had among the lowest incomes and economic growth rates of any region included for analysis. Slower growth among some of these countries than among countries of other regions might have resulted in diminished returns to economic productivity under the conservative assumptions. However, combining short-term and long-term results shows that investing in a vaccine in the Eastern Mediterranean region is justified on the basis of relative burden and net costs.

Although the projected benefits of this analysis are substantial, they manifest over a longer timeframe than benefits traditionally included in the economic analyses of vaccines. After investing in the vaccine programme starting in 2025, the first cohort of vaccinated workers would not begin working until 2040 (at the OECD workforce entry age of 15 years), representing a large time lag for this investment in the health and nutrition of young children. Using India as an example country (the driver of the high benefit–cost results observed for the South-East Asia region), the benefits accrued in the first 20 years of the programme, while the percentage of the workforce that had been vaccinated was slowly increasing, represent 10% of total programme benefits at 3% discounting. This percentage slowly builds over time; after 40 years, when all vaccinated cohorts have been in the workforce for 20 years, 43% of total benefits have been produced. Considered another way, at 3% discounting and including only the income effect, it would take around 14 years for the programme to pay for itself in India (ie, for the benefit–cost ratio to reach parity). Whether this distribution of benefits will be attractive to finance ministers depends on many factors, including the opportunity costs of current investments and prevailing discount rates within a specific country at any given time.

Our height-based productivity model was most sensitive to estimated HAZ shifts for most regions, similar to past iterations of this model.^10^ Many of the input variables for the HAZ shift (eg, regional aetiological fractions and *Shigella* diarrhoeal rates) had wide confidence or uncertainty intervals, introducing variation between upper-bound and lower-bound BCRs.

Clinical trials have not directly measured vaccine efficacy against LGF; accordingly, vaccine efficacy remains an uncertain parameter in this model and in general. There is no current evidence regarding whether averting *Shigella*-attributable diarrhoea through vaccination reduces LGF or stunting. The key hypothesised pathway for stunting is chronic inflammation resulting from enteric infections, regardless of whether diarrhoeal disease occurs. *Shigella* vaccines might prevent diarrhoeal disease without preventing chronic inflammation, which could result in minimal impact on LGF. Existing observational research from Bangladesh indicated no nutritional benefit from a rotavirus vaccine;^29^ however, double-blind studies are needed to estimate these effects with more accuracy and, unlike *Shigella*, rotavirus has not been linked to growth faltering.^8^ This data gap highlights the importance of clinical studies measuring vaccine effect on LGF to better understand this association and the health and economic benefits of vaccination. Evaluation of clinical trials, vaccine delivery scheduling, and the age distribution of *Shigella* burden (ie, higher burden in children aged 2 years and 3–5 years than in other age groups) is necessary to test the current assumption of delivery within the existing routine schedule.^20^ If delivery recommendations do not align with routine scheduling, each could incur slightly increased costs and alter the impacts of the vaccine on LGF.

This analysis has several strengths, including being the first attempt, to our knowledge, to quantify the economic benefits of improving linear growth by reducing *Shigella* infections. However, our approach also has limitations. This model is based on publicly available data, which have their own limitations. The GNI data used to model productivity gains might not adequately represent wages in LMICs, partly because they exclude those in the informal labour force engaging in subsistence activities who might be paid in-kind. Exclusion of these workers could bias wage data and represent an important limitation of this analysis. Additionally, the OECD working age was applied uniformly in our model. However, many workers in LMICs might engage in the labour force for fewer years on average than workers in higher-income countries—our conservative early retirement scenario might better represent the workers in these settings. Furthermore, gender-related differences in economic productivity are not accounted for in this study. Although the benefits estimated from this analysis are applied to the entire population of the included countries, it is probable that benefits would actually differ by several factors, including age, residence location (eg, urban or rural), and socioeconomic status, among others. Additional epidemiological data on shigellosis would be needed to discern these differences. Future iterations of this model could refine these elements. Other potential refinements in future models could include updating the model to quantify the contribution of asymptomatic *Shigella* infections to growth faltering, as the availability of data on global asymptomatic infections improves over time. Furthermore, we use rotavirus inpatient and outpatient costs as we are not aware of a data source that would give representative estimates of *Shigella* illness costs that could be applied to all of the regions in our model. This approach could be adjusted in future analyses as more data become available. Finally, we use a comparator of no vaccination; another possible comparator could be a strategy involving rapid diagnosis and treatment, which would require a rapidly implementable sensitive molecular diagnostic test coupled with appropriate antibiotic treatment. This strategy would have the benefit of targeting treatment to those most in need but would be challenging to implement in LMICs.

In the course of this modelling work, we made assumptions to set parameter values on the basis of sparse available data, which might have led to our benefit–cost outcomes being over-estimates or under-estimates of the true benefit–cost of vaccination. For example, the model focuses only on height-related benefits of reducing *Shigella* but not on benefits associated with decreasing direct *Shigella* burden (morbidity and mortality). Therefore, these results do not thoroughly account for the benefits of *Shigella* vaccination. Future research could address this gap. Additionally, rolling out a population-wide vaccine for a common childhood illness would probably improve linear growth across populations and could have other important spillover effects, such as beneficial effects on national unemployment rates through indirectly improving education and job creation. However, we have tried to estimate some of the other positive indirect effects via use of the MPC in our multiplier effects scenarios. Yet we do not account for general equilibrium effects of improving population growth and overall health in children or their families and this might contribute to our analysis presenting an under-estimate of the benefit–costs of vaccination. Along the same lines, we do not fully address all potential medium-term and long-term benefits of *Shigella* vaccination. For example, vaccination can potentially reduce antibiotic use and thus slow antibiotic resistance. Finally, we exclude partial vaccination, although even partial vaccination might result in some protection against shigellosis and hence LGF; therefore, this exclusion might result in an under-estimation of benefits. Findings from our analysis suggest that similar models should be used for enteric diseases more often. This analysis further augmented a *Shigella* vaccine's economic potential by showing it to be a cost-beneficial, and often cost-saving, intervention in the longer term. Furthermore, given that children with LGF often come from the most financially vulnerable settings, increasing their incomes would enhance both health and economic equity. Accounting for growth-related benefits for pathogens associated with LGF can result in greatly improved valuation of interventions. Accounting for broader vaccine benefits better informs decision-making and priority-setting in addressing national and global disease burden. Further research is needed to fill the evidence gap on *Shigella* vaccine efficacy against LGF, to enable more accurate estimates of broader benefits related to long-term height-related productivity.

## Data sharing

Data used in this study were obtained from publicly available datasets. No participant data were collected or used. We believe we have described the model generated for this study in adequate detail in the Article for it to be replicated for the purpose of other future studies. There are no plans to make this model publicly available.

For the **estimates from the 2019 Global Burden of Disease Study** see http://ghdx.healthdata.org/gbd-results-tool

For **economic data from the World Bank** see https://databank.worldbank.org/source/world-development-indicators

For **growth rate projection from the International Monetary Fund** see https://www.imf.org/en/Publications/SPROLLs/world-economic-outlook-databases

For **estimates of national immunisation coverage** see https://www.who.int/teams/immunization-vaccines-and-biologicals/immunization-analysis-and-insights/global-monitoring/immunization-coverage/who-unicef-estimates-of-national-immunization-coverage

For **vaccine price estimates** see https://www.who.int/teams/immunization-vaccines-and-biologicals/vaccine-access/mi4a/mi4a-vaccine-purchase-data

## Declaration of interests

We declare no competing interests.
