# Supplementary material for: Projecting the long-term economic benefits of reducing Shigella-attributable linear growth faltering with a potential vaccine: a modelling study
Source: Lancet Glob Health. 2023 May 16;11(6):e892–902. doi: 10.1016/S2214-109X(23)00050-5 (PMC10205973; doi:10.1016/S2214-109X(23)00050-5)
Supplement: Supplementary appendix [file mmc1.pdf]

THE LANCET
Global Health

Supplementary appendix

This appendix formed part of the original submission and has been peer reviewed. We post it as supplied by the authors.

Supplement to: Puett C, Anderson JD IV, Bagamian KH, et al. Projecting the long-term economic benefits of reducing *Shigella*-attributable linear growth faltering with a potential vaccine: a modelling study. *Lancet Glob Health* 2023; **11:** e892–902.

Supplementary Materials

Contents

Countries included in the model for analysis 2
Literature review for estimates of the height premium 3
Optimistic scenario description 3
Review of estimates for the Marginal Propensity to Consume (MPC) 4
Separate benefits and costs results 5
Base case results including 10% discount scenario 7
Country costs and benefits as % of regional totals 8
Sensitivity analyses, full results 16
Comparison with nutrition intervention results from similar model 22
Companion cost-effectiveness analysis 24
References 79

Countries included in the model for analysis

Table S1. Countries included in model, categorized by WHO region, World Bank income category and eligibility for Gavi support

	AFRO		AMRO	EMRO	EURO	SEARO	WPRO
**LIC**	Burkina Faso*<br>Burundi*<br>Central African Republic*<br>Chad*<br>Congo, Dem. Rep.*<br>Eritrea*<br>Ethiopia*<br>The Gambia*<br>Guinea*<br>Guinea-Bissau*	Liberia*<br>Madagascar*<br>Malawi*<br>Mali*<br>Mozambique*<br>Niger*<br>Rwanda*<br>Sierra Leone*<br>Togo*<br>Uganda*	Haiti*	Afghanistan*<br>Somalia*<br>Sudan*<br>Yemen, Rep.*	Tajikistan*	..	..
**LMIC**	Algeria<br>Angola<br>Benin*<br>Cabo Verde<br>Cameroon*<br>Comoros*<br>Congo, Rep.*<br>Cote d'Ivoire*<br>Eswatini	Ghana*<br>Kenya*<br>Lesotho*<br>Mauritania*<br>Nigeria*<br>Senegal*<br>Tanzania*<br>Zambia*<br>Zimbabwe*	Bolivia<br>El Salvador<br>Honduras<br>Nicaragua*	Djibouti*<br>Egypt, Arab Rep.<br>Morocco<br>Pakistan*<br>Tunisia	Kyrgyz Republic*<br>Ukraine<br>Uzbekistan*	Bangladesh*<br>Bhutan<br>India*<br>Myanmar*<br>Nepal*<br>Sri Lanka<br>Timor-Leste	Cambodia*<br>Kiribati<br>Lao PDR*<br>Mongolia<br>Papua New Guinea*<br>Philippines<br>Solomon Islands*<br>Vanuatu<br>Vietnam
**UMIC**	Botswana<br>Gabon<br>Namibia<br>South Africa		Argentina<br>Brazil<br>Colombia<br>Costa Rica<br>Dominican Republic<br>Ecuador<br>Guatemala<br>Jamaica<br>Mexico<br>Paraguay<br>Peru<br>Suriname	Iraq<br>Jordan	Armenia<br>Azerbaijan<br>Belarus<br>Bulgaria<br>Georgia<br>Kazakhstan<br>Turkey	Indonesia<br>Thailand	China<br>Fiji<br>Malaysia

* Gavi-eligible; AFRO: African region, AMRO: Region of the Americas, EMRO: Eastern Mediterranean Region, Gavi: Global Alliance for Vaccines and Immunisation, SEARO: Southeast Asian Region, WPRO: Western Pacific Region. LIC: low-income country, LMIC: lower-middle income country, UMIC: upper-middle income country

Literature review for estimates of the height premium

To inform the height premium estimate included in this model, we performed a review of the economic literature. This review is described in detail in a manuscript being prepared for publication. In summary, we based our review on the only published paper of which we are aware dealing with linear growth/stunting and economic outcomes.[1] We identified a subset of the papers included in this prior review which presented the semi-elasticity of the height premium, or a percent change in wages per additional unit (cm/inch) of height. Median estimates were calculated overall and disaggregated by country income category.

Optimistic scenario description

The height premium employed in the optimistic scenario represented growth faltering on the basis of z scores, a relative measure, expressed as the percentage increase in income per z score gained in height. In this scenario we bypass step four in our analytical flow chart (Figure 1) and apply the income change per z score empirical estimate to our country-specific z score shift. This scenario uses empirical evidence of income increases documented in Cohort studies from Brazil and Guatemala reporting an average of 8% increase in income per z score.[2] This represents a larger increase than the estimates used in our average and conservative scenarios and could be interpreted as an optimistic scenario. However, the context around these findings, in terms of timing of the interventions (1970s and 80s) and the geographic settings represented in these studies may be difficult to replicate or to generalize across regions and country income categories.

Table S2: Base case parameters and definitions for the three modeled scenarios, including an optimistic scenario

	Average retirement	Early retirement	Optimistic
Working age	15-64 (OECD)	15-50 (early retirement)	15-64 (OECD)
Height premium	Absolute measure (cm), median country-income category-specific<br>LIC – 0·87% (median of 2 studies from LIC, 6 from LMICs)<br>LMIC – 0·83% (median of 6 studies from LMIC)<br>UMIC – 0·87% (median of 6 studies from LMIC, 2 from UMICs)<br>HIC – 0·26% (median of 13 studies from HICs)	Same values as for average scenario	Relative measure (HAZ)[2] from Brazil and Guatemala<br>8·0% per 1 z score
Discount rates	3%, 6%, 10%	3%, 6%, 10%	3%, 6%, 10%
Z score shifts	Average estimate per country	Average estimate per country	Average estimate per country
Costs	Average estimate per country	Average estimate per country	Average estimate per country

Review of estimates for the Marginal Propensity to Consume (MPC)

Table S3. Sources consulted in the literature review on MPC estimates for different regions and income levels

Country	MPC	Region	Income level	Reference
Thailand	0·025	East Asia & Pacific	Upper middle income	Rungcharoenkitkul 2011[3]
Advanced economies (US)	0·038	North America	High Income	IMF 2009[4]
Italy	0·48	Europe & Central Asia	High income	Jappelli & Pistaferri 2014[5]
Jordan	0·531	Middle East & North Africa	Upper middle income	Glytsos 2002[6]
India	0·6	South Asia	Lower middle income	Reserve Bank of India 2002[7]
India	0·72	South Asia	Lower middle income	Khan et al 2015[8]
Morocco	0·607	Middle East & North Africa	Lower middle income	Glytsos 2002[6]
Portugal	0·667	Europe & Central Asia	High income	Glytsos 2002[6]
Kyrgyzstan	0·68	Europe & Central Asia	Lower middle income	Aitymbetov 2006[9]
South Africa	0·7	Sub-Saharan Africa	Upper middle income	Bengtsson 2012[10]
Nigeria	0·72	Sub-Saharan Africa	Lower middle income	Achoja 2013[11]
Egypt	0·735	Middle East & North Africa	Lower middle income	Glytsos 2002[6]
Bangladesh	0·75	South Asia	Lower middle income	Khan et al 2015[8]
Malaysia	0·81	East Asia & Pacific	Upper middle income	Murugasu et al 2013[12]
Malaysia	0·25	East Asia & Pacific	Upper middle income	Murugasu et al 2013[12]
Pakistan	0·82	South Asia	Lower middle income	Khan et al 2015[8]
Pakistan	0·63	South Asia	Lower middle income	Javed et al 2005[13]
Greece	0·847	Europe & Central Asia	High income	Glytsos 2002[6]
Nepal	0·89	South Asia	Lower middle income	Khan et al 2015[8]
Sri Lanka	0·96	South Asia	Lower middle income	Khan et al 2015[8]

MPC = marginal propensity to consume.

Table S4. Findings from the literature review on MPC estimates per country income category

Category	Average	Count
High Income	0·508	4
Upper middle income	0·463	5
Lower middle income	0·737	11

MPC = marginal propensity to consume.

Separate benefits and costs results

Table S5. Economic benefits of reduced *Shigella*-attributable linear growth faltering (in billions of dollars) by WHO region and country groupings for base case model scenarios

	Average scenario, OECD working age						Conservative, early retirement (age 50)						Optimistic, z-score height premium					
	Income effect			Multiplier effect			Income effect			Multiplier effect			Income effect			Multiplier effect		
Discounting	3%	6%	10%	3%	6%	10%	3%	6%	10%	3%	6%	10%	3%	6%	10%	3%	6%	10%
**LICs & LMICs Only**																		
AFRO	18·19	3·58	0·58	49·10	10·36	1·83	10·63	2·65	0·52	31·18	8·14	1·67	63·63	12·57	2·06	173·01	36·65	6·52
AMRO	0·39	0·09	0·02	1·47	0·34	0·07	0·28	0·08	0·02	1·06	0·29	0·06	1·39	0·32	0·06	5·30	1·22	0·24
EMRO	4·20	0·90	0·16	11·59	2·56	0·48	2·78	0·72	0·15	7·82	2·08	0·44	14·70	3·17	0·57	40·89	9·03	1·68
EURO	0·69	0·15	0·03	2·62	0·57	0·10	0·46	0·12	0·02	1·75	0·46	0·09	2·48	0·54	0·10	9·44	2·05	0·37
SEARO	55·54	9·94	1·43	103·86	18·63	2·69	28·20	6·63	1·20	52·83	12·45	2·26	190·82	34·16	4·92	356·90	64·02	9·26
WPRO	5·76	1·09	0·17	10·85	2·07	0·32	3·22	0·78	0·15	6·10	1·49	0·28	19·81	3·76	0·58	37·32	7·13	1·12
GAVI	74·98	13·77	2·05	157·79	29·98	4·70	39·58	9·47	1·75	87·05	21·35	4·09	258·89	47·63	7·12	547·16	104·22	16·38
Global	84·77	15·76	2·39	179·50	34·52	5·50	45·55	10·98	2·05	100·74	24·90	4·82	292·84	54·52	8·29	622·86	120·11	19·19
**LICs, LMICs & UMICs**																		
AFRO	19·05	3·79	0·63	50·71	10·76	1·92	11·29	2·83	0·56	32·42	8·49	1·75	66·60	13·30	2·21	178·54	38·02	6·81
AMRO	12·75	3·03	0·60	24·49	5·81	1·16	9·43	2·60	0·57	18·09	4·99	1·09	45·14	10·62	2·10	86·77	20·41	4·02
EMRO	4·98	1·08	0·20	13·04	2·89	0·54	3·33	0·87	0·18	8·86	2·36	0·50	17·38	3·78	0·69	45·87	10·17	1·90
EURO	3·32	0·74	0·14	7·52	1·68	0·31	2·31	0·62	0·13	5·18	1·38	0·29	11·51	2·58	0·49	26·25	5·85	1·09
SEARO	62·27	11·36	1·68	116·39	21·27	3·16	32·53	7·75	1·43	60·90	14·53	2·68	213·93	39·04	5·78	399·94	73·10	10·86
WPRO	31·34	6·21	1·02	58·48	11·61	1·90	18·62	4·64	0·90	34·79	8·67	1·69	313·59	62·58	10·31	584·41	116·67	19·23

GAVI	74·98	13·77	2·05	157·79	29·98	4·70	39·58	9·47	1·75	87·05	21·35	4·09	258·89	47·63	7·12	547·16	104·22	16·38
Global	133·70	26·22	4·26	270·62	54·01	8·98	77·51	19·31	3·77	160·24	40·42	8·01	668·15	131·91	21·56	1,321·77	264·22	43·91

AFRO: African region, AMRO: Region of the Americas, EMRO: Eastern Mediterranean Region, GAVI: Global Alliance for Vaccines and Immunisation, LIC: low-income country, LMIC: lower-middle income country, OECD: Organisation for Economic Co-operation and Development, SEARO: Southeast Asian Region, UMIC: upper-middle income country, WPRO: Western Pacific Region

Table S6. Net costs of *Shigella* vaccine (in billions of dollars) of the Shigella vaccine program, by WHO region, World Bank income category grouping, and discount rate scenario

Discounting	3%	6%	10%
**LICs & LMICs Only**			
AFRO	2·13	1·36	0·79
AMRO	0·12	0·08	0·04
EMRO	1·45	0·93	0·55
EURO	0·17	0·11	0·07
SEARO	2·56	1·67	1·00
WPRO	0·88	0·57	0·34
GAVI	5·19	3·35	1·98
Global	7·31	4·72	2·79
**LICs, LMICs & UMICs**			
AFRO	2·35	1·50	0·87
AMRO	1·17	0·76	0·45
EMRO	1·85	1·19	0·70
EURO	0·71	0·46	0·28
SEARO	4·08	2·66	1·59
WPRO	5·52	3·60	2·16
GAVI	5·19	3·35	1·98
Global	15·68	10·18	6·05

AFRO: African region, AMRO: Region of the Americas, EMRO: Eastern Mediterranean Region, GAVI: Global Alliance for Vaccines and Immunisation, LIC: low-income country, LMIC: lower-middle income country, OECD: Organisation for Economic Co-operation and Development, SEARO: Southeast Asian Region, UMIC: upper-middle income country, WPRO: Western Pacific Region

Base case results including 10% discount scenario

Across all country groupings, base case benefit-cost results were above parity at 3% discounting, but fewer showed positive returns at 10%. The use of higher discount rates is controversial as it favors front-loaded projects with immediate returns (e.g., building roads) compared to interventions whose benefits take longer to materialize. Lower discount rates are generally accepted for interventions yielding important benefits that improve equity for future generations.[14] Previous analyses in the nutrition field have used a maximum of 5-6% discounting,[15–17] though higher rates commonly are employed for private sector projects and these rates have made their way into public sector analyses.[18] The higher rates employed in the present analysis should be viewed as conservative measures to account for different levels of uncertainty in the future, and provide a test of which regions might perform best under very stringent circumstances.

Table S7. Base case benefit cost ratios based on productivity benefits of preventing growth faltering due to *Shigella*, including 10% discount scenario

	Average scenario						Conservative, early retirement (age 50)						Optimistic, z-score height premium					
	Income effect			Multiplier effect			Income effect			Multiplier effect			Income effect			Multiplier effect		
Discounting	3%	6%	10%	3%	6%	10%	3%	6%	10%	3%	6%	10%	3%	6%	10%	3%	6%	10%
**LICs & LMICs Only**																		
AFRO	8·52	2·63	0·74	23·00	7·62	2·32	4·98	1·95	0·65	14·61	5·99	2·12	29·81	9·25	2·60	81·06	26·96	8·26
AMRO	3·32	1·18	0·38	12·62	4·49	1·46	2·38	1·00	0·36	9·06	3·79	1·37	11·94	4·25	1·38	45·38	16·15	5·26
EMRO	2·90	0·97	0·29	8·01	2·73	0·86	1·92	0·77	0·27	5·40	2·22	0·80	10·16	3·39	1·03	28·25	9·66	3·05
EURO	4·06	1·36	0·41	15·43	5·15	1·57	2·70	1·09	0·38	10·27	4·15	1·45	14·61	4·88	1·49	55·57	18·56	5·65
SEARO	21·67	5·95	1·43	40·51	11·14	2·70	11·00	3·97	1·20	20·61	7·45	2·26	74·43	20·44	4·93	139·22	38·30	9·27
WPRO	6·56	1·92	0·50	12·35	3·63	0·96	3·66	1·37	0·43	6·95	2·61	0·83	22·55	6·59	1·72	42·48	12·51	3·29
GAVI	14·45	4·11	1·04	30·42	8·95	2·38	7·63	2·83	0·89	16·78	6·38	2·07	49·91	14·22	3·60	105·48	31·13	8·29
Global	11·60	3·34	0·86	24·55	7·31	1·97	6·23	2·32	0·74	13·78	5·27	1·73	40·06	11·55	2·97	85·20	25·44	6·88
**LICs, LMICs & UMICs**																		
AFRO	8·11	2·53	0·72	21·59	7·18	2·20	4·81	1·89	0·64	13·80	5·67	2·01	28·36	8·88	2·53	76·02	25·38	7·81
AMRO	10·85	3·97	1·33	20·85	7·62	2·55	8·02	3·41	1·26	15·40	6·54	2·41	38·43	13·93	4·62	73·87	26·76	8·87
EMRO	2·69	0·91	0·28	7·06	2·42	0·77	1·80	0·73	0·26	4·79	1·98	0·71	9·40	3·17	0·98	24·81	8·53	2·70
EURO	4·65	1·61	0·51	10·54	3·61	1·13	3·23	1·33	0·47	7·27	2·98	1·05	16·15	5·57	1·75	36·83	12·62	3·95
SEARO	15·26	4·27	1·06	28·52	8·00	1·99	7·97	2·91	0·90	14·92	5·46	1·69	52·42	14·68	3·64	98·00	27·49	6·84
WPRO	5·68	1·73	0·47	10·60	3·22	0·88	3·38	1·29	0·42	6·31	2·41	0·78	56·84	17·38	4·78	105·93	32·40	8·92
GAVI	14·45	4·11	1·04	30·42	8·95	2·38	7·63	2·83	0·89	16·78	6·38	2·07	49·91	14·22	3·60	105·48	31·13	8·29
Global	8·53	2·58	0·70	17·26	5·31	1·49	4·94	1·90	0·62	10·22	3·97	1·32	42·61	12·96	3·56	84·29	25·96	7·26

Note: Results ≤1.10 are highlighted, representing ratios that are below parity or borderline. AFRO: African region, AMRO: Region of the Americas, EMRO: Eastern Mediterranean Region, GAVI: Global Alliance for Vaccines and Immunisation, LIC: low-income country, LMIC: lower-middle income country, OECD:

Organisation for Economic Co-operation and Development, SEARO: Southeast Asian Region, UMIC: upper-middle income country, WPRO: Western Pacific Region

Country costs and benefits as % of regional totals

Table S8. Country costs as % of regional totals

	LIC+LMIC	+UMIC
**AFRO**		
Algeria	6·7%	6·1%
Angola	7·3%	6·7%
Benin	1·4%	1·3%
Botswana	··	0·4%
Burkina Faso	2·0%	1·9%
Burundi	1·3%	1·2%
Cabo Verde	0·1%	0·1%
Cameroon	2·4%	2·1%
Central African Republic	0·2%	0·2%
Chad	1·0%	1·0%
Comoros	0·1%	0·1%
Congo, Dem. Rep.	7·5%	6·8%
Congo, Rep.	0·5%	0·4%
Cote d'Ivoire	2·9%	2·6%
Eritrea	0·3%	0·3%
Eswatini	0·2%	0·2%
Ethiopia	7·8%	7·1%
Gabon	··	0·4%
Gambia, The	0·2%	0·2%
Ghana	2·9%	2·6%
Guinea	0·8%	0·7%
Guinea-Bissau	0·2%	0·1%
Kenya	4·7%	4·3%
Lesotho	0·1%	0·1%

Liberia	0·4%	0·4%
Madagascar	2·3%	2·1%
Malawi	1·9%	1·7%
Mali	2·0%	1·8%
Mauritania	0·5%	0·4%
Mozambique	3·1%	2·8%
Namibia	··	0·7%
Niger	3·5%	3·1%
Nigeria	15·4%	14·0%
Rwanda	1·0%	0·9%
Senegal	1·9%	1·8%
Sierra Leone	0·6%	0·5%
South Africa	··	7·7%
Tanzania	8·0%	7·2%
Togo	0·7%	0·7%
Uganda	4·3%	3·9%
Zambia	2·4%	2·2%
Zimbabwe	1·4%	1·3%
**AMRO**		
Argentina	··	8·7%
Bolivia	35·6%	3·5%
Brazil	··	38·1%
Colombia	··	5·9%
Costa Rica	··	0·5%
Dominican Republic	··	2·3%
Ecuador	··	3·8%
El Salvador	17·9%	1·8%
Guatemala	··	6·2%
Haiti	7·8%	0·8%
Honduras	31·4%	3·1%

Jamaica	··	0·5%
Mexico	··	15·4%
Nicaragua	7·3%	0·7%
Paraguay	··	2·0%
Peru	··	6·5%
Suriname	··	0·1%
**EMRO**		
Afghanistan	5·37%	4·21%
Djibouti	0·10%	0·08%
Egypt, Arab Rep.	39·20%	30·70%
Iraq	··	18·77%
Jordan	··	2·91%
Morocco	9·47%	7·42%
Pakistan	33·24%	26·04%
Somalia	1·88%	1·47%
Sudan	6·02%	4·72%
Tunisia	2·30%	1·80%
Yemen, Rep.	2·41%	1·89%
**EURO**		
Armenia	··	1·3%
Azerbaijan	··	5·5%
Belarus	··	3·9%
Bulgaria	··	2·1%
Georgia	··	1·9%
Kazakhstan	··	13·8%
Kyrgyz Republic	8·4%	2·0%
Tajikistan	13·5%	3·2%
Turkey	··	47·6%
Ukraine	45·4%	10·8%

Uzbekistan	32·7%	7·8%
**SEARO**		
Bangladesh	10·1%	6·4%
Bhutan	0·1%	0·1%
India	81·3%	51·1%
Indonesia	··	32·6%
Myanmar	3·4%	2·1%
Nepal	1·9%	1·2%
Sri Lanka	2·9%	1·8%
Thailand	··	4·6%
Timor-Leste	0·3%	0·2%
**WPRO**		
Cambodia	3·8%	0·6%
China	··	81·2%
Fiji	··	0·1%
Kiribati	0·1%	0·0%
Lao PDR	1·6%	0·3%
Malaysia	··	2·8%
Mongolia	1·9%	0·3%
Papua New Guinea	1·2%	0·2%
Philippines	54·9%	8·7%
Solomon Islands	0·2%	0·0%
Vanuatu	0·2%	0·0%
Vietnam	36·0%	5·7%

Note: At 3% discounting, including income effect only. AFRO: African region, AMRO: Region of the Americas, EMRO: Eastern Mediterranean Region, GAVI: Global Alliance for Vaccines and Immunisation, LIC: low-income country, LMIC: lower-middle income country, OECD: Organisation for Economic Co-operation and Development, SEARO: Southeast Asian Region, UMIC: upper-middle income country, WPRO: Western Pacific Region

Table S9. Country economic variables: benefits as % of regional totals, growth rate and 2019 GNI

	% of total per scenario				
	**Average**	**Conservative**	**Optimistic**	**Growth rate**	**2019 GNI (Billions)**
**AFRO**					
Algeria	1·7%	2·4%	1·8%	0·63%	166·89
Angola	1·4%	1·7%	1·4%	1·95%	81·30
Benin*	1·9%	1·8%	1·9%	5·15%	14·25
Botswana	0·9%	1·0%	0·9%	3·82%	16·95
Burkina Faso*	1·2%	1·3%	1·2%	4·32%	15·33
Burundi*	0·1%	0·1%	0·1%	2·28%	3·02
Cabo Verde	0·2%	0·2%	0·2%	5·07%	1·94
Cameroon*	2·1%	2·3%	2·1%	3·80%	38·26
Central African Republic*	0·1%	0·1%	0·1%	3·72%	2·39
Chad*	0·2%	0·3%	0·2%	2·35%	11·14
Comoros*	0·0%	0·0%	0·0%	2·12%	1·17
Congo, Dem. Rep.*	2·2%	2·2%	2·5%	3·67%	48·96
Congo, Rep.*	0·1%	0·2%	0·1%	1·00%	9·53
Cote d'Ivoire*	7·8%	7·6%	7·7%	5·22%	56·98
Eritrea*	0·1%	0·1%	0·1%	2·73%	2·19
Eswatini	0·0%	0·1%	0·0%	0·63%	4·02
Ethiopia*	22·0%	19·4%	21·8%	6·15%	95·32
Gabon	0·3%	0·4%	0·3%	2·12%	15·51
The Gambia*	0·2%	0·2%	0·2%	5·23%	1·80
Ghana*	5·6%	5·9%	5·6%	4·30%	65·53
Guinea*	0·6%	0·6%	0·6%	4·13%	12·18
Guinea-Bissau*	0·1%	0·1%	0·1%	3·50%	1·44
Kenya*	13·2%	12·7%	13·0%	5·28%	93·58
Lesotho*	0·1%	0·1%	0·1%	2·08%	2·81
Liberia*	0·2%	0·2%	0·2%	4·02%	2·72

Madagascar*	0·8%	0·9%	0·8%	3·80%	13·60
Malawi*	0·7%	0·8%	0·7%	4·62%	7·49
Mali*	1·0%	1·1%	1·1%	4·00%	16·75
Mauritania*	0·4%	0·4%	0·4%	3·73%	7·50
Mozambique*	3·3%	2·9%	3·4%	6·20%	15·00
Namibia	0·2%	0·3%	0·2%	1·73%	12·02
Niger*	5·9%	4·7%	5·9%	7·25%	13·48
Nigeria*	5·3%	6·9%	5·5%	1·30%	433·45
Rwanda*	2·2%	2·0%	2·2%	5·93%	10·01
Senegal*	5·8%	5·1%	5·7%	6·30%	23·08
Sierra Leone*	0·2%	0·2%	0·2%	3·22%	4·07
South Africa	3·1%	4·2%	3·1%	0·73%	341·52
Tanzania*	3·5%	3·9%	3·6%	3·67%	61·58
Togo*	0·4%	0·4%	0·4%	3·92%	5·51
Uganda*	4·2%	4·2%	4·4%	5·12%	34·25
Zambia*	0·2%	0·3%	0·2%	0·32%	22·91
Zimbabwe*	0·4%	0·5%	0·4%	1·85%	19·84
**AMRO**					
Argentina	7·4%	7·7%	7·2%	1·53%	427·61
Bolivia	1·5%	1·4%	1·6%	3·02%	40·08
Brazil	29·5%	30·6%	28·6%	1·40%	1,790·97
Colombia	12·2%	11·4%	11·8%	2·98%	313·49
Costa Rica	1·5%	1·5%	1·4%	2·10%	58·19
Dominican Republic	2·9%	3·1%	5·7%	4·08%	84·67
Ecuador	1·6%	1·7%	1·5%	1·05%	104·41
El Salvador	0·5%	0·5%	0·5%	1·65%	25·72
Guatemala	2·8%	2·7%	2·8%	2·75%	75·44
Haiti*	0·1%	0·1%	0·1%	0·17%	14·38
Honduras	0·7%	0·7%	0·8%	2·75%	23·18
Jamaica	0·4%	0·4%	0·4%	2·17%	16·05

Mexico	25·1%	25·7%	24·3%	1·68%	1,232·37
Nicaragua*	0·2%	0·2%	0·2%	0·93%	12·05
Paraguay	1·4%	1·3%	1·3%	2·75%	36·84
Peru	12·3%	10·9%	11·9%	3·75%	216·27
Suriname	0·0%	0·0%	0·0%	0·37%	3·28
**EMRO**					
Afghanistan*	1·6%	1·6%	1·5%	3·17%	19·60
Djibouti*	0·6%	0·6%	0·6%	4·75%	3·23
Egypt, Arab Rep.	45·9%	43·5%	45·2%	4·17%	292·08
Iraq	13·8%	14·6%	13·5%	2·73%	232·98
Jordan	1·9%	2·1%	1·8%	1·95%	44·51
Morocco	8·2%	8·6%	8·4%	2·80%	117·37
Pakistan*	23·6%	24·2%	24·3%	3·17%	272·61
Somalia*	0·2%	0·2%	0·2%	2·55%	4·91
Sudan*	1·1%	1·2%	1·1%	1·73%	28·89
Tunisia	1·0%	1·2%	1·1%	1·27%	37·48
Yemen, Rep.*	2·3%	2·2%	2·2%	3·67%	22·57
**EURO**					
Armenia	0·8%	0·8%	0·8%	2·90%	13·90
Azerbaijan	1·2%	1·4%	1·2%	0·80%	45·99
Belarus	1·2%	1·5%	1·2%	0·10%	61·21
Bulgaria	4·2%	4·3%	4·2%	2·62%	67·45
Georgia	2·1%	1·9%	2·0%	4·07%	17·04
Kazakhstan	12·2%	12·3%	12·1%	2·90%	158·97
Kyrgyz Republic*	0·7%	0·7%	0·7%	3·50%	7·84
Tajikistan*	0·9%	0·9%	0·9%	3·25%	9·45
Turkey	57·5%	57·9%	56·9%	2·92%	748·61
Ukraine	9·5%	9·7%	9·9%	2·77%	158·53
Uzbekistan*	9·7%	8·7%	10·1%	4·38%	58·79

**SEARO**					
Bangladesh*	7·4%	7·6%	7·4%	5·93%	316·91
Bhutan	0·0%	0·0%	0·0%	3·77%	2·30
India*	81·1%	78·1%	81·1%	6·67%	2,837·69
Indonesia	8·7%	10·5%	8·7%	4·27%	1,085·71
Myanmar*	0·1%	0·2%	0·1%	1·05%	73·29
Nepal*	0·2%	0·2%	0·2%	3·82%	31·00
Sri Lanka	0·4%	0·5%	0·4%	3·13%	81·59
Thailand	2·1%	2·8%	2·1%	2·78%	523·68
Timor-Leste	0·0%	0·0%	0·0%	2·02%	2·70
**WPRO**					
Cambodia*	0·5%	0·5%	0·2%	5·10%	25·53
China	79·9%	81·0%	91·8%	4·78%	14,246·12
Fiji	0·1%	0·1%	0·0%	4·38%	5·05
Kiribati	0·0%	0·0%	0·0%	1·12%	0·38
Lao PDR*	0·3%	0·3%	0·1%	4·65%	17·75
Malaysia	1·6%	1·7%	1·9%	4·58%	354·96
Mongolia	0·2%	0·2%	0·1%	4·58%	12·42
Papua New Guinea*	0·0%	0·1%	0·0%	2·07%	23·78
Philippines	9·8%	9·3%	3·4%	5·57%	414·27
Solomon Islands*	0·0%	0·0%	0·0%	2·25%	1·57
Vanuatu	0·0%	0·0%	0·0%	2·45%	0·94
Vietnam	7·5%	7·0%	2·6%	5·77%	246·72

Note: At 3% discounting, for results from the average scenario including income effect only and results from UMICs as well as LIC and LMIC.
* GAVI-eligible. AFRO: African region, AMRO: Region of the Americas, EMRO: Eastern Mediterranean Region, GAVI: Global Alliance for Vaccines and Immunisation, GNI = gross national income. LIC: low-income country, LMIC: lower-middle income country, OECD: Organisation for Economic Co-operation and Development, SEARO: Southeast Asian Region, UMIC: upper-middle income country, WPRO: Western Pacific Region

Sensitivity analyses, full results

Table S10. Sensitivity analysis for the height premium estimate, showing benefit cost ratios based on productivity benefits of reducing linear growth faltering attributable to *Shigella*, including 10% discount scenario

	Average scenario						Conservative scenario, early retirement (age 50)					
	Income effect			Multiplier effect			Income effect			Multiplier effect		
Discounting	3%	6%	10%	3%	6%	10%	3%	6%	10%	3%	6%	10%
Worst case												
**LICs & LMICs Only**												
AFRO	5·49	1·70	0·48	14·92	4·96	1·52	3·22	1·26	0·43	9·52	3·91	1·39
AMRO	2·20	0·78	0·25	8·35	2·97	0·97	1·58	0·66	0·24	5·99	2·51	0·91
EMRO	1·87	0·62	0·19	5·20	1·78	0·56	1·24	0·50	0·17	3·51	1·45	0·52
EURO	2·69	0·90	0·27	10·23	3·42	1·04	1·79	0·72	0·25	6·81	2·75	0·96
SEARO	13·70	3·76	0·91	25·62	7·05	1·71	6·96	2·51	0·76	13·03	4·71	1·43
WPRO	4·15	1·21	0·32	7·82	2·30	0·61	2·32	0·87	0·28	4·40	1·66	0·53
GAVI	9·19	2·62	0·66	19·41	5·73	1·53	4·86	1·80	0·57	10·74	4·09	1·33
Global	7·37	2·13	0·55	15·68	4·68	1·27	3·97	1·48	0·47	8·83	3·38	1·11
**LICs, LMICs & UMICs**												
AFRO	5·22	1·63	0·47	13·99	4·67	1·44	3·11	1·23	0·42	8·99	3·70	1·32
AMRO	6·92	2·53	0·84	13·31	4·86	1·62	5·11	2·17	0·80	9·81	4·16	1·53
EMRO	1·73	0·58	0·18	4·57	1·57	0·50	1·16	0·47	0·17	3·10	1·28	0·46
EURO	2·97	1·02	0·32	6·78	2·32	0·73	2·06	0·85	0·30	4·67	1·91	0·68
SEARO	9·65	2·70	0·67	18·04	5·06	1·26	5·04	1·84	0·57	9·44	3·46	1·07
WPRO	5·30	1·61	0·44	9·87	3·01	0·83	3·16	1·21	0·39	5·90	2·26	0·74
GAVI	9·19	2·62	0·66	19·41	5·73	1·53	4·86	1·80	0·57	10·74	4·09	1·33
Global	6·01	1·82	0·50	12·11	3·73	1·04	3·50	1·35	0·44	7·19	2·79	0·93
Best case												
**LICs & LMICs Only**												
AFRO	12·97	4·02	1·13	35·26	11·73	3·59	7·62	2·99	1·00	22·51	9·25	3·28
AMRO	5·19	1·85	0·60	19·74	7·02	2·29	3·73	1·56	0·56	14·16	5·92	2·15
EMRO	4·42	1·47	0·45	12·29	4·20	1·33	2·92	1·18	0·41	8·30	3·42	1·23
EURO	6·36	2·12	0·65	24·17	8·07	2·46	4·23	1·71	0·60	16·09	6·50	2·26

SEARO	32·38	8·89	2·14	60·56	16·66	4·03	16·44	5·93	1·80	30·81	11·14	3·39
WPRO	9·81	2·87	0·75	18·48	5·44	1·43	5·48	2·05	0·65	10·40	3·91	1·25
GAVI	21·71	6·19	1·57	45·88	13·54	3·61	11·49	4·26	1·34	25·40	9·67	3·14
Global	17·42	5·02	1·29	37·06	11·07	2·99	9·38	3·51	1·11	20·87	8·00	2·63
**LICs, LMICs & UMICs**												
AFRO	12·34	3·86	1·10	33·07	11·04	3·40	7·34	2·90	0·98	21·24	8·74	3·11
AMRO	16·17	5·93	1·99	31·12	11·39	3·82	11·97	5·09	1·88	23·02	9·78	3·61
EMRO	4·09	1·38	0·43	10·79	3·71	1·18	2·74	1·11	0·39	7·34	3·03	1·09
EURO	7·02	2·42	0·76	16·02	5·49	1·72	4·88	2·00	0·71	11·04	4·52	1·60
SEARO	22·80	6·39	1·58	42·63	11·96	2·97	11·91	4·36	1·34	22·31	8·17	2·53
WPRO	6·20	1·88	0·51	11·58	3·51	0·96	3·67	1·40	0·45	6·86	2·62	0·85
GAVI	21·71	6·19	1·57	45·88	13·54	3·61	11·49	4·26	1·34	25·40	9·67	3·14
Global	11·98	3·62	0·99	24·45	7·53	2·11	6·93	2·66	0·87	14·49	5·64	1·89

Note: Results ≤1.10 are highlighted, representing ratios that are below parity or borderline. AFRO: African region, AMRO: Region of the Americas, EMRO: Eastern Mediterranean Region, GAVI: Global Alliance for Vaccines and Immunisation, LIC: low-income country, LMIC: lower-middle income country, OECD: Organisation for Economic Co-operation and Development, SEARO: Southeast Asian Region, UMIC: upper-middle income country, WPRO: Western Pacific Region

Table S11. Sensitivity analysis for including only MSD, showing benefit cost ratios based on productivity benefits of reducing linear growth faltering attributable to *Shigella*, including 10% discount scenario

	Average scenario						Conservative, early retirement (age 50)						Optimistic, z-score height premium					
	Income effect			Multiplier effect			Income effect			Multiplier effect			Income effect			Multiplier effect		
Discounting	3%	6%	10%	3%	6%	10%	3%	6%	10%	3%	6%	10%	3%	6%	10%	3%	6%	10%
**LICs & LMICs Only**																		
AFRO	2·40	0·74	0·21	6·47	2·14	0·65	1·40	0·55	0·18	4·11	1·68	0·59	8·38	2·60	0·73	22·80	7·57	2·31
AMRO	1·17	0·42	0·14	4·47	1·59	0·52	0·84	0·35	0·13	3·21	1·34	0·49	4·22	1·50	0·49	16·06	5·71	1·86
EMRO	1·04	0·35	0·10	2·86	0·98	0·31	0·68	0·28	0·10	1·93	0·79	0·28	3·63	1·21	0·37	10·09	3·45	1·09
EURO	1·54	0·51	0·16	5·86	1·96	0·60	1·03	0·41	0·14	3·90	1·58	0·55	5·55	1·85	0·56	21·11	7·05	2·15
SEARO	8·18	2·25	0·54	15·31	4·21	1·02	4·16	1·50	0·45	7·78	2·81	0·85	28·12	7·72	1·86	52·59	14·47	3·50
WPRO	2·63	0·77	0·20	4·96	1·46	0·38	1·47	0·55	0·17	2·79	1·05	0·33	9·06	2·65	0·69	17·06	5·02	1·32
GAVI	4·78	1·36	0·34	10·05	2·96	0·79	2·52	0·93	0·29	5·55	2·11	0·68	16·50	4·70	1·19	34·86	10·29	2·74

Global	3·97	1·14	0·29	8·39	2·50	0·67	2·13	0·80	0·25	4·71	1·80	0·59	13·70	3·95	1·02	29·13	8·70	2·35
**LICs, LMICs & UMICs**																		
AFRO	2·27	0·71	0·20	6·05	2·01	0·61	1·35	0·53	0·18	3·87	1·58	0·56	7·95	2·48	0·71	21·30	7·10	2·18
AMRO	3·39	1·24	0·41	6·51	2·38	0·79	2·51	1·06	0·39	4·81	2·04	0·75	12·01	4·34	1·44	23·08	8·35	2·76
EMRO	0·98	0·33	0·10	2·57	0·88	0·28	0·66	0·27	0·09	1·75	0·72	0·26	3·43	1·15	0·36	9·04	3·11	0·98
EURO	1·77	0·61	0·19	4·00	1·37	0·43	1·23	0·50	0·18	2·76	1·13	0·40	6·12	2·11	0·66	13·97	4·78	1·50
SEARO	5·90	1·65	0·41	11·03	3·09	0·77	3·08	1·13	0·35	5·77	2·11	0·65	20·27	5·67	1·41	37·90	10·63	2·64
WPRO	2·25	0·68	0·19	4·19	1·28	0·35	1·34	0·51	0·17	2·49	0·95	0·31	22·49	6·87	1·89	41·90	12·81	3·53
GAVI	4·78	1·36	0·34	10·05	2·96	0·79	2·52	0·93	0·29	5·55	2·11	0·68	16·50	4·70	1·19	34·86	10·29	2·74
Global	3·07	0·93	0·25	6·21	1·91	0·53	1·78	0·68	0·22	3·68	1·43	0·48	15·33	4·66	1·28	30·32	9·34	2·61

Note: Results ≤1.10 are highlighted, representing ratios that are below parity or borderline. AFRO: African region, AMRO: Region of the Americas, EMRO: Eastern Mediterranean Region, GAVI: Global Alliance for Vaccines and Immunisation, LIC: low-income country, LMIC: lower-middle income country, OECD: Organisation for Economic Co-operation and Development, SEARO: Southeast Asian Region, UMIC: upper-middle income country, WPRO: Western Pacific Region

Table S12. Sensitivity analysis for the effect of low vaccine efficacy (10%), on benefit-cost ratios including productivity benefits for preventing growth faltering due to *Shigella*

	Average scenario						Conservative, early retirement (age 50)						Optimistic, z-score height premium					
	Income effect			Multiplier effect			Income effect			Multiplier effect			Income effect			Multiplier effect		
Discounting	3%	6%	10%	3%	6%	10%	3%	6%	10%	3%	6%	10%	3%	6%	10%	3%	6%	10%
**LICs & LMICs Only**																		
AFRO	1·83	0·57	0·16	4·94	1·64	0·50	1·07	0·42	0·14	3·14	1·29	0·46	6·41	1·99	0·56	17·42	5·79	1·77
AMRO	0·72	0·26	0·08	2·73	0·97	0·32	0·52	0·22	0·08	1·96	0·82	0·30	2·58	0·92	0·30	9·81	3·49	1·14
EMRO	0·62	0·21	0·06	1·72	0·59	0·19	0·41	0·17	0·06	1·16	0·48	0·17	2·18	0·73	0·22	6·07	2·08	0·66
EURO	0·88	0·29	0·09	3·33	1·11	0·34	0·58	0·24	0·08	2·22	0·90	0·31	3·15	1·05	0·32	11·99	4·01	1·22
SEARO	4·68	1·28	0·31	8·75	2·41	0·58	2·37	0·86	0·26	4·45	1·61	0·49	16·07	4·41	1·06	30·06	8·27	2·00
WPRO	1·42	0·41	0·11	2·67	0·78	0·21	0·79	0·30	0·09	1·50	0·56	0·18	4·87	1·42	0·37	9·17	2·70	0·71
GAVI	3·12	0·89	0·22	6·56	1·93	0·51	1·65	0·61	0·19	3·62	1·37	0·45	10·76	3·07	0·78	22·74	6·71	1·79
Global	2·50	0·72	0·18	5·29	1·58	0·42	1·34	0·50	0·16	2·97	1·14	0·37	8·64	2·49	0·64	18·37	5·48	1·48
**LICs, LMICs & UMICs**																		
AFRO	1·74	0·54	0·15	4·64	1·54	0·47	1·03	0·41	0·14	2·97	1·22	0·43	6·09	1·91	0·54	16·34	5·45	1·68

AMRO	2·35	0·86	0·29	4·51	1·65	0·55	1·73	0·74	0·27	3·33	1·41	0·52	8·31	3·01	1·00	15·97	5·79	1·92
EMRO	0·58	0·20	0·06	1·52	0·52	0·16	0·39	0·16	0·06	1·03	0·43	0·15	2·02	0·68	0·21	5·34	1·83	0·58
EURO	1·00	0·35	0·11	2·28	0·78	0·24	0·70	0·29	0·10	1·57	0·64	0·23	3·49	1·20	0·38	7·95	2·72	0·85
SEARO	3·29	0·92	0·23	6·16	1·73	0·43	1·72	0·63	0·19	3·22	1·18	0·37	11·32	3·17	0·79	21·16	5·94	1·48
WPRO	1·23	0·37	0·10	2·29	0·70	0·19	0·73	0·28	0·09	1·36	0·52	0·17	12·27	3·75	1·03	22·87	6·99	1·93
GAVI	3·12	0·89	0·22	6·56	1·93	0·51	1·65	0·61	0·19	3·62	1·37	0·45	10·76	3·07	0·78	22·74	6·71	1·79
Global	1·84	0·56	0·15	3·72	1·14	0·32	1·07	0·41	0·13	2·20	0·86	0·29	9·19	2·80	0·77	18·18	5·60	1·57

Note: Results ≤1.10 are highlighted, representing ratios that are below parity or borderline. AFRO: African region, AMRO: Region of the Americas, EMRO: Eastern Mediterranean Region, GAVI: Global Alliance for Vaccines and Immunisation, LIC: low-income country, LMIC: lower-middle income country, OECD: Organisation for Economic Co-operation and Development, SEARO: Southeast Asian Region, UMIC: upper-middle income country, WPRO: Western Pacific Region

Table S13. Sensitivity analysis for the effect of high and low cost estimates, on benefit-cost ratios including productivity benefits for preventing growth faltering due to *Shigella*

	Average scenario						Conservative, early retirement (age 50)						Optimistic, z-score height premium					
	Income effect			Multiplier effect			Income effect			Multiplier effect			Income effect			Multiplier effect		
Discounting	3%	6%	10%	3%	6%	10%	3%	6%	10%	3%	6%	10%	3%	6%	10%	3%	6%	10%
Worst case (high costs)																		
**LICs & LMICs Only**																		
AFRO	4·76	1·47	0·41	12·84	4·25	1·29	2·78	1·08	0·36	8·16	3·34	1·18	16·65	5·15	1·45	45·26	15·03	4·59
AMRO	2·11	0·75	0·24	8·03	2·86	0·93	1·52	0·63	0·23	5·76	2·41	0·87	7·59	2·70	0·88	28·87	10·27	3·34
EMRO	1·91	0·64	0·19	5·28	1·80	0·57	1·26	0·51	0·18	3·56	1·47	0·53	6·70	2·24	0·68	18·64	6·37	2·01
EURO	2·81	0·94	0·29	10·69	3·57	1·09	1·87	0·76	0·26	7·11	2·87	1·00	10·12	3·38	1·03	38·48	12·85	3·91
SEARO	16·15	4·43	1·07	30·19	8·30	2·01	8·20	2·96	0·89	15·36	5·55	1·69	55·47	15·23	3·67	103·75	28·54	6·91
WPRO	4·53	1·33	0·35	8·54	2·51	0·66	2·53	0·95	0·30	4·80	1·81	0·58	15·58	4·56	1·19	29·36	8·64	2·27
GAVI	9·53	2·71	0·69	20·05	5·90	1·57	5·03	1·86	0·58	11·06	4·21	1·36	32·89	9·38	2·38	69·52	20·53	5·47
Global	7·60	2·19	0·56	16·09	4·79	1·29	4·08	1·52	0·48	9·03	3·46	1·13	26·25	7·57	1·95	55·83	16·67	4·51
**LICs, LMICs & UMICs**																		
AFRO	4·48	1·39	0·39	11·92	3·96	1·21	2·65	1·04	0·35	7·62	3·12	1·11	15·65	4·89	1·39	41·97	13·98	4·29
AMRO	6·00	2·19	0·73	11·52	4·20	1·40	4·43	1·88	0·69	8·51	3·61	1·33	21·23	7·68	2·54	40·81	14·76	4·88
EMRO	1·77	0·60	0·18	4·65	1·59	0·50	1·19	0·48	0·17	3·16	1·30	0·47	6·19	2·09	0·64	16·34	5·61	1·78

EURO	2·96	1·02	0·32	6·70	2·29	0·72	2·05	0·84	0·30	4·62	1·89	0·67	10·25	3·53	1·11	23·39	8·01	2·51
SEARO	10·83	3·03	0·75	20·23	5·67	1·41	5·66	2·07	0·64	10·59	3·88	1·20	37·19	10·41	2·58	69·53	19·50	4·85
WPRO	3·72	1·13	0·31	6·95	2·11	0·58	2·21	0·84	0·27	4·13	1·58	0·51	37·27	11·39	3·14	69·45	21·24	5·85
GAVI	9·53	2·71	0·69	20·05	5·90	1·57	5·03	1·86	0·58	11·06	4·21	1·36	32·89	9·38	2·38	69·52	20·53	5·47
Global	5·46	1·65	0·45	11·06	3·40	0·95	3·17	1·22	0·40	6·55	2·54	0·85	27·30	8·30	2·28	54·00	16·63	4·65
Best case (low costs)																		
**LICs & LMICs Only**																		
AFRO	54·36	17·03	4·85	146·71	49·29	15·30	31·76	12·59	4·29	93·19	38·74	13·96	190·14	59·83	17·15	516·98	174·44	54·39
AMRO	6·54	2·33	0·76	24·88	8·87	2·89	4·70	1·97	0·71	17·85	7·48	2·72	23·53	8·39	2·74	89·45	31·88	10·40
EMRO	5·82	1·94	0·59	16·06	5·50	1·74	3·84	1·56	0·54	10·84	4·47	1·61	20·37	6·82	2·08	56·65	19·42	6·15
EURO	6·42	2·15	0·65	24·41	8·16	2·49	4·27	1·73	0·60	16·25	6·57	2·29	23·11	7·72	2·36	87·88	29·37	8·96
SEARO	31·77	8·73	2·10	59·42	16·35	3·96	16·13	5·82	1·76	30·22	10·93	3·32	109·17	29·99	7·23	204·18	56·20	13·61
WPRO	10·36	3·03	0·79	19·50	5·74	1·51	5·78	2·16	0·69	10·97	4·12	1·32	35·59	10·41	2·72	67·06	19·74	5·20
GAVI	31·02	8·80	2·22	65·27	19·16	5·07	16·38	6·05	1·89	36·01	13·65	4·41	107·10	30·44	7·68	226·34	66·61	17·67
Global	24·03	6·90	1·77	50·88	15·12	4·06	12·91	4·81	1·52	28·56	10·90	3·56	83·01	23·88	6·13	176·57	52·60	14·19
**LICs, LMICs & UMICs**																		
AFRO	56·11	17·77	5·14	149·32	50·43	15·74	33·25	13·28	4·58	95·47	39·80	14·40	196·14	62·38	18·14	525·78	178·26	55·89
AMRO	30·62	11·23	3·77	58·82	21·56	7·24	22·64	9·64	3·57	43·45	18·50	6·85	108·41	39·40	13·11	208·38	75·68	25·17
EMRO	5·25	1·77	0·55	13·76	4·73	1·50	3·51	1·43	0·50	9·34	3·86	1·39	18·33	6·20	1·91	48·39	16·66	5·30
EURO	8·43	2·91	0·92	19·11	6·56	2·06	5·86	2·41	0·86	13·18	5·40	1·91	29·26	10·10	3·19	66·73	22·89	7·18
SEARO	23·61	6·61	1·64	44·12	12·38	3·08	12·33	4·51	1·39	23·09	8·46	2·62	81·10	22·72	5·63	151·62	42·54	10·58
WPRO	9·74	2·96	0·81	18·18	5·53	1·51	5·79	2·21	0·72	10·82	4·13	1·35	97·50	29·83	8·22	181·70	55·61	15·32
GAVI	31·02	8·80	2·22	65·27	19·16	5·07	16·38	6·05	1·89	36·01	13·65	4·41	107·10	30·44	7·68	226·34	66·61	17·67
Global	16·82	5·08	1·39	34·03	10·46	2·92	9·75	3·74	1·23	20·15	7·83	2·61	84·03	25·54	7·02	166·23	51·15	14·29

Note: Results $\leq$1.10 are highlighted, representing ratios that are below parity or borderline. AFRO: African region, AMRO: Region of the Americas, EMRO: Eastern Mediterranean Region, GAVI: Global Alliance for Vaccines and Immunisation, LIC: low-income country, LMIC: lower-middle income country, OECD: Organisation for Economic Co-operation and Development, SEARO: Southeast Asian Region, UMIC: upper-middle income country, WPRO: Western Pacific Region

Table S14. Sensitivity analysis for the effect of high and low z score shift estimates, on benefit-cost ratios including productivity benefits for preventing growth faltering due to *Shigella*

	Average scenario						Conservative, early retirement (age 50)						Optimistic, z-score height premium					
	Income effect			Multiplier effect			Income effect			Multiplier effect			Income effect			Multiplier effect		
Discounting	3%	6%	10%	3%	6%	10%	3%	6%	10%	3%	6%	10%	3%	6%	10%	3%	6%	10%
Worst case (low Z score shift)																		
**LICs & LMICs Only**																		
AFRO	2·16	0·67	0·19	5·83	1·93	0·59	1·26	0·49	0·17	3·70	1·52	0·54	7·56	2·34	0·66	20·55	6·83	2·09
AMRO	1·04	0·37	0·12	3·95	1·41	0·46	0·75	0·31	0·11	2·84	1·18	0·43	3·74	1·33	0·43	14·21	5·05	1·64
EMRO	0·75	0·25	0·08	2·06	0·70	0·22	0·49	0·20	0·07	1·39	0·57	0·21	2·61	0·87	0·27	7·26	2·49	0·79
EURO	1·16	0·39	0·12	4·42	1·48	0·45	0·77	0·31	0·11	2·94	1·19	0·41	4·18	1·40	0·43	15·90	5·32	1·62
SEARO	5·84	1·60	0·39	10·91	3·00	0·73	2·96	1·07	0·32	5·55	2·01	0·61	20·05	5·51	1·33	37·50	10·32	2·50
WPRO	1·93	0·57	0·15	3·64	1·07	0·28	1·08	0·40	0·13	2·05	0·77	0·25	6·65	1·95	0·51	12·53	3·69	0·97
GAVI	3·84	1·09	0·28	8·05	2·36	0·63	2·02	0·75	0·23	4·43	1·68	0·54	13·25	3·77	0·96	27·89	8·22	2·18
Global	3·10	0·89	0·23	6·54	1·94	0·52	1·67	0·62	0·20	3·66	1·40	0·46	10·71	3·09	0·79	22·69	6·76	1·83
**LICs, LMICs & UMICs**																		
AFRO	2·06	0·64	0·18	5·47	1·82	0·56	1·22	0·48	0·16	3·50	1·44	0·51	7·19	2·25	0·64	19·27	6·43	1·98
AMRO	3·40	1·24	0·42	6·53	2·38	0·80	2·51	1·07	0·39	4·82	2·05	0·75	12·04	4·36	1·44	23·13	8·37	2·77
EMRO	0·69	0·23	0·07	1·81	0·62	0·20	0·46	0·19	0·07	1·23	0·51	0·18	2·42	0·82	0·25	6·38	2·20	0·70
EURO	1·33	0·46	0·15	3·02	1·04	0·32	0·93	0·38	0·14	2·08	0·85	0·30	4·62	1·60	0·50	10·54	3·61	1·13
SEARO	4·11	1·15	0·29	7·68	2·16	0·54	2·15	0·79	0·24	4·02	1·47	0·46	14·12	3·96	0·98	26·40	7·41	1·84
WPRO	1·68	0·51	0·14	3·13	0·95	0·26	1·00	0·38	0·12	1·86	0·71	0·23	16·76	5·13	1·41	31·24	9·56	2·64
GAVI	3·84	1·09	0·28	8·05	2·36	0·63	2·02	0·75	0·23	4·43	1·68	0·54	13·25	3·77	0·96	27·89	8·22	2·18
Global	2·36	0·72	0·20	4·76	1·47	0·41	1·38	0·53	0·17	2·83	1·10	0·37	12·05	3·67	1·01	23·71	7·32	2·05
Best case (high Z score shift)																		
**LICs & LMICs Only**																		
AFRO	20·31	6·28	1·76	54·83	18·18	5·55	11·87	4·64	1·56	34·85	14·30	5·07	71·04	22·06	6·22	193·21	64·34	19·75
AMRO	7·12	2·54	0·83	27·08	9·64	3·14	5·11	2·14	0·78	19·44	8·13	2·95	25·60	9·11	2·97	97·35	34·66	11·29
EMRO	6·98	2·33	0·71	19·28	6·58	2·07	4·61	1·86	0·65	13·00	5·35	1·92	24·46	8·16	2·48	68·01	23·24	7·33
EURO	9·40	3·14	0·95	35·74	11·93	3·63	6·25	2·52	0·88	23·78	9·60	3·34	33·84	11·29	3·44	128·67	42·94	13·07

SEARO	51·12	14·03	3·38	95·59	26·29	6·36	25·94	9·35	2·83	48·61	17·56	5·34	175·62	48·21	11·61	328·48	90·35	21·86
WPRO	14·97	4·37	1·14	28·19	8·29	2·18	8·35	3·12	0·99	15·85	5·96	1·90	51·44	15·04	3·92	96·92	28·52	7·50
GAVI	34·18	9·73	2·46	72·01	21·21	5·63	18·05	6·69	2·10	39·75	15·11	4·90	118·05	33·65	8·53	249·71	73·74	19·65
Global	27·37	7·88	2·02	57·99	17·28	4·66	14·71	5·49	1·74	32·56	12·47	4·08	94·54	27·26	7·01	201·25	60·11	16·27
**LICs, LMICs & UMICs**																		
AFRO	19·33	6·04	1·72	51·46	17·14	5·26	11·46	4·51	1·53	32·93	13·53	4·81	67·58	21·19	6·06	181·21	60·58	18·67
AMRO	23·28	8·52	2·86	44·73	16·36	5·48	17·22	7·32	2·70	33·05	14·04	5·18	82·45	29·90	9·93	158·46	57·43	19·06
EMRO	6·48	2·18	0·67	16·99	5·83	1·84	4·34	1·76	0·62	11·53	4·76	1·71	22·63	7·63	2·35	59·74	20·52	6·50
EURO	10·78	3·71	1·17	24·42	8·36	2·62	7·48	3·07	1·09	16·83	6·88	2·43	37·39	12·88	4·05	85·28	29·19	9·13
SEARO	36·00	10·08	2·49	67·29	18·87	4·69	18·80	6·87	2·12	35·20	12·88	3·98	123·68	34·62	8·57	231·22	64·84	16·11
WPRO	12·96	3·94	1·07	24·18	7·35	2·01	7·70	2·94	0·95	14·38	5·49	1·79	129·68	39·63	10·90	241·67	73·88	20·33
GAVI	34·18	9·73	2·46	72·01	21·21	5·63	18·05	6·69	2·10	39·75	15·11	4·90	118·05	33·65	8·53	249·71	73·74	19·65
Global	19·82	5·98	1·63	40·19	12·34	3·45	11·46	4·39	1·44	23·75	9·22	3·07	98·47	29·91	8·21	195·11	60·04	16·77

Note: Results ≤1.10 are highlighted, representing ratios that are below parity or borderline. AFRO: African region, AMRO: Region of the Americas, EMRO: Eastern Mediterranean Region, GAVI: Global Alliance for Vaccines and Immunisation, LIC: low-income country, LMIC: lower-middle income country, OECD: Organisation for Economic Co-operation and Development, SEARO: Southeast Asian Region, UMIC: upper-middle income country, WPRO: Western Pacific Region

Comparison with nutrition intervention results from similar model

The present model was adapted from a model published by Alderman et al (2017),[16] which examined the benefits and costs of a package of evidence-based nutrition. We compare some of the results from this previous analysis with the results of the present analysis, given the similarities between the models. Alderman et al (2017) presented BCRs for the South Asia region, one of which looks directly at mapping height to earnings using a height premium of 0.55%, finding a BCR of 23 at 3% discounting and 7 at 6% discounting. This can be compared with the income effect results from our worst-case height premium scenario (also 0.55%) for SEARO (including UMICs) of 9.7 at 3% discounting and 2.7 at 6% discounting.

Table S15. Authors' calculations of the total growth faltering and stunting burden attributable to Shigella by region, and the proportion preventable by a vaccine

Region	Total Linear Growth Faltering episodes	Shigella Linear Growth Faltering episodes	Shigella Linear Growth Faltering episodes (% of total)	Total Stunting episodes	Shigella Stunting episodes	Shigella Stunting episodes (% of total)	Shigella Stunting episodes averted	Shigella Stunting episodes averted (% of total)
AFRO	2,015,860,821	84,765,104	4·2%	1,096,823,339	69,297,159	6·3%	25,889,453	2·4%
AMRO	204,103,950	9,888,909	4·8%	79,193,342	5,338,218	6·7%	2,161,297	2·7%
EMRO	659,609,233	14,850,191	2·3%	366,366,640	11,881,921	3·2%	4,865,319	1·3%
EURO	54,619,626	1,389,544	2·5%	19,690,429	650,217	3·3%	287,743	1·5%
SEARO	1,552,980,185	22,959,174	1·5%	829,420,163	18,569,364	2·2%	7,874,727	0·9%
WPRO	385,707,616	6,738,268	1·7%	158,209,219	3,610,766	2·3%	1,538,884	1·0%
Global Average (%)			2·8%			4·0%		1·6%

Bhutta et al (2013) estimate that the package of nutrition interventions has the potential to reduce approximately 20% of the global burden of stunting.[19] To compare this with the potential impact of a *Shigella* vaccine, we estimated the global burden of stunting attributable to *Shigella* (by region) along with the proportion that could plausibly be averted by a vaccine, see Table A14. We estimated the global average proportion of the total Shigella-attributable stunting burden that could be prevented by a vaccine for *Shigella* would be 1.6%.

Table S16. Comparing benefit-cost results of a *Shigella* vaccine with an evidence-based package of nutrition interventions as reported by Alderman et al (2017)

	Nutrition interventions	Shigella Vaccine	Vaccine as % of Nutrition interventions	Nutrition interventions relative to vaccine program
Intervention timeframe (years)	15	20		
NPV costs (3% disc)	18,564,534,677	4,080,995,072	22·0%	4·5
NPV benefits (3% disc)	433,577,704,552	39,372,524,831	9·1%	11·0
Benefit-cost ratio (BCR)	23·4	9·6		
% Global stunting reduced	20·0%†	1·6%‡	8·0%	12·5

†Estimated by Bhutta et al (2013); ‡Authors' calculations (see Table A14)

The comparative results are examined in Table A15. Compared to the 15-year nutrition intervention roll-out, the 20-year vaccine program produced 9% of the benefits at 22% of the costs, and was estimated to reduce 8% of the global stunting burden that the nutrition interventions would. While the nutrition

interventions are more costly, they produce more benefits and avert more stunting compared to a vaccine for Shigella. However, the Shigella vaccine would produce additional benefits not quantified here, directly related to reduction in diarrheal burden and associated morbidity and mortality. For the global share of stunting burden that the Shigella vaccine was projected to address, on top of these other benefits, the costs are relatively modest.

Companion cost-effectiveness analysis

The companion cost-effectiveness analysis was completed alongside this analysis.[20] Vaccination costs and z-score shift estimates were used along with height premium estimates for all scenarios.

Shigella vaccine prices were estimated using WHO MI4A (Market Information for Access to Vaccines) Vaccine Market data.[21] First, the average rotavirus and pneumococcal conjugate vaccine prices were estimated for the following groups: LMIs and UMIs by WHO region; PAHO (AMRO) countries; and Gavi-ineligible countries. For all Gavi-eligible countries, vaccine price was estimated at $2, with a range of $1–3 upper and lower values to define the Gamma distribution used for the simulations of Gavi-eligible vaccine price (see Table 2 in companion cost-effectiveness analysis).[20] Separate prices were calculated for Gavi-ineligible AMRO countries regardless of income class, to account for vaccines procured through the Pan American Health Organization (PAHO) Revolving Fund. Ratios of average prices in LMI, UMI, and PAHO Gavi-ineligible countries to Gavi-eligible countries were applied to estimate the relative magnitude of Gavi-ineligible dose price estimates.

Vaccination costs (V) were cumulated over the first 20 years (t) post vaccine introduction starting in 2025 for each country (c) based on vaccine administration costs, vaccine price, and quantity (birth cohort times coverage rate with 10% vaccine wastage, Eq. 1, Table). Birth cohorts were calculated as the fraction of the under 5 population eligible for vaccination at 6 months. Averted costs (A) were calculated based on population, vaccine coverage, efficacy, and access to care and medical costs in each country (c). Net costs (N) for each country were aggregated by region (r), WB income class (w) and Gavi eligibility (g) as:

$$\text{Eq. 1:} \qquad N_{r,w,g} = \sum_{t=20}^{r,w,g} (V_c - A_c)$$

Vaccine benefits (*B)* for each region (*r*) were calculated based on the sum of population, coverage (*C*), efficacy (*E*), and DALY burden (*D*) in each country (*c*) cumulated over the first 20 years (*t*) after introduction (Eq. 2). DALYs were calculated using standard methods[22,23] and were not age-weighted but were discounted 3% annually from 2025−2044, with an assumed life expectancy of 84 years. The number of children fully vaccinated each year was estimated by multiplying the annual birth cohort by the assumed vaccine coverage. Benefits were projected over the first five years of life for children vaccinated in each annual birth cohort.

$$\text{Eq. 2:} \qquad B_{r,w,g} = \sum_{t=20}^{r,w,g} (C_c \cdot E_c \cdot D_c)$$

The primary cost-effectiveness measure was aggregated Incremental Cost-Effectiveness Ratios ($\text{ICER}_{r,w,g}$). Country-level incremental costs associated with each vaccine introduction were added over 20 years and aggregated for each region then divided by aggregated country-level health benefit estimates (Eq. 3).

$$\text{Eq. 3:} \qquad \text{ICER}_{r,w,g} = \frac{\sum_{t=20}^{r,w,g} N_{r,w,g}}{\sum_{t=20}^{r,w,g} B_{r,w,g}}$$

Long-term productivity gains from vaccination (*P*) were combined with the shorter-term costs and benefits from the companion cost-effectiveness analysis (Eq. 4). ICERS were estimated by subtracting monetized long-term productivity gains from net vaccination costs (*N*) from the companion cost effectiveness. The denominator (*B*) remained the same. Separate ICERs were calculated for average (*a*) and early (*e*) retirement scenarios.

Eq. 4: $$ICER_{r,w,g}^{e,a} = \frac{\sum_{t=20}^{r,w,g} N_{r,w,g} - \sum_{a,e}^{r,w,g} P_{r,w,g}}{\sum_{t=20}^{r,w,g} B_{r,w,g}}$$

Table S17. Population average z-score shifts assuming that the vaccine was 40% (20-60% range) effective against *Shigella* less severe diarrhoea (LSD) and *Shigella*-attributable stunting from LSD episodes. Estimates in parenthesis represent 95% uncertainty estimates from the results of 1000 model simulations.

Country	2025	2026	2027	2028	2029	2030	2031	2032	2033	2034	2035	2036	2037	2038	2039	20[illegible]
Afghanistan	0.010 (0.002; 0.025)	0.010 (0.002; 0.025)	0.010 (0.002; 0.025)	0.010 (0.002; 0.025)	0.010 (0.002; 0.025)	0.010 (0.002; 0.026)	0.010 (0.002; 0.026)	0.010 (0.002; 0.026)	0.010 (0.002; 0.026)	0.011 (0.002; 0.026)	0.011 (0.002; 0.026)	0.011 (0.002; 0.027)	0.011 (0.002; 0.027)	0.011 (0.002; 0.027)	0.011 (0.002; 0.027)	[illegible]
Algeria	0.022 (0.005; 0.058)	0.022 (0.005; 0.058)	0.022 (0.004; 0.058)	0.022 (0.004; 0.057)	0.022 (0.004; 0.057)	0.022 (0.004; 0.057)	0.022 (0.004; 0.056)	0.022 (0.004; 0.056)	0.021 (0.004; 0.056)	0.021 (0.004; 0.056)	0.021 (0.004; 0.055)	0.021 (0.004; 0.055)	0.021 (0.004; 0.055)	0.021 (0.004; 0.054)	0.021 (0.004; 0.054)	[illegible]
Angola	0.015 (0.003; 0.039)	0.015 (0.003; 0.039)	0.015 (0.003; 0.039)	0.015 (0.003; 0.039)	0.015 (0.003; 0.040)	0.015 (0.003; 0.040)	0.015 (0.003; 0.040)	0.015 (0.003; 0.040)	0.015 (0.003; 0.040)	0.016 (0.003; 0.040)	0.016 (0.003; 0.041)	0.016 (0.003; 0.041)	0.016 (0.003; 0.041)	0.016 (0.003; 0.041)	0.016 (0.003; 0.041)	[illegible]
Argentina	0.015 (0.004; 0.034)	0.015 (0.004; 0.034)	0.015 (0.004; 0.034)	0.015 (0.004; 0.034)	0.015 (0.004; 0.034)	0.015 (0.004; 0.034)	0.015 (0.004; 0.034)	0.015 (0.004; 0.034)	0.015 (0.004; 0.034)	0.015 (0.004; 0.034)	0.015 (0.004; 0.033)	0.015 (0.004; 0.033)	0.015 (0.004; 0.033)	0.015 (0.004; 0.033)	0.015 (0.004; 0.033)	[illegible]
Armenia	0.007 (0.002; 0.018)	0.007 (0.002; 0.018)	0.007 (0.002; 0.018)	0.007 (0.002; 0.017)	0.007 (0.002; 0.017)	0.007 (0.002; 0.017)	0.007 (0.002; 0.017)	0.007 (0.002; 0.017)	0.007 (0.002; 0.017)	0.007 (0.002; 0.017)	0.007 (0.002; 0.017)	0.007 (0.002; 0.017)	0.007 (0.002; 0.017)	0.007 (0.002; 0.017)	0.007 (0.002; 0.017)	[illegible]
Azerbaijan	0.008 (0.002; 0.018)	0.008 (0.002; 0.018)	0.008 (0.002; 0.018)	0.008 (0.002; 0.018)	0.007 (0.002; 0.018)	0.007 (0.002; 0.018)	0.007 (0.002; 0.018)	0.007 (0.002; 0.018)	0.007 (0.002; 0.018)	0.007 (0.002; 0.018)	0.007 (0.002; 0.017)	0.007 (0.002; 0.017)	0.007 (0.002; 0.017)	0.007 (0.002; 0.017)	0.007 (0.002; 0.017)	[illegible]
Bangladesh	0.008 (0.002; 0.019)	0.008 (0.002; 0.019)	0.008 (0.002; 0.019)	0.008 (0.002; 0.019)	0.008 (0.002; 0.019)	0.008 (0.002; 0.019)	0.008 (0.002; 0.019)	0.008 (0.002; 0.019)	0.008 (0.002; 0.019)	0.008 (0.002; 0.019)	0.008 (0.002; 0.019)	0.008 (0.002; 0.019)	0.008 (0.002; 0.019)	0.008 (0.002; 0.019)	0.008 (0.002; 0.019)	[illegible]
Belarus	0.008 (0.002; 0.019)	0.008 (0.002; 0.019)	0.008 (0.002; 0.019)	0.008 (0.002; 0.019)	0.008 (0.002; 0.019)	0.008 (0.002; 0.018)	0.008 (0.002; 0.018)	0.008 (0.002; 0.018)	0.008 (0.002; 0.018)	0.008 (0.002; 0.018)	0.008 (0.002; 0.018)	0.008 (0.002; 0.018)	0.008 (0.002; 0.018)	0.008 (0.002; 0.018)	0.008 (0.002; 0.018)	[illegible]
Benin	0.020 (0.004; 0.052)	0.020 (0.004; 0.052)	0.020 (0.004; 0.052)	0.020 (0.004; 0.052)	0.020 (0.004; 0.053)	0.020 (0.004; 0.053)	0.020 (0.004; 0.053)	0.021 (0.004; 0.053)	0.021 (0.004; 0.054)	0.021 (0.004; 0.054)	0.021 (0.004; 0.054)	0.021 (0.004; 0.054)	0.021 (0.004; 0.054)	0.021 (0.004; 0.054)	0.021 (0.004; 0.053)	[illegible]
Bhutan	0.008 (0.002; 0.019)	0.008 (0.002; 0.019)	0.008 (0.002; 0.019)	0.008 (0.002; 0.019)	0.008 (0.002; 0.019)	0.008 (0.002; 0.019)	0.008 (0.002; 0.019)	0.008 (0.002; 0.019)	0.008 (0.002; 0.019)	0.008 (0.002; 0.019)	0.008 (0.002; 0.019)	0.008 (0.002; 0.019)	0.008 (0.002; 0.019)	0.008 (0.002; 0.019)	0.008 (0.002; 0.019)	[illegible]

Bolivia	0.014 (0.004; 0.031)	0.014 (0.004; 0.031)	0.014 (0.004; 0.031)	0.014 (0.004; 0.031)	0.014 (0.004; 0.031)	0.014 (0.004; 0.032)	0.014 (0.004; 0.032)	0.014 (0.004; 0.032)	0.014 (0.004; 0.032)	0.014 (0.004; 0.032)	0.014 (0.004; 0.033)	0.015 (0.004; 0.033)	0.015 (0.004; 0.033)	0.015 (0.004; 0.033)	0.015 (0.004; 0.033)	[illegible]
Botswana	0.023 (0.005; 0.061)	0.023 (0.005; 0.061)	0.023 (0.005; 0.060)	0.023 (0.005; 0.060)	0.023 (0.005; 0.060)	0.023 (0.005; 0.059)	0.023 (0.005; 0.059)	0.023 (0.005; 0.059)	0.022 (0.005; 0.058)	0.022 (0.005; 0.058)	0.022 (0.005; 0.058)	0.022 (0.005; 0.057)	0.022 (0.005; 0.057)	0.022 (0.005; 0.057)	0.022 (0.004; 0.056)	[illegible]
Brazil	0.013 (0.004; 0.029)	0.013 (0.004; 0.029)	0.013 (0.004; 0.029)	0.013 (0.004; 0.029)	0.013 (0.004; 0.029)	0.013 (0.004; 0.030)	0.013 (0.004; 0.030)	0.013 (0.004; 0.030)	0.013 (0.004; 0.030)	0.013 (0.004; 0.030)	0.014 (0.004; 0.030)	0.014 (0.004; 0.031)	0.014 (0.004; 0.031)	0.014 (0.004; 0.031)	0.014 (0.004; 0.031)	[illegible]
Bulgaria	0.008 (0.002; 0.018)	0.008 (0.002; 0.018)	0.007 (0.002; 0.018)	0.007 (0.002; 0.018)	0.007 (0.002; 0.018)	0.007 (0.002; 0.018)	0.007 (0.002; 0.017)	0.007 (0.002; 0.017)	0.007 (0.002; 0.017)	0.007 (0.002; 0.017)	0.007 (0.002; 0.017)	0.007 (0.002; 0.017)	0.007 (0.002; 0.017)	0.007 (0.002; 0.017)	0.007 (0.002; 0.017)	[illegible]
Burkina Faso	0.022 (0.005; 0.058)	0.022 (0.005; 0.058)	0.022 (0.004; 0.058)	0.022 (0.004; 0.057)	0.022 (0.004; 0.057)	0.022 (0.004; 0.057)	0.022 (0.004; 0.056)	0.022 (0.004; 0.056)	0.021 (0.004; 0.056)	0.021 (0.004; 0.056)	0.021 (0.004; 0.055)	0.021 (0.004; 0.055)	0.021 (0.004; 0.055)	0.021 (0.004; 0.054)	0.021 (0.004; 0.054)	[illegible]
Burundi	0.023 (0.005; 0.060)	0.023 (0.005; 0.059)	0.022 (0.005; 0.059)	0.022 (0.005; 0.059)	0.022 (0.005; 0.058)	0.022 (0.005; 0.058)	0.022 (0.004; 0.058)	0.022 (0.004; 0.057)	0.022 (0.004; 0.057)	0.022 (0.004; 0.057)	0.022 (0.004; 0.056)	0.022 (0.004; 0.056)	0.022 (0.004; 0.056)	0.022 (0.004; 0.055)	0.021 (0.004; 0.055)	[illegible]
Cabo Verde	0.023 (0.005; 0.062)	0.023 (0.005; 0.061)	0.023 (0.005; 0.061)	0.023 (0.005; 0.061)	0.023 (0.005; 0.060)	0.023 (0.005; 0.060)	0.023 (0.005; 0.060)	0.023 (0.005; 0.059)	0.023 (0.005; 0.059)	0.023 (0.005; 0.059)	0.023 (0.005; 0.058)	0.022 (0.005; 0.058)	0.022 (0.005; 0.058)	0.022 (0.005; 0.057)	0.022 (0.005; 0.057)	[illegible]
Cambodia	0.006 (0.002; 0.014)	0.006 (0.002; 0.014)	0.006 (0.002; 0.014)	0.006 (0.002; 0.014)	0.006 (0.001; 0.014)	0.006 (0.001; 0.014)	0.006 (0.001; 0.014)	0.006 (0.001; 0.014)	0.006 (0.001; 0.014)	0.006 (0.001; 0.014)	0.006 (0.001; 0.014)	0.006 (0.001; 0.014)	0.006 (0.001; 0.014)	0.006 (0.001; 0.014)	0.006 (0.001; 0.014)	[illegible]
Cameroon	0.017 (0.004; 0.046)	0.017 (0.004; 0.046)	0.018 (0.004; 0.046)	0.018 (0.004; 0.046)	0.018 (0.004; 0.046)	0.018 (0.004; 0.047)	0.018 (0.004; 0.047)	0.018 (0.004; 0.047)	0.018 (0.004; 0.047)	0.018 (0.004; 0.047)	0.018 (0.004; 0.048)	0.019 (0.004; 0.048)	0.019 (0.004; 0.048)	0.019 (0.004; 0.048)	0.019 (0.004; 0.048)	[illegible]
Central African Republic	0.011 (0.002; 0.029)	0.011 (0.002; 0.029)	0.011 (0.002; 0.029)	0.011 (0.002; 0.029)	0.011 (0.002; 0.029)	0.011 (0.002; 0.029)	0.011 (0.002; 0.029)	0.011 (0.002; 0.030)	0.011 (0.002; 0.030)	0.011 (0.002; 0.030)	0.012 (0.002; 0.030)	0.012 (0.002; 0.030)	0.012 (0.002; 0.030)	0.012 (0.002; 0.030)	0.012 (0.002; 0.030)	[illegible]
Chad	0.013 (0.003; 0.034)	0.013 (0.003; 0.034)	0.013 (0.003; 0.034)	0.013 (0.003; 0.035)	0.013 (0.003; 0.035)	0.013 (0.003; 0.035)	0.013 (0.003; 0.035)	0.013 (0.003; 0.035)	0.014 (0.003; 0.035)	0.014 (0.003; 0.035)	0.014 (0.003; 0.036)	0.014 (0.003; 0.036)	0.014 (0.003; 0.036)	0.014 (0.003; 0.036)	0.014 (0.003; 0.036)	[illegible]
China	0.006 (0.002; 0.015)	0.006 (0.002; 0.015)	0.006 (0.002; 0.015)	0.006 (0.002; 0.015)	0.006 (0.002; 0.015)	0.006 (0.002; 0.015)	0.006 (0.002; 0.015)	0.006 (0.002; 0.015)	0.006 (0.002; 0.015)	0.006 (0.002; 0.015)	0.006 (0.002; 0.015)	0.006 (0.002; 0.015)	0.006 (0.002; 0.015)	0.006 (0.002; 0.015)	0.006 (0.002; 0.015)	[illegible]

Colombia	0.016 (0.004; 0.036)	0.016 (0.004; 0.036)	0.016 (0.004; 0.036)	0.016 (0.004; 0.036)	0.016 (0.004; 0.036)	0.016 (0.004; 0.036)	0.016 (0.004; 0.035)	0.016 (0.004; 0.035)	0.016 (0.004; 0.035)	0.016 (0.004; 0.035)	0.015 (0.004; 0.035)	0.015 (0.004; 0.035)	0.015 (0.004; 0.035)	0.015 (0.004; 0.034)	0.015 (0.004; 0.034)	0.0[illegible] (0.0[illegible] 0.03[illegible]
Comoros	0.022 (0.005; 0.058)	0.022 (0.005; 0.058)	0.022 (0.004; 0.058)	0.022 (0.004; 0.057)	0.022 (0.004; 0.057)	0.022 (0.004; 0.057)	0.022 (0.004; 0.056)	0.022 (0.004; 0.056)	0.021 (0.004; 0.056)	0.021 (0.004; 0.056)	0.021 (0.004; 0.055)	0.021 (0.004; 0.055)	0.021 (0.004; 0.055)	0.021 (0.004; 0.054)	0.021 (0.004; 0.054)	0.0[illegible] (0.0[illegible] 0.05[illegible]
Congo, Dem. Rep.	0.015 (0.003; 0.039)	0.015 (0.003; 0.039)	0.015 (0.003; 0.039)	0.015 (0.003; 0.039)	0.015 (0.003; 0.040)	0.015 (0.003; 0.040)	0.015 (0.003; 0.040)	0.015 (0.003; 0.040)	0.015 (0.003; 0.040)	0.016 (0.003; 0.040)	0.016 (0.003; 0.041)	0.016 (0.003; 0.041)	0.016 (0.003; 0.041)	0.016 (0.003; 0.041)	0.016 (0.003; 0.041)	0.0[illegible] (0.0[illegible] 0.04[illegible]
Congo, Rep.	0.020 (0.004; 0.054)	0.021 (0.004; 0.054)	0.021 (0.004; 0.054)	0.021 (0.004; 0.055)	0.021 (0.004; 0.055)	0.021 (0.004; 0.055)	0.021 (0.004; 0.055)	0.021 (0.004; 0.055)	0.021 (0.004; 0.055)	0.021 (0.004; 0.055)	0.021 (0.004; 0.055)	0.021 (0.004; 0.054)	0.021 (0.004; 0.054)	0.021 (0.004; 0.054)	0.021 (0.004; 0.053)	0.0[illegible] (0.0[illegible] 0.05[illegible]
Costa Rica	0.016 (0.005; 0.036)	0.016 (0.005; 0.036)	0.016 (0.005; 0.036)	0.016 (0.004; 0.036)	0.016 (0.004; 0.036)	0.016 (0.004; 0.036)	0.016 (0.004; 0.036)	0.016 (0.004; 0.036)	0.016 (0.004; 0.036)	0.016 (0.004; 0.035)	0.016 (0.004; 0.035)	0.016 (0.004; 0.035)	0.016 (0.004; 0.035)	0.015 (0.004; 0.035)	0.015 (0.004; 0.035)	0.0[illegible] (0.0[illegible] 0.03[illegible]
Cote d'Ivoire	0.022 (0.004; 0.057)	0.022 (0.004; 0.057)	0.022 (0.004; 0.057)	0.022 (0.004; 0.057)	0.022 (0.004; 0.057)	0.021 (0.004; 0.056)	0.021 (0.004; 0.056)	0.021 (0.004; 0.056)	0.021 (0.004; 0.055)	0.021 (0.004; 0.055)	0.021 (0.004; 0.055)	0.021 (0.004; 0.054)	0.021 (0.004; 0.054)	0.021 (0.004; 0.054)	0.021 (0.004; 0.053)	0.0[illegible] (0.0[illegible] 0.05[illegible]
Djibouti	0.012 (0.003; 0.029)	0.012 (0.003; 0.029)	0.012 (0.003; 0.029)	0.012 (0.003; 0.029)	0.012 (0.003; 0.029)	0.012 (0.003; 0.029)	0.011 (0.003; 0.029)	0.011 (0.003; 0.029)	0.011 (0.003; 0.028)	0.011 (0.003; 0.028)	0.011 (0.003; 0.028)	0.011 (0.003; 0.028)	0.011 (0.002; 0.028)	0.011 (0.002; 0.028)	0.011 (0.002; 0.028)	0.0[illegible] (0.0[illegible] 0.02[illegible]
Dominican Republic	0.015 (0.004; 0.035)	0.015 (0.004; 0.034)	0.015 (0.004; 0.034)	0.015 (0.004; 0.034)	0.015 (0.004; 0.034)	0.015 (0.004; 0.034)	0.015 (0.004; 0.034)	0.015 (0.004; 0.034)	0.015 (0.004; 0.034)	0.015 (0.004; 0.034)	0.015 (0.004; 0.033)	0.015 (0.004; 0.033)	0.015 (0.004; 0.033)	0.015 (0.004; 0.033)	0.015 (0.004; 0.033)	0.0[illegible] (0.0[illegible] 0.03[illegible]
Ecuador	0.015 (0.004; 0.035)	0.015 (0.004; 0.034)	0.015 (0.004; 0.034)	0.015 (0.004; 0.034)	0.015 (0.004; 0.034)	0.015 (0.004; 0.034)	0.015 (0.004; 0.034)	0.015 (0.004; 0.034)	0.015 (0.004; 0.034)	0.015 (0.004; 0.034)	0.015 (0.004; 0.033)	0.015 (0.004; 0.033)	0.015 (0.004; 0.033)	0.015 (0.004; 0.033)	0.015 (0.004; 0.033)	0.0[illegible] (0.0[illegible] 0.03[illegible]
Egypt, Arab Rep.	0.012 (0.003; 0.031)	0.012 (0.003; 0.031)	0.012 (0.003; 0.031)	0.012 (0.003; 0.030)	0.012 (0.003; 0.030)	0.012 (0.003; 0.030)	0.012 (0.003; 0.030)	0.012 (0.003; 0.030)	0.012 (0.003; 0.030)	0.012 (0.003; 0.030)	0.012 (0.003; 0.030)	0.012 (0.003; 0.030)	0.012 (0.003; 0.029)	0.012 (0.003; 0.029)	0.012 (0.003; 0.029)	0.0[illegible] (0.0[illegible] 0.02[illegible]
El Salvador	0.015 (0.004; 0.033)	0.015 (0.004; 0.033)	0.015 (0.004; 0.034)	0.015 (0.004; 0.034)	0.015 (0.004; 0.034)	0.015 (0.004; 0.034)	0.015 (0.004; 0.034)	0.015 (0.004; 0.034)	0.015 (0.004; 0.034)	0.015 (0.004; 0.034)	0.015 (0.004; 0.033)	0.015 (0.004; 0.033)	0.015 (0.004; 0.033)	0.015 (0.004; 0.033)	0.015 (0.004; 0.033)	0.0[illegible] (0.0[illegible] 0.03[illegible]
Eritrea	0.023 (0.005; 0.061)	0.023 (0.005; 0.061)	0.023 (0.005; 0.060)	0.023 (0.005; 0.060)	0.023 (0.005; 0.060)	0.023 (0.005; 0.059)	0.023 (0.005; 0.059)	0.023 (0.005; 0.059)	0.022 (0.005; 0.058)	0.022 (0.005; 0.058)	0.022 (0.005; 0.058)	0.022 (0.005; 0.057)	0.022 (0.005; 0.057)	0.022 (0.005; 0.057)	0.022 (0.004; 0.056)	0.0[illegible] (0.0[illegible] 0.05[illegible]

Eswatini	0.022 (0.004; 0.058)	0.022 (0.004; 0.057)	0.022 (0.004; 0.057)	0.022 (0.004; 0.057)	0.022 (0.004; 0.057)	0.021 (0.004; 0.056)	0.021 (0.004; 0.056)	0.021 (0.004; 0.056)	0.021 (0.004; 0.055)	0.021 (0.004; 0.055)	0.021 (0.004; 0.055)	0.021 (0.004; 0.054)	0.021 (0.004; 0.054)	0.021 (0.004; 0.054)	0.021 (0.004; 0.053)	0.0 (0.0 0.05
Ethiopia	0.018 (0.004; 0.046)	0.018 (0.004; 0.047)	0.018 (0.004; 0.047)	0.018 (0.004; 0.047)	0.018 (0.004; 0.047)	0.018 (0.004; 0.047)	0.018 (0.004; 0.048)	0.018 (0.004; 0.048)	0.018 (0.004; 0.048)	0.019 (0.004; 0.048)	0.019 (0.004; 0.048)	0.019 (0.004; 0.049)	0.019 (0.004; 0.049)	0.019 (0.004; 0.049)	0.019 (0.004; 0.049)	0.0 (0.0 0.04
Fiji	0.006 (0.002; 0.015)	0.006 (0.002; 0.015)	0.006 (0.002; 0.015)	0.006 (0.002; 0.015)	0.006 (0.002; 0.015)	0.006 (0.002; 0.015)	0.006 (0.002; 0.015)	0.006 (0.002; 0.015)	0.006 (0.002; 0.015)	0.006 (0.002; 0.015)	0.006 (0.002; 0.015)	0.006 (0.002; 0.015)	0.006 (0.002; 0.015)	0.006 (0.002; 0.015)	0.006 (0.002; 0.015)	0.0 (0.0 0.01
Gabon	0.018 (0.004; 0.048)	0.018 (0.004; 0.048)	0.018 (0.004; 0.048)	0.018 (0.004; 0.048)	0.019 (0.004; 0.049)	0.019 (0.004; 0.049)	0.019 (0.004; 0.049)	0.019 (0.004; 0.049)	0.019 (0.004; 0.049)	0.019 (0.004; 0.050)	0.019 (0.004; 0.050)	0.019 (0.004; 0.050)	0.019 (0.004; 0.050)	0.020 (0.004; 0.050)	0.020 (0.004; 0.051)	0.0 (0.0 0.05
Gambia, The	0.022 (0.004; 0.058)	0.022 (0.004; 0.057)	0.022 (0.004; 0.057)	0.022 (0.004; 0.057)	0.022 (0.004; 0.057)	0.021 (0.004; 0.056)	0.021 (0.004; 0.056)	0.021 (0.004; 0.056)	0.021 (0.004; 0.055)	0.021 (0.004; 0.055)	0.021 (0.004; 0.055)	0.021 (0.004; 0.054)	0.021 (0.004; 0.054)	0.021 (0.004; 0.054)	0.021 (0.004; 0.053)	0.0 (0.0 0.05
Georgia	0.008 (0.002; 0.018)	0.008 (0.002; 0.018)	0.008 (0.002; 0.018)	0.008 (0.002; 0.018)	0.007 (0.002; 0.018)	0.007 (0.002; 0.018)	0.007 (0.002; 0.018)	0.007 (0.002; 0.018)	0.007 (0.002; 0.018)	0.007 (0.002; 0.018)	0.007 (0.002; 0.017)	0.007 (0.002; 0.017)	0.007 (0.002; 0.017)	0.007 (0.002; 0.017)	0.007 (0.002; 0.017)	0.0 (0.0 0.01
Ghana	0.024 (0.005; 0.062)	0.024 (0.005; 0.062)	0.023 (0.005; 0.062)	0.023 (0.005; 0.061)	0.023 (0.005; 0.061)	0.023 (0.005; 0.061)	0.023 (0.005; 0.060)	0.023 (0.005; 0.060)	0.023 (0.005; 0.060)	0.023 (0.005; 0.059)	0.023 (0.005; 0.059)	0.023 (0.005; 0.059)	0.023 (0.005; 0.058)	0.022 (0.005; 0.058)	0.022 (0.005; 0.058)	0.0 (0.0 0.05
Guatemala	0.015 (0.004; 0.035)	0.015 (0.004; 0.034)	0.015 (0.004; 0.034)	0.015 (0.004; 0.034)	0.015 (0.004; 0.034)	0.015 (0.004; 0.034)	0.015 (0.004; 0.034)	0.015 (0.004; 0.034)	0.015 (0.004; 0.034)	0.015 (0.004; 0.034)	0.015 (0.004; 0.033)	0.015 (0.004; 0.033)	0.015 (0.004; 0.033)	0.015 (0.004; 0.033)	0.015 (0.004; 0.033)	0.0 (0.0 0.03
Guinea	0.012 (0.002; 0.032)	0.012 (0.003; 0.032)	0.012 (0.003; 0.032)	0.012 (0.003; 0.032)	0.012 (0.003; 0.033)	0.013 (0.003; 0.033)	0.013 (0.003; 0.033)	0.013 (0.003; 0.033)	0.013 (0.003; 0.033)	0.013 (0.003; 0.033)	0.013 (0.003; 0.033)	0.013 (0.003; 0.034)	0.013 (0.003; 0.034)	0.013 (0.003; 0.034)	0.013 (0.003; 0.034)	0.0 (0.0 0.03
Guinea-Bissau	0.020 (0.004; 0.053)	0.020 (0.004; 0.053)	0.020 (0.004; 0.054)	0.021 (0.004; 0.054)	0.021 (0.004; 0.054)	0.021 (0.004; 0.054)	0.021 (0.004; 0.055)	0.021 (0.004; 0.055)	0.021 (0.004; 0.055)	0.021 (0.004; 0.055)	0.021 (0.004; 0.055)	0.021 (0.004; 0.054)	0.021 (0.004; 0.054)	0.021 (0.004; 0.054)	0.021 (0.004; 0.053)	0.0 (0.0 0.05
Haiti	0.009 (0.003; 0.021)	0.009 (0.003; 0.021)	0.009 (0.003; 0.021)	0.009 (0.003; 0.021)	0.009 (0.003; 0.021)	0.010 (0.003; 0.022)	0.010 (0.003; 0.022)	0.010 (0.003; 0.022)	0.010 (0.003; 0.022)	0.010 (0.003; 0.022)	0.010 (0.003; 0.022)	0.010 (0.003; 0.022)	0.010 (0.003; 0.022)	0.010 (0.003; 0.023)	0.010 (0.003; 0.023)	0.0 (0.0 0.02
Honduras	0.015 (0.004; 0.035)	0.015 (0.004; 0.034)	0.015 (0.004; 0.034)	0.015 (0.004; 0.034)	0.015 (0.004; 0.034)	0.015 (0.004; 0.034)	0.015 (0.004; 0.034)	0.015 (0.004; 0.034)	0.015 (0.004; 0.034)	0.015 (0.004; 0.034)	0.015 (0.004; 0.033)	0.015 (0.004; 0.033)	0.015 (0.004; 0.033)	0.015 (0.004; 0.033)	0.015 (0.004; 0.033)	0.0 (0.0 0.03

India	0.007 (0.002; 0.018)	0.007 (0.002; 0.018)	0.007 (0.002; 0.018)	0.007 (0.002; 0.018)	0.007 (0.002; 0.018)	0.007 (0.002; 0.018)	0.007 (0.002; 0.018)	0.007 (0.002; 0.018)	0.007 (0.002; 0.018)	0.007 (0.002; 0.018)	0.007 (0.002; 0.018)	0.007 (0.002; 0.018)	0.007 (0.002; 0.017)	0.007 (0.002; 0.017)	0.007 (0.002; 0.017)	0.0 (0.0 0.0
Indonesia	0.007 (0.002; 0.018)	0.007 (0.002; 0.018)	0.007 (0.002; 0.018)	0.007 (0.002; 0.018)	0.007 (0.002; 0.018)	0.007 (0.002; 0.018)	0.007 (0.002; 0.018)	0.007 (0.002; 0.018)	0.007 (0.002; 0.017)	0.007 (0.002; 0.017)	0.007 (0.002; 0.017)	0.007 (0.002; 0.017)	0.007 (0.002; 0.017)	0.007 (0.002; 0.017)	0.007 (0.002; 0.017)	0.0 (0.0 0.0
Iraq	0.012 (0.003; 0.029)	0.012 (0.003; 0.029)	0.012 (0.003; 0.029)	0.012 (0.003; 0.029)	0.012 (0.003; 0.029)	0.012 (0.003; 0.029)	0.011 (0.003; 0.029)	0.011 (0.003; 0.029)	0.011 (0.003; 0.028)	0.011 (0.003; 0.028)	0.011 (0.003; 0.028)	0.011 (0.003; 0.028)	0.011 (0.002; 0.028)	0.011 (0.002; 0.028)	0.011 (0.002; 0.028)	0.0 (0.0 0.0
Jamaica	0.016 (0.005; 0.037)	0.016 (0.005; 0.037)	0.016 (0.005; 0.037)	0.016 (0.005; 0.037)	0.016 (0.005; 0.036)	0.016 (0.004; 0.036)	0.016 (0.004; 0.036)	0.016 (0.004; 0.036)	0.016 (0.004; 0.036)	0.016 (0.004; 0.036)	0.016 (0.004; 0.036)	0.016 (0.004; 0.036)	0.016 (0.004; 0.035)	0.016 (0.004; 0.035)	0.016 (0.004; 0.035)	0.0 (0.0 0.0
Jordan	0.012 (0.003; 0.029)	0.012 (0.003; 0.029)	0.012 (0.003; 0.029)	0.012 (0.003; 0.029)	0.012 (0.003; 0.029)	0.012 (0.003; 0.029)	0.011 (0.003; 0.029)	0.011 (0.003; 0.029)	0.011 (0.003; 0.028)	0.011 (0.003; 0.028)	0.011 (0.003; 0.028)	0.011 (0.003; 0.028)	0.011 (0.002; 0.028)	0.011 (0.002; 0.028)	0.011 (0.002; 0.028)	0.0 (0.0 0.0
Kazakhstan	0.008 (0.002; 0.019)	0.008 (0.002; 0.019)	0.008 (0.002; 0.018)	0.008 (0.002; 0.018)	0.008 (0.002; 0.018)	0.008 (0.002; 0.018)	0.008 (0.002; 0.018)	0.008 (0.002; 0.018)	0.008 (0.002; 0.018)	0.008 (0.002; 0.018)	0.008 (0.002; 0.018)	0.008 (0.002; 0.018)	0.008 (0.002; 0.018)	0.007 (0.002; 0.018)	0.007 (0.002; 0.018)	0.0 (0.0 0.0
Kenya	0.022 (0.005; 0.058)	0.022 (0.005; 0.058)	0.022 (0.004; 0.058)	0.022 (0.004; 0.057)	0.022 (0.004; 0.057)	0.022 (0.004; 0.057)	0.022 (0.004; 0.056)	0.022 (0.004; 0.056)	0.021 (0.004; 0.056)	0.021 (0.004; 0.056)	0.021 (0.004; 0.055)	0.021 (0.004; 0.055)	0.021 (0.004; 0.055)	0.021 (0.004; 0.054)	0.021 (0.004; 0.054)	0.0 (0.0 0.0
Kiribati	0.006 (0.002; 0.015)	0.006 (0.002; 0.015)	0.006 (0.002; 0.015)	0.006 (0.002; 0.015)	0.006 (0.002; 0.015)	0.006 (0.002; 0.015)	0.006 (0.002; 0.015)	0.006 (0.002; 0.015)	0.006 (0.002; 0.015)	0.006 (0.002; 0.015)	0.006 (0.002; 0.014)	0.006 (0.002; 0.014)	0.006 (0.002; 0.014)	0.006 (0.002; 0.014)	0.006 (0.001; 0.014)	0.0 (0.0 0.0
Kyrgyz Republic	0.008 (0.002; 0.018)	0.008 (0.002; 0.018)	0.008 (0.002; 0.018)	0.008 (0.002; 0.018)	0.008 (0.002; 0.018)	0.008 (0.002; 0.018)	0.008 (0.002; 0.018)	0.007 (0.002; 0.018)	0.007 (0.002; 0.018)	0.007 (0.002; 0.018)	0.007 (0.002; 0.018)	0.007 (0.002; 0.018)	0.007 (0.002; 0.018)	0.007 (0.002; 0.017)	0.007 (0.002; 0.017)	0.0 (0.0 0.0
Lao PDR	0.005 (0.001; 0.013)	0.005 (0.001; 0.013)	0.005 (0.001; 0.013)	0.006 (0.001; 0.013)	0.006 (0.001; 0.013)	0.006 (0.001; 0.014)	0.006 (0.001; 0.014)	0.006 (0.001; 0.014)	0.006 (0.001; 0.014)	0.006 (0.001; 0.013)	0.006 (0.001; 0.013)	0.006 (0.001; 0.013)	0.005 (0.001; 0.013)	0.005 (0.001; 0.013)	0.005 (0.001; 0.013)	0.0 (0.0 0.0
Lesotho	0.022 (0.004; 0.058)	0.022 (0.004; 0.057)	0.022 (0.004; 0.057)	0.022 (0.004; 0.057)	0.022 (0.004; 0.057)	0.021 (0.004; 0.056)	0.021 (0.004; 0.056)	0.021 (0.004; 0.056)	0.021 (0.004; 0.055)	0.021 (0.004; 0.055)	0.021 (0.004; 0.055)	0.021 (0.004; 0.054)	0.021 (0.004; 0.054)	0.021 (0.004; 0.054)	0.021 (0.004; 0.053)	0.0 (0.0 0.0
Liberia	0.018 (0.004; 0.048)	0.018 (0.004; 0.048)	0.018 (0.004; 0.048)	0.018 (0.004; 0.048)	0.019 (0.004; 0.049)	0.019 (0.004; 0.049)	0.019 (0.004; 0.049)	0.019 (0.004; 0.049)	0.019 (0.004; 0.049)	0.019 (0.004; 0.050)	0.019 (0.004; 0.050)	0.019 (0.004; 0.050)	0.019 (0.004; 0.050)	0.020 (0.004; 0.050)	0.020 (0.004; 0.051)	0.0 (0.0 0.0

Madagascar	0.018 (0.004; 0.048)	0.018 (0.004; 0.048)	0.018 (0.004; 0.048)	0.018 (0.004; 0.048)	0.019 (0.004; 0.049)	0.019 (0.004; 0.049)	0.019 (0.004; 0.049)	0.019 (0.004; 0.049)	0.019 (0.004; 0.049)	0.019 (0.004; 0.050)	0.019 (0.004; 0.050)	0.019 (0.004; 0.050)	0.019 (0.004; 0.050)	0.020 (0.004; 0.050)	0.020 (0.004; 0.051)	[illegible]
Malawi	0.023 (0.005; 0.061)	0.023 (0.005; 0.061)	0.023 (0.005; 0.060)	0.023 (0.005; 0.060)	0.023 (0.005; 0.060)	0.023 (0.005; 0.059)	0.023 (0.005; 0.059)	0.023 (0.005; 0.059)	0.022 (0.005; 0.058)	0.022 (0.005; 0.058)	0.022 (0.005; 0.058)	0.022 (0.005; 0.057)	0.022 (0.005; 0.057)	0.022 (0.005; 0.057)	0.022 (0.004; 0.056)	[illegible]
Malaysia	0.006 (0.002; 0.015)	0.006 (0.002; 0.015)	0.006 (0.002; 0.015)	0.006 (0.002; 0.015)	0.006 (0.002; 0.015)	0.006 (0.002; 0.015)	0.006 (0.002; 0.015)	0.006 (0.002; 0.015)	0.006 (0.002; 0.015)	0.006 (0.002; 0.015)	0.006 (0.002; 0.015)	0.006 (0.002; 0.015)	0.006 (0.002; 0.015)	0.006 (0.002; 0.014)	0.006 (0.002; 0.014)	[illegible]
Mali	0.020 (0.004; 0.053)	0.020 (0.004; 0.053)	0.020 (0.004; 0.053)	0.020 (0.004; 0.053)	0.020 (0.004; 0.053)	0.021 (0.004; 0.054)	0.021 (0.004; 0.054)	0.021 (0.004; 0.054)	0.021 (0.004; 0.054)	0.021 (0.004; 0.055)	0.021 (0.004; 0.055)	0.021 (0.004; 0.054)	0.021 (0.004; 0.054)	0.021 (0.004; 0.054)	0.021 (0.004; 0.053)	[illegible]
Mauritania	0.021 (0.004; 0.055)	0.021 (0.004; 0.055)	0.021 (0.004; 0.056)	0.021 (0.004; 0.056)	0.021 (0.004; 0.056)	0.021 (0.004; 0.056)	0.021 (0.004; 0.056)	0.021 (0.004; 0.056)	0.021 (0.004; 0.055)	0.021 (0.004; 0.055)	0.021 (0.004; 0.055)	0.021 (0.004; 0.054)	0.021 (0.004; 0.054)	0.021 (0.004; 0.054)	0.021 (0.004; 0.053)	[illegible]
Mexico	0.015 (0.004; 0.033)	0.015 (0.004; 0.034)	0.015 (0.004; 0.034)	0.015 (0.004; 0.034)	0.015 (0.004; 0.034)	0.015 (0.004; 0.034)	0.015 (0.004; 0.034)	0.015 (0.004; 0.034)	0.015 (0.004; 0.034)	0.015 (0.004; 0.034)	0.015 (0.004; 0.033)	0.015 (0.004; 0.033)	0.015 (0.004; 0.033)	0.015 (0.004; 0.033)	0.015 (0.004; 0.033)	[illegible]
Mongolia	0.006 (0.002; 0.015)	0.006 (0.002; 0.015)	0.006 (0.002; 0.015)	0.006 (0.002; 0.015)	0.006 (0.002; 0.015)	0.006 (0.002; 0.015)	0.006 (0.002; 0.015)	0.006 (0.002; 0.015)	0.006 (0.002; 0.015)	0.006 (0.002; 0.015)	0.006 (0.002; 0.015)	0.006 (0.002; 0.015)	0.006 (0.002; 0.015)	0.006 (0.002; 0.014)	0.006 (0.002; 0.014)	[illegible]
Morocco	0.013 (0.003; 0.032)	0.013 (0.003; 0.032)	0.013 (0.003; 0.032)	0.013 (0.003; 0.032)	0.013 (0.003; 0.032)	0.013 (0.003; 0.032)	0.013 (0.003; 0.031)	0.013 (0.003; 0.031)	0.013 (0.003; 0.031)	0.012 (0.003; 0.031)	0.012 (0.003; 0.031)	0.012 (0.003; 0.031)	0.012 (0.003; 0.031)	0.012 (0.003; 0.031)	0.012 (0.003; 0.031)	[illegible]
Mozambique	0.022 (0.004; 0.058)	0.022 (0.004; 0.057)	0.022 (0.004; 0.057)	0.022 (0.004; 0.057)	0.022 (0.004; 0.057)	0.021 (0.004; 0.056)	0.021 (0.004; 0.056)	0.021 (0.004; 0.056)	0.021 (0.004; 0.055)	0.021 (0.004; 0.055)	0.021 (0.004; 0.055)	0.021 (0.004; 0.054)	0.021 (0.004; 0.054)	0.021 (0.004; 0.054)	0.021 (0.004; 0.053)	[illegible]
Myanmar	0.007 (0.002; 0.018)	0.007 (0.002; 0.018)	0.007 (0.002; 0.018)	0.007 (0.002; 0.018)	0.007 (0.002; 0.018)	0.007 (0.002; 0.018)	0.007 (0.002; 0.018)	0.007 (0.002; 0.018)	0.007 (0.002; 0.017)	0.007 (0.002; 0.017)	0.007 (0.002; 0.017)	0.007 (0.002; 0.017)	0.007 (0.002; 0.017)	0.007 (0.002; 0.017)	0.007 (0.002; 0.017)	[illegible]
Namibia	0.022 (0.004; 0.058)	0.022 (0.004; 0.057)	0.022 (0.004; 0.057)	0.022 (0.004; 0.057)	0.022 (0.004; 0.057)	0.021 (0.004; 0.056)	0.021 (0.004; 0.056)	0.021 (0.004; 0.056)	0.021 (0.004; 0.055)	0.021 (0.004; 0.055)	0.021 (0.004; 0.055)	0.021 (0.004; 0.054)	0.021 (0.004; 0.054)	0.021 (0.004; 0.054)	0.021 (0.004; 0.053)	[illegible]
Nepal	0.008 (0.002; 0.018)	0.008 (0.002; 0.018)	0.008 (0.002; 0.018)	0.008 (0.002; 0.018)	0.008 (0.002; 0.018)	0.008 (0.002; 0.018)	0.007 (0.002; 0.018)	0.007 (0.002; 0.018)	0.007 (0.002; 0.018)	0.007 (0.002; 0.018)	0.007 (0.002; 0.018)	0.007 (0.002; 0.018)	0.007 (0.002; 0.018)	0.007 (0.002; 0.018)	0.007 (0.002; 0.018)	[illegible]

Nicaragua	0.017 (0.005; 0.038)	0.017 (0.005; 0.038)	0.017 (0.005; 0.037)	0.017 (0.005; 0.037)	0.017 (0.005; 0.037)	0.016 (0.005; 0.037)	0.016 (0.005; 0.037)	0.016 (0.005; 0.037)	0.016 (0.005; 0.037)	0.016 (0.005; 0.037)	0.016 (0.005; 0.036)	0.016 (0.005; 0.036)	0.016 (0.004; 0.036)	0.016 (0.004; 0.036)	0.016 (0.004; 0.036)	0.0[illegible] (0.0[illegible] 0.03[illegible]
Niger	0.021 (0.004; 0.055)	0.021 (0.004; 0.055)	0.021 (0.004; 0.056)	0.021 (0.004; 0.056)	0.021 (0.004; 0.056)	0.021 (0.004; 0.056)	0.021 (0.004; 0.056)	0.021 (0.004; 0.056)	0.021 (0.004; 0.055)	0.021 (0.004; 0.055)	0.021 (0.004; 0.055)	0.021 (0.004; 0.054)	0.021 (0.004; 0.054)	0.021 (0.004; 0.054)	0.021 (0.004; 0.053)	0.0[illegible] (0.0[illegible] 0.05[illegible]
Nigeria	0.015 (0.003; 0.039)	0.015 (0.003; 0.039)	0.015 (0.003; 0.039)	0.015 (0.003; 0.039)	0.015 (0.003; 0.040)	0.015 (0.003; 0.040)	0.015 (0.003; 0.040)	0.015 (0.003; 0.040)	0.015 (0.003; 0.040)	0.016 (0.003; 0.040)	0.016 (0.003; 0.041)	0.016 (0.003; 0.041)	0.016 (0.003; 0.041)	0.016 (0.003; 0.041)	0.016 (0.003; 0.041)	0.0[illegible] (0.0[illegible] 0.04[illegible]
Pakistan	0.012 (0.003; 0.029)	0.012 (0.003; 0.029)	0.012 (0.003; 0.029)	0.012 (0.003; 0.029)	0.012 (0.003; 0.029)	0.012 (0.003; 0.029)	0.011 (0.003; 0.029)	0.011 (0.003; 0.029)	0.011 (0.003; 0.028)	0.011 (0.003; 0.028)	0.011 (0.003; 0.028)	0.011 (0.003; 0.028)	0.011 (0.002; 0.028)	0.011 (0.002; 0.028)	0.011 (0.002; 0.028)	0.0[illegible] (0.0[illegible] 0.02[illegible]
Papua New Guinea	0.002 (0.001; 0.006)	0.002 (0.001; 0.006)	0.002 (0.001; 0.006)	0.002 (0.001; 0.006)	0.002 (0.001; 0.006)	0.002 (0.001; 0.006)	0.002 (0.001; 0.006)	0.002 (0.001; 0.006)	0.002 (0.001; 0.006)	0.003 (0.001; 0.006)	0.003 (0.001; 0.006)	0.003 (0.001; 0.006)	0.003 (0.001; 0.006)	0.003 (0.001; 0.006)	0.003 (0.001; 0.006)	0.0[illegible] (0.0[illegible] 0.00[illegible]
Paraguay	0.015 (0.004; 0.035)	0.015 (0.004; 0.034)	0.015 (0.004; 0.034)	0.015 (0.004; 0.034)	0.015 (0.004; 0.034)	0.015 (0.004; 0.034)	0.015 (0.004; 0.034)	0.015 (0.004; 0.034)	0.015 (0.004; 0.034)	0.015 (0.004; 0.034)	0.015 (0.004; 0.033)	0.015 (0.004; 0.033)	0.015 (0.004; 0.033)	0.015 (0.004; 0.033)	0.015 (0.004; 0.033)	0.0[illegible] (0.0[illegible] 0.03[illegible]
Peru	0.015 (0.004; 0.035)	0.015 (0.004; 0.034)	0.015 (0.004; 0.034)	0.015 (0.004; 0.034)	0.015 (0.004; 0.034)	0.015 (0.004; 0.034)	0.015 (0.004; 0.034)	0.015 (0.004; 0.034)	0.015 (0.004; 0.034)	0.015 (0.004; 0.034)	0.015 (0.004; 0.033)	0.015 (0.004; 0.033)	0.015 (0.004; 0.033)	0.015 (0.004; 0.033)	0.015 (0.004; 0.033)	0.0[illegible] (0.0[illegible] 0.03[illegible]
Philippines	0.005 (0.001; 0.013)	0.005 (0.001; 0.013)	0.005 (0.001; 0.013)	0.005 (0.001; 0.013)	0.005 (0.001; 0.013)	0.005 (0.001; 0.013)	0.005 (0.001; 0.013)	0.005 (0.001; 0.013)	0.005 (0.001; 0.013)	0.006 (0.001; 0.013)	0.006 (0.001; 0.013)	0.006 (0.001; 0.013)	0.005 (0.001; 0.013)	0.005 (0.001; 0.013)	0.005 (0.001; 0.013)	0.0[illegible] (0.0[illegible] 0.01[illegible]
Rwanda	0.024 (0.005; 0.063)	0.024 (0.005; 0.063)	0.024 (0.005; 0.062)	0.024 (0.005; 0.062)	0.023 (0.005; 0.062)	0.023 (0.005; 0.061)	0.023 (0.005; 0.061)	0.023 (0.005; 0.060)	0.023 (0.005; 0.060)	0.023 (0.005; 0.060)	0.023 (0.005; 0.059)	0.023 (0.005; 0.059)	0.023 (0.005; 0.059)	0.023 (0.005; 0.058)	0.023 (0.005; 0.058)	0.0[illegible] (0.0[illegible] 0.05[illegible]
Senegal	0.023 (0.005; 0.061)	0.023 (0.005; 0.061)	0.023 (0.005; 0.060)	0.023 (0.005; 0.060)	0.023 (0.005; 0.060)	0.023 (0.005; 0.059)	0.023 (0.005; 0.059)	0.023 (0.005; 0.059)	0.022 (0.005; 0.058)	0.022 (0.005; 0.058)	0.022 (0.005; 0.058)	0.022 (0.005; 0.057)	0.022 (0.005; 0.057)	0.022 (0.005; 0.057)	0.022 (0.004; 0.056)	0.0[illegible] (0.0[illegible] 0.05[illegible]
Sierra Leone	0.023 (0.005; 0.061)	0.023 (0.005; 0.061)	0.023 (0.005; 0.060)	0.023 (0.005; 0.060)	0.023 (0.005; 0.060)	0.023 (0.005; 0.059)	0.023 (0.005; 0.059)	0.023 (0.005; 0.059)	0.022 (0.005; 0.058)	0.022 (0.005; 0.058)	0.022 (0.005; 0.058)	0.022 (0.005; 0.057)	0.022 (0.005; 0.057)	0.022 (0.005; 0.057)	0.022 (0.004; 0.056)	0.0[illegible] (0.0[illegible] 0.05[illegible]
Solomon Islands	0.006 (0.002; 0.015)	0.006 (0.002; 0.014)	0.006 (0.002; 0.014)	0.006 (0.002; 0.014)	0.006 (0.002; 0.014)	0.006 (0.002; 0.014)	0.006 (0.002; 0.014)	0.006 (0.002; 0.014)	0.006 (0.001; 0.014)	0.006 (0.001; 0.014)	0.006 (0.001; 0.014)	0.006 (0.001; 0.014)	0.006 (0.001; 0.014)	0.006 (0.001; 0.014)	0.006 (0.001; 0.014)	0.0[illegible] (0.0[illegible] 0.01[illegible]

Somalia	0.006 (0.001; 0.014)	0.006 (0.001; 0.015)	0.006 (0.001; 0.015)	0.006 (0.001; 0.015)	0.006 (0.001; 0.015)	0.006 (0.001; 0.015)	0.006 (0.001; 0.015)	0.006 (0.001; 0.015)	0.006 (0.001; 0.015)	0.006 (0.001; 0.015)	0.006 (0.001; 0.015)	0.006 (0.001; 0.016)	0.006 (0.001; 0.016)	0.006 (0.001; 0.016)	0.006 (0.001; 0.016)	[illegible]
South Africa	0.022 (0.004; 0.058)	0.022 (0.004; 0.057)	0.022 (0.004; 0.057)	0.022 (0.004; 0.057)	0.022 (0.004; 0.057)	0.021 (0.004; 0.056)	0.021 (0.004; 0.056)	0.021 (0.004; 0.056)	0.021 (0.004; 0.055)	0.021 (0.004; 0.055)	0.021 (0.004; 0.055)	0.021 (0.004; 0.054)	0.021 (0.004; 0.054)	0.021 (0.004; 0.054)	0.021 (0.004; 0.053)	[illegible]
Sri Lanka	0.008 (0.002; 0.020)	0.008 (0.002; 0.020)	0.008 (0.002; 0.020)	0.008 (0.002; 0.020)	0.008 (0.002; 0.019)	0.008 (0.002; 0.019)	0.008 (0.002; 0.019)	0.008 (0.002; 0.019)	0.008 (0.002; 0.019)	0.008 (0.002; 0.019)	0.008 (0.002; 0.019)	0.008 (0.002; 0.019)	0.008 (0.002; 0.019)	0.008 (0.002; 0.019)	0.008 (0.002; 0.019)	[illegible]
Sudan	0.012 (0.003; 0.030)	0.012 (0.003; 0.030)	0.012 (0.003; 0.030)	0.012 (0.003; 0.030)	0.012 (0.003; 0.030)	0.012 (0.003; 0.030)	0.012 (0.003; 0.030)	0.012 (0.003; 0.029)	0.012 (0.003; 0.029)	0.012 (0.003; 0.029)	0.012 (0.003; 0.029)	0.012 (0.003; 0.029)	0.012 (0.003; 0.029)	0.012 (0.003; 0.029)	0.012 (0.003; 0.029)	[illegible]
Suriname	0.014 (0.004; 0.031)	0.014 (0.004; 0.032)	0.014 (0.004; 0.032)	0.014 (0.004; 0.032)	0.014 (0.004; 0.032)	0.014 (0.004; 0.033)	0.015 (0.004; 0.033)	0.015 (0.004; 0.033)	0.015 (0.004; 0.033)	0.015 (0.004; 0.033)	0.015 (0.004; 0.033)	0.015 (0.004; 0.033)	0.015 (0.004; 0.033)	0.015 (0.004; 0.033)	0.015 (0.004; 0.033)	[illegible]
Tajikistan	0.008 (0.002; 0.019)	0.008 (0.002; 0.019)	0.008 (0.002; 0.018)	0.008 (0.002; 0.018)	0.008 (0.002; 0.018)	0.008 (0.002; 0.018)	0.008 (0.002; 0.018)	0.008 (0.002; 0.018)	0.008 (0.002; 0.018)	0.008 (0.002; 0.018)	0.008 (0.002; 0.018)	0.008 (0.002; 0.018)	0.008 (0.002; 0.018)	0.007 (0.002; 0.018)	0.007 (0.002; 0.018)	[illegible]
Tanzania	0.022 (0.004; 0.058)	0.022 (0.004; 0.057)	0.022 (0.004; 0.057)	0.022 (0.004; 0.057)	0.022 (0.004; 0.057)	0.021 (0.004; 0.056)	0.021 (0.004; 0.056)	0.021 (0.004; 0.056)	0.021 (0.004; 0.055)	0.021 (0.004; 0.055)	0.021 (0.004; 0.055)	0.021 (0.004; 0.054)	0.021 (0.004; 0.054)	0.021 (0.004; 0.054)	0.021 (0.004; 0.053)	[illegible]
Thailand	0.008 (0.002; 0.019)	0.008 (0.002; 0.019)	0.008 (0.002; 0.019)	0.008 (0.002; 0.019)	0.008 (0.002; 0.019)	0.008 (0.002; 0.019)	0.008 (0.002; 0.019)	0.008 (0.002; 0.019)	0.008 (0.002; 0.019)	0.008 (0.002; 0.019)	0.008 (0.002; 0.019)	0.008 (0.002; 0.019)	0.008 (0.002; 0.019)	0.008 (0.002; 0.019)	0.008 (0.002; 0.019)	[illegible]
Timor-Leste	0.007 (0.002; 0.018)	0.007 (0.002; 0.018)	0.007 (0.002; 0.018)	0.007 (0.002; 0.018)	0.007 (0.002; 0.018)	0.007 (0.002; 0.018)	0.007 (0.002; 0.018)	0.007 (0.002; 0.018)	0.007 (0.002; 0.017)	0.007 (0.002; 0.017)	0.007 (0.002; 0.017)	0.007 (0.002; 0.017)	0.007 (0.002; 0.017)	0.007 (0.002; 0.017)	0.007 (0.002; 0.017)	[illegible]
Togo	0.022 (0.004; 0.057)	0.022 (0.004; 0.057)	0.022 (0.004; 0.057)	0.022 (0.004; 0.057)	0.022 (0.004; 0.057)	0.021 (0.004; 0.056)	0.021 (0.004; 0.056)	0.021 (0.004; 0.056)	0.021 (0.004; 0.055)	0.021 (0.004; 0.055)	0.021 (0.004; 0.055)	0.021 (0.004; 0.054)	0.021 (0.004; 0.054)	0.021 (0.004; 0.054)	0.021 (0.004; 0.053)	[illegible]
Tunisia	0.012 (0.003; 0.030)	0.012 (0.003; 0.030)	0.012 (0.003; 0.030)	0.012 (0.003; 0.030)	0.012 (0.003; 0.029)	0.012 (0.003; 0.029)	0.012 (0.003; 0.029)	0.012 (0.003; 0.029)	0.012 (0.003; 0.029)	0.012 (0.003; 0.029)	0.012 (0.003; 0.029)	0.012 (0.003; 0.029)	0.011 (0.003; 0.029)	0.011 (0.003; 0.028)	0.011 (0.003; 0.028)	[illegible]
Turkey	0.008 (0.002; 0.019)	0.008 (0.002; 0.019)	0.008 (0.002; 0.019)	0.008 (0.002; 0.019)	0.008 (0.002; 0.019)	0.008 (0.002; 0.019)	0.008 (0.002; 0.019)	0.008 (0.002; 0.019)	0.008 (0.002; 0.018)	0.008 (0.002; 0.018)	0.008 (0.002; 0.018)	0.008 (0.002; 0.018)	0.008 (0.002; 0.018)	0.008 (0.002; 0.018)	0.008 (0.002; 0.018)	[illegible]

Uganda	0.023 (0.005; 0.060)	0.023 (0.005; 0.059)	0.022 (0.005; 0.059)	0.022 (0.005; 0.059)	0.022 (0.005; 0.058)	0.022 (0.005; 0.058)	0.022 (0.004; 0.058)	0.022 (0.004; 0.057)	0.022 (0.004; 0.057)	0.022 (0.004; 0.057)	0.022 (0.004; 0.056)	0.022 (0.004; 0.056)	0.022 (0.004; 0.056)	0.022 (0.004; 0.055)	0.021 (0.004; 0.055)	[illegible]
Ukraine	0.007 (0.002; 0.016)	0.007 (0.002; 0.016)	0.007 (0.002; 0.017)	0.007 (0.002; 0.017)	0.007 (0.002; 0.017)	0.007 (0.002; 0.017)	0.007 (0.002; 0.017)	0.007 (0.002; 0.017)	0.007 (0.002; 0.017)	0.007 (0.002; 0.017)	0.007 (0.002; 0.017)	0.007 (0.002; 0.017)	0.007 (0.002; 0.017)	0.007 (0.002; 0.017)	0.007 (0.002; 0.017)	[illegible]
Uzbekistan	0.008 (0.002; 0.018)	0.008 (0.002; 0.018)	0.008 (0.002; 0.018)	0.008 (0.002; 0.018)	0.008 (0.002; 0.018)	0.008 (0.002; 0.018)	0.008 (0.002; 0.018)	0.008 (0.002; 0.018)	0.008 (0.002; 0.018)	0.008 (0.002; 0.018)	0.007 (0.002; 0.018)	0.007 (0.002; 0.018)	0.007 (0.002; 0.018)	0.007 (0.002; 0.018)	0.007 (0.002; 0.018)	[illegible]
Vanuatu	0.006 (0.001; 0.014)	0.006 (0.001; 0.014)	0.006 (0.001; 0.014)	0.006 (0.001; 0.014)	0.006 (0.001; 0.014)	0.006 (0.001; 0.014)	0.006 (0.001; 0.014)	0.006 (0.001; 0.014)	0.006 (0.001; 0.014)	0.006 (0.001; 0.013)	0.006 (0.001; 0.013)	0.006 (0.001; 0.013)	0.005 (0.001; 0.013)	0.005 (0.001; 0.013)	0.005 (0.001; 0.013)	[illegible]
Vietnam	0.006 (0.001; 0.014)	0.006 (0.001; 0.014)	0.006 (0.001; 0.014)	0.006 (0.001; 0.014)	0.006 (0.001; 0.014)	0.006 (0.001; 0.014)	0.006 (0.001; 0.014)	0.006 (0.001; 0.014)	0.006 (0.001; 0.014)	0.006 (0.001; 0.013)	0.006 (0.001; 0.013)	0.006 (0.001; 0.013)	0.005 (0.001; 0.013)	0.005 (0.001; 0.013)	0.005 (0.001; 0.013)	[illegible]
Yemen, Rep.	0.010 (0.002; 0.025)	0.010 (0.002; 0.025)	0.010 (0.002; 0.025)	0.010 (0.002; 0.026)	0.010 (0.002; 0.026)	0.010 (0.002; 0.026)	0.011 (0.002; 0.026)	0.011 (0.002; 0.026)	0.011 (0.002; 0.027)	0.011 (0.002; 0.027)	0.011 (0.002; 0.027)	0.011 (0.002; 0.027)	0.011 (0.002; 0.027)	0.011 (0.002; 0.027)	0.011 (0.002; 0.027)	[illegible]
Zambia	0.022 (0.004; 0.058)	0.022 (0.004; 0.057)	0.022 (0.004; 0.057)	0.022 (0.004; 0.057)	0.022 (0.004; 0.057)	0.021 (0.004; 0.056)	0.021 (0.004; 0.056)	0.021 (0.004; 0.056)	0.021 (0.004; 0.055)	0.021 (0.004; 0.055)	0.021 (0.004; 0.055)	0.021 (0.004; 0.054)	0.021 (0.004; 0.054)	0.021 (0.004; 0.054)	0.021 (0.004; 0.053)	[illegible]
Zimbabwe	0.022 (0.004; 0.058)	0.022 (0.004; 0.057)	0.022 (0.004; 0.057)	0.022 (0.004; 0.057)	0.022 (0.004; 0.057)	0.021 (0.004; 0.056)	0.021 (0.004; 0.056)	0.021 (0.004; 0.056)	0.021 (0.004; 0.055)	0.021 (0.004; 0.055)	0.021 (0.004; 0.055)	0.021 (0.004; 0.054)	0.021 (0.004; 0.054)	0.021 (0.004; 0.054)	0.021 (0.004; 0.053)	[illegible]

Table S18. Population average z-score shifts assuming that the vaccine was only 10% effective against *Shigella* less severe diarrhoea (LSD) and *Shigella*-attributable stunting from LSD episodes. Estimates in parenthesis represent 95% uncertainty estimates from the results of 1000 model simulations.

Country	2025	2026	2027	2028	2029	2030	2031	2032	2033	2034	2035	2036	2037	2038	2039	20[illegible]
Afghanistan	0.003 (0.001; 0.006)	0.003 (0.001; 0.006)	0.003 (0.001; 0.006)	0.003 (0.001; 0.006)	0.003 (0.001; 0.006)	0.003 (0.001; 0.006)	0.003 (0.001; 0.006)	0.003 (0.001; 0.006)	0.003 (0.001; 0.006)	0.003 (0.001; 0.006)	0.003 (0.001; 0.006)	0.003 (0.001; 0.006)	0.003 (0.001; 0.006)	0.003 (0.001; 0.006)	0.003 (0.001; 0.006)	[illegible]
Algeria	0.006 (0.001; 0.012)	0.006 (0.001; 0.012)	0.006 (0.001; 0.012)	0.006 (0.001; 0.012)	0.005 (0.001; 0.012)	0.005 (0.001; 0.012)	0.005 (0.001; 0.012)	0.005 (0.001; 0.012)	0.005 (0.001; 0.012)	0.005 (0.001; 0.012)	0.005 (0.001; 0.012)	0.005 (0.001; 0.012)	0.005 (0.001; 0.012)	0.005 (0.001; 0.012)	0.005 (0.001; 0.012)	[illegible]

Angola	0.004 (0.001; 0.008)	0.004 (0.001; 0.008)	0.004 (0.001; 0.008)	0.004 (0.001; 0.008)	0.004 (0.001; 0.008)	0.004 (0.001; 0.008)	0.004 (0.001; 0.008)	0.004 (0.001; 0.008)	0.004 (0.001; 0.008)	0.004 (0.001; 0.009)	0.004 (0.001; 0.009)	0.004 (0.001; 0.009)	0.004 (0.001; 0.009)	0.004 (0.001; 0.009)	0.004 (0.001; 0.009)	[illegible]
Argentina	0.004 (0.001; 0.008)	0.004 (0.001; 0.008)	0.004 (0.001; 0.008)	0.004 (0.001; 0.008)	0.004 (0.001; 0.008)	0.004 (0.001; 0.008)	0.004 (0.001; 0.008)	0.004 (0.001; 0.008)	0.004 (0.001; 0.008)	0.004 (0.001; 0.008)	0.004 (0.001; 0.008)	0.004 (0.001; 0.008)	0.004 (0.001; 0.008)	0.004 (0.001; 0.008)	0.004 (0.001; 0.008)	[illegible]
Armenia	0.002 (0.001; 0.004)	0.002 (0.001; 0.004)	0.002 (0.001; 0.004)	0.002 (0.001; 0.004)	0.002 (0.001; 0.004)	0.002 (0.001; 0.004)	0.002 (0.001; 0.004)	0.002 (0.001; 0.004)	0.002 (0.001; 0.004)	0.002 (0.001; 0.004)	0.002 (0.001; 0.004)	0.002 (0.001; 0.004)	0.002 (0.001; 0.004)	0.002 (0.001; 0.004)	0.002 (0.001; 0.004)	[illegible]
Azerbaijan	0.002 (0.001; 0.004)	0.002 (0.001; 0.004)	0.002 (0.001; 0.004)	0.002 (0.001; 0.004)	0.002 (0.001; 0.004)	0.002 (0.001; 0.004)	0.002 (0.001; 0.004)	0.002 (0.001; 0.004)	0.002 (0.001; 0.004)	0.002 (0.001; 0.004)	0.002 (0.001; 0.004)	0.002 (0.001; 0.004)	0.002 (0.001; 0.004)	0.002 (0.001; 0.004)	0.002 (0.001; 0.004)	[illegible]
Bangladesh	0.002 (0.001; 0.005)	0.002 (0.001; 0.004)	0.002 (0.001; 0.004)	0.002 (0.001; 0.004)	0.002 (0.001; 0.004)	0.002 (0.001; 0.004)	0.002 (0.001; 0.004)	0.002 (0.001; 0.004)	0.002 (0.000; 0.004)	0.002 (0.000; 0.004)	0.002 (0.000; 0.004)	0.002 (0.000; 0.004)	0.002 (0.000; 0.004)	0.002 (0.000; 0.004)	0.002 (0.000; 0.004)	[illegible]
Belarus	0.002 (0.001; 0.004)	0.002 (0.001; 0.004)	0.002 (0.001; 0.004)	0.002 (0.001; 0.004)	0.002 (0.001; 0.004)	0.002 (0.001; 0.004)	0.002 (0.001; 0.004)	0.002 (0.001; 0.004)	0.002 (0.001; 0.004)	0.002 (0.001; 0.004)	0.002 (0.001; 0.004)	0.002 (0.001; 0.004)	0.002 (0.001; 0.004)	0.002 (0.001; 0.004)	0.002 (0.001; 0.004)	[illegible]
Benin	0.005 (0.001; 0.011)	0.005 (0.001; 0.011)	0.005 (0.001; 0.011)	0.005 (0.001; 0.011)	0.005 (0.001; 0.011)	0.005 (0.001; 0.011)	0.005 (0.001; 0.011)	0.005 (0.001; 0.011)	0.005 (0.001; 0.011)	0.005 (0.001; 0.011)	0.005 (0.001; 0.011)	0.005 (0.001; 0.012)	0.005 (0.001; 0.012)	0.005 (0.001; 0.012)	0.005 (0.001; 0.011)	[illegible]
Bhutan	0.002 (0.001; 0.004)	0.002 (0.001; 0.004)	0.002 (0.001; 0.004)	0.002 (0.001; 0.004)	0.002 (0.001; 0.004)	0.002 (0.000; 0.004)	0.002 (0.000; 0.004)	0.002 (0.000; 0.004)	0.002 (0.000; 0.004)	0.002 (0.000; 0.004)	0.002 (0.000; 0.004)	0.002 (0.000; 0.004)	0.002 (0.000; 0.004)	0.002 (0.000; 0.004)	0.002 (0.000; 0.004)	[illegible]
Bolivia	0.003 (0.001; 0.007)	0.003 (0.001; 0.007)	0.004 (0.001; 0.007)	0.004 (0.001; 0.007)	0.004 (0.001; 0.007)	0.004 (0.001; 0.007)	0.004 (0.001; 0.007)	0.004 (0.001; 0.007)	0.004 (0.001; 0.007)	0.004 (0.001; 0.007)	0.004 (0.001; 0.008)	0.004 (0.001; 0.008)	0.004 (0.001; 0.008)	0.004 (0.001; 0.008)	0.004 (0.001; 0.008)	[illegible]
Botswana	0.006 (0.001; 0.013)	0.006 (0.001; 0.013)	0.006 (0.001; 0.012)	0.006 (0.001; 0.012)	0.006 (0.001; 0.012)	0.006 (0.001; 0.012)	0.006 (0.001; 0.012)	0.006 (0.001; 0.012)	0.006 (0.001; 0.012)	0.006 (0.001; 0.012)	0.006 (0.001; 0.012)	0.006 (0.001; 0.012)	0.006 (0.001; 0.012)	0.006 (0.001; 0.012)	0.006 (0.001; 0.012)	[illegible]
Brazil	0.003 (0.001; 0.007)	0.003 (0.001; 0.007)	0.003 (0.001; 0.007)	0.003 (0.001; 0.007)	0.003 (0.001; 0.007)	0.003 (0.001; 0.007)	0.003 (0.001; 0.007)	0.003 (0.001; 0.007)	0.003 (0.001; 0.007)	0.003 (0.001; 0.007)	0.003 (0.001; 0.007)	0.003 (0.001; 0.007)	0.003 (0.001; 0.007)	0.004 (0.001; 0.007)	0.004 (0.001; 0.007)	[illegible]
Bulgaria	0.002 (0.001; 0.004)	0.002 (0.001; 0.004)	0.002 (0.001; 0.004)	0.002 (0.001; 0.004)	0.002 (0.001; 0.004)	0.002 (0.001; 0.004)	0.002 (0.001; 0.004)	0.002 (0.001; 0.004)	0.002 (0.001; 0.004)	0.002 (0.001; 0.004)	0.002 (0.001; 0.004)	0.002 (0.001; 0.004)	0.002 (0.001; 0.004)	0.002 (0.001; 0.004)	0.002 (0.001; 0.004)	[illegible]

Burkina Faso	0.006 (0.001; 0.012)	0.006 (0.001; 0.012)	0.006 (0.001; 0.012)	0.006 (0.001; 0.012)	0.005 (0.001; 0.012)	0.005 (0.001; 0.012)	0.005 (0.001; 0.012)	0.005 (0.001; 0.012)	0.005 (0.001; 0.012)	0.005 (0.001; 0.012)	0.005 (0.001; 0.012)	0.005 (0.001; 0.012)	0.005 (0.001; 0.012)	0.005 (0.001; 0.012)	0.005 (0.001; 0.012)	[illegible]
Burundi	0.006 (0.001; 0.012)	0.006 (0.001; 0.012)	0.006 (0.001; 0.012)	0.006 (0.001; 0.012)	0.006 (0.001; 0.012)	0.006 (0.001; 0.012)	0.006 (0.001; 0.012)	0.006 (0.001; 0.012)	0.006 (0.001; 0.012)	0.005 (0.001; 0.012)	0.005 (0.001; 0.012)	0.005 (0.001; 0.012)	0.005 (0.001; 0.012)	0.005 (0.001; 0.012)	0.005 (0.001; 0.012)	[illegible]
Cabo Verde	0.006 (0.001; 0.013)	0.006 (0.001; 0.013)	0.006 (0.001; 0.013)	0.006 (0.001; 0.013)	0.006 (0.001; 0.013)	0.006 (0.001; 0.013)	0.006 (0.001; 0.012)	0.006 (0.001; 0.012)	0.006 (0.001; 0.012)	0.006 (0.001; 0.012)	0.006 (0.001; 0.012)	0.006 (0.001; 0.012)	0.006 (0.001; 0.012)	0.006 (0.001; 0.012)	0.006 (0.001; 0.012)	[illegible]
Cambodia	0.001 (0.000; 0.003)	0.001 (0.000; 0.003)	0.001 (0.000; 0.003)	0.001 (0.000; 0.003)	0.001 (0.000; 0.003)	0.001 (0.000; 0.003)	0.001 (0.000; 0.003)	0.001 (0.000; 0.003)	0.001 (0.000; 0.003)	0.001 (0.000; 0.003)	0.001 (0.000; 0.003)	0.001 (0.000; 0.003)	0.001 (0.000; 0.003)	0.001 (0.000; 0.003)	0.001 (0.000; 0.003)	[illegible]
Cameroon	0.004 (0.001; 0.009)	0.004 (0.001; 0.009)	0.004 (0.001; 0.010)	0.004 (0.001; 0.010)	0.004 (0.001; 0.010)	0.004 (0.001; 0.010)	0.005 (0.001; 0.010)	0.005 (0.001; 0.010)	0.005 (0.001; 0.010)	0.005 (0.001; 0.010)	0.005 (0.001; 0.010)	0.005 (0.001; 0.010)	0.005 (0.001; 0.010)	0.005 (0.001; 0.010)	0.005 (0.001; 0.010)	[illegible]
Central African Republic	0.003 (0.001; 0.006)	0.003 (0.001; 0.006)	0.003 (0.001; 0.006)	0.003 (0.001; 0.006)	0.003 (0.001; 0.006)	0.003 (0.001; 0.006)	0.003 (0.001; 0.006)	0.003 (0.001; 0.006)	0.003 (0.001; 0.006)	0.003 (0.001; 0.006)	0.003 (0.001; 0.006)	0.003 (0.001; 0.006)	0.003 (0.001; 0.006)	0.003 (0.001; 0.006)	0.003 (0.001; 0.007)	[illegible]
Chad	0.003 (0.001; 0.007)	0.003 (0.001; 0.007)	0.003 (0.001; 0.007)	0.003 (0.001; 0.007)	0.003 (0.001; 0.007)	0.003 (0.001; 0.007)	0.003 (0.001; 0.007)	0.003 (0.001; 0.007)	0.003 (0.001; 0.007)	0.003 (0.001; 0.007)	0.003 (0.001; 0.008)	0.003 (0.001; 0.008)	0.003 (0.001; 0.008)	0.004 (0.001; 0.008)	0.004 (0.001; 0.008)	[illegible]
China	0.002 (0.000; 0.003)	0.002 (0.000; 0.003)	0.002 (0.000; 0.003)	0.002 (0.000; 0.003)	0.002 (0.000; 0.003)	0.002 (0.000; 0.003)	0.002 (0.000; 0.003)	0.002 (0.000; 0.003)	0.002 (0.000; 0.003)	0.002 (0.000; 0.003)	0.002 (0.000; 0.003)	0.002 (0.000; 0.003)	0.002 (0.000; 0.003)	0.002 (0.000; 0.003)	0.002 (0.000; 0.003)	[illegible]
Colombia	0.004 (0.001; 0.008)	0.004 (0.001; 0.008)	0.004 (0.001; 0.008)	0.004 (0.001; 0.008)	0.004 (0.001; 0.008)	0.004 (0.001; 0.008)	0.004 (0.001; 0.008)	0.004 (0.001; 0.008)	0.004 (0.001; 0.008)	0.004 (0.001; 0.008)	0.004 (0.001; 0.008)	0.004 (0.001; 0.008)	0.004 (0.001; 0.008)	0.004 (0.001; 0.008)	0.004 (0.001; 0.008)	[illegible]
Comoros	0.006 (0.001; 0.012)	0.006 (0.001; 0.012)	0.006 (0.001; 0.012)	0.006 (0.001; 0.012)	0.005 (0.001; 0.012)	0.005 (0.001; 0.012)	0.005 (0.001; 0.012)	0.005 (0.001; 0.012)	0.005 (0.001; 0.012)	0.005 (0.001; 0.012)	0.005 (0.001; 0.012)	0.005 (0.001; 0.012)	0.005 (0.001; 0.012)	0.005 (0.001; 0.012)	0.005 (0.001; 0.012)	[illegible]
Congo, Dem. Rep.	0.004 (0.001; 0.008)	0.004 (0.001; 0.008)	0.004 (0.001; 0.008)	0.004 (0.001; 0.008)	0.004 (0.001; 0.008)	0.004 (0.001; 0.008)	0.004 (0.001; 0.008)	0.004 (0.001; 0.008)	0.004 (0.001; 0.008)	0.004 (0.001; 0.009)	0.004 (0.001; 0.009)	0.004 (0.001; 0.009)	0.004 (0.001; 0.009)	0.004 (0.001; 0.009)	0.004 (0.001; 0.009)	[illegible]
Congo, Rep.	0.005 (0.001; 0.011)	0.005 (0.001; 0.011)	0.005 (0.001; 0.011)	0.005 (0.001; 0.011)	0.005 (0.001; 0.011)	0.005 (0.001; 0.011)	0.005 (0.001; 0.012)	0.005 (0.001; 0.012)	0.005 (0.001; 0.012)	0.005 (0.001; 0.012)	0.005 (0.001; 0.012)	0.005 (0.001; 0.012)	0.005 (0.001; 0.012)	0.005 (0.001; 0.012)	0.005 (0.001; 0.011)	[illegible]

Costa Rica	0.004 (0.001; 0.009)	0.004 (0.001; 0.008)	0.004 (0.001; 0.008)	0.004 (0.001; 0.008)	0.004 (0.001; 0.008)	0.004 (0.001; 0.008)	0.004 (0.001; 0.008)	0.004 (0.001; 0.008)	0.004 (0.001; 0.008)	0.004 (0.001; 0.008)	0.004 (0.001; 0.008)	0.004 (0.001; 0.008)	0.004 (0.001; 0.008)	0.004 (0.001; 0.008)	0.004 (0.001; 0.008)	0.0 (0.0 0.00
Cote d'Ivoire	0.005 (0.001; 0.012)	0.005 (0.001; 0.012)	0.005 (0.001; 0.012)	0.005 (0.001; 0.012)	0.005 (0.001; 0.012)	0.005 (0.001; 0.012)	0.005 (0.001; 0.012)	0.005 (0.001; 0.012)	0.005 (0.001; 0.012)	0.005 (0.001; 0.012)	0.005 (0.001; 0.012)	0.005 (0.001; 0.012)	0.005 (0.001; 0.012)	0.005 (0.001; 0.012)	0.005 (0.001; 0.011)	0.0 (0.0 0.01
Djibouti	0.003 (0.001; 0.007)	0.003 (0.001; 0.007)	0.003 (0.001; 0.007)	0.003 (0.001; 0.007)	0.003 (0.001; 0.006)	0.003 (0.001; 0.006)	0.003 (0.001; 0.006)	0.003 (0.001; 0.006)	0.003 (0.001; 0.006)	0.003 (0.001; 0.006)	0.003 (0.001; 0.006)	0.003 (0.001; 0.006)	0.003 (0.001; 0.006)	0.003 (0.001; 0.006)	0.003 (0.001; 0.006)	0.0 (0.0 0.00
Dominican Republic	0.004 (0.001; 0.008)	0.004 (0.001; 0.008)	0.004 (0.001; 0.008)	0.004 (0.001; 0.008)	0.004 (0.001; 0.008)	0.004 (0.001; 0.008)	0.004 (0.001; 0.008)	0.004 (0.001; 0.008)	0.004 (0.001; 0.008)	0.004 (0.001; 0.008)	0.004 (0.001; 0.008)	0.004 (0.001; 0.008)	0.004 (0.001; 0.008)	0.004 (0.001; 0.008)	0.004 (0.001; 0.008)	0.0 (0.0 0.00
Ecuador	0.004 (0.001; 0.008)	0.004 (0.001; 0.008)	0.004 (0.001; 0.008)	0.004 (0.001; 0.008)	0.004 (0.001; 0.008)	0.004 (0.001; 0.008)	0.004 (0.001; 0.008)	0.004 (0.001; 0.008)	0.004 (0.001; 0.008)	0.004 (0.001; 0.008)	0.004 (0.001; 0.008)	0.004 (0.001; 0.008)	0.004 (0.001; 0.008)	0.004 (0.001; 0.008)	0.004 (0.001; 0.008)	0.0 (0.0 0.00
Egypt, Arab Rep.	0.003 (0.001; 0.007)	0.003 (0.001; 0.007)	0.003 (0.001; 0.007)	0.003 (0.001; 0.007)	0.003 (0.001; 0.007)	0.003 (0.001; 0.007)	0.003 (0.001; 0.007)	0.003 (0.001; 0.007)	0.003 (0.001; 0.007)	0.003 (0.001; 0.007)	0.003 (0.001; 0.007)	0.003 (0.001; 0.007)	0.003 (0.001; 0.007)	0.003 (0.001; 0.007)	0.003 (0.001; 0.007)	0.0 (0.0 0.00
El Salvador	0.004 (0.001; 0.008)	0.004 (0.001; 0.008)	0.004 (0.001; 0.008)	0.004 (0.001; 0.008)	0.004 (0.001; 0.008)	0.004 (0.001; 0.008)	0.004 (0.001; 0.008)	0.004 (0.001; 0.008)	0.004 (0.001; 0.008)	0.004 (0.001; 0.008)	0.004 (0.001; 0.008)	0.004 (0.001; 0.008)	0.004 (0.001; 0.008)	0.004 (0.001; 0.008)	0.004 (0.001; 0.008)	0.0 (0.0 0.00
Eritrea	0.006 (0.001; 0.013)	0.006 (0.001; 0.013)	0.006 (0.001; 0.012)	0.006 (0.001; 0.012)	0.006 (0.001; 0.012)	0.006 (0.001; 0.012)	0.006 (0.001; 0.012)	0.006 (0.001; 0.012)	0.006 (0.001; 0.012)	0.006 (0.001; 0.012)	0.006 (0.001; 0.012)	0.006 (0.001; 0.012)	0.006 (0.001; 0.012)	0.006 (0.001; 0.012)	0.006 (0.001; 0.012)	0.0 (0.0 0.01
Eswatini	0.006 (0.001; 0.012)	0.005 (0.001; 0.012)	0.005 (0.001; 0.012)	0.005 (0.001; 0.012)	0.005 (0.001; 0.012)	0.005 (0.001; 0.012)	0.005 (0.001; 0.012)	0.005 (0.001; 0.012)	0.005 (0.001; 0.012)	0.005 (0.001; 0.012)	0.005 (0.001; 0.012)	0.005 (0.001; 0.012)	0.005 (0.001; 0.012)	0.005 (0.001; 0.012)	0.005 (0.001; 0.011)	0.0 (0.0 0.01
Ethiopia	0.004 (0.001; 0.010)	0.004 (0.001; 0.010)	0.004 (0.001; 0.010)	0.004 (0.001; 0.010)	0.005 (0.001; 0.010)	0.005 (0.001; 0.010)	0.005 (0.001; 0.010)	0.005 (0.001; 0.010)	0.005 (0.001; 0.010)	0.005 (0.001; 0.010)	0.005 (0.001; 0.010)	0.005 (0.001; 0.010)	0.005 (0.001; 0.010)	0.005 (0.001; 0.011)	0.005 (0.001; 0.011)	0.0 (0.0 0.01
Fiji	0.002 (0.000; 0.003)	0.002 (0.000; 0.003)	0.002 (0.000; 0.003)	0.002 (0.000; 0.003)	0.002 (0.000; 0.003)	0.002 (0.000; 0.003)	0.002 (0.000; 0.003)	0.002 (0.000; 0.003)	0.002 (0.000; 0.003)	0.002 (0.000; 0.003)	0.002 (0.000; 0.003)	0.002 (0.000; 0.003)	0.002 (0.000; 0.003)	0.002 (0.000; 0.003)	0.002 (0.000; 0.003)	0.0 (0.0 0.00
Gabon	0.005 (0.001; 0.010)	0.005 (0.001; 0.010)	0.005 (0.001; 0.010)	0.005 (0.001; 0.010)	0.005 (0.001; 0.010)	0.005 (0.001; 0.010)	0.005 (0.001; 0.010)	0.005 (0.001; 0.010)	0.005 (0.001; 0.010)	0.005 (0.001; 0.010)	0.005 (0.001; 0.011)	0.005 (0.001; 0.011)	0.005 (0.001; 0.011)	0.005 (0.001; 0.011)	0.005 (0.001; 0.011)	0.0 (0.0 0.01

Gambia, The	0.006 (0.001; 0.012)	0.005 (0.001; 0.012)	0.005 (0.001; 0.012)	0.005 (0.001; 0.012)	0.005 (0.001; 0.012)	0.005 (0.001; 0.012)	0.005 (0.001; 0.012)	0.005 (0.001; 0.012)	0.005 (0.001; 0.012)	0.005 (0.001; 0.012)	0.005 (0.001; 0.012)	0.005 (0.001; 0.012)	0.005 (0.001; 0.012)	0.005 (0.001; 0.012)	0.005 (0.001; 0.011)	[illegible]
Georgia	0.002 (0.001; 0.004)	0.002 (0.001; 0.004)	0.002 (0.001; 0.004)	0.002 (0.001; 0.004)	0.002 (0.001; 0.004)	0.002 (0.001; 0.004)	0.002 (0.001; 0.004)	0.002 (0.001; 0.004)	0.002 (0.001; 0.004)	0.002 (0.001; 0.004)	0.002 (0.001; 0.004)	0.002 (0.001; 0.004)	0.002 (0.001; 0.004)	0.002 (0.001; 0.004)	0.002 (0.001; 0.004)	[illegible]
Ghana	0.006 (0.001; 0.013)	0.006 (0.001; 0.013)	0.006 (0.001; 0.013)	0.006 (0.001; 0.013)	0.006 (0.001; 0.013)	0.006 (0.001; 0.013)	0.006 (0.001; 0.013)	0.006 (0.001; 0.013)	0.006 (0.001; 0.013)	0.006 (0.001; 0.013)	0.006 (0.001; 0.012)	0.006 (0.001; 0.012)	0.006 (0.001; 0.012)	0.006 (0.001; 0.012)	0.006 (0.001; 0.012)	[illegible]
Guatemala	0.004 (0.001; 0.008)	0.004 (0.001; 0.008)	0.004 (0.001; 0.008)	0.004 (0.001; 0.008)	0.004 (0.001; 0.008)	0.004 (0.001; 0.008)	0.004 (0.001; 0.008)	0.004 (0.001; 0.008)	0.004 (0.001; 0.008)	0.004 (0.001; 0.008)	0.004 (0.001; 0.008)	0.004 (0.001; 0.008)	0.004 (0.001; 0.008)	0.004 (0.001; 0.008)	0.004 (0.001; 0.008)	[illegible]
Guinea	0.003 (0.001; 0.007)	0.003 (0.001; 0.007)	0.003 (0.001; 0.007)	0.003 (0.001; 0.007)	0.003 (0.001; 0.007)	0.003 (0.001; 0.007)	0.003 (0.001; 0.007)	0.003 (0.001; 0.007)	0.003 (0.001; 0.007)	0.003 (0.001; 0.007)	0.003 (0.001; 0.007)	0.003 (0.001; 0.007)	0.003 (0.001; 0.007)	0.003 (0.001; 0.007)	0.003 (0.001; 0.007)	[illegible]
Guinea-Bissau	0.005 (0.001; 0.011)	0.005 (0.001; 0.011)	0.005 (0.001; 0.011)	0.005 (0.001; 0.011)	0.005 (0.001; 0.011)	0.005 (0.001; 0.011)	0.005 (0.001; 0.011)	0.005 (0.001; 0.012)	0.005 (0.001; 0.012)	0.005 (0.001; 0.012)	0.005 (0.001; 0.012)	0.005 (0.001; 0.012)	0.005 (0.001; 0.012)	0.005 (0.001; 0.012)	0.005 (0.001; 0.011)	[illegible]
Haiti	0.002 (0.001; 0.005)	0.002 (0.001; 0.005)	0.002 (0.001; 0.005)	0.002 (0.001; 0.005)	0.002 (0.001; 0.005)	0.002 (0.001; 0.005)	0.002 (0.001; 0.005)	0.002 (0.001; 0.005)	0.002 (0.001; 0.005)	0.002 (0.001; 0.005)	0.003 (0.001; 0.005)	0.003 (0.001; 0.005)	0.003 (0.001; 0.005)	0.003 (0.001; 0.005)	0.003 (0.001; 0.005)	[illegible]
Honduras	0.004 (0.001; 0.008)	0.004 (0.001; 0.008)	0.004 (0.001; 0.008)	0.004 (0.001; 0.008)	0.004 (0.001; 0.008)	0.004 (0.001; 0.008)	0.004 (0.001; 0.008)	0.004 (0.001; 0.008)	0.004 (0.001; 0.008)	0.004 (0.001; 0.008)	0.004 (0.001; 0.008)	0.004 (0.001; 0.008)	0.004 (0.001; 0.008)	0.004 (0.001; 0.008)	0.004 (0.001; 0.008)	[illegible]
India	0.002 (0.000; 0.004)	0.002 (0.000; 0.004)	0.002 (0.000; 0.004)	0.002 (0.000; 0.004)	0.002 (0.000; 0.004)	0.002 (0.000; 0.004)	0.002 (0.000; 0.004)	0.002 (0.000; 0.004)	0.002 (0.000; 0.004)	0.002 (0.000; 0.004)	0.002 (0.000; 0.004)	0.002 (0.000; 0.004)	0.002 (0.000; 0.004)	0.002 (0.000; 0.004)	0.002 (0.000; 0.004)	[illegible]
Indonesia	0.002 (0.000; 0.004)	0.002 (0.000; 0.004)	0.002 (0.000; 0.004)	0.002 (0.000; 0.004)	0.002 (0.000; 0.004)	0.002 (0.000; 0.004)	0.002 (0.000; 0.004)	0.002 (0.000; 0.004)	0.002 (0.000; 0.004)	0.002 (0.000; 0.004)	0.002 (0.000; 0.004)	0.002 (0.000; 0.004)	0.002 (0.000; 0.004)	0.002 (0.000; 0.004)	0.002 (0.000; 0.004)	[illegible]
Iraq	0.003 (0.001; 0.007)	0.003 (0.001; 0.007)	0.003 (0.001; 0.007)	0.003 (0.001; 0.007)	0.003 (0.001; 0.006)	0.003 (0.001; 0.006)	0.003 (0.001; 0.006)	0.003 (0.001; 0.006)	0.003 (0.001; 0.006)	0.003 (0.001; 0.006)	0.003 (0.001; 0.006)	0.003 (0.001; 0.006)	0.003 (0.001; 0.006)	0.003 (0.001; 0.006)	0.003 (0.001; 0.006)	[illegible]
Jamaica	0.004 (0.001; 0.009)	0.004 (0.001; 0.009)	0.004 (0.001; 0.009)	0.004 (0.001; 0.008)	0.004 (0.001; 0.008)	0.004 (0.001; 0.008)	0.004 (0.001; 0.008)	0.004 (0.001; 0.008)	0.004 (0.001; 0.008)	0.004 (0.001; 0.008)	0.004 (0.001; 0.008)	0.004 (0.001; 0.008)	0.004 (0.001; 0.008)	0.004 (0.001; 0.008)	0.004 (0.001; 0.008)	[illegible]

Jordan	0.003 (0.001; 0.007)	0.003 (0.001; 0.007)	0.003 (0.001; 0.007)	0.003 (0.001; 0.007)	0.003 (0.001; 0.006)	0.003 (0.001; 0.006)	0.003 (0.001; 0.006)	0.003 (0.001; 0.006)	0.003 (0.001; 0.006)	0.003 (0.001; 0.006)	0.003 (0.001; 0.006)	0.003 (0.001; 0.006)	0.003 (0.001; 0.006)	0.003 (0.001; 0.006)	0.003 (0.001; 0.006)	0.0 (0.0 0.0
Kazakhstan	0.002 (0.001; 0.004)	0.002 (0.001; 0.004)	0.002 (0.001; 0.004)	0.002 (0.001; 0.004)	0.002 (0.001; 0.004)	0.002 (0.001; 0.004)	0.002 (0.001; 0.004)	0.002 (0.001; 0.004)	0.002 (0.001; 0.004)	0.002 (0.001; 0.004)	0.002 (0.001; 0.004)	0.002 (0.001; 0.004)	0.002 (0.001; 0.004)	0.002 (0.001; 0.004)	0.002 (0.001; 0.004)	0.0 (0.0 0.0
Kenya	0.006 (0.001; 0.012)	0.006 (0.001; 0.012)	0.006 (0.001; 0.012)	0.006 (0.001; 0.012)	0.005 (0.001; 0.012)	0.005 (0.001; 0.012)	0.005 (0.001; 0.012)	0.005 (0.001; 0.012)	0.005 (0.001; 0.012)	0.005 (0.001; 0.012)	0.005 (0.001; 0.012)	0.005 (0.001; 0.012)	0.005 (0.001; 0.012)	0.005 (0.001; 0.012)	0.005 (0.001; 0.012)	0.0 (0.0 0.0
Kiribati	0.002 (0.000; 0.003)	0.002 (0.000; 0.003)	0.002 (0.000; 0.003)	0.002 (0.000; 0.003)	0.002 (0.000; 0.003)	0.002 (0.000; 0.003)	0.002 (0.000; 0.003)	0.002 (0.000; 0.003)	0.002 (0.000; 0.003)	0.002 (0.000; 0.003)	0.002 (0.000; 0.003)	0.002 (0.000; 0.003)	0.002 (0.000; 0.003)	0.001 (0.000; 0.003)	0.001 (0.000; 0.003)	0.0 (0.0 0.0
Kyrgyz Republic	0.002 (0.001; 0.004)	0.002 (0.001; 0.004)	0.002 (0.001; 0.004)	0.002 (0.001; 0.004)	0.002 (0.001; 0.004)	0.002 (0.001; 0.004)	0.002 (0.001; 0.004)	0.002 (0.001; 0.004)	0.002 (0.001; 0.004)	0.002 (0.001; 0.004)	0.002 (0.001; 0.004)	0.002 (0.001; 0.004)	0.002 (0.001; 0.004)	0.002 (0.001; 0.004)	0.002 (0.001; 0.004)	0.0 (0.0 0.0
Lao PDR	0.001 (0.000; 0.003)	0.001 (0.000; 0.003)	0.001 (0.000; 0.003)	0.001 (0.000; 0.003)	0.001 (0.000; 0.003)	0.001 (0.000; 0.003)	0.001 (0.000; 0.003)	0.001 (0.000; 0.003)	0.001 (0.000; 0.003)	0.001 (0.000; 0.003)	0.001 (0.000; 0.003)	0.001 (0.000; 0.003)	0.001 (0.000; 0.003)	0.001 (0.000; 0.003)	0.001 (0.000; 0.003)	0.0 (0.0 0.0
Lesotho	0.006 (0.001; 0.012)	0.005 (0.001; 0.012)	0.005 (0.001; 0.012)	0.005 (0.001; 0.012)	0.005 (0.001; 0.012)	0.005 (0.001; 0.012)	0.005 (0.001; 0.012)	0.005 (0.001; 0.012)	0.005 (0.001; 0.012)	0.005 (0.001; 0.012)	0.005 (0.001; 0.012)	0.005 (0.001; 0.012)	0.005 (0.001; 0.012)	0.005 (0.001; 0.012)	0.005 (0.001; 0.011)	0.0 (0.0 0.0
Liberia	0.005 (0.001; 0.010)	0.005 (0.001; 0.010)	0.005 (0.001; 0.010)	0.005 (0.001; 0.010)	0.005 (0.001; 0.010)	0.005 (0.001; 0.010)	0.005 (0.001; 0.010)	0.005 (0.001; 0.010)	0.005 (0.001; 0.010)	0.005 (0.001; 0.010)	0.005 (0.001; 0.011)	0.005 (0.001; 0.011)	0.005 (0.001; 0.011)	0.005 (0.001; 0.011)	0.005 (0.001; 0.011)	0.0 (0.0 0.0
Madagascar	0.005 (0.001; 0.010)	0.005 (0.001; 0.010)	0.005 (0.001; 0.010)	0.005 (0.001; 0.010)	0.005 (0.001; 0.010)	0.005 (0.001; 0.010)	0.005 (0.001; 0.010)	0.005 (0.001; 0.010)	0.005 (0.001; 0.010)	0.005 (0.001; 0.010)	0.005 (0.001; 0.011)	0.005 (0.001; 0.011)	0.005 (0.001; 0.011)	0.005 (0.001; 0.011)	0.005 (0.001; 0.011)	0.0 (0.0 0.0
Malawi	0.006 (0.001; 0.013)	0.006 (0.001; 0.013)	0.006 (0.001; 0.012)	0.006 (0.001; 0.012)	0.006 (0.001; 0.012)	0.006 (0.001; 0.012)	0.006 (0.001; 0.012)	0.006 (0.001; 0.012)	0.006 (0.001; 0.012)	0.006 (0.001; 0.012)	0.006 (0.001; 0.012)	0.006 (0.001; 0.012)	0.006 (0.001; 0.012)	0.006 (0.001; 0.012)	0.006 (0.001; 0.012)	0.0 (0.0 0.0
Malaysia	0.002 (0.000; 0.003)	0.002 (0.000; 0.003)	0.002 (0.000; 0.003)	0.002 (0.000; 0.003)	0.002 (0.000; 0.003)	0.002 (0.000; 0.003)	0.002 (0.000; 0.003)	0.002 (0.000; 0.003)	0.002 (0.000; 0.003)	0.002 (0.000; 0.003)	0.002 (0.000; 0.003)	0.002 (0.000; 0.003)	0.002 (0.000; 0.003)	0.002 (0.000; 0.003)	0.002 (0.000; 0.003)	0.0 (0.0 0.0
Mali	0.005 (0.001; 0.011)	0.005 (0.001; 0.011)	0.005 (0.001; 0.011)	0.005 (0.001; 0.011)	0.005 (0.001; 0.011)	0.005 (0.001; 0.011)	0.005 (0.001; 0.011)	0.005 (0.001; 0.011)	0.005 (0.001; 0.011)	0.005 (0.001; 0.012)	0.005 (0.001; 0.012)	0.005 (0.001; 0.012)	0.005 (0.001; 0.012)	0.005 (0.001; 0.012)	0.005 (0.001; 0.011)	0.0 (0.0 0.0

Mauritania	0.005 (0.001; 0.011)	0.005 (0.001; 0.011)	0.005 (0.001; 0.012)	0.005 (0.001; 0.012)	0.005 (0.001; 0.012)	0.005 (0.001; 0.012)	0.005 (0.001; 0.012)	0.005 (0.001; 0.012)	0.005 (0.001; 0.012)	0.005 (0.001; 0.012)	0.005 (0.001; 0.012)	0.005 (0.001; 0.012)	0.005 (0.001; 0.012)	0.005 (0.001; 0.012)	0.005 (0.001; 0.011)	[illegible]
Mexico	0.004 (0.001; 0.008)	0.004 (0.001; 0.008)	0.004 (0.001; 0.008)	0.004 (0.001; 0.008)	0.004 (0.001; 0.008)	0.004 (0.001; 0.008)	0.004 (0.001; 0.008)	0.004 (0.001; 0.008)	0.004 (0.001; 0.008)	0.004 (0.001; 0.008)	0.004 (0.001; 0.008)	0.004 (0.001; 0.008)	0.004 (0.001; 0.008)	0.004 (0.001; 0.008)	0.004 (0.001; 0.008)	[illegible]
Mongolia	0.002 (0.000; 0.003)	0.002 (0.000; 0.003)	0.002 (0.000; 0.003)	0.002 (0.000; 0.003)	0.002 (0.000; 0.003)	0.002 (0.000; 0.003)	0.002 (0.000; 0.003)	0.002 (0.000; 0.003)	0.002 (0.000; 0.003)	0.002 (0.000; 0.003)	0.002 (0.000; 0.003)	0.002 (0.000; 0.003)	0.002 (0.000; 0.003)	0.002 (0.000; 0.003)	0.002 (0.000; 0.003)	[illegible]
Morocco	0.003 (0.001; 0.007)	0.003 (0.001; 0.007)	0.003 (0.001; 0.007)	0.003 (0.001; 0.007)	0.003 (0.001; 0.007)	0.003 (0.001; 0.007)	0.003 (0.001; 0.007)	0.003 (0.001; 0.007)	0.003 (0.001; 0.007)	0.003 (0.001; 0.007)	0.003 (0.001; 0.007)	0.003 (0.001; 0.007)	0.003 (0.001; 0.007)	0.003 (0.001; 0.007)	0.003 (0.001; 0.007)	[illegible]
Mozambique	0.006 (0.001; 0.012)	0.005 (0.001; 0.012)	0.005 (0.001; 0.012)	0.005 (0.001; 0.012)	0.005 (0.001; 0.012)	0.005 (0.001; 0.012)	0.005 (0.001; 0.012)	0.005 (0.001; 0.012)	0.005 (0.001; 0.012)	0.005 (0.001; 0.012)	0.005 (0.001; 0.012)	0.005 (0.001; 0.012)	0.005 (0.001; 0.012)	0.005 (0.001; 0.012)	0.005 (0.001; 0.011)	[illegible]
Myanmar	0.002 (0.000; 0.004)	0.002 (0.000; 0.004)	0.002 (0.000; 0.004)	0.002 (0.000; 0.004)	0.002 (0.000; 0.004)	0.002 (0.000; 0.004)	0.002 (0.000; 0.004)	0.002 (0.000; 0.004)	0.002 (0.000; 0.004)	0.002 (0.000; 0.004)	0.002 (0.000; 0.004)	0.002 (0.000; 0.004)	0.002 (0.000; 0.004)	0.002 (0.000; 0.004)	0.002 (0.000; 0.004)	[illegible]
Namibia	0.006 (0.001; 0.012)	0.005 (0.001; 0.012)	0.005 (0.001; 0.012)	0.005 (0.001; 0.012)	0.005 (0.001; 0.012)	0.005 (0.001; 0.012)	0.005 (0.001; 0.012)	0.005 (0.001; 0.012)	0.005 (0.001; 0.012)	0.005 (0.001; 0.012)	0.005 (0.001; 0.012)	0.005 (0.001; 0.012)	0.005 (0.001; 0.012)	0.005 (0.001; 0.012)	0.005 (0.001; 0.011)	[illegible]
Nepal	0.002 (0.000; 0.004)	0.002 (0.000; 0.004)	0.002 (0.000; 0.004)	0.002 (0.000; 0.004)	0.002 (0.000; 0.004)	0.002 (0.000; 0.004)	0.002 (0.000; 0.004)	0.002 (0.000; 0.004)	0.002 (0.000; 0.004)	0.002 (0.000; 0.004)	0.002 (0.000; 0.004)	0.002 (0.000; 0.004)	0.002 (0.000; 0.004)	0.002 (0.000; 0.004)	0.002 (0.000; 0.004)	[illegible]
Nicaragua	0.004 (0.001; 0.009)	0.004 (0.001; 0.009)	0.004 (0.001; 0.009)	0.004 (0.001; 0.009)	0.004 (0.001; 0.009)	0.004 (0.001; 0.009)	0.004 (0.001; 0.009)	0.004 (0.001; 0.009)	0.004 (0.001; 0.008)	0.004 (0.001; 0.008)	0.004 (0.001; 0.008)	0.004 (0.001; 0.008)	0.004 (0.001; 0.008)	0.004 (0.001; 0.008)	0.004 (0.001; 0.008)	[illegible]
Niger	0.005 (0.001; 0.011)	0.005 (0.001; 0.011)	0.005 (0.001; 0.012)	0.005 (0.001; 0.012)	0.005 (0.001; 0.012)	0.005 (0.001; 0.012)	0.005 (0.001; 0.012)	0.005 (0.001; 0.012)	0.005 (0.001; 0.012)	0.005 (0.001; 0.012)	0.005 (0.001; 0.012)	0.005 (0.001; 0.012)	0.005 (0.001; 0.012)	0.005 (0.001; 0.012)	0.005 (0.001; 0.011)	[illegible]
Nigeria	0.004 (0.001; 0.008)	0.004 (0.001; 0.008)	0.004 (0.001; 0.008)	0.004 (0.001; 0.008)	0.004 (0.001; 0.008)	0.004 (0.001; 0.008)	0.004 (0.001; 0.008)	0.004 (0.001; 0.008)	0.004 (0.001; 0.008)	0.004 (0.001; 0.009)	0.004 (0.001; 0.009)	0.004 (0.001; 0.009)	0.004 (0.001; 0.009)	0.004 (0.001; 0.009)	0.004 (0.001; 0.009)	[illegible]
Pakistan	0.003 (0.001; 0.007)	0.003 (0.001; 0.007)	0.003 (0.001; 0.007)	0.003 (0.001; 0.007)	0.003 (0.001; 0.006)	0.003 (0.001; 0.006)	0.003 (0.001; 0.006)	0.003 (0.001; 0.006)	0.003 (0.001; 0.006)	0.003 (0.001; 0.006)	0.003 (0.001; 0.006)	0.003 (0.001; 0.006)	0.003 (0.001; 0.006)	0.003 (0.001; 0.006)	0.003 (0.001; 0.006)	[illegible]

Papua New Guinea	0.001 (0.000; 0.001)	0.001 (0.000; 0.001)	0.001 (0.000; 0.001)	0.001 (0.000; 0.001)	0.001 (0.000; 0.001)	0.001 (0.000; 0.001)	0.001 (0.000; 0.001)	0.001 (0.000; 0.001)	0.001 (0.000; 0.001)	0.001 (0.000; 0.001)	0.001 (0.000; 0.001)	0.001 (0.000; 0.001)	0.001 (0.000; 0.001)	0.001 (0.000; 0.001)	0.001 (0.000; 0.001)	[illegible]
Paraguay	0.004 (0.001; 0.008)	0.004 (0.001; 0.008)	0.004 (0.001; 0.008)	0.004 (0.001; 0.008)	0.004 (0.001; 0.008)	0.004 (0.001; 0.008)	0.004 (0.001; 0.008)	0.004 (0.001; 0.008)	0.004 (0.001; 0.008)	0.004 (0.001; 0.008)	0.004 (0.001; 0.008)	0.004 (0.001; 0.008)	0.004 (0.001; 0.008)	0.004 (0.001; 0.008)	0.004 (0.001; 0.008)	[illegible]
Peru	0.004 (0.001; 0.008)	0.004 (0.001; 0.008)	0.004 (0.001; 0.008)	0.004 (0.001; 0.008)	0.004 (0.001; 0.008)	0.004 (0.001; 0.008)	0.004 (0.001; 0.008)	0.004 (0.001; 0.008)	0.004 (0.001; 0.008)	0.004 (0.001; 0.008)	0.004 (0.001; 0.008)	0.004 (0.001; 0.008)	0.004 (0.001; 0.008)	0.004 (0.001; 0.008)	0.004 (0.001; 0.008)	[illegible]
Philippines	0.001 (0.000; 0.003)	0.001 (0.000; 0.003)	0.001 (0.000; 0.003)	0.001 (0.000; 0.003)	0.001 (0.000; 0.003)	0.001 (0.000; 0.003)	0.001 (0.000; 0.003)	0.001 (0.000; 0.003)	0.001 (0.000; 0.003)	0.001 (0.000; 0.003)	0.001 (0.000; 0.003)	0.001 (0.000; 0.003)	0.001 (0.000; 0.003)	0.001 (0.000; 0.003)	0.001 (0.000; 0.003)	[illegible]
Rwanda	0.006 (0.001; 0.013)	0.006 (0.001; 0.013)	0.006 (0.001; 0.013)	0.006 (0.001; 0.013)	0.006 (0.001; 0.013)	0.006 (0.001; 0.013)	0.006 (0.001; 0.013)	0.006 (0.001; 0.013)	0.006 (0.001; 0.013)	0.006 (0.001; 0.013)	0.006 (0.001; 0.013)	0.006 (0.001; 0.013)	0.006 (0.001; 0.013)	0.006 (0.001; 0.013)	0.006 (0.001; 0.012)	[illegible]
Senegal	0.006 (0.001; 0.013)	0.006 (0.001; 0.013)	0.006 (0.001; 0.012)	0.006 (0.001; 0.012)	0.006 (0.001; 0.012)	0.006 (0.001; 0.012)	0.006 (0.001; 0.012)	0.006 (0.001; 0.012)	0.006 (0.001; 0.012)	0.006 (0.001; 0.012)	0.006 (0.001; 0.012)	0.006 (0.001; 0.012)	0.006 (0.001; 0.012)	0.006 (0.001; 0.012)	0.006 (0.001; 0.012)	[illegible]
Sierra Leone	0.006 (0.001; 0.013)	0.006 (0.001; 0.013)	0.006 (0.001; 0.012)	0.006 (0.001; 0.012)	0.006 (0.001; 0.012)	0.006 (0.001; 0.012)	0.006 (0.001; 0.012)	0.006 (0.001; 0.012)	0.006 (0.001; 0.012)	0.006 (0.001; 0.012)	0.006 (0.001; 0.012)	0.006 (0.001; 0.012)	0.006 (0.001; 0.012)	0.006 (0.001; 0.012)	0.006 (0.001; 0.012)	[illegible]
Solomon Islands	0.002 (0.000; 0.003)	0.002 (0.000; 0.003)	0.002 (0.000; 0.003)	0.002 (0.000; 0.003)	0.001 (0.000; 0.003)	0.001 (0.000; 0.003)	0.001 (0.000; 0.003)	0.001 (0.000; 0.003)	0.001 (0.000; 0.003)	0.001 (0.000; 0.003)	0.001 (0.000; 0.003)	0.001 (0.000; 0.003)	0.001 (0.000; 0.003)	0.001 (0.000; 0.003)	0.001 (0.000; 0.003)	[illegible]
Somalia	0.001 (0.000; 0.003)	0.001 (0.000; 0.003)	0.001 (0.000; 0.003)	0.001 (0.000; 0.003)	0.002 (0.000; 0.003)	0.002 (0.000; 0.003)	0.002 (0.000; 0.003)	0.002 (0.000; 0.003)	0.002 (0.000; 0.003)	0.002 (0.000; 0.003)	0.002 (0.000; 0.003)	0.002 (0.000; 0.003)	0.002 (0.000; 0.004)	0.002 (0.000; 0.004)	0.002 (0.000; 0.004)	[illegible]
South Africa	0.006 (0.001; 0.012)	0.005 (0.001; 0.012)	0.005 (0.001; 0.012)	0.005 (0.001; 0.012)	0.005 (0.001; 0.012)	0.005 (0.001; 0.012)	0.005 (0.001; 0.012)	0.005 (0.001; 0.012)	0.005 (0.001; 0.012)	0.005 (0.001; 0.012)	0.005 (0.001; 0.012)	0.005 (0.001; 0.012)	0.005 (0.001; 0.012)	0.005 (0.001; 0.012)	0.005 (0.001; 0.011)	[illegible]
Sri Lanka	0.002 (0.001; 0.005)	0.002 (0.001; 0.005)	0.002 (0.001; 0.005)	0.002 (0.001; 0.005)	0.002 (0.001; 0.004)	0.002 (0.001; 0.004)	0.002 (0.001; 0.004)	0.002 (0.001; 0.004)	0.002 (0.001; 0.004)	0.002 (0.001; 0.004)	0.002 (0.000; 0.004)	0.002 (0.000; 0.004)	0.002 (0.000; 0.004)	0.002 (0.000; 0.004)	0.002 (0.000; 0.004)	[illegible]
Sudan	0.003 (0.001; 0.007)	0.003 (0.001; 0.007)	0.003 (0.001; 0.007)	0.003 (0.001; 0.007)	0.003 (0.001; 0.007)	0.003 (0.001; 0.007)	0.003 (0.001; 0.007)	0.003 (0.001; 0.007)	0.003 (0.001; 0.007)	0.003 (0.001; 0.007)	0.003 (0.001; 0.007)	0.003 (0.001; 0.007)	0.003 (0.001; 0.007)	0.003 (0.001; 0.006)	0.003 (0.001; 0.006)	[illegible]

Suriname	0.004 (0.001; 0.007)	0.004 (0.001; 0.007)	0.004 (0.001; 0.007)	0.004 (0.001; 0.007)	0.004 (0.001; 0.007)	0.004 (0.001; 0.008)	0.004 (0.001; 0.008)	0.004 (0.001; 0.008)	0.004 (0.001; 0.008)	0.004 (0.001; 0.008)	0.004 (0.001; 0.008)	0.004 (0.001; 0.008)	0.004 (0.001; 0.008)	0.004 (0.001; 0.008)	0.004 (0.001; 0.008)	[illegible]
Tajikistan	0.002 (0.001; 0.004)	0.002 (0.001; 0.004)	0.002 (0.001; 0.004)	0.002 (0.001; 0.004)	0.002 (0.001; 0.004)	0.002 (0.001; 0.004)	0.002 (0.001; 0.004)	0.002 (0.001; 0.004)	0.002 (0.001; 0.004)	0.002 (0.001; 0.004)	0.002 (0.001; 0.004)	0.002 (0.001; 0.004)	0.002 (0.001; 0.004)	0.002 (0.001; 0.004)	0.002 (0.001; 0.004)	[illegible]
Tanzania	0.006 (0.001; 0.012)	0.005 (0.001; 0.012)	0.005 (0.001; 0.012)	0.005 (0.001; 0.012)	0.005 (0.001; 0.012)	0.005 (0.001; 0.012)	0.005 (0.001; 0.012)	0.005 (0.001; 0.012)	0.005 (0.001; 0.012)	0.005 (0.001; 0.012)	0.005 (0.001; 0.012)	0.005 (0.001; 0.012)	0.005 (0.001; 0.012)	0.005 (0.001; 0.012)	0.005 (0.001; 0.011)	[illegible]
Thailand	0.002 (0.001; 0.004)	0.002 (0.001; 0.004)	0.002 (0.001; 0.004)	0.002 (0.001; 0.004)	0.002 (0.001; 0.004)	0.002 (0.000; 0.004)	0.002 (0.000; 0.004)	0.002 (0.000; 0.004)	0.002 (0.000; 0.004)	0.002 (0.000; 0.004)	0.002 (0.000; 0.004)	0.002 (0.000; 0.004)	0.002 (0.000; 0.004)	0.002 (0.000; 0.004)	0.002 (0.000; 0.004)	[illegible]
Timor-Leste	0.002 (0.000; 0.004)	0.002 (0.000; 0.004)	0.002 (0.000; 0.004)	0.002 (0.000; 0.004)	0.002 (0.000; 0.004)	0.002 (0.000; 0.004)	0.002 (0.000; 0.004)	0.002 (0.000; 0.004)	0.002 (0.000; 0.004)	0.002 (0.000; 0.004)	0.002 (0.000; 0.004)	0.002 (0.000; 0.004)	0.002 (0.000; 0.004)	0.002 (0.000; 0.004)	0.002 (0.000; 0.004)	[illegible]
Togo	0.005 (0.001; 0.012)	0.005 (0.001; 0.012)	0.005 (0.001; 0.012)	0.005 (0.001; 0.012)	0.005 (0.001; 0.012)	0.005 (0.001; 0.012)	0.005 (0.001; 0.012)	0.005 (0.001; 0.012)	0.005 (0.001; 0.012)	0.005 (0.001; 0.012)	0.005 (0.001; 0.012)	0.005 (0.001; 0.012)	0.005 (0.001; 0.012)	0.005 (0.001; 0.012)	0.005 (0.001; 0.011)	[illegible]
Tunisia	0.003 (0.001; 0.007)	0.003 (0.001; 0.007)	0.003 (0.001; 0.007)	0.003 (0.001; 0.007)	0.003 (0.001; 0.007)	0.003 (0.001; 0.007)	0.003 (0.001; 0.007)	0.003 (0.001; 0.007)	0.003 (0.001; 0.007)	0.003 (0.001; 0.007)	0.003 (0.001; 0.006)	0.003 (0.001; 0.006)	0.003 (0.001; 0.006)	0.003 (0.001; 0.006)	0.003 (0.001; 0.006)	[illegible]
Turkey	0.002 (0.001; 0.004)	0.002 (0.001; 0.004)	0.002 (0.001; 0.004)	0.002 (0.001; 0.004)	0.002 (0.001; 0.004)	0.002 (0.001; 0.004)	0.002 (0.001; 0.004)	0.002 (0.001; 0.004)	0.002 (0.001; 0.004)	0.002 (0.001; 0.004)	0.002 (0.001; 0.004)	0.002 (0.001; 0.004)	0.002 (0.001; 0.004)	0.002 (0.001; 0.004)	0.002 (0.001; 0.004)	[illegible]
Uganda	0.006 (0.001; 0.012)	0.006 (0.001; 0.012)	0.006 (0.001; 0.012)	0.006 (0.001; 0.012)	0.006 (0.001; 0.012)	0.006 (0.001; 0.012)	0.006 (0.001; 0.012)	0.006 (0.001; 0.012)	0.006 (0.001; 0.012)	0.005 (0.001; 0.012)	0.005 (0.001; 0.012)	0.005 (0.001; 0.012)	0.005 (0.001; 0.012)	0.005 (0.001; 0.012)	0.005 (0.001; 0.012)	[illegible]
Ukraine	0.002 (0.000; 0.004)	0.002 (0.000; 0.004)	0.002 (0.000; 0.004)	0.002 (0.001; 0.004)	0.002 (0.001; 0.004)	0.002 (0.001; 0.004)	0.002 (0.001; 0.004)	0.002 (0.001; 0.004)	0.002 (0.001; 0.004)	0.002 (0.001; 0.004)	0.002 (0.001; 0.004)	0.002 (0.000; 0.004)	0.002 (0.000; 0.004)	0.002 (0.000; 0.004)	0.002 (0.000; 0.004)	[illegible]
Uzbekistan	0.002 (0.001; 0.004)	0.002 (0.001; 0.004)	0.002 (0.001; 0.004)	0.002 (0.001; 0.004)	0.002 (0.001; 0.004)	0.002 (0.001; 0.004)	0.002 (0.001; 0.004)	0.002 (0.001; 0.004)	0.002 (0.001; 0.004)	0.002 (0.001; 0.004)	0.002 (0.001; 0.004)	0.002 (0.001; 0.004)	0.002 (0.001; 0.004)	0.002 (0.001; 0.004)	0.002 (0.001; 0.004)	[illegible]
Vanuatu	0.001 (0.000; 0.003)	0.001 (0.000; 0.003)	0.001 (0.000; 0.003)	0.001 (0.000; 0.003)	0.001 (0.000; 0.003)	0.001 (0.000; 0.003)	0.001 (0.000; 0.003)	0.001 (0.000; 0.003)	0.001 (0.000; 0.003)	0.001 (0.000; 0.003)	0.001 (0.000; 0.003)	0.001 (0.000; 0.003)	0.001 (0.000; 0.003)	0.001 (0.000; 0.003)	0.001 (0.000; 0.003)	[illegible]

Vietnam	0.001 (0.000; 0.003)	0.001 (0.000; 0.003)	0.001 (0.000; 0.003)	0.001 (0.000; 0.003)	0.001 (0.000; 0.003)	0.001 (0.000; 0.003)	0.001 (0.000; 0.003)	0.001 (0.000; 0.003)	0.001 (0.000; 0.003)	0.001 (0.000; 0.003)	0.001 (0.000; 0.003)	0.001 (0.000; 0.003)	0.001 (0.000; 0.003)	0.001 (0.000; 0.003)	0.001 (0.000; 0.003)	[illegible]
Yemen, Rep.	0.003 (0.001; 0.006)	0.003 (0.001; 0.006)	0.003 (0.001; 0.006)	0.003 (0.001; 0.006)	0.003 (0.001; 0.006)	0.003 (0.001; 0.006)	0.003 (0.001; 0.006)	0.003 (0.001; 0.006)	0.003 (0.001; 0.006)	0.003 (0.001; 0.006)	0.003 (0.001; 0.006)	0.003 (0.001; 0.006)	0.003 (0.001; 0.006)	0.003 (0.001; 0.006)	0.003 (0.001; 0.006)	[illegible]
Zambia	0.006 (0.001; 0.012)	0.005 (0.001; 0.012)	0.005 (0.001; 0.012)	0.005 (0.001; 0.012)	0.005 (0.001; 0.012)	0.005 (0.001; 0.012)	0.005 (0.001; 0.012)	0.005 (0.001; 0.012)	0.005 (0.001; 0.012)	0.005 (0.001; 0.012)	0.005 (0.001; 0.012)	0.005 (0.001; 0.012)	0.005 (0.001; 0.012)	0.005 (0.001; 0.012)	0.005 (0.001; 0.011)	[illegible]
Zimbabwe	0.006 (0.001; 0.012)	0.005 (0.001; 0.012)	0.005 (0.001; 0.012)	0.005 (0.001; 0.012)	0.005 (0.001; 0.012)	0.005 (0.001; 0.012)	0.005 (0.001; 0.012)	0.005 (0.001; 0.012)	0.005 (0.001; 0.012)	0.005 (0.001; 0.012)	0.005 (0.001; 0.012)	0.005 (0.001; 0.012)	0.005 (0.001; 0.012)	0.005 (0.001; 0.012)	0.005 (0.001; 0.011)	[illegible]

Table S19. Population average z-score shifts assuming that the vaccine was 60% (40-80% range) effective against *Shigella* moderate-to-severe diarrhoea MSD) and *Shigella*-attributable stunting from MSD episodes. Estimates in parenthesis represent 95% uncertainty estimates from the results of 1000 model simulations.

Country	2025	2026	2027	2028	2029	2030	2031	2032	2033	2034	2035	2036	2037	2038	2039	2040
Afghanistan	0.007 (0.002; 0.017)	0.007 (0.002; 0.017)	0.007 (0.002; 0.017)	0.007 (0.002; 0.017)	0.007 (0.002; 0.017)	0.008 (0.002; 0.017)	0.008 (0.002; 0.017)	0.008 (0.002; 0.017)	0.008 (0.002; 0.017)	0.008 (0.002; 0.017)	0.008 (0.002; 0.018)	0.008 (0.002; 0.018)	0.008 (0.002; 0.018)	0.008 (0.002; 0.018)	0.008 (0.002; 0.018)	0.008 (0.002; 0.018)
Algeria	0.016 (0.005; 0.034)	0.016 (0.005; 0.034)	0.016 (0.005; 0.034)	0.016 (0.005; 0.034)	0.016 (0.005; 0.034)	0.016 (0.005; 0.034)	0.016 (0.005; 0.034)	0.016 (0.005; 0.033)	0.015 (0.005; 0.033)	0.015 (0.005; 0.033)	0.015 (0.005; 0.033)	0.015 (0.005; 0.033)	0.015 (0.005; 0.033)	0.015 (0.005; 0.033)	0.015 (0.005; 0.033)	0.015 (0.005; 0.032)
Angola	0.011 (0.003; 0.023)	0.011 (0.003; 0.023)	0.011 (0.003; 0.023)	0.011 (0.003; 0.023)	0.011 (0.003; 0.023)	0.011 (0.004; 0.024)	0.011 (0.004; 0.024)	0.011 (0.004; 0.024)	0.011 (0.004; 0.024)	0.011 (0.004; 0.024)	0.011 (0.004; 0.024)	0.011 (0.004; 0.024)	0.011 (0.004; 0.025)	0.011 (0.004; 0.025)	0.012 (0.004; 0.025)	0.012 (0.004; 0.025)
Argentina	0.011 (0.004; 0.023)	0.011 (0.004; 0.023)	0.011 (0.004; 0.023)	0.011 (0.004; 0.023)	0.011 (0.004; 0.023)	0.011 (0.004; 0.023)	0.011 (0.004; 0.022)	0.011 (0.004; 0.022)	0.011 (0.004; 0.022)	0.011 (0.004; 0.022)	0.011 (0.004; 0.022)	0.011 (0.004; 0.022)	0.011 (0.004; 0.022)	0.011 (0.004; 0.022)	0.011 (0.004; 0.022)	0.011 (0.004; 0.022)
Armenia	0.005 (0.002; 0.012)	0.005 (0.002; 0.012)	0.005 (0.002; 0.012)	0.005 (0.002; 0.012)	0.005 (0.002; 0.012)	0.005 (0.002; 0.012)	0.005 (0.002; 0.012)	0.005 (0.002; 0.012)	0.005 (0.002; 0.012)	0.005 (0.002; 0.012)	0.005 (0.002; 0.012)	0.005 (0.002; 0.012)	0.005 (0.002; 0.012)	0.005 (0.002; 0.011)	0.005 (0.002; 0.011)	0.005 (0.002; 0.011)

Azerbaijan	0.006 (0.002; 0.012)	0.006 (0.002; 0.012)	0.006 (0.002; 0.012)	0.006 (0.002; 0.012)	0.006 (0.002; 0.012)	0.005 (0.002; 0.012)	0.005 (0.002; 0.012)	0.005 (0.002; 0.012)	0.005 (0.002; 0.012)	0.005 (0.002; 0.012)	0.005 (0.002; 0.012)	0.005 (0.002; 0.012)	0.005 (0.002; 0.012)	0.005 (0.002; 0.012)	0.005 (0.002; 0.012)	0.005 (0.002; 0.012)
Bangladesh	0.006 (0.002; 0.013)	0.006 (0.002; 0.013)	0.006 (0.002; 0.013)	0.006 (0.002; 0.013)	0.006 (0.002; 0.013)	0.006 (0.002; 0.013)	0.006 (0.002; 0.013)	0.006 (0.002; 0.013)	0.006 (0.002; 0.013)	0.006 (0.002; 0.013)	0.006 (0.002; 0.013)	0.006 (0.002; 0.013)	0.006 (0.002; 0.013)	0.006 (0.002; 0.012)	0.006 (0.002; 0.012)	0.006 (0.002; 0.012)
Belarus	0.006 (0.002; 0.013)	0.006 (0.002; 0.013)	0.006 (0.002; 0.013)	0.006 (0.002; 0.013)	0.006 (0.002; 0.013)	0.006 (0.002; 0.013)	0.006 (0.002; 0.013)	0.006 (0.002; 0.012)	0.006 (0.002; 0.012)	0.006 (0.002; 0.012)	0.006 (0.002; 0.012)	0.006 (0.002; 0.012)	0.006 (0.002; 0.012)	0.006 (0.002; 0.012)	0.006 (0.002; 0.012)	0.006 (0.002; 0.012)
Benin	0.014 (0.005; 0.030)	0.014 (0.005; 0.031)	0.014 (0.005; 0.031)	0.014 (0.005; 0.031)	0.014 (0.005; 0.031)	0.015 (0.005; 0.031)	0.015 (0.005; 0.032)	0.015 (0.005; 0.032)	0.015 (0.005; 0.032)	0.015 (0.005; 0.032)	0.015 (0.005; 0.032)	0.015 (0.005; 0.033)	0.015 (0.005; 0.032)	0.015 (0.005; 0.032)	0.015 (0.005; 0.032)	0.015 (0.005; 0.032)
Bhutan	0.006 (0.002; 0.013)	0.006 (0.002; 0.013)	0.006 (0.002; 0.013)	0.006 (0.002; 0.013)	0.006 (0.002; 0.013)	0.006 (0.002; 0.013)	0.006 (0.002; 0.013)	0.006 (0.002; 0.013)	0.006 (0.002; 0.013)	0.006 (0.002; 0.013)	0.006 (0.002; 0.012)	0.006 (0.002; 0.012)	0.006 (0.002; 0.012)	0.006 (0.002; 0.012)	0.006 (0.002; 0.012)	0.006 (0.002; 0.012)
Bolivia	0.010 (0.004; 0.020)	0.010 (0.004; 0.020)	0.010 (0.004; 0.021)	0.010 (0.004; 0.021)	0.010 (0.004; 0.021)	0.010 (0.004; 0.021)	0.010 (0.004; 0.021)	0.011 (0.004; 0.021)	0.011 (0.004; 0.021)	0.011 (0.004; 0.021)	0.011 (0.004; 0.022)	0.011 (0.004; 0.022)	0.011 (0.004; 0.022)	0.011 (0.004; 0.022)	0.011 (0.004; 0.022)	0.011 (0.004; 0.022)
Botswana	0.017 (0.005; 0.036)	0.017 (0.005; 0.036)	0.017 (0.005; 0.036)	0.016 (0.005; 0.035)	0.016 (0.005; 0.035)	0.016 (0.005; 0.035)	0.016 (0.005; 0.035)	0.016 (0.005; 0.035)	0.016 (0.005; 0.035)	0.016 (0.005; 0.035)	0.016 (0.005; 0.035)	0.016 (0.005; 0.034)	0.016 (0.005; 0.034)	0.016 (0.005; 0.034)	0.016 (0.005; 0.034)	0.016 (0.005; 0.034)
Brazil	0.009 (0.003; 0.019)	0.009 (0.003; 0.019)	0.010 (0.003; 0.019)	0.010 (0.003; 0.019)	0.010 (0.003; 0.019)	0.010 (0.003; 0.020)	0.010 (0.003; 0.020)	0.010 (0.003; 0.020)	0.010 (0.003; 0.020)	0.010 (0.003; 0.020)	0.010 (0.004; 0.020)	0.010 (0.004; 0.020)	0.010 (0.004; 0.020)	0.010 (0.004; 0.021)	0.010 (0.004; 0.021)	0.010 (0.004; 0.021)
Bulgaria	0.006 (0.002; 0.012)	0.006 (0.002; 0.012)	0.005 (0.002; 0.012)	0.005 (0.002; 0.012)	0.005 (0.002; 0.012)	0.005 (0.002; 0.012)	0.005 (0.002; 0.012)	0.005 (0.002; 0.012)	0.005 (0.002; 0.012)	0.005 (0.002; 0.012)	0.005 (0.002; 0.012)	0.005 (0.002; 0.012)	0.005 (0.002; 0.012)	0.005 (0.002; 0.012)	0.005 (0.002; 0.012)	0.005 (0.002; 0.012)
Burkina Faso	0.016 (0.005; 0.034)	0.016 (0.005; 0.034)	0.016 (0.005; 0.034)	0.016 (0.005; 0.034)	0.016 (0.005; 0.034)	0.016 (0.005; 0.034)	0.016 (0.005; 0.034)	0.016 (0.005; 0.033)	0.015 (0.005; 0.033)	0.015 (0.005; 0.033)	0.015 (0.005; 0.033)	0.015 (0.005; 0.033)	0.015 (0.005; 0.033)	0.015 (0.005; 0.033)	0.015 (0.005; 0.033)	0.015 (0.005; 0.032)
Burundi	0.016 (0.005; 0.035)	0.016 (0.005; 0.035)	0.016 (0.005; 0.035)	0.016 (0.005; 0.035)	0.016 (0.005; 0.035)	0.016 (0.005; 0.034)	0.016 (0.005; 0.034)	0.016 (0.005; 0.034)	0.016 (0.005; 0.034)	0.016 (0.005; 0.034)	0.016 (0.005; 0.034)	0.016 (0.005; 0.034)	0.016 (0.005; 0.034)	0.016 (0.005; 0.033)	0.015 (0.005; 0.033)	0.015 (0.005; 0.033)
Cabo Verde	0.017 (0.005; 0.036)	0.017 (0.005; 0.036)	0.017 (0.005; 0.036)	0.017 (0.005; 0.036)	0.017 (0.005; 0.036)	0.017 (0.005; 0.036)	0.016 (0.005; 0.035)	0.016 (0.005; 0.035)	0.016 (0.005; 0.035)	0.016 (0.005; 0.035)	0.016 (0.005; 0.035)	0.016 (0.005; 0.035)	0.016 (0.005; 0.035)	0.016 (0.005; 0.035)	0.016 (0.005; 0.034)	0.016 (0.005; 0.034)

Cambodia	0.004 (0.002; 0.009)	0.004 (0.002; 0.009)	0.004 (0.002; 0.009)	0.004 (0.002; 0.009)	0.004 (0.001; 0.009)	0.004 (0.001; 0.009)	0.004 (0.001; 0.009)	0.004 (0.001; 0.009)	0.004 (0.001; 0.009)	0.004 (0.001; 0.009)	0.004 (0.001; 0.008)	0.004 (0.001; 0.008)	0.004 (0.001; 0.008)	0.004 (0.001; 0.008)	0.004 (0.001; 0.008)	0.004 (0.001; 0.008)
Cameroon	0.012 (0.004; 0.027)	0.013 (0.004; 0.027)	0.013 (0.004; 0.027)	0.013 (0.004; 0.027)	0.013 (0.004; 0.027)	0.013 (0.004; 0.028)	0.013 (0.004; 0.028)	0.013 (0.004; 0.028)	0.013 (0.004; 0.028)	0.013 (0.004; 0.028)	0.013 (0.004; 0.029)	0.013 (0.004; 0.029)	0.013 (0.004; 0.029)	0.014 (0.004; 0.029)	0.014 (0.004; 0.029)	0.014 (0.004; 0.029)
Central African Republic	0.008 (0.002; 0.017)	0.008 (0.003; 0.017)	0.008 (0.003; 0.017)	0.008 (0.003; 0.017)	0.008 (0.003; 0.017)	0.008 (0.003; 0.017)	0.008 (0.003; 0.017)	0.008 (0.003; 0.018)	0.008 (0.003; 0.018)	0.008 (0.003; 0.018)	0.008 (0.003; 0.018)	0.008 (0.003; 0.018)	0.008 (0.003; 0.018)	0.008 (0.003; 0.018)	0.009 (0.003; 0.018)	0.009 (0.003; 0.018)
Chad	0.009 (0.003; 0.020)	0.009 (0.003; 0.020)	0.009 (0.003; 0.020)	0.009 (0.003; 0.020)	0.010 (0.003; 0.021)	0.010 (0.003; 0.021)	0.010 (0.003; 0.021)	0.010 (0.003; 0.021)	0.010 (0.003; 0.021)	0.010 (0.003; 0.021)	0.010 (0.003; 0.021)	0.010 (0.003; 0.021)	0.010 (0.003; 0.022)	0.010 (0.003; 0.022)	0.010 (0.003; 0.022)	0.010 (0.003; 0.022)
China	0.005 (0.002; 0.009)	0.005 (0.002; 0.009)	0.005 (0.002; 0.009)	0.005 (0.002; 0.009)	0.005 (0.002; 0.009)	0.005 (0.002; 0.009)	0.004 (0.002; 0.009)	0.004 (0.002; 0.009)	0.004 (0.002; 0.009)	0.004 (0.002; 0.009)	0.004 (0.002; 0.009)	0.004 (0.002; 0.009)	0.004 (0.002; 0.009)	0.004 (0.002; 0.009)	0.004 (0.002; 0.009)	0.004 (0.002; 0.009)
Colombia	0.012 (0.004; 0.024)	0.012 (0.004; 0.024)	0.012 (0.004; 0.024)	0.012 (0.004; 0.024)	0.012 (0.004; 0.024)	0.012 (0.004; 0.024)	0.012 (0.004; 0.023)	0.012 (0.004; 0.023)	0.012 (0.004; 0.023)	0.011 (0.004; 0.023)	0.011 (0.004; 0.023)	0.011 (0.004; 0.023)	0.011 (0.004; 0.023)	0.011 (0.004; 0.023)	0.011 (0.004; 0.023)	0.011 (0.004; 0.023)
Comoros	0.016 (0.005; 0.034)	0.016 (0.005; 0.034)	0.016 (0.005; 0.034)	0.016 (0.005; 0.034)	0.016 (0.005; 0.034)	0.016 (0.005; 0.034)	0.016 (0.005; 0.034)	0.016 (0.005; 0.033)	0.015 (0.005; 0.033)	0.015 (0.005; 0.033)	0.015 (0.005; 0.033)	0.015 (0.005; 0.033)	0.015 (0.005; 0.033)	0.015 (0.005; 0.033)	0.015 (0.005; 0.033)	0.015 (0.005; 0.032)
Congo, Dem. Rep.	0.011 (0.003; 0.023)	0.011 (0.003; 0.023)	0.011 (0.003; 0.023)	0.011 (0.003; 0.023)	0.011 (0.003; 0.023)	0.011 (0.004; 0.024)	0.011 (0.004; 0.024)	0.011 (0.004; 0.024)	0.011 (0.004; 0.024)	0.011 (0.004; 0.024)	0.011 (0.004; 0.024)	0.011 (0.004; 0.024)	0.011 (0.004; 0.025)	0.011 (0.004; 0.025)	0.012 (0.004; 0.025)	0.012 (0.004; 0.025)
Congo, Rep.	0.015 (0.005; 0.032)	0.015 (0.005; 0.032)	0.015 (0.005; 0.032)	0.015 (0.005; 0.032)	0.015 (0.005; 0.032)	0.015 (0.005; 0.033)	0.015 (0.005; 0.033)	0.015 (0.005; 0.033)	0.015 (0.005; 0.033)	0.015 (0.005; 0.033)	0.015 (0.005; 0.033)	0.015 (0.005; 0.033)	0.015 (0.005; 0.032)	0.015 (0.005; 0.032)	0.015 (0.005; 0.032)	0.015 (0.005; 0.032)
Costa Rica	0.012 (0.004; 0.024)	0.012 (0.004; 0.024)	0.012 (0.004; 0.024)	0.012 (0.004; 0.024)	0.012 (0.004; 0.024)	0.012 (0.004; 0.024)	0.012 (0.004; 0.024)	0.012 (0.004; 0.024)	0.012 (0.004; 0.023)	0.012 (0.004; 0.023)	0.012 (0.004; 0.023)	0.012 (0.004; 0.023)	0.011 (0.004; 0.023)	0.011 (0.004; 0.023)	0.011 (0.004; 0.023)	0.011 (0.004; 0.023)
Cote d'Ivoire	0.016 (0.005; 0.034)	0.016 (0.005; 0.034)	0.016 (0.005; 0.034)	0.016 (0.005; 0.034)	0.016 (0.005; 0.033)	0.015 (0.005; 0.033)	0.015 (0.005; 0.033)	0.015 (0.005; 0.033)	0.015 (0.005; 0.033)	0.015 (0.005; 0.033)	0.015 (0.005; 0.033)	0.015 (0.005; 0.033)	0.015 (0.005; 0.032)	0.015 (0.005; 0.032)	0.015 (0.005; 0.032)	0.015 (0.005; 0.032)
Djibouti	0.009 (0.003; 0.020)	0.009 (0.003; 0.020)	0.009 (0.003; 0.019)	0.008 (0.003; 0.019)	0.008 (0.003; 0.019)	0.008 (0.003; 0.019)	0.008 (0.003; 0.019)	0.008 (0.003; 0.019)	0.008 (0.003; 0.019)	0.008 (0.003; 0.019)	0.008 (0.003; 0.019)	0.008 (0.003; 0.019)	0.008 (0.003; 0.019)	0.008 (0.003; 0.018)	0.008 (0.003; 0.018)	0.008 (0.003; 0.018)

Dominican Republic	0.011 (0.004; 0.023)	0.011 (0.004; 0.023)	0.011 (0.004; 0.023)	0.011 (0.004; 0.023)	0.011 (0.004; 0.023)	0.011 (0.004; 0.023)	0.011 (0.004; 0.022)	0.011 (0.004; 0.022)	0.011 (0.004; 0.022)	0.011 (0.004; 0.022)	0.011 (0.004; 0.022)	0.011 (0.004; 0.022)	0.011 (0.004; 0.022)	0.011 (0.004; 0.022)	0.011 (0.004; 0.022)	0.011 (0.004; 0.022)
Ecuador	0.011 (0.004; 0.023)	0.011 (0.004; 0.023)	0.011 (0.004; 0.023)	0.011 (0.004; 0.023)	0.011 (0.004; 0.023)	0.011 (0.004; 0.023)	0.011 (0.004; 0.022)	0.011 (0.004; 0.022)	0.011 (0.004; 0.022)	0.011 (0.004; 0.022)	0.011 (0.004; 0.022)	0.011 (0.004; 0.022)	0.011 (0.004; 0.022)	0.011 (0.004; 0.022)	0.011 (0.004; 0.022)	0.011 (0.004; 0.022)
Egypt, Arab Rep.	0.009 (0.003; 0.021)	0.009 (0.003; 0.021)	0.009 (0.003; 0.020)	0.009 (0.003; 0.020)	0.009 (0.003; 0.020)	0.009 (0.003; 0.020)	0.009 (0.003; 0.020)	0.009 (0.003; 0.020)	0.009 (0.003; 0.020)	0.009 (0.003; 0.020)	0.009 (0.003; 0.020)	0.009 (0.003; 0.020)	0.009 (0.003; 0.020)	0.009 (0.003; 0.020)	0.009 (0.003; 0.019)	0.009 (0.003; 0.019)
El Salvador	0.011 (0.004; 0.022)	0.011 (0.004; 0.022)	0.011 (0.004; 0.022)	0.011 (0.004; 0.022)	0.011 (0.004; 0.023)	0.011 (0.004; 0.023)	0.011 (0.004; 0.022)	0.011 (0.004; 0.022)	0.011 (0.004; 0.022)	0.011 (0.004; 0.022)	0.011 (0.004; 0.022)	0.011 (0.004; 0.022)	0.011 (0.004; 0.022)	0.011 (0.004; 0.022)	0.011 (0.004; 0.022)	0.011 (0.004; 0.022)
Eritrea	0.017 (0.005; 0.036)	0.017 (0.005; 0.036)	0.017 (0.005; 0.036)	0.016 (0.005; 0.035)	0.016 (0.005; 0.035)	0.016 (0.005; 0.035)	0.016 (0.005; 0.035)	0.016 (0.005; 0.035)	0.016 (0.005; 0.035)	0.016 (0.005; 0.035)	0.016 (0.005; 0.035)	0.016 (0.005; 0.034)	0.016 (0.005; 0.034)	0.016 (0.005; 0.034)	0.016 (0.005; 0.034)	0.016 (0.005; 0.034)
Eswatini	0.016 (0.005; 0.034)	0.016 (0.005; 0.034)	0.016 (0.005; 0.034)	0.016 (0.005; 0.034)	0.016 (0.005; 0.033)	0.015 (0.005; 0.033)	0.015 (0.005; 0.033)	0.015 (0.005; 0.033)	0.015 (0.005; 0.033)	0.015 (0.005; 0.033)	0.015 (0.005; 0.033)	0.015 (0.005; 0.033)	0.015 (0.005; 0.032)	0.015 (0.005; 0.032)	0.015 (0.005; 0.032)	0.015 (0.005; 0.032)
Ethiopia	0.013 (0.004; 0.027)	0.013 (0.004; 0.027)	0.013 (0.004; 0.028)	0.013 (0.004; 0.028)	0.013 (0.004; 0.028)	0.013 (0.004; 0.028)	0.013 (0.004; 0.028)	0.013 (0.004; 0.028)	0.013 (0.004; 0.029)	0.013 (0.004; 0.029)	0.013 (0.004; 0.029)	0.014 (0.004; 0.029)	0.014 (0.004; 0.029)	0.014 (0.004; 0.030)	0.014 (0.004; 0.030)	0.014 (0.004; 0.030)
Fiji	0.005 (0.002; 0.009)	0.005 (0.002; 0.009)	0.005 (0.002; 0.009)	0.005 (0.002; 0.009)	0.005 (0.002; 0.009)	0.005 (0.002; 0.009)	0.004 (0.002; 0.009)	0.004 (0.002; 0.009)	0.004 (0.002; 0.009)	0.004 (0.002; 0.009)	0.004 (0.002; 0.009)	0.004 (0.002; 0.009)	0.004 (0.002; 0.009)	0.004 (0.002; 0.009)	0.004 (0.002; 0.009)	0.004 (0.002; 0.009)
Gabon	0.013 (0.004; 0.028)	0.013 (0.004; 0.028)	0.013 (0.004; 0.028)	0.013 (0.004; 0.029)	0.013 (0.004; 0.029)	0.013 (0.004; 0.029)	0.014 (0.004; 0.029)	0.014 (0.004; 0.029)	0.014 (0.004; 0.029)	0.014 (0.004; 0.030)	0.014 (0.004; 0.030)	0.014 (0.004; 0.030)	0.014 (0.004; 0.030)	0.014 (0.005; 0.030)	0.014 (0.005; 0.031)	0.014 (0.005; 0.031)
Gambia, The	0.016 (0.005; 0.034)	0.016 (0.005; 0.034)	0.016 (0.005; 0.034)	0.016 (0.005; 0.034)	0.016 (0.005; 0.033)	0.015 (0.005; 0.033)	0.015 (0.005; 0.033)	0.015 (0.005; 0.033)	0.015 (0.005; 0.033)	0.015 (0.005; 0.033)	0.015 (0.005; 0.033)	0.015 (0.005; 0.033)	0.015 (0.005; 0.032)	0.015 (0.005; 0.032)	0.015 (0.005; 0.032)	0.015 (0.005; 0.032)
Georgia	0.006 (0.002; 0.012)	0.006 (0.002; 0.012)	0.006 (0.002; 0.012)	0.006 (0.002; 0.012)	0.006 (0.002; 0.012)	0.005 (0.002; 0.012)	0.005 (0.002; 0.012)	0.005 (0.002; 0.012)	0.005 (0.002; 0.012)	0.005 (0.002; 0.012)	0.005 (0.002; 0.012)	0.005 (0.002; 0.012)	0.005 (0.002; 0.012)	0.005 (0.002; 0.012)	0.005 (0.002; 0.012)	0.005 (0.002; 0.012)
Ghana	0.017 (0.005; 0.037)	0.017 (0.005; 0.036)	0.017 (0.005; 0.036)	0.017 (0.005; 0.036)	0.017 (0.005; 0.036)	0.017 (0.005; 0.036)	0.017 (0.005; 0.036)	0.017 (0.005; 0.036)	0.016 (0.005; 0.036)	0.016 (0.005; 0.035)	0.016 (0.005; 0.035)	0.016 (0.005; 0.035)	0.016 (0.005; 0.035)	0.016 (0.005; 0.035)	0.016 (0.005; 0.035)	0.016 (0.005; 0.035)

Guatemala	0.011 (0.004; 0.023)	0.011 (0.004; 0.023)	0.011 (0.004; 0.023)	0.011 (0.004; 0.023)	0.011 (0.004; 0.023)	0.011 (0.004; 0.023)	0.011 (0.004; 0.022)	0.011 (0.004; 0.022)	0.011 (0.004; 0.022)	0.011 (0.004; 0.022)	0.011 (0.004; 0.022)	0.011 (0.004; 0.022)	0.011 (0.004; 0.022)	0.011 (0.004; 0.022)	0.011 (0.004; 0.022)	0.011 (0.004; 0.022)
Guinea	0.009 (0.003; 0.019)	0.009 (0.003; 0.019)	0.009 (0.003; 0.019)	0.009 (0.003; 0.019)	0.009 (0.003; 0.019)	0.009 (0.003; 0.019)	0.009 (0.003; 0.020)	0.009 (0.003; 0.020)	0.009 (0.003; 0.020)	0.009 (0.003; 0.020)	0.009 (0.003; 0.020)	0.009 (0.003; 0.020)	0.009 (0.003; 0.020)	0.009 (0.003; 0.020)	0.010 (0.003; 0.021)	0.010 (0.003; 0.021)
Guinea-Bissau	0.015 (0.005; 0.031)	0.015 (0.005; 0.031)	0.015 (0.005; 0.032)	0.015 (0.005; 0.032)	0.015 (0.005; 0.032)	0.015 (0.005; 0.032)	0.015 (0.005; 0.032)	0.015 (0.005; 0.033)	0.015 (0.005; 0.033)	0.015 (0.005; 0.033)	0.015 (0.005; 0.033)	0.015 (0.005; 0.033)	0.015 (0.005; 0.032)	0.015 (0.005; 0.032)	0.015 (0.005; 0.032)	0.015 (0.005; 0.032)
Haiti	0.007 (0.002; 0.014)	0.007 (0.002; 0.014)	0.007 (0.002; 0.014)	0.007 (0.002; 0.014)	0.007 (0.002; 0.014)	0.007 (0.002; 0.014)	0.007 (0.002; 0.014)	0.007 (0.003; 0.014)	0.007 (0.003; 0.014)	0.007 (0.003; 0.015)	0.007 (0.003; 0.015)	0.007 (0.003; 0.015)	0.007 (0.003; 0.015)	0.007 (0.003; 0.015)	0.007 (0.003; 0.015)	0.008 (0.003; 0.015)
Honduras	0.011 (0.004; 0.023)	0.011 (0.004; 0.023)	0.011 (0.004; 0.023)	0.011 (0.004; 0.023)	0.011 (0.004; 0.023)	0.011 (0.004; 0.023)	0.011 (0.004; 0.022)	0.011 (0.004; 0.022)	0.011 (0.004; 0.022)	0.011 (0.004; 0.022)	0.011 (0.004; 0.022)	0.011 (0.004; 0.022)	0.011 (0.004; 0.022)	0.011 (0.004; 0.022)	0.011 (0.004; 0.022)	0.011 (0.004; 0.022)
India	0.005 (0.002; 0.012)	0.005 (0.002; 0.012)	0.005 (0.002; 0.012)	0.005 (0.002; 0.012)	0.005 (0.002; 0.012)	0.005 (0.002; 0.012)	0.005 (0.002; 0.012)	0.005 (0.002; 0.012)	0.005 (0.002; 0.012)	0.005 (0.002; 0.012)	0.005 (0.002; 0.012)	0.005 (0.002; 0.012)	0.005 (0.002; 0.012)	0.005 (0.002; 0.012)	0.005 (0.002; 0.012)	0.005 (0.002; 0.012)
Indonesia	0.005 (0.002; 0.012)	0.005 (0.002; 0.012)	0.005 (0.002; 0.012)	0.005 (0.002; 0.012)	0.005 (0.002; 0.012)	0.005 (0.002; 0.012)	0.005 (0.002; 0.012)	0.005 (0.002; 0.012)	0.005 (0.002; 0.012)	0.005 (0.002; 0.012)	0.005 (0.002; 0.012)	0.005 (0.002; 0.012)	0.005 (0.002; 0.012)	0.005 (0.002; 0.011)	0.005 (0.002; 0.011)	0.005 (0.002; 0.011)
Iraq	0.008 (0.003; 0.019)	0.009 (0.003; 0.020)	0.009 (0.003; 0.019)	0.008 (0.003; 0.019)	0.008 (0.003; 0.019)	0.008 (0.003; 0.019)	0.008 (0.003; 0.019)	0.008 (0.003; 0.019)	0.008 (0.003; 0.019)	0.008 (0.003; 0.019)	0.008 (0.003; 0.019)	0.008 (0.003; 0.019)	0.008 (0.003; 0.019)	0.008 (0.003; 0.018)	0.008 (0.003; 0.018)	0.008 (0.003; 0.018)
Jamaica	0.012 (0.004; 0.025)	0.012 (0.004; 0.024)	0.012 (0.004; 0.024)	0.012 (0.004; 0.024)	0.012 (0.004; 0.024)	0.012 (0.004; 0.024)	0.012 (0.004; 0.024)	0.012 (0.004; 0.024)	0.012 (0.004; 0.024)	0.012 (0.004; 0.024)	0.012 (0.004; 0.024)	0.012 (0.004; 0.023)	0.012 (0.004; 0.023)	0.012 (0.004; 0.023)	0.012 (0.004; 0.023)	0.011 (0.004; 0.023)
Jordan	0.009 (0.003; 0.020)	0.009 (0.003; 0.020)	0.009 (0.003; 0.019)	0.008 (0.003; 0.019)	0.008 (0.003; 0.019)	0.008 (0.003; 0.019)	0.008 (0.003; 0.019)	0.008 (0.003; 0.019)	0.008 (0.003; 0.019)	0.008 (0.003; 0.019)	0.008 (0.003; 0.019)	0.008 (0.003; 0.019)	0.008 (0.003; 0.019)	0.008 (0.003; 0.018)	0.008 (0.003; 0.018)	0.008 (0.003; 0.018)
Kazakhstan	0.006 (0.002; 0.013)	0.006 (0.002; 0.013)	0.006 (0.002; 0.013)	0.006 (0.002; 0.013)	0.006 (0.002; 0.012)	0.006 (0.002; 0.012)	0.006 (0.002; 0.012)	0.006 (0.002; 0.012)	0.006 (0.002; 0.012)	0.006 (0.002; 0.012)	0.006 (0.002; 0.012)	0.006 (0.002; 0.012)	0.006 (0.002; 0.012)	0.005 (0.002; 0.012)	0.005 (0.002; 0.012)	0.005 (0.002; 0.012)
Kenya	0.016 (0.005; 0.034)	0.016 (0.005; 0.034)	0.016 (0.005; 0.034)	0.016 (0.005; 0.034)	0.016 (0.005; 0.034)	0.016 (0.005; 0.034)	0.016 (0.005; 0.034)	0.016 (0.005; 0.033)	0.015 (0.005; 0.033)	0.015 (0.005; 0.033)	0.015 (0.005; 0.033)	0.015 (0.005; 0.033)	0.015 (0.005; 0.033)	0.015 (0.005; 0.033)	0.015 (0.005; 0.033)	0.015 (0.005; 0.032)

Kiribati	0.005 (0.002; 0.009)	0.004 (0.002; 0.009)	0.004 (0.002; 0.009)	0.004 (0.002; 0.009)	0.004 (0.002; 0.009)	0.004 (0.002; 0.009)	0.004 (0.002; 0.009)	0.004 (0.002; 0.009)	0.004 (0.002; 0.009)	0.004 (0.002; 0.009)	0.004 (0.002; 0.009)	0.004 (0.002; 0.009)	0.004 (0.002; 0.009)	0.004 (0.002; 0.009)	0.004 (0.002; 0.009)	0.004 (0.002; 0.009)
Kyrgyz Republic	0.006 (0.002; 0.012)	0.006 (0.002; 0.012)	0.006 (0.002; 0.012)	0.006 (0.002; 0.012)	0.006 (0.002; 0.012)	0.006 (0.002; 0.012)	0.006 (0.002; 0.012)	0.006 (0.002; 0.012)	0.005 (0.002; 0.012)	0.005 (0.002; 0.012)	0.005 (0.002; 0.012)	0.005 (0.002; 0.012)	0.005 (0.002; 0.012)	0.005 (0.002; 0.012)	0.005 (0.002; 0.012)	0.005 (0.002; 0.012)
Lao PDR	0.004 (0.001; 0.008)	0.004 (0.001; 0.008)	0.004 (0.001; 0.008)	0.004 (0.001; 0.008)	0.004 (0.001; 0.008)	0.004 (0.001; 0.008)	0.004 (0.001; 0.008)	0.004 (0.001; 0.008)	0.004 (0.001; 0.008)	0.004 (0.001; 0.008)	0.004 (0.001; 0.008)	0.004 (0.001; 0.008)	0.004 (0.001; 0.008)	0.004 (0.001; 0.008)	0.004 (0.001; 0.008)	0.004 (0.001; 0.008)
Lesotho	0.016 (0.005; 0.034)	0.016 (0.005; 0.034)	0.016 (0.005; 0.034)	0.016 (0.005; 0.034)	0.016 (0.005; 0.033)	0.015 (0.005; 0.033)	0.015 (0.005; 0.033)	0.015 (0.005; 0.033)	0.015 (0.005; 0.033)	0.015 (0.005; 0.033)	0.015 (0.005; 0.033)	0.015 (0.005; 0.033)	0.015 (0.005; 0.032)	0.015 (0.005; 0.032)	0.015 (0.005; 0.032)	0.015 (0.005; 0.032)
Liberia	0.013 (0.004; 0.028)	0.013 (0.004; 0.028)	0.013 (0.004; 0.028)	0.013 (0.004; 0.029)	0.013 (0.004; 0.029)	0.013 (0.004; 0.029)	0.014 (0.004; 0.029)	0.014 (0.004; 0.029)	0.014 (0.004; 0.029)	0.014 (0.004; 0.030)	0.014 (0.004; 0.030)	0.014 (0.004; 0.030)	0.014 (0.004; 0.030)	0.014 (0.005; 0.030)	0.014 (0.005; 0.031)	0.014 (0.005; 0.031)
Madagascar	0.013 (0.004; 0.028)	0.013 (0.004; 0.028)	0.013 (0.004; 0.028)	0.013 (0.004; 0.029)	0.013 (0.004; 0.029)	0.013 (0.004; 0.029)	0.014 (0.004; 0.029)	0.014 (0.004; 0.029)	0.014 (0.004; 0.029)	0.014 (0.004; 0.030)	0.014 (0.004; 0.030)	0.014 (0.004; 0.030)	0.014 (0.004; 0.030)	0.014 (0.005; 0.030)	0.014 (0.005; 0.031)	0.014 (0.005; 0.031)
Malawi	0.017 (0.005; 0.036)	0.017 (0.005; 0.036)	0.017 (0.005; 0.036)	0.016 (0.005; 0.035)	0.016 (0.005; 0.035)	0.016 (0.005; 0.035)	0.016 (0.005; 0.035)	0.016 (0.005; 0.035)	0.016 (0.005; 0.035)	0.016 (0.005; 0.035)	0.016 (0.005; 0.035)	0.016 (0.005; 0.034)	0.016 (0.005; 0.034)	0.016 (0.005; 0.034)	0.016 (0.005; 0.034)	0.016 (0.005; 0.034)
Malaysia	0.005 (0.002; 0.009)	0.005 (0.002; 0.009)	0.005 (0.002; 0.009)	0.004 (0.002; 0.009)	0.004 (0.002; 0.009)	0.004 (0.002; 0.009)	0.004 (0.002; 0.009)	0.004 (0.002; 0.009)	0.004 (0.002; 0.009)	0.004 (0.002; 0.009)	0.004 (0.002; 0.009)	0.004 (0.002; 0.009)	0.004 (0.002; 0.009)	0.004 (0.002; 0.009)	0.004 (0.002; 0.009)	0.004 (0.002; 0.009)
Mali	0.014 (0.005; 0.031)	0.014 (0.005; 0.031)	0.014 (0.005; 0.031)	0.015 (0.005; 0.031)	0.015 (0.005; 0.032)	0.015 (0.005; 0.032)	0.015 (0.005; 0.032)	0.015 (0.005; 0.032)	0.015 (0.005; 0.032)	0.015 (0.005; 0.033)	0.015 (0.005; 0.033)	0.015 (0.005; 0.033)	0.015 (0.005; 0.032)	0.015 (0.005; 0.032)	0.015 (0.005; 0.032)	0.015 (0.005; 0.032)
Mauritania	0.015 (0.005; 0.032)	0.015 (0.005; 0.033)	0.015 (0.005; 0.033)	0.015 (0.005; 0.033)	0.015 (0.005; 0.033)	0.015 (0.005; 0.033)	0.015 (0.005; 0.033)	0.015 (0.005; 0.033)	0.015 (0.005; 0.033)	0.015 (0.005; 0.033)	0.015 (0.005; 0.033)	0.015 (0.005; 0.033)	0.015 (0.005; 0.032)	0.015 (0.005; 0.032)	0.015 (0.005; 0.032)	0.015 (0.005; 0.032)
Mexico	0.011 (0.004; 0.022)	0.011 (0.004; 0.022)	0.011 (0.004; 0.023)	0.011 (0.004; 0.023)	0.011 (0.004; 0.023)	0.011 (0.004; 0.023)	0.011 (0.004; 0.022)	0.011 (0.004; 0.022)	0.011 (0.004; 0.022)	0.011 (0.004; 0.022)	0.011 (0.004; 0.022)	0.011 (0.004; 0.022)	0.011 (0.004; 0.022)	0.011 (0.004; 0.022)	0.011 (0.004; 0.022)	0.011 (0.004; 0.022)
Mongolia	0.005 (0.002; 0.009)	0.005 (0.002; 0.009)	0.005 (0.002; 0.009)	0.004 (0.002; 0.009)	0.004 (0.002; 0.009)	0.004 (0.002; 0.009)	0.004 (0.002; 0.009)	0.004 (0.002; 0.009)	0.004 (0.002; 0.009)	0.004 (0.002; 0.009)	0.004 (0.002; 0.009)	0.004 (0.002; 0.009)	0.004 (0.002; 0.009)	0.004 (0.002; 0.009)	0.004 (0.002; 0.009)	0.004 (0.002; 0.009)

Morocco	0.009 (0.003; 0.022)	0.009 (0.003; 0.021)	0.009 (0.003; 0.021)	0.009 (0.003; 0.021)	0.009 (0.003; 0.021)	0.009 (0.003; 0.021)	0.009 (0.003; 0.021)	0.009 (0.003; 0.021)	0.009 (0.003; 0.021)	0.009 (0.003; 0.021)	0.009 (0.003; 0.021)	0.009 (0.003; 0.020)	0.009 (0.003; 0.020)	0.009 (0.003; 0.020)	0.009 (0.003; 0.020)	0.009 (0.003; 0.020)
Mozambique	0.016 (0.005; 0.034)	0.016 (0.005; 0.034)	0.016 (0.005; 0.034)	0.016 (0.005; 0.034)	0.016 (0.005; 0.033)	0.015 (0.005; 0.033)	0.015 (0.005; 0.033)	0.015 (0.005; 0.033)	0.015 (0.005; 0.033)	0.015 (0.005; 0.033)	0.015 (0.005; 0.033)	0.015 (0.005; 0.033)	0.015 (0.005; 0.032)	0.015 (0.005; 0.032)	0.015 (0.005; 0.032)	0.015 (0.005; 0.032)
Myanmar	0.005 (0.002; 0.012)	0.005 (0.002; 0.012)	0.005 (0.002; 0.012)	0.005 (0.002; 0.012)	0.005 (0.002; 0.012)	0.005 (0.002; 0.012)	0.005 (0.002; 0.012)	0.005 (0.002; 0.012)	0.005 (0.002; 0.012)	0.005 (0.002; 0.012)	0.005 (0.002; 0.012)	0.005 (0.002; 0.012)	0.005 (0.002; 0.012)	0.005 (0.002; 0.011)	0.005 (0.002; 0.011)	0.005 (0.002; 0.011)
Namibia	0.016 (0.005; 0.034)	0.016 (0.005; 0.034)	0.016 (0.005; 0.034)	0.016 (0.005; 0.034)	0.016 (0.005; 0.033)	0.015 (0.005; 0.033)	0.015 (0.005; 0.033)	0.015 (0.005; 0.033)	0.015 (0.005; 0.033)	0.015 (0.005; 0.033)	0.015 (0.005; 0.033)	0.015 (0.005; 0.033)	0.015 (0.005; 0.032)	0.015 (0.005; 0.032)	0.015 (0.005; 0.032)	0.015 (0.005; 0.032)
Nepal	0.006 (0.002; 0.012)	0.006 (0.002; 0.012)	0.006 (0.002; 0.012)	0.006 (0.002; 0.012)	0.005 (0.002; 0.012)	0.005 (0.002; 0.012)	0.005 (0.002; 0.012)	0.005 (0.002; 0.012)	0.005 (0.002; 0.012)	0.005 (0.002; 0.012)	0.005 (0.002; 0.012)	0.005 (0.002; 0.012)	0.005 (0.002; 0.012)	0.005 (0.002; 0.012)	0.005 (0.002; 0.012)	0.005 (0.002; 0.012)
Nicaragua	0.012 (0.004; 0.025)	0.012 (0.004; 0.025)	0.012 (0.004; 0.025)	0.012 (0.004; 0.025)	0.012 (0.004; 0.025)	0.012 (0.004; 0.025)	0.012 (0.004; 0.024)	0.012 (0.004; 0.024)	0.012 (0.004; 0.024)	0.012 (0.004; 0.024)	0.012 (0.004; 0.024)	0.012 (0.004; 0.024)	0.012 (0.004; 0.024)	0.012 (0.004; 0.024)	0.012 (0.004; 0.024)	0.012 (0.004; 0.024)
Niger	0.015 (0.005; 0.032)	0.015 (0.005; 0.033)	0.015 (0.005; 0.033)	0.015 (0.005; 0.033)	0.015 (0.005; 0.033)	0.015 (0.005; 0.033)	0.015 (0.005; 0.033)	0.015 (0.005; 0.033)	0.015 (0.005; 0.033)	0.015 (0.005; 0.033)	0.015 (0.005; 0.033)	0.015 (0.005; 0.033)	0.015 (0.005; 0.032)	0.015 (0.005; 0.032)	0.015 (0.005; 0.032)	0.015 (0.005; 0.032)
Nigeria	0.011 (0.003; 0.023)	0.011 (0.003; 0.023)	0.011 (0.003; 0.023)	0.011 (0.003; 0.023)	0.011 (0.003; 0.023)	0.011 (0.004; 0.024)	0.011 (0.004; 0.024)	0.011 (0.004; 0.024)	0.011 (0.004; 0.024)	0.011 (0.004; 0.024)	0.011 (0.004; 0.024)	0.011 (0.004; 0.024)	0.011 (0.004; 0.025)	0.011 (0.004; 0.025)	0.012 (0.004; 0.025)	0.012 (0.004; 0.025)
Pakistan	0.008 (0.003; 0.019)	0.009 (0.003; 0.020)	0.009 (0.003; 0.019)	0.008 (0.003; 0.019)	0.008 (0.003; 0.019)	0.008 (0.003; 0.019)	0.008 (0.003; 0.019)	0.008 (0.003; 0.019)	0.008 (0.003; 0.019)	0.008 (0.003; 0.019)	0.008 (0.003; 0.019)	0.008 (0.003; 0.019)	0.008 (0.003; 0.019)	0.008 (0.003; 0.018)	0.008 (0.003; 0.018)	0.008 (0.003; 0.018)
Papua New Guinea	0.002 (0.001; 0.004)	0.002 (0.001; 0.004)	0.002 (0.001; 0.004)	0.002 (0.001; 0.004)	0.002 (0.001; 0.004)	0.002 (0.001; 0.004)	0.002 (0.001; 0.004)	0.002 (0.001; 0.004)	0.002 (0.001; 0.004)	0.002 (0.001; 0.004)	0.002 (0.001; 0.004)	0.002 (0.001; 0.004)	0.002 (0.001; 0.004)	0.002 (0.001; 0.004)	0.002 (0.001; 0.004)	0.002 (0.001; 0.004)
Paraguay	0.011 (0.004; 0.023)	0.011 (0.004; 0.023)	0.011 (0.004; 0.023)	0.011 (0.004; 0.023)	0.011 (0.004; 0.023)	0.011 (0.004; 0.023)	0.011 (0.004; 0.022)	0.011 (0.004; 0.022)	0.011 (0.004; 0.022)	0.011 (0.004; 0.022)	0.011 (0.004; 0.022)	0.011 (0.004; 0.022)	0.011 (0.004; 0.022)	0.011 (0.004; 0.022)	0.011 (0.004; 0.022)	0.011 (0.004; 0.022)
Peru	0.011 (0.004; 0.023)	0.011 (0.004; 0.023)	0.011 (0.004; 0.023)	0.011 (0.004; 0.023)	0.011 (0.004; 0.023)	0.011 (0.004; 0.023)	0.011 (0.004; 0.022)	0.011 (0.004; 0.022)	0.011 (0.004; 0.022)	0.011 (0.004; 0.022)	0.011 (0.004; 0.022)	0.011 (0.004; 0.022)	0.011 (0.004; 0.022)	0.011 (0.004; 0.022)	0.011 (0.004; 0.022)	0.011 (0.004; 0.022)

Philippines	0.004 (0.001; 0.008)	0.004 (0.001; 0.008)	0.004 (0.001; 0.008)	0.004 (0.001; 0.008)	0.004 (0.001; 0.008)	0.004 (0.001; 0.008)	0.004 (0.001; 0.008)	0.004 (0.001; 0.008)	0.004 (0.001; 0.008)	0.004 (0.001; 0.008)	0.004 (0.001; 0.008)	0.004 (0.001; 0.008)	0.004 (0.001; 0.008)	0.004 (0.001; 0.008)	0.004 (0.001; 0.008)	0.004 (0.001; 0.008)
Rwanda	0.017 (0.005; 0.037)	0.017 (0.005; 0.037)	0.017 (0.005; 0.037)	0.017 (0.005; 0.037)	0.017 (0.005; 0.036)	0.017 (0.005; 0.036)	0.017 (0.005; 0.036)	0.017 (0.005; 0.036)	0.017 (0.005; 0.036)	0.017 (0.005; 0.036)	0.017 (0.005; 0.036)	0.016 (0.005; 0.035)	0.016 (0.005; 0.035)	0.016 (0.005; 0.035)	0.016 (0.005; 0.035)	0.016 (0.005; 0.035)
Senegal	0.017 (0.005; 0.036)	0.017 (0.005; 0.036)	0.017 (0.005; 0.036)	0.016 (0.005; 0.035)	0.016 (0.005; 0.035)	0.016 (0.005; 0.035)	0.016 (0.005; 0.035)	0.016 (0.005; 0.035)	0.016 (0.005; 0.035)	0.016 (0.005; 0.035)	0.016 (0.005; 0.035)	0.016 (0.005; 0.034)	0.016 (0.005; 0.034)	0.016 (0.005; 0.034)	0.016 (0.005; 0.034)	0.016 (0.005; 0.034)
Sierra Leone	0.017 (0.005; 0.036)	0.017 (0.005; 0.036)	0.017 (0.005; 0.036)	0.016 (0.005; 0.035)	0.016 (0.005; 0.035)	0.016 (0.005; 0.035)	0.016 (0.005; 0.035)	0.016 (0.005; 0.035)	0.016 (0.005; 0.035)	0.016 (0.005; 0.035)	0.016 (0.005; 0.035)	0.016 (0.005; 0.034)	0.016 (0.005; 0.034)	0.016 (0.005; 0.034)	0.016 (0.005; 0.034)	0.016 (0.005; 0.034)
Solomon Islands	0.004 (0.002; 0.009)	0.004 (0.002; 0.009)	0.004 (0.002; 0.009)	0.004 (0.002; 0.009)	0.004 (0.002; 0.009)	0.004 (0.002; 0.009)	0.004 (0.002; 0.009)	0.004 (0.002; 0.009)	0.004 (0.001; 0.009)	0.004 (0.001; 0.009)	0.004 (0.001; 0.009)	0.004 (0.001; 0.009)	0.004 (0.001; 0.009)	0.004 (0.001; 0.009)	0.004 (0.001; 0.009)	0.004 (0.001; 0.009)
Somalia	0.004 (0.001; 0.010)	0.004 (0.001; 0.010)	0.004 (0.001; 0.010)	0.004 (0.001; 0.010)	0.004 (0.001; 0.010)	0.004 (0.001; 0.010)	0.004 (0.001; 0.010)	0.004 (0.001; 0.010)	0.004 (0.001; 0.010)	0.004 (0.001; 0.010)	0.005 (0.001; 0.010)	0.005 (0.001; 0.010)	0.005 (0.001; 0.010)	0.005 (0.001; 0.010)	0.005 (0.001; 0.010)	0.005 (0.001; 0.011)
South Africa	0.016 (0.005; 0.034)	0.016 (0.005; 0.034)	0.016 (0.005; 0.034)	0.016 (0.005; 0.034)	0.016 (0.005; 0.033)	0.015 (0.005; 0.033)	0.015 (0.005; 0.033)	0.015 (0.005; 0.033)	0.015 (0.005; 0.033)	0.015 (0.005; 0.033)	0.015 (0.005; 0.033)	0.015 (0.005; 0.033)	0.015 (0.005; 0.032)	0.015 (0.005; 0.032)	0.015 (0.005; 0.032)	0.015 (0.005; 0.032)
Sri Lanka	0.006 (0.002; 0.013)	0.006 (0.002; 0.013)	0.006 (0.002; 0.013)	0.006 (0.002; 0.013)	0.006 (0.002; 0.013)	0.006 (0.002; 0.013)	0.006 (0.002; 0.013)	0.006 (0.002; 0.013)	0.006 (0.002; 0.013)	0.006 (0.002; 0.013)	0.006 (0.002; 0.013)	0.006 (0.002; 0.013)	0.006 (0.002; 0.013)	0.006 (0.002; 0.013)	0.006 (0.002; 0.013)	0.006 (0.002; 0.013)
Sudan	0.009 (0.003; 0.020)	0.009 (0.003; 0.020)	0.009 (0.003; 0.020)	0.009 (0.003; 0.020)	0.009 (0.003; 0.020)	0.009 (0.003; 0.020)	0.009 (0.003; 0.020)	0.009 (0.003; 0.020)	0.009 (0.003; 0.019)	0.009 (0.003; 0.019)	0.009 (0.003; 0.019)	0.008 (0.003; 0.019)	0.008 (0.003; 0.019)	0.008 (0.003; 0.019)	0.008 (0.003; 0.019)	0.008 (0.003; 0.019)
Suriname	0.010 (0.004; 0.021)	0.010 (0.004; 0.021)	0.010 (0.004; 0.021)	0.011 (0.004; 0.021)	0.011 (0.004; 0.021)	0.011 (0.004; 0.022)	0.011 (0.004; 0.022)	0.011 (0.004; 0.022)	0.011 (0.004; 0.022)	0.011 (0.004; 0.022)	0.011 (0.004; 0.022)	0.011 (0.004; 0.022)	0.011 (0.004; 0.022)	0.011 (0.004; 0.022)	0.011 (0.004; 0.022)	0.011 (0.004; 0.022)
Tajikistan	0.006 (0.002; 0.013)	0.006 (0.002; 0.013)	0.006 (0.002; 0.013)	0.006 (0.002; 0.013)	0.006 (0.002; 0.012)	0.006 (0.002; 0.012)	0.006 (0.002; 0.012)	0.006 (0.002; 0.012)	0.006 (0.002; 0.012)	0.006 (0.002; 0.012)	0.006 (0.002; 0.012)	0.006 (0.002; 0.012)	0.006 (0.002; 0.012)	0.005 (0.002; 0.012)	0.005 (0.002; 0.012)	0.005 (0.002; 0.012)
Tanzania	0.016 (0.005; 0.034)	0.016 (0.005; 0.034)	0.016 (0.005; 0.034)	0.016 (0.005; 0.034)	0.016 (0.005; 0.033)	0.015 (0.005; 0.033)	0.015 (0.005; 0.033)	0.015 (0.005; 0.033)	0.015 (0.005; 0.033)	0.015 (0.005; 0.033)	0.015 (0.005; 0.033)	0.015 (0.005; 0.033)	0.015 (0.005; 0.032)	0.015 (0.005; 0.032)	0.015 (0.005; 0.032)	0.015 (0.005; 0.032)

Thailand	0.006 (0.002; 0.013)	0.006 (0.002; 0.013)	0.006 (0.002; 0.013)	0.006 (0.002; 0.013)	0.006 (0.002; 0.013)	0.006 (0.002; 0.013)	0.006 (0.002; 0.013)	0.006 (0.002; 0.013)	0.006 (0.002; 0.013)	0.006 (0.002; 0.013)	0.006 (0.002; 0.012)	0.006 (0.002; 0.012)	0.006 (0.002; 0.012)	0.006 (0.002; 0.012)	0.006 (0.002; 0.012)	0.006 (0.002; 0.012)
Timor-Leste	0.005 (0.002; 0.012)	0.005 (0.002; 0.012)	0.005 (0.002; 0.012)	0.005 (0.002; 0.012)	0.005 (0.002; 0.012)	0.005 (0.002; 0.012)	0.005 (0.002; 0.012)	0.005 (0.002; 0.012)	0.005 (0.002; 0.012)	0.005 (0.002; 0.012)	0.005 (0.002; 0.012)	0.005 (0.002; 0.012)	0.005 (0.002; 0.012)	0.005 (0.002; 0.011)	0.005 (0.002; 0.011)	0.005 (0.002; 0.011)
Togo	0.016 (0.005; 0.034)	0.016 (0.005; 0.034)	0.016 (0.005; 0.034)	0.016 (0.005; 0.034)	0.016 (0.005; 0.033)	0.015 (0.005; 0.033)	0.015 (0.005; 0.033)	0.015 (0.005; 0.033)	0.015 (0.005; 0.033)	0.015 (0.005; 0.033)	0.015 (0.005; 0.033)	0.015 (0.005; 0.033)	0.015 (0.005; 0.032)	0.015 (0.005; 0.032)	0.015 (0.005; 0.032)	0.015 (0.005; 0.032)
Tunisia	0.009 (0.003; 0.020)	0.009 (0.003; 0.020)	0.009 (0.003; 0.020)	0.009 (0.003; 0.020)	0.009 (0.003; 0.020)	0.009 (0.003; 0.020)	0.009 (0.003; 0.019)	0.009 (0.003; 0.019)	0.009 (0.003; 0.019)	0.008 (0.003; 0.019)	0.008 (0.003; 0.019)	0.008 (0.003; 0.019)	0.008 (0.003; 0.019)	0.008 (0.003; 0.019)	0.008 (0.003; 0.019)	0.008 (0.003; 0.019)
Turkey	0.006 (0.002; 0.013)	0.006 (0.002; 0.013)	0.006 (0.002; 0.013)	0.006 (0.002; 0.013)	0.006 (0.002; 0.013)	0.006 (0.002; 0.013)	0.006 (0.002; 0.013)	0.006 (0.002; 0.013)	0.006 (0.002; 0.013)	0.006 (0.002; 0.013)	0.006 (0.002; 0.012)	0.006 (0.002; 0.012)	0.006 (0.002; 0.012)	0.006 (0.002; 0.012)	0.006 (0.002; 0.012)	0.006 (0.002; 0.012)
Uganda	0.016 (0.005; 0.035)	0.016 (0.005; 0.035)	0.016 (0.005; 0.035)	0.016 (0.005; 0.035)	0.016 (0.005; 0.035)	0.016 (0.005; 0.034)	0.016 (0.005; 0.034)	0.016 (0.005; 0.034)	0.016 (0.005; 0.034)	0.016 (0.005; 0.034)	0.016 (0.005; 0.034)	0.016 (0.005; 0.034)	0.016 (0.005; 0.034)	0.016 (0.005; 0.033)	0.015 (0.005; 0.033)	0.015 (0.005; 0.033)
Ukraine	0.005 (0.002; 0.011)	0.005 (0.002; 0.011)	0.005 (0.002; 0.011)	0.005 (0.002; 0.011)	0.005 (0.002; 0.011)	0.005 (0.002; 0.011)	0.005 (0.002; 0.011)	0.005 (0.002; 0.011)	0.005 (0.002; 0.011)	0.005 (0.002; 0.011)	0.005 (0.002; 0.011)	0.005 (0.002; 0.011)	0.005 (0.002; 0.011)	0.005 (0.002; 0.011)	0.005 (0.002; 0.011)	0.005 (0.002; 0.011)
Uzbekistan	0.006 (0.002; 0.012)	0.006 (0.002; 0.012)	0.006 (0.002; 0.012)	0.006 (0.002; 0.012)	0.006 (0.002; 0.012)	0.006 (0.002; 0.012)	0.006 (0.002; 0.012)	0.006 (0.002; 0.012)	0.006 (0.002; 0.012)	0.006 (0.002; 0.012)	0.006 (0.002; 0.012)	0.005 (0.002; 0.012)	0.005 (0.002; 0.012)	0.005 (0.002; 0.012)	0.005 (0.002; 0.012)	0.005 (0.002; 0.012)
Vanuatu	0.004 (0.001; 0.009)	0.004 (0.001; 0.009)	0.004 (0.001; 0.009)	0.004 (0.001; 0.008)	0.004 (0.001; 0.008)	0.004 (0.001; 0.008)	0.004 (0.001; 0.008)	0.004 (0.001; 0.008)	0.004 (0.001; 0.008)	0.004 (0.001; 0.008)	0.004 (0.001; 0.008)	0.004 (0.001; 0.008)	0.004 (0.001; 0.008)	0.004 (0.001; 0.008)	0.004 (0.001; 0.008)	0.004 (0.001; 0.008)
Vietnam	0.004 (0.001; 0.009)	0.004 (0.001; 0.009)	0.004 (0.001; 0.009)	0.004 (0.001; 0.008)	0.004 (0.001; 0.008)	0.004 (0.001; 0.008)	0.004 (0.001; 0.008)	0.004 (0.001; 0.008)	0.004 (0.001; 0.008)	0.004 (0.001; 0.008)	0.004 (0.001; 0.008)	0.004 (0.001; 0.008)	0.004 (0.001; 0.008)	0.004 (0.001; 0.008)	0.004 (0.001; 0.008)	0.004 (0.001; 0.008)
Yemen, Rep.	0.007 (0.002; 0.017)	0.007 (0.002; 0.017)	0.007 (0.002; 0.017)	0.008 (0.002; 0.017)	0.008 (0.002; 0.017)	0.008 (0.002; 0.017)	0.008 (0.002; 0.017)	0.008 (0.002; 0.018)	0.008 (0.002; 0.018)	0.008 (0.002; 0.018)	0.008 (0.002; 0.018)	0.008 (0.002; 0.018)	0.008 (0.002; 0.018)	0.008 (0.002; 0.018)	0.008 (0.002; 0.018)	0.008 (0.003; 0.018)
Zambia	0.016 (0.005; 0.034)	0.016 (0.005; 0.034)	0.016 (0.005; 0.034)	0.016 (0.005; 0.034)	0.016 (0.005; 0.033)	0.015 (0.005; 0.033)	0.015 (0.005; 0.033)	0.015 (0.005; 0.033)	0.015 (0.005; 0.033)	0.015 (0.005; 0.033)	0.015 (0.005; 0.033)	0.015 (0.005; 0.033)	0.015 (0.005; 0.032)	0.015 (0.005; 0.032)	0.015 (0.005; 0.032)	0.015 (0.005; 0.032)

Zimbabwe	0.016 (0.005; 0.034)	0.016 (0.005; 0.034)	0.016 (0.005; 0.034)	0.016 (0.005; 0.034)	0.016 (0.005; 0.033)	0.015 (0.005; 0.033)	0.015 (0.005; 0.033)	0.015 (0.005; 0.033)	0.015 (0.005; 0.033)	0.015 (0.005; 0.033)	0.015 (0.005; 0.033)	0.015 (0.005; 0.033)	0.015 (0.005; 0.032)	0.015 (0.005; 0.032)	0.015 (0.005; 0.032)	0.015 (0.005; 0.032)

Table S20. Population average z-score shifts assuming that the vaccine was only 10% effective against *Shigella* moderate-to-severe diarrhoea (MSD) and *Shigella*-attributable stunting from MSD episodes. Estimates in parenthesis represent 95% uncertainty estimates from the results of 1000 model simulations.

Country	2025	2026	2027	2028	2029	2030	2031	2032	2033	2034	2035	2036	2037	2038	203[illegible]
Afghanistan	0.001 (0.000; 0.002)	0.001 (0.000; 0.002)	0.001 (0.000; 0.002)	0.001 (0.000; 0.002)	0.001 (0.000; 0.003)	0.001 (0.000; 0.003)	0.001 (0.000; 0.003)	0.001 (0.000; 0.003)	0.001 (0.000; 0.003)	0.001 (0.000; 0.003)	0.001 (0.000; 0.003)	0.001 (0.000; 0.003)	0.001 (0.000; 0.003)	0.001 (0.000; 0.003)	[illegible]
Algeria	0.003 (0.001; 0.005)	0.003 (0.001; 0.005)	0.003 (0.001; 0.005)	0.003 (0.001; 0.005)	0.003 (0.001; 0.005)	0.003 (0.001; 0.005)	0.003 (0.001; 0.005)	0.003 (0.001; 0.005)	0.003 (0.001; 0.005)	0.003 (0.001; 0.005)	0.003 (0.001; 0.005)	0.003 (0.001; 0.005)	0.003 (0.001; 0.005)	0.002 (0.001; 0.005)	[illegible]
Angola	0.002 (0.001; 0.004)	0.002 (0.001; 0.004)	0.002 (0.001; 0.004)	0.002 (0.001; 0.004)	0.002 (0.001; 0.004)	0.002 (0.001; 0.004)	0.002 (0.001; 0.004)	0.002 (0.001; 0.004)	0.002 (0.001; 0.004)	0.002 (0.001; 0.004)	0.002 (0.001; 0.004)	0.002 (0.001; 0.004)	0.002 (0.001; 0.004)	0.002 (0.001; 0.004)	[illegible]
Argentina	0.002 (0.001; 0.003)	0.002 (0.001; 0.004)	0.002 (0.001; 0.004)	0.002 (0.001; 0.004)	0.002 (0.001; 0.004)	0.002 (0.001; 0.003)	0.002 (0.001; 0.003)	0.002 (0.001; 0.003)	0.002 (0.001; 0.003)	0.002 (0.001; 0.003)	0.002 (0.001; 0.003)	0.002 (0.001; 0.003)	0.002 (0.001; 0.003)	0.002 (0.001; 0.003)	[illegible]
Armenia	0.001 (0.000; 0.002)	0.001 (0.000; 0.002)	0.001 (0.000; 0.002)	0.001 (0.000; 0.002)	0.001 (0.000; 0.002)	0.001 (0.000; 0.002)	0.001 (0.000; 0.002)	0.001 (0.000; 0.002)	0.001 (0.000; 0.002)	0.001 (0.000; 0.002)	0.001 (0.000; 0.002)	0.001 (0.000; 0.002)	0.001 (0.000; 0.002)	0.001 (0.000; 0.002)	[illegible]
Azerbaijan	0.001 (0.000; 0.002)	0.001 (0.000; 0.002)	0.001 (0.000; 0.002)	0.001 (0.000; 0.002)	0.001 (0.000; 0.002)	0.001 (0.000; 0.002)	0.001 (0.000; 0.002)	0.001 (0.000; 0.002)	0.001 (0.000; 0.002)	0.001 (0.000; 0.002)	0.001 (0.000; 0.002)	0.001 (0.000; 0.002)	0.001 (0.000; 0.002)	0.001 (0.000; 0.002)	[illegible]
Bangladesh	0.001 (0.000; 0.002)	0.001 (0.000; 0.002)	0.001 (0.000; 0.002)	0.001 (0.000; 0.002)	0.001 (0.000; 0.002)	0.001 (0.000; 0.002)	0.001 (0.000; 0.002)	0.001 (0.000; 0.002)	0.001 (0.000; 0.002)	0.001 (0.000; 0.002)	0.001 (0.000; 0.002)	0.001 (0.000; 0.002)	0.001 (0.000; 0.002)	0.001 (0.000; 0.002)	[illegible]
Belarus	0.001 (0.000; 0.002)	0.001 (0.000; 0.002)	0.001 (0.000; 0.002)	0.001 (0.000; 0.002)	0.001 (0.000; 0.002)	0.001 (0.000; 0.002)	0.001 (0.000; 0.002)	0.001 (0.000; 0.002)	0.001 (0.000; 0.002)	0.001 (0.000; 0.002)	0.001 (0.000; 0.002)	0.001 (0.000; 0.002)	0.001 (0.000; 0.002)	0.001 (0.000; 0.002)	[illegible]

Benin	0.002 (0.001; 0.005)	0.002 (0.001; 0.005)	0.002 (0.001; 0.005)	0.002 (0.001; 0.005)	0.002 (0.001; 0.005)	0.002 (0.001; 0.005)	0.002 (0.001; 0.005)	0.002 (0.001; 0.005)	0.002 (0.001; 0.005)	0.002 (0.001; 0.005)	0.002 (0.001; 0.005)	0.002 (0.001; 0.005)	0.002 (0.001; 0.005)	0.002 (0.001; 0.005)	0.0[illegible] (0.0[illegible]; 0.0[illegible]
Bhutan	0.001 (0.000; 0.002)	0.001 (0.000; 0.002)	0.001 (0.000; 0.002)	0.001 (0.000; 0.002)	0.001 (0.000; 0.002)	0.001 (0.000; 0.002)	0.001 (0.000; 0.002)	0.001 (0.000; 0.002)	0.001 (0.000; 0.002)	0.001 (0.000; 0.002)	0.001 (0.000; 0.002)	0.001 (0.000; 0.002)	0.001 (0.000; 0.002)	0.001 (0.000; 0.002)	0.0[illegible] (0.0[illegible]; 0.0[illegible]
Bolivia	0.002 (0.001; 0.003)	0.002 (0.001; 0.003)	0.002 (0.001; 0.003)	0.002 (0.001; 0.003)	0.002 (0.001; 0.003)	0.002 (0.001; 0.003)	0.002 (0.001; 0.003)	0.002 (0.001; 0.003)	0.002 (0.001; 0.003)	0.002 (0.001; 0.003)	0.002 (0.001; 0.003)	0.002 (0.001; 0.003)	0.002 (0.001; 0.003)	0.002 (0.001; 0.003)	0.0[illegible] (0.0[illegible]; 0.0[illegible]
Botswana	0.003 (0.001; 0.006)	0.003 (0.001; 0.006)	0.003 (0.001; 0.006)	0.003 (0.001; 0.006)	0.003 (0.001; 0.006)	0.003 (0.001; 0.006)	0.003 (0.001; 0.006)	0.003 (0.001; 0.006)	0.003 (0.001; 0.006)	0.003 (0.001; 0.005)	0.003 (0.001; 0.005)	0.003 (0.001; 0.005)	0.003 (0.001; 0.005)	0.003 (0.001; 0.005)	0.0[illegible] (0.0[illegible]; 0.0[illegible]
Brazil	0.002 (0.001; 0.003)	0.002 (0.001; 0.003)	0.002 (0.001; 0.003)	0.002 (0.001; 0.003)	0.002 (0.001; 0.003)	0.002 (0.001; 0.003)	0.002 (0.001; 0.003)	0.002 (0.001; 0.003)	0.002 (0.001; 0.003)	0.002 (0.001; 0.003)	0.002 (0.001; 0.003)	0.002 (0.001; 0.003)	0.002 (0.001; 0.003)	0.002 (0.001; 0.003)	0.0[illegible] (0.0[illegible]; 0.0[illegible]
Bulgaria	0.001 (0.000; 0.002)	0.001 (0.000; 0.002)	0.001 (0.000; 0.002)	0.001 (0.000; 0.002)	0.001 (0.000; 0.002)	0.001 (0.000; 0.002)	0.001 (0.000; 0.002)	0.001 (0.000; 0.002)	0.001 (0.000; 0.002)	0.001 (0.000; 0.002)	0.001 (0.000; 0.002)	0.001 (0.000; 0.002)	0.001 (0.000; 0.002)	0.001 (0.000; 0.002)	0.0[illegible] (0.0[illegible]; 0.0[illegible]
Burkina Faso	0.003 (0.001; 0.005)	0.003 (0.001; 0.005)	0.003 (0.001; 0.005)	0.003 (0.001; 0.005)	0.003 (0.001; 0.005)	0.003 (0.001; 0.005)	0.003 (0.001; 0.005)	0.003 (0.001; 0.005)	0.003 (0.001; 0.005)	0.003 (0.001; 0.005)	0.003 (0.001; 0.005)	0.003 (0.001; 0.005)	0.003 (0.001; 0.005)	0.002 (0.001; 0.005)	0.0[illegible] (0.0[illegible]; 0.0[illegible]
Burundi	0.003 (0.001; 0.006)	0.003 (0.001; 0.006)	0.003 (0.001; 0.006)	0.003 (0.001; 0.005)	0.003 (0.001; 0.005)	0.003 (0.001; 0.005)	0.003 (0.001; 0.005)	0.003 (0.001; 0.005)	0.003 (0.001; 0.005)	0.003 (0.001; 0.005)	0.003 (0.001; 0.005)	0.003 (0.001; 0.005)	0.003 (0.001; 0.005)	0.003 (0.001; 0.005)	0.0[illegible] (0.0[illegible]; 0.0[illegible]
Cabo Verde	0.003 (0.001; 0.006)	0.003 (0.001; 0.006)	0.003 (0.001; 0.006)	0.003 (0.001; 0.006)	0.003 (0.001; 0.006)	0.003 (0.001; 0.006)	0.003 (0.001; 0.006)	0.003 (0.001; 0.006)	0.003 (0.001; 0.006)	0.003 (0.001; 0.006)	0.003 (0.001; 0.006)	0.003 (0.001; 0.006)	0.003 (0.001; 0.005)	0.003 (0.001; 0.005)	0.0[illegible] (0.0[illegible]; 0.0[illegible]
Cambodia	0.001 (0.000; 0.001)	0.001 (0.000; 0.001)	0.001 (0.000; 0.001)	0.001 (0.000; 0.001)	0.001 (0.000; 0.001)	0.001 (0.000; 0.001)	0.001 (0.000; 0.001)	0.001 (0.000; 0.001)	0.001 (0.000; 0.001)	0.001 (0.000; 0.001)	0.001 (0.000; 0.001)	0.001 (0.000; 0.001)	0.001 (0.000; 0.001)	0.001 (0.000; 0.001)	0.0[illegible] (0.0[illegible]; 0.0[illegible]
Cameroon	0.002 (0.001; 0.004)	0.002 (0.001; 0.004)	0.002 (0.001; 0.004)	0.002 (0.001; 0.004)	0.002 (0.001; 0.004)	0.002 (0.001; 0.004)	0.002 (0.001; 0.004)	0.002 (0.001; 0.004)	0.002 (0.001; 0.004)	0.002 (0.001; 0.004)	0.002 (0.001; 0.005)	0.002 (0.001; 0.005)	0.002 (0.001; 0.005)	0.002 (0.001; 0.005)	0.0[illegible] (0.0[illegible]; 0.0[illegible]
Central African Republic	0.001 (0.000; 0.003)	0.001 (0.000; 0.003)	0.001 (0.000; 0.003)	0.001 (0.000; 0.003)	0.001 (0.000; 0.003)	0.001 (0.000; 0.003)	0.001 (0.000; 0.003)	0.001 (0.000; 0.003)	0.001 (0.000; 0.003)	0.001 (0.000; 0.003)	0.001 (0.000; 0.003)	0.001 (0.000; 0.003)	0.001 (0.000; 0.003)	0.001 (0.001; 0.003)	0.0[illegible] (0.0[illegible]; 0.0[illegible]

Chad	0.002 (0.001; 0.003)	0.002 (0.001; 0.003)	0.002 (0.001; 0.003)	0.002 (0.001; 0.003)	0.002 (0.001; 0.003)	0.002 (0.001; 0.003)	0.002 (0.001; 0.003)	0.002 (0.001; 0.003)	0.002 (0.001; 0.003)	0.002 (0.001; 0.003)	0.002 (0.001; 0.003)	0.002 (0.001; 0.003)	0.002 (0.001; 0.003)	0.002 (0.001; 0.003)	[illegible]
China	0.001 (0.000; 0.001)	0.001 (0.000; 0.001)	0.001 (0.000; 0.001)	0.001 (0.000; 0.001)	0.001 (0.000; 0.001)	0.001 (0.000; 0.001)	0.001 (0.000; 0.001)	0.001 (0.000; 0.001)	0.001 (0.000; 0.001)	0.001 (0.000; 0.001)	0.001 (0.000; 0.001)	0.001 (0.000; 0.001)	0.001 (0.000; 0.001)	0.001 (0.000; 0.001)	[illegible]
Colombia	0.002 (0.001; 0.004)	0.002 (0.001; 0.004)	0.002 (0.001; 0.004)	0.002 (0.001; 0.004)	0.002 (0.001; 0.004)	0.002 (0.001; 0.004)	0.002 (0.001; 0.004)	0.002 (0.001; 0.004)	0.002 (0.001; 0.004)	0.002 (0.001; 0.004)	0.002 (0.001; 0.004)	0.002 (0.001; 0.004)	0.002 (0.001; 0.004)	0.002 (0.001; 0.004)	[illegible]
Comoros	0.003 (0.001; 0.005)	0.003 (0.001; 0.005)	0.003 (0.001; 0.005)	0.003 (0.001; 0.005)	0.003 (0.001; 0.005)	0.003 (0.001; 0.005)	0.003 (0.001; 0.005)	0.003 (0.001; 0.005)	0.003 (0.001; 0.005)	0.003 (0.001; 0.005)	0.003 (0.001; 0.005)	0.003 (0.001; 0.005)	0.003 (0.001; 0.005)	0.002 (0.001; 0.005)	[illegible]
Congo, Dem. Rep.	0.002 (0.001; 0.004)	0.002 (0.001; 0.004)	0.002 (0.001; 0.004)	0.002 (0.001; 0.004)	0.002 (0.001; 0.004)	0.002 (0.001; 0.004)	0.002 (0.001; 0.004)	0.002 (0.001; 0.004)	0.002 (0.001; 0.004)	0.002 (0.001; 0.004)	0.002 (0.001; 0.004)	0.002 (0.001; 0.004)	0.002 (0.001; 0.004)	0.002 (0.001; 0.004)	[illegible]
Congo, Rep.	0.002 (0.001; 0.005)	0.002 (0.001; 0.005)	0.002 (0.001; 0.005)	0.002 (0.001; 0.005)	0.002 (0.001; 0.005)	0.002 (0.001; 0.005)	0.003 (0.001; 0.005)	0.003 (0.001; 0.005)	0.003 (0.001; 0.005)	0.003 (0.001; 0.005)	0.002 (0.001; 0.005)	0.002 (0.001; 0.005)	0.002 (0.001; 0.005)	0.002 (0.001; 0.005)	[illegible]
Costa Rica	0.002 (0.001; 0.004)	0.002 (0.001; 0.004)	0.002 (0.001; 0.004)	0.002 (0.001; 0.004)	0.002 (0.001; 0.004)	0.002 (0.001; 0.004)	0.002 (0.001; 0.004)	0.002 (0.001; 0.004)	0.002 (0.001; 0.004)	0.002 (0.001; 0.004)	0.002 (0.001; 0.004)	0.002 (0.001; 0.004)	0.002 (0.001; 0.004)	0.002 (0.001; 0.004)	[illegible]
Cote d'Ivoire	0.003 (0.001; 0.005)	0.003 (0.001; 0.005)	0.003 (0.001; 0.005)	0.003 (0.001; 0.005)	0.003 (0.001; 0.005)	0.003 (0.001; 0.005)	0.003 (0.001; 0.005)	0.003 (0.001; 0.005)	0.003 (0.001; 0.005)	0.003 (0.001; 0.005)	0.002 (0.001; 0.005)	0.002 (0.001; 0.005)	0.002 (0.001; 0.005)	0.002 (0.001; 0.005)	[illegible]
Djibouti	0.001 (0.000; 0.003)	0.001 (0.000; 0.003)	0.001 (0.000; 0.003)	0.001 (0.000; 0.003)	0.001 (0.000; 0.003)	0.001 (0.000; 0.003)	0.001 (0.000; 0.003)	0.001 (0.000; 0.003)	0.001 (0.000; 0.003)	0.001 (0.000; 0.003)	0.001 (0.000; 0.003)	0.001 (0.000; 0.003)	0.001 (0.000; 0.003)	0.001 (0.000; 0.003)	[illegible]
Dominican Republic	0.002 (0.001; 0.004)	0.002 (0.001; 0.004)	0.002 (0.001; 0.004)	0.002 (0.001; 0.004)	0.002 (0.001; 0.004)	0.002 (0.001; 0.003)	0.002 (0.001; 0.003)	0.002 (0.001; 0.003)	0.002 (0.001; 0.003)	0.002 (0.001; 0.003)	0.002 (0.001; 0.003)	0.002 (0.001; 0.003)	0.002 (0.001; 0.003)	0.002 (0.001; 0.003)	[illegible]
Ecuador	0.002 (0.001; 0.004)	0.002 (0.001; 0.004)	0.002 (0.001; 0.004)	0.002 (0.001; 0.004)	0.002 (0.001; 0.004)	0.002 (0.001; 0.003)	0.002 (0.001; 0.003)	0.002 (0.001; 0.003)	0.002 (0.001; 0.003)	0.002 (0.001; 0.003)	0.002 (0.001; 0.003)	0.002 (0.001; 0.003)	0.002 (0.001; 0.003)	0.002 (0.001; 0.003)	[illegible]
Egypt, Arab Rep.	0.001 (0.000; 0.003)	0.001 (0.000; 0.003)	0.001 (0.000; 0.003)	0.001 (0.000; 0.003)	0.001 (0.000; 0.003)	0.001 (0.000; 0.003)	0.001 (0.000; 0.003)	0.001 (0.000; 0.003)	0.001 (0.000; 0.003)	0.001 (0.000; 0.003)	0.001 (0.000; 0.003)	0.001 (0.000; 0.003)	0.001 (0.000; 0.003)	0.001 (0.000; 0.003)	[illegible]

El Salvador	0.002 (0.001; 0.003)	0.002 (0.001; 0.003)	0.002 (0.001; 0.003)	0.002 (0.001; 0.003)	0.002 (0.001; 0.003)	0.002 (0.001; 0.003)	0.002 (0.001; 0.003)	0.002 (0.001; 0.003)	0.002 (0.001; 0.003)	0.002 (0.001; 0.003)	0.002 (0.001; 0.003)	0.002 (0.001; 0.003)	0.002 (0.001; 0.003)	0.002 (0.001; 0.003)	[illegible]
Eritrea	0.003 (0.001; 0.006)	0.003 (0.001; 0.006)	0.003 (0.001; 0.006)	0.003 (0.001; 0.006)	0.003 (0.001; 0.006)	0.003 (0.001; 0.006)	0.003 (0.001; 0.006)	0.003 (0.001; 0.006)	0.003 (0.001; 0.006)	0.003 (0.001; 0.005)	0.003 (0.001; 0.005)	0.003 (0.001; 0.005)	0.003 (0.001; 0.005)	0.003 (0.001; 0.005)	[illegible]
Eswatini	0.003 (0.001; 0.005)	0.003 (0.001; 0.005)	0.003 (0.001; 0.005)	0.003 (0.001; 0.005)	0.003 (0.001; 0.005)	0.003 (0.001; 0.005)	0.003 (0.001; 0.005)	0.003 (0.001; 0.005)	0.003 (0.001; 0.005)	0.003 (0.001; 0.005)	0.002 (0.001; 0.005)	0.002 (0.001; 0.005)	0.002 (0.001; 0.005)	0.002 (0.001; 0.005)	[illegible]
Ethiopia	0.002 (0.001; 0.004)	0.002 (0.001; 0.004)	0.002 (0.001; 0.004)	0.002 (0.001; 0.004)	0.002 (0.001; 0.004)	0.002 (0.001; 0.004)	0.002 (0.001; 0.004)	0.002 (0.001; 0.005)	0.002 (0.001; 0.005)	0.002 (0.001; 0.005)	0.002 (0.001; 0.005)	0.002 (0.001; 0.005)	0.002 (0.001; 0.005)	0.002 (0.001; 0.005)	[illegible]
Fiji	0.001 (0.000; 0.001)	0.001 (0.000; 0.001)	0.001 (0.000; 0.001)	0.001 (0.000; 0.001)	0.001 (0.000; 0.001)	0.001 (0.000; 0.001)	0.001 (0.000; 0.001)	0.001 (0.000; 0.001)	0.001 (0.000; 0.001)	0.001 (0.000; 0.001)	0.001 (0.000; 0.001)	0.001 (0.000; 0.001)	0.001 (0.000; 0.001)	0.001 (0.000; 0.001)	[illegible]
Gabon	0.002 (0.001; 0.004)	0.002 (0.001; 0.004)	0.002 (0.001; 0.004)	0.002 (0.001; 0.005)	0.002 (0.001; 0.005)	0.002 (0.001; 0.005)	0.002 (0.001; 0.005)	0.002 (0.001; 0.005)	0.002 (0.001; 0.005)	0.002 (0.001; 0.005)	0.002 (0.001; 0.005)	0.002 (0.001; 0.005)	0.002 (0.001; 0.005)	0.002 (0.001; 0.005)	[illegible]
Gambia, The	0.003 (0.001; 0.005)	0.003 (0.001; 0.005)	0.003 (0.001; 0.005)	0.003 (0.001; 0.005)	0.003 (0.001; 0.005)	0.003 (0.001; 0.005)	0.003 (0.001; 0.005)	0.003 (0.001; 0.005)	0.003 (0.001; 0.005)	0.003 (0.001; 0.005)	0.002 (0.001; 0.005)	0.002 (0.001; 0.005)	0.002 (0.001; 0.005)	0.002 (0.001; 0.005)	[illegible]
Georgia	0.001 (0.000; 0.002)	0.001 (0.000; 0.002)	0.001 (0.000; 0.002)	0.001 (0.000; 0.002)	0.001 (0.000; 0.002)	0.001 (0.000; 0.002)	0.001 (0.000; 0.002)	0.001 (0.000; 0.002)	0.001 (0.000; 0.002)	0.001 (0.000; 0.002)	0.001 (0.000; 0.002)	0.001 (0.000; 0.002)	0.001 (0.000; 0.002)	0.001 (0.000; 0.002)	[illegible]
Ghana	0.003 (0.001; 0.006)	0.003 (0.001; 0.006)	0.003 (0.001; 0.006)	0.003 (0.001; 0.006)	0.003 (0.001; 0.006)	0.003 (0.001; 0.006)	0.003 (0.001; 0.006)	0.003 (0.001; 0.006)	0.003 (0.001; 0.006)	0.003 (0.001; 0.006)	0.003 (0.001; 0.006)	0.003 (0.001; 0.006)	0.003 (0.001; 0.006)	0.003 (0.001; 0.006)	[illegible]
Guatemala	0.002 (0.001; 0.004)	0.002 (0.001; 0.004)	0.002 (0.001; 0.004)	0.002 (0.001; 0.004)	0.002 (0.001; 0.004)	0.002 (0.001; 0.003)	0.002 (0.001; 0.003)	0.002 (0.001; 0.003)	0.002 (0.001; 0.003)	0.002 (0.001; 0.003)	0.002 (0.001; 0.003)	0.002 (0.001; 0.003)	0.002 (0.001; 0.003)	0.002 (0.001; 0.003)	[illegible]
Guinea	0.001 (0.001; 0.003)	0.001 (0.001; 0.003)	0.001 (0.001; 0.003)	0.001 (0.001; 0.003)	0.001 (0.001; 0.003)	0.001 (0.001; 0.003)	0.001 (0.001; 0.003)	0.001 (0.001; 0.003)	0.002 (0.001; 0.003)	0.002 (0.001; 0.003)	0.002 (0.001; 0.003)	0.002 (0.001; 0.003)	0.002 (0.001; 0.003)	0.002 (0.001; 0.003)	[illegible]
Guinea-Bissau	0.002 (0.001; 0.005)	0.002 (0.001; 0.005)	0.002 (0.001; 0.005)	0.002 (0.001; 0.005)	0.002 (0.001; 0.005)	0.002 (0.001; 0.005)	0.002 (0.001; 0.005)	0.002 (0.001; 0.005)	0.003 (0.001; 0.005)	0.003 (0.001; 0.005)	0.002 (0.001; 0.005)	0.002 (0.001; 0.005)	0.002 (0.001; 0.005)	0.002 (0.001; 0.005)	[illegible]

Haiti	0.001 (0.000; 0.002)	0.001 (0.000; 0.002)	0.001 (0.000; 0.002)	0.001 (0.000; 0.002)	0.001 (0.000; 0.002)	0.001 (0.000; 0.002)	0.001 (0.000; 0.002)	0.001 (0.000; 0.002)	0.001 (0.000; 0.002)	0.001 (0.000; 0.002)	0.001 (0.000; 0.002)	0.001 (0.000; 0.002)	0.001 (0.000; 0.002)	0.001 (0.000; 0.002)	[illegible]
Honduras	0.002 (0.001; 0.004)	0.002 (0.001; 0.004)	0.002 (0.001; 0.004)	0.002 (0.001; 0.004)	0.002 (0.001; 0.004)	0.002 (0.001; 0.003)	0.002 (0.001; 0.003)	0.002 (0.001; 0.003)	0.002 (0.001; 0.003)	0.002 (0.001; 0.003)	0.002 (0.001; 0.003)	0.002 (0.001; 0.003)	0.002 (0.001; 0.003)	0.002 (0.001; 0.003)	[illegible]
India	0.001 (0.000; 0.002)	0.001 (0.000; 0.002)	0.001 (0.000; 0.002)	0.001 (0.000; 0.002)	0.001 (0.000; 0.002)	0.001 (0.000; 0.002)	0.001 (0.000; 0.002)	0.001 (0.000; 0.002)	0.001 (0.000; 0.002)	0.001 (0.000; 0.002)	0.001 (0.000; 0.002)	0.001 (0.000; 0.002)	0.001 (0.000; 0.002)	0.001 (0.000; 0.002)	[illegible]
Indonesia	0.001 (0.000; 0.002)	0.001 (0.000; 0.002)	0.001 (0.000; 0.002)	0.001 (0.000; 0.002)	0.001 (0.000; 0.002)	0.001 (0.000; 0.002)	0.001 (0.000; 0.002)	0.001 (0.000; 0.002)	0.001 (0.000; 0.002)	0.001 (0.000; 0.002)	0.001 (0.000; 0.002)	0.001 (0.000; 0.002)	0.001 (0.000; 0.002)	0.001 (0.000; 0.002)	[illegible]
Iraq	0.001 (0.000; 0.003)	0.001 (0.000; 0.003)	0.001 (0.000; 0.003)	0.001 (0.000; 0.003)	0.001 (0.000; 0.003)	0.001 (0.000; 0.003)	0.001 (0.000; 0.003)	0.001 (0.000; 0.003)	0.001 (0.000; 0.003)	0.001 (0.000; 0.003)	0.001 (0.000; 0.003)	0.001 (0.000; 0.003)	0.001 (0.000; 0.003)	0.001 (0.000; 0.003)	[illegible]
Jamaica	0.002 (0.001; 0.004)	0.002 (0.001; 0.004)	0.002 (0.001; 0.004)	0.002 (0.001; 0.004)	0.002 (0.001; 0.004)	0.002 (0.001; 0.004)	0.002 (0.001; 0.004)	0.002 (0.001; 0.004)	0.002 (0.001; 0.004)	0.002 (0.001; 0.004)	0.002 (0.001; 0.004)	0.002 (0.001; 0.004)	0.002 (0.001; 0.004)	0.002 (0.001; 0.004)	[illegible]
Jordan	0.001 (0.000; 0.003)	0.001 (0.000; 0.003)	0.001 (0.000; 0.003)	0.001 (0.000; 0.003)	0.001 (0.000; 0.003)	0.001 (0.000; 0.003)	0.001 (0.000; 0.003)	0.001 (0.000; 0.003)	0.001 (0.000; 0.003)	0.001 (0.000; 0.003)	0.001 (0.000; 0.003)	0.001 (0.000; 0.003)	0.001 (0.000; 0.003)	0.001 (0.000; 0.003)	[illegible]
Kazakhstan	0.001 (0.000; 0.002)	0.001 (0.000; 0.002)	0.001 (0.000; 0.002)	0.001 (0.000; 0.002)	0.001 (0.000; 0.002)	0.001 (0.000; 0.002)	0.001 (0.000; 0.002)	0.001 (0.000; 0.002)	0.001 (0.000; 0.002)	0.001 (0.000; 0.002)	0.001 (0.000; 0.002)	0.001 (0.000; 0.002)	0.001 (0.000; 0.002)	0.001 (0.000; 0.002)	[illegible]
Kenya	0.003 (0.001; 0.005)	0.003 (0.001; 0.005)	0.003 (0.001; 0.005)	0.003 (0.001; 0.005)	0.003 (0.001; 0.005)	0.003 (0.001; 0.005)	0.003 (0.001; 0.005)	0.003 (0.001; 0.005)	0.003 (0.001; 0.005)	0.003 (0.001; 0.005)	0.003 (0.001; 0.005)	0.003 (0.001; 0.005)	0.003 (0.001; 0.005)	0.002 (0.001; 0.005)	[illegible]
Kiribati	0.001 (0.000; 0.001)	0.001 (0.000; 0.001)	0.001 (0.000; 0.001)	0.001 (0.000; 0.001)	0.001 (0.000; 0.001)	0.001 (0.000; 0.001)	0.001 (0.000; 0.001)	0.001 (0.000; 0.001)	0.001 (0.000; 0.001)	0.001 (0.000; 0.001)	0.001 (0.000; 0.001)	0.001 (0.000; 0.001)	0.001 (0.000; 0.001)	0.001 (0.000; 0.001)	[illegible]
Kyrgyz Republic	0.001 (0.000; 0.002)	0.001 (0.000; 0.002)	0.001 (0.000; 0.002)	0.001 (0.000; 0.002)	0.001 (0.000; 0.002)	0.001 (0.000; 0.002)	0.001 (0.000; 0.002)	0.001 (0.000; 0.002)	0.001 (0.000; 0.002)	0.001 (0.000; 0.002)	0.001 (0.000; 0.002)	0.001 (0.000; 0.002)	0.001 (0.000; 0.002)	0.001 (0.000; 0.002)	[illegible]
Lao PDR	0.001 (0.000; 0.001)	0.001 (0.000; 0.001)	0.001 (0.000; 0.001)	0.001 (0.000; 0.001)	0.001 (0.000; 0.001)	0.001 (0.000; 0.001)	0.001 (0.000; 0.001)	0.001 (0.000; 0.001)	0.001 (0.000; 0.001)	0.001 (0.000; 0.001)	0.001 (0.000; 0.001)	0.001 (0.000; 0.001)	0.001 (0.000; 0.001)	0.001 (0.000; 0.001)	[illegible]

Lesotho	0.003 (0.001; 0.005)	0.003 (0.001; 0.005)	0.003 (0.001; 0.005)	0.003 (0.001; 0.005)	0.003 (0.001; 0.005)	0.003 (0.001; 0.005)	0.003 (0.001; 0.005)	0.003 (0.001; 0.005)	0.003 (0.001; 0.005)	0.003 (0.001; 0.005)	0.002 (0.001; 0.005)	0.002 (0.001; 0.005)	0.002 (0.001; 0.005)	0.002 (0.001; 0.005)	[illegible]
Liberia	0.002 (0.001; 0.004)	0.002 (0.001; 0.004)	0.002 (0.001; 0.004)	0.002 (0.001; 0.005)	0.002 (0.001; 0.005)	0.002 (0.001; 0.005)	0.002 (0.001; 0.005)	0.002 (0.001; 0.005)	0.002 (0.001; 0.005)	0.002 (0.001; 0.005)	0.002 (0.001; 0.005)	0.002 (0.001; 0.005)	0.002 (0.001; 0.005)	0.002 (0.001; 0.005)	[illegible]
Madagascar	0.002 (0.001; 0.004)	0.002 (0.001; 0.004)	0.002 (0.001; 0.004)	0.002 (0.001; 0.005)	0.002 (0.001; 0.005)	0.002 (0.001; 0.005)	0.002 (0.001; 0.005)	0.002 (0.001; 0.005)	0.002 (0.001; 0.005)	0.002 (0.001; 0.005)	0.002 (0.001; 0.005)	0.002 (0.001; 0.005)	0.002 (0.001; 0.005)	0.002 (0.001; 0.005)	[illegible]
Malawi	0.003 (0.001; 0.006)	0.003 (0.001; 0.006)	0.003 (0.001; 0.006)	0.003 (0.001; 0.006)	0.003 (0.001; 0.006)	0.003 (0.001; 0.006)	0.003 (0.001; 0.006)	0.003 (0.001; 0.006)	0.003 (0.001; 0.006)	0.003 (0.001; 0.005)	0.003 (0.001; 0.005)	0.003 (0.001; 0.005)	0.003 (0.001; 0.005)	0.003 (0.001; 0.005)	[illegible]
Malaysia	0.001 (0.000; 0.001)	0.001 (0.000; 0.001)	0.001 (0.000; 0.001)	0.001 (0.000; 0.001)	0.001 (0.000; 0.001)	0.001 (0.000; 0.001)	0.001 (0.000; 0.001)	0.001 (0.000; 0.001)	0.001 (0.000; 0.001)	0.001 (0.000; 0.001)	0.001 (0.000; 0.001)	0.001 (0.000; 0.001)	0.001 (0.000; 0.001)	0.001 (0.000; 0.001)	[illegible]
Mali	0.002 (0.001; 0.005)	0.002 (0.001; 0.005)	0.002 (0.001; 0.005)	0.002 (0.001; 0.005)	0.002 (0.001; 0.005)	0.002 (0.001; 0.005)	0.002 (0.001; 0.005)	0.002 (0.001; 0.005)	0.002 (0.001; 0.005)	0.002 (0.001; 0.005)	0.002 (0.001; 0.005)	0.002 (0.001; 0.005)	0.002 (0.001; 0.005)	0.002 (0.001; 0.005)	[illegible]
Mauritania	0.002 (0.001; 0.005)	0.002 (0.001; 0.005)	0.003 (0.001; 0.005)	0.003 (0.001; 0.005)	0.003 (0.001; 0.005)	0.003 (0.001; 0.005)	0.003 (0.001; 0.005)	0.003 (0.001; 0.005)	0.003 (0.001; 0.005)	0.003 (0.001; 0.005)	0.002 (0.001; 0.005)	0.002 (0.001; 0.005)	0.002 (0.001; 0.005)	0.002 (0.001; 0.005)	[illegible]
Mexico	0.002 (0.001; 0.003)	0.002 (0.001; 0.003)	0.002 (0.001; 0.003)	0.002 (0.001; 0.004)	0.002 (0.001; 0.004)	0.002 (0.001; 0.003)	0.002 (0.001; 0.003)	0.002 (0.001; 0.003)	0.002 (0.001; 0.003)	0.002 (0.001; 0.003)	0.002 (0.001; 0.003)	0.002 (0.001; 0.003)	0.002 (0.001; 0.003)	0.002 (0.001; 0.003)	[illegible]
Mongolia	0.001 (0.000; 0.001)	0.001 (0.000; 0.001)	0.001 (0.000; 0.001)	0.001 (0.000; 0.001)	0.001 (0.000; 0.001)	0.001 (0.000; 0.001)	0.001 (0.000; 0.001)	0.001 (0.000; 0.001)	0.001 (0.000; 0.001)	0.001 (0.000; 0.001)	0.001 (0.000; 0.001)	0.001 (0.000; 0.001)	0.001 (0.000; 0.001)	0.001 (0.000; 0.001)	[illegible]
Morocco	0.002 (0.001; 0.003)	0.002 (0.001; 0.003)	0.002 (0.001; 0.003)	0.002 (0.001; 0.003)	0.002 (0.001; 0.003)	0.002 (0.001; 0.003)	0.002 (0.001; 0.003)	0.002 (0.001; 0.003)	0.001 (0.000; 0.003)	0.001 (0.000; 0.003)	0.001 (0.000; 0.003)	0.001 (0.000; 0.003)	0.001 (0.000; 0.003)	0.001 (0.000; 0.003)	[illegible]
Mozambique	0.003 (0.001; 0.005)	0.003 (0.001; 0.005)	0.003 (0.001; 0.005)	0.003 (0.001; 0.005)	0.003 (0.001; 0.005)	0.003 (0.001; 0.005)	0.003 (0.001; 0.005)	0.003 (0.001; 0.005)	0.003 (0.001; 0.005)	0.003 (0.001; 0.005)	0.002 (0.001; 0.005)	0.002 (0.001; 0.005)	0.002 (0.001; 0.005)	0.002 (0.001; 0.005)	[illegible]
Myanmar	0.001 (0.000; 0.002)	0.001 (0.000; 0.002)	0.001 (0.000; 0.002)	0.001 (0.000; 0.002)	0.001 (0.000; 0.002)	0.001 (0.000; 0.002)	0.001 (0.000; 0.002)	0.001 (0.000; 0.002)	0.001 (0.000; 0.002)	0.001 (0.000; 0.002)	0.001 (0.000; 0.002)	0.001 (0.000; 0.002)	0.001 (0.000; 0.002)	0.001 (0.000; 0.002)	[illegible]

Namibia	0.003 (0.001; 0.005)	0.003 (0.001; 0.005)	0.003 (0.001; 0.005)	0.003 (0.001; 0.005)	0.003 (0.001; 0.005)	0.003 (0.001; 0.005)	0.003 (0.001; 0.005)	0.003 (0.001; 0.005)	0.003 (0.001; 0.005)	0.003 (0.001; 0.005)	0.002 (0.001; 0.005)	0.002 (0.001; 0.005)	0.002 (0.001; 0.005)	0.002 (0.001; 0.005)	[illegible]
Nepal	0.001 (0.000; 0.002)	0.001 (0.000; 0.002)	0.001 (0.000; 0.002)	0.001 (0.000; 0.002)	0.001 (0.000; 0.002)	0.001 (0.000; 0.002)	0.001 (0.000; 0.002)	0.001 (0.000; 0.002)	0.001 (0.000; 0.002)	0.001 (0.000; 0.002)	0.001 (0.000; 0.002)	0.001 (0.000; 0.002)	0.001 (0.000; 0.002)	0.001 (0.000; 0.002)	[illegible]
Nicaragua	0.002 (0.001; 0.004)	0.002 (0.001; 0.004)	0.002 (0.001; 0.004)	0.002 (0.001; 0.004)	0.002 (0.001; 0.004)	0.002 (0.001; 0.004)	0.002 (0.001; 0.004)	0.002 (0.001; 0.004)	0.002 (0.001; 0.004)	0.002 (0.001; 0.004)	0.002 (0.001; 0.004)	0.002 (0.001; 0.004)	0.002 (0.001; 0.004)	0.002 (0.001; 0.004)	[illegible]
Niger	0.002 (0.001; 0.005)	0.002 (0.001; 0.005)	0.003 (0.001; 0.005)	0.003 (0.001; 0.005)	0.003 (0.001; 0.005)	0.003 (0.001; 0.005)	0.003 (0.001; 0.005)	0.003 (0.001; 0.005)	0.003 (0.001; 0.005)	0.003 (0.001; 0.005)	0.002 (0.001; 0.005)	0.002 (0.001; 0.005)	0.002 (0.001; 0.005)	0.002 (0.001; 0.005)	[illegible]
Nigeria	0.002 (0.001; 0.004)	0.002 (0.001; 0.004)	0.002 (0.001; 0.004)	0.002 (0.001; 0.004)	0.002 (0.001; 0.004)	0.002 (0.001; 0.004)	0.002 (0.001; 0.004)	0.002 (0.001; 0.004)	0.002 (0.001; 0.004)	0.002 (0.001; 0.004)	0.002 (0.001; 0.004)	0.002 (0.001; 0.004)	0.002 (0.001; 0.004)	0.002 (0.001; 0.004)	[illegible]
Pakistan	0.001 (0.000; 0.003)	0.001 (0.000; 0.003)	0.001 (0.000; 0.003)	0.001 (0.000; 0.003)	0.001 (0.000; 0.003)	0.001 (0.000; 0.003)	0.001 (0.000; 0.003)	0.001 (0.000; 0.003)	0.001 (0.000; 0.003)	0.001 (0.000; 0.003)	0.001 (0.000; 0.003)	0.001 (0.000; 0.003)	0.001 (0.000; 0.003)	0.001 (0.000; 0.003)	[illegible]
Papua New Guinea	0.000 (0.000; 0.001)	0.000 (0.000; 0.001)	0.000 (0.000; 0.001)	0.000 (0.000; 0.001)	0.000 (0.000; 0.001)	0.000 (0.000; 0.001)	0.000 (0.000; 0.001)	0.000 (0.000; 0.001)	0.000 (0.000; 0.001)	0.000 (0.000; 0.001)	0.000 (0.000; 0.001)	0.000 (0.000; 0.001)	0.000 (0.000; 0.001)	0.000 (0.000; 0.001)	[illegible]
Paraguay	0.002 (0.001; 0.004)	0.002 (0.001; 0.004)	0.002 (0.001; 0.004)	0.002 (0.001; 0.004)	0.002 (0.001; 0.004)	0.002 (0.001; 0.003)	0.002 (0.001; 0.003)	0.002 (0.001; 0.003)	0.002 (0.001; 0.003)	0.002 (0.001; 0.003)	0.002 (0.001; 0.003)	0.002 (0.001; 0.003)	0.002 (0.001; 0.003)	0.002 (0.001; 0.003)	[illegible]
Peru	0.002 (0.001; 0.004)	0.002 (0.001; 0.004)	0.002 (0.001; 0.004)	0.002 (0.001; 0.004)	0.002 (0.001; 0.004)	0.002 (0.001; 0.003)	0.002 (0.001; 0.003)	0.002 (0.001; 0.003)	0.002 (0.001; 0.003)	0.002 (0.001; 0.003)	0.002 (0.001; 0.003)	0.002 (0.001; 0.003)	0.002 (0.001; 0.003)	0.002 (0.001; 0.003)	[illegible]
Philippines	0.001 (0.000; 0.001)	0.001 (0.000; 0.001)	0.001 (0.000; 0.001)	0.001 (0.000; 0.001)	0.001 (0.000; 0.001)	0.001 (0.000; 0.001)	0.001 (0.000; 0.001)	0.001 (0.000; 0.001)	0.001 (0.000; 0.001)	0.001 (0.000; 0.001)	0.001 (0.000; 0.001)	0.001 (0.000; 0.001)	0.001 (0.000; 0.001)	0.001 (0.000; 0.001)	[illegible]
Rwanda	0.003 (0.001; 0.006)	0.003 (0.001; 0.006)	0.003 (0.001; 0.006)	0.003 (0.001; 0.006)	0.003 (0.001; 0.006)	0.003 (0.001; 0.006)	0.003 (0.001; 0.006)	0.003 (0.001; 0.006)	0.003 (0.001; 0.006)	0.003 (0.001; 0.006)	0.003 (0.001; 0.006)	0.003 (0.001; 0.006)	0.003 (0.001; 0.006)	0.003 (0.001; 0.006)	[illegible]
Senegal	0.003 (0.001; 0.006)	0.003 (0.001; 0.006)	0.003 (0.001; 0.006)	0.003 (0.001; 0.006)	0.003 (0.001; 0.006)	0.003 (0.001; 0.006)	0.003 (0.001; 0.006)	0.003 (0.001; 0.006)	0.003 (0.001; 0.006)	0.003 (0.001; 0.005)	0.003 (0.001; 0.005)	0.003 (0.001; 0.005)	0.003 (0.001; 0.005)	0.003 (0.001; 0.005)	[illegible]

Sierra Leone	0.003 (0.001; 0.006)	0.003 (0.001; 0.006)	0.003 (0.001; 0.006)	0.003 (0.001; 0.006)	0.003 (0.001; 0.006)	0.003 (0.001; 0.006)	0.003 (0.001; 0.006)	0.003 (0.001; 0.006)	0.003 (0.001; 0.006)	0.003 (0.001; 0.005)	0.003 (0.001; 0.005)	0.003 (0.001; 0.005)	0.003 (0.001; 0.005)	0.003 (0.001; 0.005)	[illegible]
Solomon Islands	0.001 (0.000; 0.001)	0.001 (0.000; 0.001)	0.001 (0.000; 0.001)	0.001 (0.000; 0.001)	0.001 (0.000; 0.001)	0.001 (0.000; 0.001)	0.001 (0.000; 0.001)	0.001 (0.000; 0.001)	0.001 (0.000; 0.001)	0.001 (0.000; 0.001)	0.001 (0.000; 0.001)	0.001 (0.000; 0.001)	0.001 (0.000; 0.001)	0.001 (0.000; 0.001)	[illegible]
Somalia	0.001 (0.000; 0.001)	0.001 (0.000; 0.001)	0.001 (0.000; 0.001)	0.001 (0.000; 0.001)	0.001 (0.000; 0.001)	0.001 (0.000; 0.001)	0.001 (0.000; 0.001)	0.001 (0.000; 0.001)	0.001 (0.000; 0.001)	0.001 (0.000; 0.002)	0.001 (0.000; 0.002)	0.001 (0.000; 0.002)	0.001 (0.000; 0.002)	0.001 (0.000; 0.002)	[illegible]
South Africa	0.003 (0.001; 0.005)	0.003 (0.001; 0.005)	0.003 (0.001; 0.005)	0.003 (0.001; 0.005)	0.003 (0.001; 0.005)	0.003 (0.001; 0.005)	0.003 (0.001; 0.005)	0.003 (0.001; 0.005)	0.003 (0.001; 0.005)	0.003 (0.001; 0.005)	0.002 (0.001; 0.005)	0.002 (0.001; 0.005)	0.002 (0.001; 0.005)	0.002 (0.001; 0.005)	[illegible]
Sri Lanka	0.001 (0.000; 0.002)	0.001 (0.000; 0.002)	0.001 (0.000; 0.002)	0.001 (0.000; 0.002)	0.001 (0.000; 0.002)	0.001 (0.000; 0.002)	0.001 (0.000; 0.002)	0.001 (0.000; 0.002)	0.001 (0.000; 0.002)	0.001 (0.000; 0.002)	0.001 (0.000; 0.002)	0.001 (0.000; 0.002)	0.001 (0.000; 0.002)	0.001 (0.000; 0.002)	[illegible]
Sudan	0.001 (0.000; 0.003)	0.001 (0.000; 0.003)	0.001 (0.000; 0.003)	0.001 (0.000; 0.003)	0.001 (0.000; 0.003)	0.001 (0.000; 0.003)	0.001 (0.000; 0.003)	0.001 (0.000; 0.003)	0.001 (0.000; 0.003)	0.001 (0.000; 0.003)	0.001 (0.000; 0.003)	0.001 (0.000; 0.003)	0.001 (0.000; 0.003)	0.001 (0.000; 0.003)	[illegible]
Suriname	0.002 (0.001; 0.003)	0.002 (0.001; 0.003)	0.002 (0.001; 0.003)	0.002 (0.001; 0.003)	0.002 (0.001; 0.003)	0.002 (0.001; 0.003)	0.002 (0.001; 0.003)	0.002 (0.001; 0.003)	0.002 (0.001; 0.003)	0.002 (0.001; 0.003)	0.002 (0.001; 0.003)	0.002 (0.001; 0.003)	0.002 (0.001; 0.003)	0.002 (0.001; 0.003)	[illegible]
Tajikistan	0.001 (0.000; 0.002)	0.001 (0.000; 0.002)	0.001 (0.000; 0.002)	0.001 (0.000; 0.002)	0.001 (0.000; 0.002)	0.001 (0.000; 0.002)	0.001 (0.000; 0.002)	0.001 (0.000; 0.002)	0.001 (0.000; 0.002)	0.001 (0.000; 0.002)	0.001 (0.000; 0.002)	0.001 (0.000; 0.002)	0.001 (0.000; 0.002)	0.001 (0.000; 0.002)	[illegible]
Tanzania	0.003 (0.001; 0.005)	0.003 (0.001; 0.005)	0.003 (0.001; 0.005)	0.003 (0.001; 0.005)	0.003 (0.001; 0.005)	0.003 (0.001; 0.005)	0.003 (0.001; 0.005)	0.003 (0.001; 0.005)	0.003 (0.001; 0.005)	0.003 (0.001; 0.005)	0.002 (0.001; 0.005)	0.002 (0.001; 0.005)	0.002 (0.001; 0.005)	0.002 (0.001; 0.005)	[illegible]
Thailand	0.001 (0.000; 0.002)	0.001 (0.000; 0.002)	0.001 (0.000; 0.002)	0.001 (0.000; 0.002)	0.001 (0.000; 0.002)	0.001 (0.000; 0.002)	0.001 (0.000; 0.002)	0.001 (0.000; 0.002)	0.001 (0.000; 0.002)	0.001 (0.000; 0.002)	0.001 (0.000; 0.002)	0.001 (0.000; 0.002)	0.001 (0.000; 0.002)	0.001 (0.000; 0.002)	[illegible]
Timor-Leste	0.001 (0.000; 0.002)	0.001 (0.000; 0.002)	0.001 (0.000; 0.002)	0.001 (0.000; 0.002)	0.001 (0.000; 0.002)	0.001 (0.000; 0.002)	0.001 (0.000; 0.002)	0.001 (0.000; 0.002)	0.001 (0.000; 0.002)	0.001 (0.000; 0.002)	0.001 (0.000; 0.002)	0.001 (0.000; 0.002)	0.001 (0.000; 0.002)	0.001 (0.000; 0.002)	[illegible]
Togo	0.003 (0.001; 0.005)	0.003 (0.001; 0.005)	0.003 (0.001; 0.005)	0.003 (0.001; 0.005)	0.003 (0.001; 0.005)	0.003 (0.001; 0.005)	0.003 (0.001; 0.005)	0.003 (0.001; 0.005)	0.003 (0.001; 0.005)	0.003 (0.001; 0.005)	0.002 (0.001; 0.005)	0.002 (0.001; 0.005)	0.002 (0.001; 0.005)	0.002 (0.001; 0.005)	[illegible]

Tunisia	0.001 (0.000; 0.003)	0.001 (0.000; 0.003)	0.001 (0.000; 0.003)	0.001 (0.000; 0.003)	0.001 (0.000; 0.003)	0.001 (0.000; 0.003)	0.001 (0.000; 0.003)	0.001 (0.000; 0.003)	0.001 (0.000; 0.003)	0.001 (0.000; 0.003)	0.001 (0.000; 0.003)	0.001 (0.000; 0.003)	0.001 (0.000; 0.003)	0.001 (0.000; 0.003)	[illegible]
Turkey	0.001 (0.000; 0.002)	0.001 (0.000; 0.002)	0.001 (0.000; 0.002)	0.001 (0.000; 0.002)	0.001 (0.000; 0.002)	0.001 (0.000; 0.002)	0.001 (0.000; 0.002)	0.001 (0.000; 0.002)	0.001 (0.000; 0.002)	0.001 (0.000; 0.002)	0.001 (0.000; 0.002)	0.001 (0.000; 0.002)	0.001 (0.000; 0.002)	0.001 (0.000; 0.002)	[illegible]
Uganda	0.003 (0.001; 0.006)	0.003 (0.001; 0.006)	0.003 (0.001; 0.006)	0.003 (0.001; 0.005)	0.003 (0.001; 0.005)	0.003 (0.001; 0.005)	0.003 (0.001; 0.005)	0.003 (0.001; 0.005)	0.003 (0.001; 0.005)	0.003 (0.001; 0.005)	0.003 (0.001; 0.005)	0.003 (0.001; 0.005)	0.003 (0.001; 0.005)	0.003 (0.001; 0.005)	[illegible]
Ukraine	0.001 (0.000; 0.002)	0.001 (0.000; 0.002)	0.001 (0.000; 0.002)	0.001 (0.000; 0.002)	0.001 (0.000; 0.002)	0.001 (0.000; 0.002)	0.001 (0.000; 0.002)	0.001 (0.000; 0.002)	0.001 (0.000; 0.002)	0.001 (0.000; 0.002)	0.001 (0.000; 0.002)	0.001 (0.000; 0.002)	0.001 (0.000; 0.002)	0.001 (0.000; 0.002)	[illegible]
Uzbekistan	0.001 (0.000; 0.002)	0.001 (0.000; 0.002)	0.001 (0.000; 0.002)	0.001 (0.000; 0.002)	0.001 (0.000; 0.002)	0.001 (0.000; 0.002)	0.001 (0.000; 0.002)	0.001 (0.000; 0.002)	0.001 (0.000; 0.002)	0.001 (0.000; 0.002)	0.001 (0.000; 0.002)	0.001 (0.000; 0.002)	0.001 (0.000; 0.002)	0.001 (0.000; 0.002)	[illegible]
Vanuatu	0.001 (0.000; 0.001)	0.001 (0.000; 0.001)	0.001 (0.000; 0.001)	0.001 (0.000; 0.001)	0.001 (0.000; 0.001)	0.001 (0.000; 0.001)	0.001 (0.000; 0.001)	0.001 (0.000; 0.001)	0.001 (0.000; 0.001)	0.001 (0.000; 0.001)	0.001 (0.000; 0.001)	0.001 (0.000; 0.001)	0.001 (0.000; 0.001)	0.001 (0.000; 0.001)	[illegible]
Vietnam	0.001 (0.000; 0.001)	0.001 (0.000; 0.001)	0.001 (0.000; 0.001)	0.001 (0.000; 0.001)	0.001 (0.000; 0.001)	0.001 (0.000; 0.001)	0.001 (0.000; 0.001)	0.001 (0.000; 0.001)	0.001 (0.000; 0.001)	0.001 (0.000; 0.001)	0.001 (0.000; 0.001)	0.001 (0.000; 0.001)	0.001 (0.000; 0.001)	0.001 (0.000; 0.001)	[illegible]
Yemen, Rep.	0.001 (0.000; 0.002)	0.001 (0.000; 0.002)	0.001 (0.000; 0.003)	0.001 (0.000; 0.003)	0.001 (0.000; 0.003)	0.001 (0.000; 0.003)	0.001 (0.000; 0.003)	0.001 (0.000; 0.003)	0.001 (0.000; 0.003)	0.001 (0.000; 0.003)	0.001 (0.000; 0.003)	0.001 (0.000; 0.003)	0.001 (0.000; 0.003)	0.001 (0.000; 0.003)	[illegible]
Zambia	0.003 (0.001; 0.005)	0.003 (0.001; 0.005)	0.003 (0.001; 0.005)	0.003 (0.001; 0.005)	0.003 (0.001; 0.005)	0.003 (0.001; 0.005)	0.003 (0.001; 0.005)	0.003 (0.001; 0.005)	0.003 (0.001; 0.005)	0.003 (0.001; 0.005)	0.002 (0.001; 0.005)	0.002 (0.001; 0.005)	0.002 (0.001; 0.005)	0.002 (0.001; 0.005)	[illegible]
Zimbabwe	0.003 (0.001; 0.005)	0.003 (0.001; 0.005)	0.003 (0.001; 0.005)	0.003 (0.001; 0.005)	0.003 (0.001; 0.005)	0.003 (0.001; 0.005)	0.003 (0.001; 0.005)	0.003 (0.001; 0.005)	0.003 (0.001; 0.005)	0.003 (0.001; 0.005)	0.002 (0.001; 0.005)	0.002 (0.001; 0.005)	0.002 (0.001; 0.005)	0.002 (0.001; 0.005)	[illegible]

Table S21. Net costs of vaccination estimates assuming the vaccine is effective against Shigella less severe diarrhoea (LSD) and Shigella moderate-to-severe diarrhoea (MSD) from companion cost effectiveness analysis. Costs were aggregated from 2025 to 2044 and are reported in 2019 US$. Estimates in parenthesis represent 95% uncertainty estimates from the results of 1000 model simulations.

Country	2025	2026	2027	2028	2029	2030	2031	2032	2033

Afghanistan	5,522,617 (3,141,705; 8,222,023)	5,583,756 (3,184,625; 8,305,532)	5,645,542 (3,228,032; 8,389,886)	5,707,982 (3,271,933; 8,475,093)	5,771,081 (3,316,376; 8,561,162)	5,913,291 (3,406,557; 8,764,367)	5,978,597 (3,452,670; 8,853,367)	6,044,594 (3,499,305; 8,943,268)	6,111,287 (3,546,467; 9,034,078)
Algeria	11,994,469 (3,733,336; 21,066,745)	12,017,078 (3,773,910; 21,084,489)	12,039,597 (3,816,485; 21,102,159)	12,062,026 (3,870,436; 21,119,754)	12,084,367 (3,924,153; 21,137,275)	10,966,077 (3,602,912; 19,161,776)	10,986,153 (3,651,147; 19,177,514)	11,006,149 (3,699,173; 19,193,185)	11,026,066 (3,746,991; 19,208,790)
Angola	9,296,696 (-106,165; 18,841,632)	9,419,101 (-45,418; 19,054,163)	9,542,906 (14,145; 19,268,981)	9,668,126 (55,915; 19,486,109)	9,794,777 (98,366; 19,705,571)	11,198,526 (159,699; 22,489,189)	11,344,741 (209,174; 22,742,216)	11,492,623 (259,447; 22,997,960)	11,642,190 (310,528; 23,256,451)
Argentina	7,839,495 (1,612,004; 14,740,848)	7,938,900 (1,661,534; 14,902,479)	8,039,425 (1,712,298; 15,065,822)	8,071,498 (1,748,730; 15,100,712)	8,092,494 (1,782,715; 15,114,955)	8,033,944 (1,798,804; 14,980,975)	8,054,569 (1,832,249; 14,994,985)	8,075,113 (1,849,953; 15,008,949)	8,095,575 (1,879,574; 15,022,866)
Armenia	834,729 (499,290; 1,282,381)	835,038 (499,707; 1,282,643)	835,346 (500,121; 1,282,904)	835,653 (500,535; 1,283,165)	835,959 (500,947; 1,283,424)	734,878 (440,574; 1,128,053)	735,144 (440,934; 1,128,279)	735,410 (441,292; 1,128,505)	735,674 (441,648; 1,128,729)
Azerbaijan	3,289,964 (1,914,933; 5,090,260)	3,291,387 (1,917,941; 5,091,465)	3,292,804 (1,920,940; 5,092,665)	3,294,216 (1,923,930; 5,093,861)	3,295,621 (1,926,912; 5,095,051)	2,982,183 (1,745,596; 4,609,588)	2,983,444 (1,748,277; 4,610,657)	2,984,700 (1,750,950; 4,611,722)	2,985,952 (1,753,616; 4,612,782)
Bangladesh	22,092,441 (15,413,230; 29,400,372)	22,106,471 (15,423,515; 29,413,899)	22,120,444 (15,433,787; 29,422,329)	22,134,363 (15,444,074; 29,430,731)	22,148,226 (15,454,312; 29,439,108)	20,852,733 (14,550,879; 27,707,743)	20,865,674 (14,560,421; 27,715,575)	20,878,564 (14,569,917; 27,723,382)	20,891,404 (14,579,368; 27,731,165)
Belarus	2,419,460 (1,350,278; 3,808,865)	2,420,816 (1,352,531; 3,809,872)	2,422,167 (1,355,024; 3,810,876)	2,423,512 (1,357,665; 3,811,876)	2,424,853 (1,360,296; 3,812,874)	2,153,191 (1,209,561; 3,384,729)	2,154,371 (1,211,879; 3,385,610)	2,155,546 (1,214,189; 3,386,488)	2,156,716 (1,216,491; 3,387,363)
Benin	1,917,818 (497,753; 3,254,005)	1,942,112 (509,026; 3,289,709)	1,966,679 (520,458; 3,325,785)	1,991,523 (532,051; 3,362,235)	2,016,646 (543,807; 3,400,633)	2,225,635 (605,775; 3,748,803)	2,253,636 (619,076; 3,791,701)	2,281,952 (632,564; 3,835,054)	2,310,585 (646,240; 3,878,865)
Bhutan	231,362 (139,132; 342,337)	231,458 (139,339; 342,437)	231,553 (139,461; 342,537)	231,648 (139,521; 342,636)	231,743 (139,580; 342,722)	214,184 (129,008; 316,706)	214,271 (129,063; 316,786)	214,357 (129,117; 316,865)	214,443 (129,172; 316,943)
Bolivia	3,007,243 (1,476,345; 4,820,203)	3,040,949 (1,499,567; 4,871,626)	3,075,014 (1,518,999; 4,923,581)	3,109,442 (1,538,426; 4,976,073)	3,144,236 (1,562,055; 5,029,109)	3,196,557 (1,594,516; 5,110,122)	3,232,287 (1,618,825; 5,164,553)	3,268,396 (1,643,416; 5,219,547)	3,304,890 (1,666,381; 5,275,588)
Botswana	663,920 (-253,575; 1,424,058)	666,701 (-250,752; 1,424,954)	669,472 (-247,937; 1,426,238)	672,231 (-245,129; 1,427,680)	674,980 (-242,254; 1,429,118)	679,363 (-239,948; 1,434,025)	682,096 (-237,062; 1,435,459)	684,819 (-234,183; 1,436,889)	687,531 (-231,312; 1,438,314)

Brazil	34,859,766 (19,457,498; 54,043,032)	35,234,411 (19,677,347; 54,595,088)	35,612,959 (19,899,524; 55,152,757)	35,995,448 (20,124,054; 55,720,398)	36,381,920 (20,351,066; 56,303,183)	34,459,382 (19,286,129; 53,313,378)	34,829,123 (19,503,578; 53,870,789)	35,202,713 (19,723,326; 54,433,929)	35,580,190 (19,945,398; 55,002,855)
Bulgaria	1,275,826 (691,721; 2,035,940)	1,276,668 (693,173; 2,036,349)	1,277,507 (694,294; 2,036,756)	1,278,343 (695,410; 2,037,161)	1,279,175 (696,375; 2,037,560)	1,205,053 (656,122; 1,918,620)	1,205,830 (656,643; 1,918,989)	1,206,604 (657,163; 1,919,356)	1,207,375 (657,681; 1,919,721)
Burkina Faso	2,970,851 (173,084; 5,500,729)	2,980,510 (182,816; 5,501,539)	2,990,130 (192,525; 5,502,348)	2,999,712 (202,211; 5,503,156)	3,009,256 (211,873; 5,503,963)	3,302,675 (242,346; 6,022,489)	3,313,034 (252,867; 6,023,370)	3,323,352 (263,363; 6,028,789)	3,333,629 (274,754; 6,036,715)
Burundi	1,983,325 (429,337; 3,441,603)	1,988,347 (437,886; 3,444,208)	1,993,349 (446,402; 3,446,805)	1,998,332 (454,989; 3,449,396)	2,003,294 (463,582; 3,451,979)	2,159,952 (507,810; 3,715,535)	2,165,248 (516,980; 3,718,298)	2,170,522 (526,114; 3,721,053)	2,175,775 (535,213; 3,723,801)
Cabo Verde	141,578 (52,344; 243,186)	141,813 (52,596; 243,369)	142,047 (52,847; 243,550)	142,281 (53,097; 243,731)	142,513 (53,346; 243,912)	134,011 (50,315; 229,156)	134,227 (50,547; 229,323)	134,443 (50,778; 229,491)	134,658 (51,008; 229,657)
Cambodia	2,696,387 (1,963,242; 3,564,908)	2,697,749 (1,965,678; 3,565,827)	2,699,105 (1,968,102; 3,566,743)	2,700,456 (1,970,529; 3,567,656)	2,701,802 (1,972,955; 3,568,565)	2,618,312 (1,913,377; 3,457,454)	2,619,605 (1,915,704; 3,458,329)	2,620,893 (1,918,019; 3,459,201)	2,622,175 (1,920,323; 3,460,070)
Cameroon	3,221,040 (194,644; 5,880,512)	3,264,089 (218,362; 5,944,856)	3,307,633 (242,459; 6,009,878)	3,351,678 (266,938; 6,076,284)	3,396,230 (291,805; 6,143,953)	3,690,311 (340,007; 6,661,886)	3,739,190 (367,519; 6,736,017)	3,788,629 (395,462; 6,810,941)	3,838,634 (423,842; 6,886,669)
Central African Republic	305,115 (16,025; 566,384)	309,174 (17,242; 572,132)	313,280 (18,439; 577,938)	317,433 (19,655; 583,804)	321,633 (20,892; 589,728)	352,013 (23,924; 643,480)	356,655 (25,318; 650,010)	361,350 (26,876; 656,731)	366,098 (28,460; 663,920)
Chad	1,413,624 (-3,951; 2,688,373)	1,432,798 (3,952; 2,717,789)	1,452,195 (9,102; 2,747,517)	1,471,815 (14,342; 2,777,561)	1,491,662 (19,674; 2,807,924)	1,639,705 (27,223; 3,078,891)	1,661,732 (33,208; 3,112,527)	1,684,014 (39,297; 3,145,279)	1,706,552 (45,490; 3,177,220)
China	376,059,238 (214,797,797; 579,055,435)	376,225,394 (214,958,350; 579,302,471)	376,390,889 (215,161,926; 579,548,589)	376,555,726 (215,412,057; 579,793,793)	376,719,908 (215,661,069; 580,038,085)	350,674,793 (200,894,561; 539,928,434)	350,826,346 (201,124,189; 540,154,051)	350,977,297 (201,352,789; 540,378,831)	351,127,649 (201,580,368; 540,602,775)
Colombia	5,635,225 (-1,966,911; 13,029,449)	5,664,303 (-1,918,924; 13,054,786)	5,693,265 (-1,870,770; 13,092,251)	5,722,112 (-1,822,828; 13,112,320)	5,750,844 (-1,777,305; 13,132,323)	5,415,887 (-1,642,478; 12,324,874)	5,442,597 (-1,585,354; 12,343,492)	5,469,202 (-1,532,522; 12,362,048)	5,495,701 (-1,489,761; 12,380,053)
Comoros	132,897 (33,583; 226,226)	133,251 (34,019; 226,445)	133,604 (34,455; 226,663)	133,956 (34,889; 226,880)	134,307 (35,322; 227,096)	140,358 (37,273; 237,031)	140,720 (37,730; 237,372)	141,081 (38,186; 237,711)	141,440 (38,641; 238,049)

Congo, Dem. Rep.	9,951,833 (1,918,320; 17,442,958)	10,077,847 (1,982,612; 17,631,131)	10,205,280 (2,047,822; 17,821,284)	10,334,147 (2,113,961; 18,013,439)	10,464,463 (2,181,038; 18,207,615)	11,628,845 (2,468,237; 20,197,282)	11,775,093 (2,544,248; 20,414,887)	11,922,983 (2,621,894; 20,634,780)	12,072,533 (2,700,633; 20,856,985)
Congo, Rep.	658,500 (-169,237; 1,334,183)	668,027 (-165,630; 1,349,913)	677,667 (-162,049; 1,365,816)	687,421 (-158,362; 1,381,893)	697,291 (-154,599; 1,397,306)	774,457 (-165,079; 1,547,033)	785,521 (-160,790; 1,564,220)	796,715 (-156,415; 1,581,590)	800,848 (-150,599; 1,584,906)
Costa Rica	446,713 (-345,013; 1,188,053)	449,823 (-338,215; 1,190,417)	452,920 (-332,088; 1,191,935)	456,006 (-326,266; 1,193,293)	459,079 (-320,473; 1,194,637)	432,560 (-294,564; 1,119,428)	435,414 (-289,665; 1,120,679)	438,256 (-283,907; 1,121,927)	441,087 (-278,711; 1,123,171)
Cote d'Ivoire	4,198,793 (376,981; 7,600,888)	4,251,675 (408,005; 7,680,375)	4,265,310 (435,414; 7,688,889)	4,278,891 (462,728; 7,697,378)	4,292,418 (489,946; 7,705,842)	4,661,051 (559,718; 8,350,573)	4,675,578 (588,975; 8,359,683)	4,690,047 (618,130; 8,368,766)	4,704,460 (647,183; 8,377,822)
Djibouti	118,505 (46,205; 175,692)	118,693 (46,669; 175,796)	118,880 (47,131; 175,924)	119,067 (47,592; 176,053)	119,252 (48,051; 176,180)	114,547 (46,522; 169,194)	114,724 (46,958; 169,430)	114,900 (47,393; 169,665)	115,075 (47,827; 169,774)
Dominican Republic	2,217,702 (618,580; 4,025,798)	2,222,766 (625,309; 4,029,199)	2,227,810 (632,011; 4,032,588)	2,232,834 (638,686; 4,035,966)	2,237,838 (645,333; 4,039,333)	2,142,543 (622,805; 3,861,937)	2,147,286 (629,104; 3,865,131)	2,152,009 (635,378; 3,868,316)	2,156,713 (641,627; 3,871,490)
Ecuador	3,560,220 (689,527; 6,720,970)	3,569,811 (704,714; 6,727,459)	3,579,364 (719,855; 6,733,926)	3,588,879 (734,962; 6,740,372)	3,598,356 (750,312; 6,746,796)	3,535,403 (750,253; 6,617,693)	3,544,616 (765,203; 6,623,947)	3,553,793 (780,107; 6,630,180)	3,562,933 (793,922; 6,636,393)
Egypt, Arab Rep.	42,751,284 (22,674,301; 65,364,686)	42,783,750 (22,700,140; 65,380,525)	42,816,085 (22,725,892; 65,396,330)	42,848,291 (22,751,556; 65,412,100)	42,880,369 (22,777,133; 65,428,172)	42,948,121 (22,821,648; 65,505,984)	42,979,970 (22,847,073; 65,529,123)	43,011,691 (22,872,412; 65,551,771)	43,043,285 (22,897,665; 65,574,174)
El Salvador	1,735,260 (1,046,300; 2,592,100)	1,753,505 (1,057,331; 2,618,472)	1,771,938 (1,068,476; 2,645,110)	1,790,561 (1,079,737; 2,672,019)	1,809,375 (1,091,116; 2,699,200)	1,698,573 (1,024,330; 2,533,071)	1,699,419 (1,024,873; 2,533,501)	1,700,262 (1,025,485; 2,533,930)	1,701,101 (1,026,494; 2,534,358)
Eritrea	461,168 (107,577; 794,457)	462,301 (109,539; 795,045)	463,429 (111,494; 795,631)	464,553 (113,440; 796,215)	465,673 (115,380; 796,798)	494,610 (124,303; 844,900)	495,787 (126,690; 845,502)	496,959 (129,212; 846,102)	498,127 (131,725; 846,701)
Eswatini	373,381 (138,114; 640,551)	373,999 (138,778; 641,033)	374,615 (139,438; 641,512)	375,229 (140,096; 641,989)	375,840 (140,751; 642,465)	378,581 (142,205; 646,579)	379,191 (142,859; 647,053)	379,798 (143,510; 647,542)	380,403 (144,158; 648,039)
Ethiopia	11,298,845 (1,015,053; 20,570,636)	11,447,282 (1,076,524; 20,779,311)	11,597,416 (1,128,205; 21,011,694)	11,749,266 (1,180,713; 21,249,361)	11,902,848 (1,234,060; 21,489,592)	12,497,407 (1,335,180; 22,524,026)	12,660,236 (1,392,240; 22,778,406)	12,824,920 (1,450,202; 23,035,529)	12,991,479 (1,509,078; 23,295,422)

Fiji	440,187 (260,665; 672,082)	440,331 (260,892; 672,337)	440,476 (261,119; 672,591)	440,619 (261,344; 672,843)	440,762 (261,569; 673,094)	431,096 (255,970; 658,364)	431,234 (256,189; 658,606)	431,372 (256,409; 658,848)	431,510 (256,628; 659,088)
Gabon	677,711 (-162,114; 1,379,409)	686,953 (-161,210; 1,394,489)	696,302 (-160,279; 1,409,729)	705,759 (-159,320; 1,425,131)	715,326 (-158,332; 1,440,695)	728,472 (-158,067; 1,463,394)	738,308 (-157,015; 1,479,367)	748,258 (-155,933; 1,495,509)	758,322 (-154,820; 1,511,823)
Gambia, The	275,733 (-105,469; 598,357)	277,113 (-103,858; 599,089)	278,487 (-102,250; 599,818)	279,856 (-100,646; 600,544)	281,219 (-99,046; 601,267)	302,946 (-104,474; 645,381)	304,396 (-102,767; 646,150)	305,840 (-101,064; 646,917)	307,278 (-99,365; 647,681)
Georgia	1,137,934 (688,762; 1,740,808)	1,138,314 (689,279; 1,741,130)	1,138,692 (689,793; 1,741,451)	1,139,069 (690,306; 1,741,770)	1,139,444 (690,817; 1,742,089)	1,042,249 (632,149; 1,593,256)	1,042,590 (632,613; 1,593,545)	1,042,928 (633,075; 1,593,832)	1,043,266 (633,535; 1,594,119)
Ghana	4,488,993 (564,043; 8,034,698)	4,502,936 (592,069; 8,043,395)	4,516,824 (619,996; 8,052,044)	4,530,658 (647,826; 8,060,647)	4,544,436 (675,558; 8,069,203)	4,757,798 (733,992; 8,431,501)	4,772,065 (762,737; 8,440,336)	4,786,277 (791,381; 8,449,122)	4,800,432 (819,925; 8,457,861)
Guatemala	5,718,054 (2,536,632; 9,444,348)	5,725,860 (2,542,515; 9,447,840)	5,733,635 (2,552,846; 9,451,325)	5,741,379 (2,563,288; 9,454,802)	5,749,093 (2,573,697; 9,458,272)	5,777,124 (2,593,206; 9,495,180)	5,784,803 (2,603,585; 9,498,648)	5,792,451 (2,613,931; 9,502,109)	5,800,070 (2,624,244; 9,505,564)
Guinea	1,031,445 (133,541; 1,850,639)	1,044,809 (140,057; 1,871,483)	1,058,326 (146,670; 1,892,552)	1,071,996 (153,380; 1,913,848)	1,085,821 (159,234; 1,935,372)	1,184,073 (177,027; 2,107,090)	1,199,297 (182,704; 2,130,765)	1,214,694 (188,467; 2,154,694)	1,230,265 (194,318; 2,178,881)
Guinea-Bissau	224,492 (7,723; 420,503)	227,498 (8,728; 425,090)	230,539 (9,588; 429,433)	233,615 (10,463; 433,792)	236,726 (11,352; 438,195)	251,628 (12,857; 464,335)	254,967 (13,821; 469,049)	258,345 (14,802; 473,810)	261,761 (15,798; 478,619)
Haiti	664,363 (274,123; 1,071,791)	672,567 (279,697; 1,083,714)	680,862 (285,346; 1,095,764)	689,249 (291,069; 1,107,942)	697,730 (296,868; 1,120,250)	693,372 (297,201; 1,111,949)	701,883 (303,045; 1,123,932)	710,489 (308,967; 1,135,992)	719,190 (314,967; 1,148,178)
Honduras	2,936,014 (1,475,061; 4,680,051)	2,939,381 (1,479,549; 4,683,003)	2,942,734 (1,482,953; 4,685,940)	2,946,074 (1,487,639; 4,688,864)	2,949,400 (1,495,058; 4,691,776)	2,920,576 (1,486,085; 4,643,578)	2,923,840 (1,493,345; 4,646,433)	2,927,091 (1,500,566; 4,649,276)	2,930,330 (1,504,401; 4,652,106)
India	168,246,492 (113,884,132; 226,337,097)	168,373,492 (114,030,168; 226,534,710)	168,499,989 (114,167,675; 226,622,958)	168,625,985 (114,278,083; 226,674,905)	168,751,483 (114,388,226; 226,726,704)	166,679,682 (113,008,674; 223,828,346)	166,802,569 (113,116,863; 223,879,179)	166,924,970 (113,224,792; 223,929,867)	167,046,888 (113,332,461; 223,980,411)
Indonesia	106,901,470 (63,115,393; 162,661,459)	106,930,246 (63,122,892; 162,693,321)	106,958,909 (63,130,385; 162,725,071)	106,987,458 (63,137,874; 162,756,710)	107,015,894 (63,145,357; 162,788,239)	105,253,647 (62,096,452; 160,096,109)	105,281,387 (62,117,109; 160,126,894)	105,309,017 (62,139,611; 160,157,571)	105,336,537 (62,162,059; 160,188,141)

Iraq	25,241,574 (14,144,495; 38,555,198)	25,488,333 (14,313,993; 38,925,807)	25,499,439 (14,351,288; 38,936,497)	25,510,500 (14,388,409; 38,947,148)	25,521,517 (14,425,356; 38,957,763)	26,966,143 (15,274,181; 41,156,418)	26,977,686 (15,312,838; 41,167,242)	26,989,182 (15,351,314; 41,177,875)	27,000,633 (15,371,434; 41,188,471)
Jamaica	531,705 (142,242; 971,668)	532,956 (143,905; 972,508)	534,202 (145,562; 973,346)	535,444 (147,212; 974,181)	536,680 (148,856; 975,014)	500,900 (140,138; 908,698)	502,042 (141,656; 909,468)	503,180 (143,168; 910,236)	504,313 (144,674; 911,001)
Jordan	4,324,465 (2,323,025; 6,705,065)	4,326,979 (2,328,590; 6,706,329)	4,329,482 (2,334,078; 6,707,587)	4,331,975 (2,335,939; 6,708,839)	4,334,458 (2,337,796; 6,710,087)	4,217,836 (2,275,399; 6,527,030)	4,220,232 (2,277,196; 6,528,233)	4,222,618 (2,278,988; 6,531,399)	4,224,995 (2,280,775; 6,536,154)
Kazakhstan	7,767,754 (4,184,641; 12,427,912)	7,773,000 (4,194,084; 12,430,459)	7,778,224 (4,205,769; 12,432,995)	7,783,428 (4,212,719; 12,435,518)	7,788,611 (4,219,644; 12,438,030)	7,363,651 (3,993,290; 11,753,961)	7,368,509 (3,999,786; 11,756,275)	7,373,347 (4,006,258; 11,758,570)	7,378,167 (4,010,986; 11,760,854)
Kenya	7,301,982 (1,034,137; 12,990,076)	7,324,264 (1,078,925; 13,003,974)	7,346,457 (1,123,556; 13,017,796)	7,368,563 (1,168,031; 13,031,543)	7,390,581 (1,212,351; 13,045,216)	7,848,984 (1,330,503; 13,827,759)	7,872,114 (1,377,105; 13,842,018)	7,895,152 (1,423,543; 13,856,193)	7,918,099 (1,469,819; 13,870,292)
Kiribati	58,938 (36,515; 86,095)	58,957 (36,527; 86,133)	58,977 (36,538; 86,170)	58,996 (36,549; 86,208)	59,016 (36,560; 86,245)	58,479 (36,227; 85,469)	58,498 (36,238; 85,506)	58,517 (36,249; 85,542)	58,536 (36,261; 85,578)
Kyrgyz Republic	1,121,521 (776,594; 1,521,812)	1,122,270 (777,339; 1,522,465)	1,123,016 (778,094; 1,523,115)	1,123,759 (778,846; 1,523,763)	1,124,499 (779,596; 1,524,409)	1,084,860 (752,342; 1,470,329)	1,085,568 (753,059; 1,470,947)	1,086,273 (753,774; 1,471,562)	1,086,976 (754,486; 1,472,175)
Lao PDR	1,110,879 (800,927; 1,475,619)	1,122,607 (810,045; 1,490,794)	1,134,455 (819,260; 1,506,123)	1,146,426 (828,573; 1,521,608)	1,158,520 (837,985; 1,537,250)	1,127,816 (816,426; 1,496,113)	1,137,862 (824,349; 1,509,042)	1,138,473 (825,440; 1,509,456)	1,139,081 (826,525; 1,509,912)
Lesotho	237,058 (36,260; 419,912)	237,772 (37,696; 420,355)	238,484 (39,126; 420,796)	239,192 (40,552; 421,235)	239,898 (41,973; 421,671)	238,199 (42,955; 417,890)	238,892 (44,352; 418,317)	239,583 (45,744; 418,742)	240,270 (47,131; 419,165)
Liberia	555,890 (99,628; 980,110)	562,964 (103,203; 990,697)	570,119 (106,829; 1,001,396)	577,353 (110,507; 1,012,208)	584,670 (114,237; 1,023,133)	635,936 (126,766; 1,110,798)	643,972 (130,888; 1,122,781)	652,099 (135,069; 1,134,891)	660,317 (139,324; 1,147,128)
Madagascar	3,150,785 (591,931; 5,536,283)	3,190,771 (612,266; 5,596,047)	3,231,208 (632,892; 5,656,440)	3,272,100 (653,812; 5,717,468)	3,313,452 (675,031; 5,779,139)	3,622,431 (752,014; 6,306,581)	3,668,086 (775,577; 6,374,571)	3,714,254 (799,553; 6,443,276)	3,760,941 (824,061; 6,512,704)
Malawi	2,823,710 (436,007; 5,023,298)	2,831,656 (449,513; 5,029,389)	2,839,571 (462,966; 5,035,460)	2,847,454 (476,366; 5,041,511)	2,855,305 (489,715; 5,047,544)	3,124,995 (549,020; 5,514,866)	3,133,497 (562,007; 5,519,306)	3,141,966 (571,801; 5,523,734)	3,150,400 (581,571; 5,528,149)

Malaysia	12,604,743 (6,900,187; 19,662,392)	12,611,596 (6,907,959; 19,667,724)	12,618,422 (6,915,698; 19,673,033)	12,625,221 (6,923,403; 19,678,317)	12,631,994 (6,931,074; 19,683,578)	12,209,359 (6,702,981; 19,019,921)	12,215,849 (6,710,328; 19,024,957)	12,222,314 (6,717,642; 19,029,970)	12,228,752 (6,724,925; 19,034,960)
Mali	2,725,693 (40,513; 5,146,239)	2,762,437 (54,355; 5,202,468)	2,799,607 (64,527; 5,259,294)	2,837,205 (74,875; 5,316,722)	2,875,236 (85,399; 5,374,568)	3,221,644 (106,260; 6,002,922)	3,264,669 (118,297; 6,063,871)	3,308,189 (130,537; 6,125,438)	3,352,210 (142,984; 6,187,628)
Mauritania	702,409 (133,054; 1,226,829)	711,484 (138,502; 1,240,371)	720,661 (144,034; 1,254,053)	729,943 (149,648; 1,267,878)	739,330 (155,346; 1,281,847)	802,358 (172,646; 1,388,613)	804,599 (176,979; 1,389,994)	806,831 (179,794; 1,391,368)	809,055 (182,602; 1,392,734)
Mexico	13,843,824 (-9,300,944; 35,606,580)	14,073,470 (-9,222,033; 36,001,025)	14,305,959 (-9,141,469; 36,399,666)	14,541,321 (-9,059,231; 36,802,544)	14,685,112 (-8,897,559; 36,971,848)	14,238,829 (-8,419,574; 35,662,677)	14,327,024 (-8,264,684; 35,706,129)	14,414,868 (-8,101,747; 35,765,084)	14,502,362 (-7,939,609; 35,823,845)
Mongolia	1,353,796 (848,348; 1,972,238)	1,354,171 (848,692; 1,972,477)	1,354,545 (849,035; 1,972,715)	1,354,917 (849,376; 1,972,952)	1,355,288 (849,716; 1,973,188)	1,274,968 (799,459; 1,855,965)	1,275,314 (799,776; 1,856,186)	1,275,659 (800,092; 1,856,407)	1,276,002 (800,406; 1,856,626)
Morocco	11,155,782 (5,566,781; 17,255,737)	11,165,708 (5,596,909; 17,264,162)	11,175,594 (5,626,891; 17,272,554)	11,185,440 (5,656,728; 17,280,911)	11,195,247 (5,686,421; 17,289,234)	10,799,046 (5,508,876; 16,670,818)	10,808,423 (5,537,218; 16,678,774)	10,817,761 (5,565,422; 16,686,698)	10,827,063 (5,593,491; 16,694,590)
Mozambique	4,511,687 (394,714; 8,223,391)	4,525,744 (415,208; 8,224,568)	4,539,745 (431,272; 8,230,648)	4,553,690 (447,297; 8,241,356)	4,567,580 (463,285; 8,252,031)	5,005,896 (523,636; 9,028,233)	5,020,953 (541,022; 9,039,823)	5,035,950 (558,366; 9,051,377)	5,050,888 (575,668; 9,062,893)
Myanmar	6,971,043 (5,199,743; 9,063,051)	6,973,843 (5,204,825; 9,063,868)	6,976,632 (5,209,890; 9,064,684)	6,979,409 (5,214,939; 9,065,498)	6,982,176 (5,219,972; 9,066,311)	6,930,501 (5,184,273; 8,996,466)	6,933,225 (5,188,028; 8,997,270)	6,935,938 (5,189,824; 8,998,072)	6,938,640 (5,191,617; 8,998,873)
Namibia	1,167,087 (356,409; 1,978,591)	1,169,098 (357,996; 1,979,544)	1,171,101 (359,606; 1,980,492)	1,173,096 (361,212; 1,981,436)	1,175,083 (363,080; 1,982,375)	1,188,538 (370,443; 2,002,647)	1,190,529 (374,265; 2,003,586)	1,192,511 (378,093; 2,004,521)	1,194,486 (381,905; 2,005,452)
Nepal	4,203,543 (2,975,500; 5,548,310)	4,205,888 (2,979,487; 5,549,671)	4,208,224 (2,983,459; 5,551,344)	4,210,551 (2,987,417; 5,553,739)	4,212,868 (2,991,362; 5,556,124)	3,900,718 (2,771,630; 5,143,827)	3,902,846 (2,774,182; 5,146,016)	3,904,965 (2,776,727; 5,148,195)	3,907,076 (2,779,265; 5,150,366)
Nicaragua	683,860 (220,281; 1,087,435)	685,756 (223,191; 1,088,428)	687,645 (226,090; 1,089,416)	689,526 (228,979; 1,090,400)	691,399 (231,857; 1,091,380)	669,217 (226,583; 1,054,463)	671,011 (229,342; 1,055,400)	672,798 (232,091; 1,056,333)	674,578 (234,846; 1,057,263)
Niger	4,526,124 (889,203; 7,922,340)	4,583,375 (918,496; 8,007,785)	4,641,270 (948,205; 8,094,129)	4,699,817 (978,337; 8,181,381)	4,759,022 (1,008,896; 8,269,551)	5,545,231 (1,196,626; 9,618,526)	5,559,306 (1,220,862; 9,625,873)	5,573,325 (1,245,130; 9,633,198)	5,587,289 (1,269,303; 9,640,503)

Nigeria	20,539,785 (-3,482,863; 40,527,339)	20,830,586 (-3,345,891; 40,976,700)	21,124,808 (-3,206,436; 41,430,828)	21,422,486 (-3,064,467; 41,889,772)	21,723,660 (-2,919,951; 42,353,584)	23,865,476 (-3,004,104; 46,393,584)	24,199,464 (-2,841,910; 46,906,786)	24,537,363 (-2,676,849; 47,424,695)	24,879,216 (-2,508,884; 47,948,071)
Pakistan	37,114,691 (18,736,154; 53,304,476)	37,506,735 (19,049,175; 53,844,301)	37,552,162 (19,202,646; 53,886,410)	37,597,408 (19,338,835; 53,924,185)	37,642,473 (19,463,876; 53,954,249)	37,685,632 (19,587,872; 53,981,729)	37,730,336 (19,712,178; 54,011,567)	37,774,861 (19,835,906; 54,041,004)	37,819,208 (19,958,709; 54,094,219)
Papua New Guinea	688,445 (471,924; 945,931)	695,893 (478,226; 955,839)	703,419 (483,455; 965,730)	711,024 (488,554; 975,638)	718,709 (494,073; 985,647)	760,462 (523,150; 1,042,346)	768,675 (529,175; 1,053,037)	776,974 (535,287; 1,063,837)	785,359 (541,467; 1,074,747)
Paraguay	1,856,915 (790,796; 3,086,873)	1,859,650 (792,463; 3,089,328)	1,862,375 (794,124; 3,091,776)	1,865,089 (795,779; 3,094,218)	1,867,792 (797,428; 3,096,652)	1,829,514 (781,614; 3,031,199)	1,832,137 (783,282; 3,033,567)	1,834,750 (786,207; 3,035,929)	1,837,352 (789,681; 3,038,285)
Peru	6,185,780 (1,440,837; 11,519,369)	6,201,550 (1,458,141; 11,530,040)	6,217,256 (1,475,389; 11,540,677)	6,232,900 (1,492,580; 11,551,278)	6,248,482 (1,509,716; 11,561,844)	5,990,238 (1,472,386; 11,066,613)	6,005,021 (1,500,096; 11,076,650)	6,019,745 (1,512,180; 11,086,654)	6,034,410 (1,524,242; 11,096,625)
Philippines	35,493,393 (22,204,506; 51,816,233)	35,859,049 (22,436,377; 52,354,945)	36,228,426 (22,670,624; 52,892,187)	36,601,562 (22,907,270; 53,428,019)	36,978,493 (23,146,341; 53,969,258)	37,719,616 (23,613,454; 55,041,802)	38,107,965 (23,859,801; 55,599,343)	38,500,266 (24,108,672; 56,162,509)	38,896,556 (24,360,091; 56,731,356)
Rwanda	1,623,163 (60,869; 3,036,597)	1,628,591 (67,302; 3,038,470)	1,633,997 (72,758; 3,038,925)	1,639,381 (78,201; 3,039,379)	1,644,745 (83,630; 3,039,833)	1,707,234 (92,131; 3,145,579)	1,712,739 (97,722; 3,146,047)	1,718,222 (103,300; 3,146,515)	1,723,684 (108,865; 3,146,982)
Senegal	2,862,156 (453,897; 5,058,952)	2,870,726 (471,122; 5,064,273)	2,879,263 (488,287; 5,069,565)	2,887,765 (505,392; 5,074,828)	2,896,234 (522,437; 5,080,063)	3,119,323 (579,285; 5,461,070)	3,128,345 (597,462; 5,466,630)	3,137,332 (615,575; 5,472,160)	3,146,283 (633,625; 5,477,660)
Sierra Leone	957,008 (24,137; 1,799,189)	960,260 (28,424; 1,800,822)	963,499 (31,693; 1,802,451)	966,726 (34,954; 1,803,298)	969,939 (38,207; 1,803,570)	998,257 (42,523; 1,850,398)	1,001,527 (45,844; 1,850,676)	1,004,785 (49,158; 1,850,954)	1,008,029 (52,464; 1,851,232)
Solomon Islands	151,798 (96,838; 212,926)	151,963 (97,133; 213,101)	152,128 (97,426; 213,320)	152,292 (97,717; 213,539)	152,455 (97,927; 213,756)	162,152 (104,186; 227,339)	162,324 (104,328; 227,568)	162,496 (104,472; 227,796)	162,667 (104,659; 228,020)
Somalia	1,684,508 (841,855; 2,606,868)	1,703,627 (856,326; 2,634,090)	1,722,952 (871,822; 2,661,592)	1,742,483 (887,505; 2,689,377)	1,762,223 (903,382; 2,717,447)	1,997,075 (1,030,424; 3,076,905)	2,019,672 (1,048,751; 3,109,267)	2,042,510 (1,064,710; 3,142,110)	2,065,592 (1,078,027; 3,175,291)
South Africa	13,864,436 (-4,359,113; 28,996,187)	13,918,682 (-4,304,093; 29,024,536)	13,972,714 (-4,249,208; 29,052,807)	14,026,531 (-4,194,374; 29,080,998)	14,080,135 (-4,139,236; 29,109,111)	13,962,687 (-4,034,867; 28,784,949)	14,015,224 (-3,980,532; 28,812,567)	14,067,552 (-3,925,216; 28,840,108)	14,119,673 (-3,870,045; 28,867,572)

Sri Lanka	5,993,633 (3,632,005; 8,844,340)	5,995,972 (3,633,797; 8,846,485)	5,998,302 (3,635,263; 8,848,606)	6,000,623 (3,636,727; 8,850,717)	6,002,934 (3,638,187; 8,852,813)	5,775,136 (3,500,186; 8,515,584)	5,777,341 (3,501,586; 8,517,555)	5,779,538 (3,502,982; 8,519,517)	5,781,725 (3,504,376; 8,521,470)
Sudan	6,135,415 (1,044,196; 10,658,977)	6,150,332 (1,063,319; 10,663,596)	6,165,190 (1,087,250; 10,668,194)	6,179,988 (1,128,161; 10,672,773)	6,194,728 (1,152,450; 10,677,333)	6,698,092 (1,269,848; 11,522,541)	6,713,864 (1,306,512; 11,527,417)	6,729,573 (1,352,920; 11,532,872)	6,745,220 (1,399,405; 11,542,957)
Suriname	103,824 (25,764; 191,550)	105,119 (26,612; 193,640)	106,429 (27,388; 195,751)	107,753 (28,008; 197,885)	109,093 (28,636; 200,041)	107,807 (28,572; 197,385)	109,144 (29,202; 199,533)	110,495 (29,839; 201,704)	111,862 (30,444; 203,898)
Tajikistan	1,686,841 (1,099,387; 2,401,076)	1,688,174 (1,101,992; 2,401,448)	1,689,502 (1,104,588; 2,402,097)	1,690,825 (1,107,175; 2,403,015)	1,692,142 (1,109,752; 2,403,929)	1,691,272 (1,110,886; 2,401,742)	1,692,577 (1,113,442; 2,402,649)	1,693,877 (1,115,988; 2,403,553)	1,695,172 (1,117,180; 2,404,453)
Tanzania	11,420,162 (3,463,080; 19,001,788)	11,448,184 (3,497,579; 19,028,343)	11,476,095 (3,531,989; 19,047,121)	11,503,895 (3,566,309; 19,059,232)	11,531,585 (3,600,540; 19,071,282)	12,832,328 (4,035,017; 21,185,163)	12,862,825 (4,072,820; 21,198,131)	12,893,200 (4,110,525; 21,210,779)	12,923,455 (4,148,483; 21,223,364)
Thailand	15,758,894 (8,792,924; 24,414,562)	15,766,263 (8,798,859; 24,421,554)	15,773,602 (8,804,780; 24,428,519)	15,780,913 (8,810,688; 24,435,457)	15,788,195 (8,816,580; 24,442,369)	14,761,514 (8,244,962; 22,848,863)	14,768,266 (8,250,442; 22,855,273)	14,774,991 (8,255,910; 22,861,659)	14,781,689 (8,261,365; 22,868,020)
Timor-Leste	681,224 (429,564; 986,997)	681,388 (429,666; 987,120)	681,551 (429,768; 987,242)	681,713 (429,869; 987,364)	681,875 (429,970; 987,485)	690,995 (435,723; 1,000,580)	691,157 (435,830; 1,000,702)	691,319 (435,938; 1,000,825)	691,481 (436,045; 1,000,947)
Togo	1,056,595 (139,861; 1,893,868)	1,069,566 (146,446; 1,913,928)	1,072,661 (151,706; 1,916,303)	1,075,745 (156,945; 1,918,671)	1,078,816 (161,691; 1,921,031)	1,170,410 (178,738; 2,080,783)	1,173,706 (182,544; 2,083,320)	1,176,989 (186,341; 2,085,849)	1,180,259 (190,129; 2,088,369)
Tunisia	2,836,982 (1,305,200; 4,484,633)	2,840,097 (1,320,082; 4,487,271)	2,843,201 (1,334,899; 4,489,898)	2,846,292 (1,349,654; 4,492,515)	2,849,370 (1,364,346; 4,494,431)	2,584,548 (1,249,468; 4,073,244)	2,587,315 (1,257,246; 4,074,153)	2,590,071 (1,258,817; 4,075,059)	2,592,816 (1,260,384; 4,075,963)
Turkey	27,918,739 (13,924,798; 45,968,692)	27,943,501 (13,957,894; 45,993,597)	27,968,164 (13,990,870; 46,018,397)	27,992,728 (14,023,726; 46,043,092)	28,017,195 (14,056,583; 46,067,556)	26,819,546 (13,475,624; 44,083,249)	26,842,760 (13,507,119; 44,106,413)	26,865,881 (13,538,500; 44,129,479)	26,888,910 (13,576,343; 44,152,449)
Uganda	6,539,864 (458,348; 12,030,887)	6,560,762 (479,409; 12,032,639)	6,581,578 (500,420; 12,034,389)	6,602,311 (521,382; 12,036,137)	6,622,962 (544,874; 12,037,883)	7,028,854 (601,563; 12,747,211)	7,050,529 (626,590; 12,763,900)	7,072,119 (651,557; 12,780,536)	7,093,623 (676,464; 12,797,120)
Ukraine	6,140,817 (3,774,184; 9,205,922)	6,204,699 (3,815,115; 9,300,939)	6,269,234 (3,856,472; 9,396,926)	6,334,430 (3,898,260; 9,493,891)	6,400,292 (3,940,483; 9,591,827)	5,976,063 (3,680,016; 8,955,311)	6,028,399 (3,712,470; 9,032,993)	6,030,741 (3,714,139; 9,035,761)	6,033,074 (3,715,802; 9,038,519)

Uzbekistan	4,498,018 (2,794,906; 6,332,696)	4,503,032 (2,801,845; 6,337,117)	4,508,026 (2,808,756; 6,341,520)	4,513,000 (2,815,637; 6,345,907)	4,517,955 (2,822,606; 6,350,276)	4,224,980 (2,640,834; 5,936,069)	4,229,571 (2,649,458; 5,940,119)	4,234,144 (2,655,627; 5,944,154)	4,238,699 (2,661,921; 5,948,173)
Vanuatu	148,177 (87,489; 221,777)	148,269 (87,614; 221,807)	148,360 (87,739; 221,836)	148,451 (87,864; 221,866)	148,542 (87,987; 221,895)	157,662 (93,460; 235,407)	157,757 (93,586; 235,438)	157,852 (93,711; 235,469)	157,946 (93,836; 235,500)
Vietnam	26,940,863 (16,917,240; 39,122,695)	26,947,579 (16,923,360; 39,126,970)	26,954,269 (16,929,455; 39,131,231)	26,960,932 (16,935,526; 39,135,480)	26,967,569 (16,941,617; 39,139,715)	25,511,189 (16,028,553; 37,020,842)	25,517,416 (16,034,323; 37,024,618)	25,523,618 (16,040,069; 37,028,384)	25,529,796 (16,045,792; 37,032,138)
Yemen, Rep.	2,425,677 (-837,928; 5,005,569)	2,460,345 (-829,215; 5,061,497)	2,495,421 (-820,300; 5,118,026)	2,530,911 (-811,179; 5,175,163)	2,566,818 (-801,850; 5,232,913)	2,622,238 (-798,120; 5,330,086)	2,659,264 (-788,294; 5,389,515)	2,696,724 (-777,918; 5,449,581)	2,734,625 (-767,188; 5,510,292)
Zambia	3,442,793 (990,727; 5,765,314)	3,451,471 (1,001,408; 5,773,540)	3,460,114 (1,012,061; 5,781,721)	3,468,723 (1,022,686; 5,789,860)	3,477,297 (1,033,283; 5,797,955)	3,861,721 (1,156,413; 6,428,952)	3,871,145 (1,168,092; 6,433,047)	3,880,532 (1,179,911; 6,437,121)	3,889,881 (1,191,734; 6,441,143)
Zimbabwe	2,173,156 (668,727; 3,609,106)	2,178,446 (675,241; 3,613,057)	2,183,715 (681,738; 3,615,354)	2,188,963 (688,217; 3,617,640)	2,194,191 (694,680; 3,619,914)	2,273,329 (724,694; 3,743,915)	2,278,689 (731,340; 3,746,857)	2,284,028 (737,968; 3,751,334)	2,289,346 (744,591; 3,755,799)

Table S22. Net costs of vaccination estimates assuming the vaccine is effective against Shigella moderate-to-severe diarrhoea (MSD) only from companion cost effectiveness analysis. Costs were aggregated from 2025 to 2044 and are reported in 2019 US$. Estimates in parenthesis represent 95% uncertainty estimates from the results of 1000 model simulations.

Country	2025	2026	2027	2028	2029	2030	2031	2032	2033	2034
Afghanistan	6,532,586 (4,676,489; 9,051,384)	6,600,015 (4,726,499; 9,144,409)	6,668,131 (4,777,032; 9,238,378)	6,736,940 (4,828,094; 9,333,300)	6,806,451 (4,879,691; 9,429,184)	6,969,119 (4,998,130; 9,654,109)	7,041,005 (5,051,520; 9,753,264)	7,113,624 (5,105,467; 9,853,425)	7,186,982 (5,159,979; 9,954,602)	7,261,087 (5,215,061 10,056,804
Algeria	15,641,600 (9,244,777; 24,113,460)	15,650,695 (9,256,600; 24,119,920)	15,659,753 (9,271,041; 24,126,349)	15,668,776 (9,279,894; 24,132,748)	15,677,762 (9,291,334; 24,139,116)	14,208,898 (8,426,338; 21,870,757)	14,216,973 (8,436,621; 21,876,470)	14,225,017 (8,446,867; 21,882,157)	14,233,028 (8,457,073; 21,887,816)	14,241,008 (8,467,241 21,893,448
Angola	13,984,236 (7,440,883; 22,271,862)	14,135,973 (7,528,350; 22,502,063)	14,289,298 (7,616,906; 22,734,611)	14,444,229 (7,710,801; 22,969,531)	14,600,781 (7,809,704; 23,206,845)	16,656,334 (8,926,567; 26,460,792)	16,836,728 (9,040,753; 26,734,102)	17,019,009 (9,156,256; 27,010,199)	17,203,196 (9,273,056; 27,289,109)	17,389,308 (9,391,170 27,570,862
Argentina	11,132,970 (6,133,091; 17,462,312)	11,252,938 (6,202,488; 17,642,471)	11,374,157 (6,272,640; 17,824,467)	11,398,377 (6,288,409; 17,854,399)	11,407,009 (6,293,621; 17,859,939)	11,303,803 (6,237,130; 17,690,484)	11,312,283 (6,242,267; 17,695,932)	11,320,729 (6,247,391; 17,701,360)	11,329,142 (6,252,503; 17,706,769)	11,337,521 (6,257,603 17,712,159

Armenia	885,743 (558,823; 1,326,163)	885,862 (558,961; 1,326,259)	885,981 (559,098; 1,326,356)	886,100 (559,235; 1,326,451)	886,218 (559,372; 1,326,547)	778,879 (491,675; 1,165,805)	778,982 (491,794; 1,165,888)	779,084 (491,913; 1,165,971)	779,186 (492,031; 1,166,054)	779,288 (492,149; 1,166,136)
Azerbaijan	3,520,409 (2,203,035; 5,281,430)	3,520,973 (2,203,481; 5,282,294)	3,521,535 (2,203,925; 5,283,153)	3,522,095 (2,204,367; 5,284,008)	3,522,653 (2,205,258; 5,284,859)	3,186,770 (1,995,988; 4,780,962)	3,187,270 (1,997,295; 4,781,724)	3,187,769 (1,998,594; 4,782,481)	3,188,265 (1,999,887; 4,783,234)	3,188,760 (2,001,172 4,783,984)
Bangladesh	24,484,002 (18,877,786; 31,427,921)	24,489,179 (18,885,527; 31,429,948)	24,494,336 (18,893,233; 31,431,968)	24,499,473 (18,900,906; 31,433,981)	24,504,589 (18,908,544; 31,435,986)	23,061,687 (17,798,609; 29,580,672)	23,066,462 (17,805,732; 29,582,546)	23,071,219 (17,811,230; 29,584,413)	23,075,957 (17,816,793; 29,586,273)	23,080,677 (17,826,360 29,588,127
Belarus	2,635,131 (1,636,431; 3,967,371)	2,635,684 (1,637,242; 3,967,734)	2,636,234 (1,638,050; 3,968,442)	2,636,783 (1,638,855; 3,969,279)	2,637,329 (1,639,657; 3,970,113)	2,341,058 (1,455,871; 3,524,128)	2,341,538 (1,456,577; 3,524,860)	2,342,018 (1,457,282; 3,525,588)	2,342,495 (1,457,983; 3,526,313)	2,342,970 (1,458,682 3,527,033)
Benin	2,787,159 (1,970,266; 3,828,961)	2,816,893 (1,991,774; 3,869,259)	2,846,935 (2,013,510; 3,909,969)	2,877,289 (2,035,477; 3,951,095)	2,907,958 (2,057,676; 3,992,642)	3,203,159 (2,265,957; 4,397,331)	3,237,282 (2,289,331; 4,443,543)	3,271,758 (2,313,076; 4,490,229)	3,306,592 (2,342,187; 4,537,391)	3,341,787 (2,367,679 4,585,035)
Bhutan	246,948 (158,751; 356,406)	246,986 (158,826; 356,450)	247,024 (158,902; 356,494)	247,062 (158,977; 356,537)	247,100 (159,052; 356,580)	228,320 (147,010; 329,469)	228,354 (147,078; 329,509)	228,388 (147,146; 329,548)	228,422 (147,214; 329,587)	228,456 (147,281; 329,626)
Bolivia	3,596,469 (2,229,626; 5,336,804)	3,633,855 (2,252,902; 5,391,125)	3,671,622 (2,276,417; 5,445,996)	3,709,776 (2,300,174; 5,501,420)	3,748,320 (2,324,175; 5,557,404)	3,807,696 (2,361,096; 5,644,240)	3,847,244 (2,385,725; 5,701,657)	3,887,197 (2,410,606; 5,759,654)	3,927,557 (2,435,744; 5,818,236)	3,968,331 (2,461,140 5,877,410)
Botswana	1,089,851 (488,753; 1,770,292)	1,091,054 (490,029; 1,771,718)	1,092,253 (491,302; 1,773,133)	1,093,447 (492,569; 1,774,543)	1,094,636 (492,961; 1,775,947)	1,098,480 (494,460; 1,781,661)	1,099,662 (494,763; 1,782,363)	1,100,840 (495,065; 1,782,877)	1,102,013 (495,367; 1,783,391)	1,103,182 (495,676; 1,783,902)
Brazil	39,061,644 (24,293,983; 58,624,165)	39,462,524 (24,545,053; 59,217,714)	39,867,474 (24,798,689; 59,817,249)	40,276,536 (25,054,916; 60,422,832)	40,689,750 (25,313,761; 61,034,522)	38,521,464 (23,966,532; 57,774,365)	38,916,587 (24,214,077; 58,360,398)	39,315,721 (24,464,149; 58,962,870)	39,718,906 (24,716,775; 59,571,452)	40,126,183 (24,971,982 60,186,207
Bulgaria	1,409,155 (866,137; 2,130,009)	1,409,501 (866,542; 2,130,249)	1,409,845 (866,944; 2,130,488)	1,410,188 (867,346; 2,130,727)	1,410,529 (867,746; 2,130,965)	1,328,255 (817,310; 2,006,397)	1,328,574 (817,683; 2,006,608)	1,328,892 (818,056; 2,006,818)	1,329,208 (818,427; 2,007,028)	1,329,523 (818,797; 2,007,237)
Burkina Faso	4,637,161 (3,007,205; 6,565,666)	4,640,645 (3,014,366; 6,566,264)	4,644,115 (3,021,505; 6,566,861)	4,647,572 (3,028,620; 6,567,456)	4,651,014 (3,035,711; 6,568,050)	5,092,191 (3,328,614; 7,186,419)	5,095,927 (3,331,747; 7,187,065)	5,099,649 (3,334,820; 7,187,710)	5,103,356 (3,337,887; 7,188,353)	5,107,049 (3,340,948 7,188,995)
Burundi	2,859,560 (1,931,700; 3,997,753)	2,861,336 (1,934,130; 3,998,727)	2,863,104 (1,936,554; 3,999,700)	2,864,865 (1,938,978; 4,000,671)	2,866,619 (1,940,757; 4,002,021)	3,085,061 (2,088,905; 4,305,826)	3,086,933 (2,090,433; 4,307,286)	3,088,797 (2,092,004; 4,308,741)	3,090,654 (2,093,649; 4,310,190)	3,092,504 (2,095,291 4,311,633)

Cabo Verde	180,393 (108,704; 275,020)	180,484 (108,821; 275,085)	180,576 (108,937; 275,150)	180,666 (109,053; 275,215)	180,757 (109,169; 275,279)	169,781 (102,599; 258,495)	169,866 (102,707; 258,554)	169,949 (102,816; 258,614)	170,033 (102,924; 258,673)	170,116 (103,031; 258,732)
Cambodia	2,925,416 (2,267,231; 3,763,480)	2,925,926 (2,267,919; 3,764,121)	2,926,434 (2,268,617; 3,764,759)	2,926,939 (2,269,323; 3,765,394)	2,927,442 (2,270,027; 3,766,025)	2,836,059 (2,199,469; 3,648,448)	2,836,543 (2,200,147; 3,649,053)	2,837,025 (2,200,823; 3,649,656)	2,837,505 (2,201,498; 3,650,255)	2,837,983 (2,202,633 3,650,852)
Cameroon	5,043,139 (3,453,043; 6,983,362)	5,097,590 (3,499,709; 7,055,017)	5,152,609 (3,546,895; 7,127,403)	5,208,203 (3,590,489; 7,200,529)	5,264,377 (3,628,838; 7,274,363)	5,706,185 (3,932,985; 7,883,329)	5,767,688 (3,974,979; 7,967,217)	5,829,832 (4,017,449; 8,051,964)	5,892,624 (4,060,408; 8,137,579)	5,956,072 (4,103,818 8,224,070)
Central African Republic	477,833 (310,269; 676,284)	482,973 (314,115; 683,109)	488,166 (318,004; 690,003)	493,414 (321,937; 696,966)	498,716 (325,915; 703,999)	544,492 (355,959; 768,122)	550,339 (359,876; 775,873)	556,248 (363,833; 783,702)	562,217 (367,833; 791,610)	568,249 (371,874; 799,597)
Chad	2,271,541 (1,454,880; 3,230,684)	2,296,084 (1,471,511; 3,264,554)	2,320,883 (1,488,320; 3,298,772)	2,345,941 (1,505,309; 3,333,342)	2,371,261 (1,522,481; 3,368,268)	2,599,733 (1,670,180; 3,691,656)	2,627,773 (1,689,207; 3,730,321)	2,656,105 (1,708,437; 3,769,159)	2,684,732 (1,728,090; 3,808,215)	2,713,659 (1,748,524 3,847,671)
China	401,947,550 (246,557,594; 605,345,895)	402,017,380 (246,580,634; 605,408,668)	402,086,931 (246,603,629; 605,471,213)	402,156,205 (246,626,579; 605,533,530)	402,225,202 (246,649,483; 605,595,621)	374,318,224 (229,518,637; 563,539,791)	374,381,914 (229,539,865; 563,597,145)	374,445,350 (229,561,051; 563,654,291)	374,508,533 (229,582,196; 563,711,229)	374,571,46 (229,603,29 563,767,960
Colombia	10,432,647 (5,251,000; 16,870,449)	10,443,882 (5,271,179; 16,875,628)	10,455,073 (5,283,922; 16,880,791)	10,466,218 (5,294,716; 16,885,938)	10,477,320 (5,305,481; 16,891,069)	9,828,573 (4,981,783; 15,833,276)	9,838,893 (4,991,817; 15,838,054)	9,849,173 (5,002,455; 15,842,818)	9,859,411 (5,011,595; 15,847,566)	9,869,608 (5,021,326 15,852,300
Comoros	193,880 (136,999; 266,406)	194,008 (137,124; 266,545)	194,136 (137,248; 266,683)	194,264 (137,373; 266,820)	194,391 (137,496; 266,956)	202,755 (143,390; 278,402)	202,886 (143,434; 278,543)	203,017 (143,485; 278,683)	203,147 (143,747; 278,822)	203,277 (143,967; 278,961)
Congo, Dem. Rep.	14,530,298 (9,772,302; 20,344,444)	14,684,962 (9,882,898; 20,555,181)	14,841,228 (9,994,683; 20,768,069)	14,999,114 (10,107,280; 20,983,131)	15,158,635 (10,216,101; 21,200,389)	16,812,720 (11,332,344; 23,507,225)	16,991,431 (11,454,305; 23,750,547)	17,171,993 (11,577,554; 23,996,354)	17,354,425 (11,702,104; 24,244,669)	17,538,745 (11,828,142 24,495,518
Congo, Rep.	1,144,416 (753,758; 1,606,148)	1,156,984 (763,769; 1,623,426)	1,169,684 (774,122; 1,640,882)	1,182,519 (783,905; 1,658,519)	1,195,488 (792,873; 1,676,337)	1,323,389 (878,111; 1,855,273)	1,337,891 (888,414; 1,875,189)	1,352,545 (899,311; 1,895,311)	1,355,180 (901,571; 1,898,586)	1,356,447 (902,824; 1,899,943)
Costa Rica	926,720 (419,138; 1,541,319)	928,045 (420,008; 1,542,227)	929,364 (420,883; 1,543,132)	930,679 (421,756; 1,544,034)	931,988 (422,626; 1,544,933)	873,555 (396,682; 1,446,886)	874,770 (398,836; 1,447,721)	875,981 (400,980; 1,448,554)	877,187 (403,112; 1,449,384)	878,388 (405,233; 1,450,212)
Cote d'Ivoire	6,506,629 (4,492,072; 9,006,938)	6,572,421 (4,545,486; 9,093,302)	6,577,459 (4,550,174; 9,095,590)	6,582,477 (4,553,165; 9,097,874)	6,587,475 (4,556,151; 9,100,155)	7,136,214 (4,935,178; 9,853,221)	7,141,581 (4,938,468; 9,855,682)	7,146,927 (4,941,773; 9,858,140)	7,152,252 (4,945,073; 9,860,594)	7,157,556 (4,948,365 9,863,951)

Djibouti	149,766 (105,095; 200,229)	149,837 (105,164; 200,281)	149,908 (105,242; 200,318)	149,979 (105,400; 200,346)	150,050 (105,556; 200,374)	143,973 (101,383; 192,197)	144,040 (101,532; 192,224)	144,107 (101,680; 192,250)	144,173 (101,827; 192,281)	144,240 (101,908; 192,354)
Dominican Republic	3,018,046 (1,691,277; 4,688,253)	3,020,133 (1,693,424; 4,689,591)	3,022,212 (1,695,578; 4,690,924)	3,024,283 (1,697,731; 4,692,252)	3,026,346 (1,699,873; 4,693,576)	2,892,998 (1,625,911; 4,484,983)	2,894,953 (1,627,981; 4,486,239)	2,896,900 (1,630,045; 4,487,490)	2,898,839 (1,632,106; 4,488,737)	2,900,770 (1,634,159 4,489,979)
Ecuador	5,065,911 (2,769,967; 7,965,530)	5,069,903 (2,773,717; 7,968,087)	5,073,878 (2,777,457; 7,970,636)	5,077,837 (2,781,688; 7,973,176)	5,081,781 (2,786,398; 7,975,707)	4,983,662 (2,735,076; 7,818,142)	4,987,496 (2,739,637; 7,820,605)	4,991,314 (2,744,171; 7,823,060)	4,995,117 (2,748,679; 7,825,505)	4,998,906 (2,751,109 7,827,943)
Egypt, Arab Rep.	47,978,209 (30,276,621; 69,812,483)	47,991,151 (30,310,970; 69,816,279)	48,004,042 (30,345,149; 69,820,062)	48,016,881 (30,379,160; 69,823,831)	48,029,668 (30,413,004; 69,827,587)	48,082,488 (30,472,083; 69,889,592)	48,095,184 (30,505,622; 69,893,324)	48,107,829 (30,538,995; 69,897,044)	48,120,424 (30,572,205; 69,900,751)	48,132,968 (30,605,250 69,904,444
El Salvador	1,885,857 (1,205,384; 2,710,028)	1,905,042 (1,217,698; 2,737,397)	1,924,421 (1,230,137; 2,765,042)	1,943,997 (1,242,702; 2,792,964)	1,963,769 (1,255,394; 2,821,168)	1,842,902 (1,178,175; 2,647,338)	1,843,212 (1,178,421; 2,647,594)	1,843,520 (1,178,667; 2,647,849)	1,843,827 (1,178,912; 2,648,103)	1,844,134 (1,179,156 2,648,356)
Eritrea	659,033 (446,822; 920,763)	659,433 (447,369; 921,104)	659,831 (447,915; 921,444)	660,228 (448,458; 921,728)	660,623 (448,945; 921,946)	700,415 (476,071; 977,128)	700,830 (476,438; 977,358)	701,244 (476,804; 977,589)	701,656 (477,169; 977,819)	702,066 (477,642; 978,048)
Eswatini	474,747 (285,753; 724,272)	474,990 (286,062; 724,444)	475,232 (286,371; 724,616)	475,474 (286,678; 724,787)	475,714 (286,984; 724,957)	478,648 (288,916; 729,232)	478,887 (289,223; 729,402)	479,126 (289,531; 729,571)	479,364 (289,838; 729,738)	479,600 (290,144; 729,905)
Ethiopia	17,372,037 (11,356,787; 24,477,839)	17,558,477 (11,481,638; 24,724,802)	17,746,858 (11,614,722; 24,974,251)	17,937,200 (11,754,423; 25,226,210)	18,129,522 (11,895,666; 25,480,706)	18,991,298 (12,476,975; 26,675,272)	19,194,794 (12,626,613; 26,944,374)	19,400,407 (12,777,704; 27,216,185)	19,608,157 (12,916,512; 27,495,836)	19,818,067 (13,056,796 27,782,335
Fiji	463,328 (285,393; 696,488)	463,387 (285,432; 696,611)	463,445 (285,472; 696,732)	463,503 (285,512; 696,853)	463,561 (285,551; 696,973)	453,304 (279,237; 681,585)	453,360 (279,275; 681,701)	453,416 (279,314; 681,817)	453,472 (279,352; 681,932)	453,528 (279,390; 682,047)
Gabon	1,052,572 (486,129; 1,690,876)	1,064,159 (491,666; 1,708,236)	1,075,869 (498,296; 1,725,774)	1,087,702 (505,212; 1,743,490)	1,099,659 (511,681; 1,761,386)	1,117,063 (520,703; 1,787,979)	1,129,332 (527,348; 1,806,330)	1,141,731 (534,066; 1,824,867)	1,154,261 (540,857; 1,843,964)	1,166,922 (547,723; 1,863,317)
Gambia, The	510,208 (309,981; 735,828)	510,718 (310,617; 736,139)	511,227 (311,334; 736,738)	511,734 (312,051; 737,436)	512,239 (312,760; 738,128)	549,701 (336,067; 791,696)	550,238 (336,820; 792,056)	550,773 (337,572; 792,415)	551,305 (338,321; 792,774)	551,836 (339,068; 793,132)
Georgia	1,200,896 (760,094; 1,794,905)	1,201,041 (760,262; 1,795,023)	1,201,185 (760,429; 1,795,140)	1,201,329 (760,596; 1,795,256)	1,201,473 (760,762; 1,795,372)	1,098,757 (695,792; 1,641,794)	1,098,887 (695,943; 1,641,900)	1,099,017 (696,093; 1,642,005)	1,099,146 (696,243; 1,642,109)	1,099,274 (696,392; 1,642,213)

Ghana	6,868,714 (4,771,993; 9,504,206)	6,873,840 (4,775,931; 9,506,528)	6,878,945 (4,778,967; 9,508,846)	6,884,030 (4,781,998; 9,511,161)	6,889,095 (4,785,067; 9,513,472)	7,196,089 (4,997,886; 9,932,552)	7,201,334 (5,001,132; 9,934,958)	7,206,558 (5,004,424; 9,937,359)	7,211,761 (5,007,710; 9,939,757)	7,216,944 (5,010,991 9,942,152)
Guatemala	6,991,242 (4,192,026; 10,644,002)	6,994,313 (4,193,940; 10,648,354)	6,997,372 (4,195,849; 10,652,686)	7,000,418 (4,197,751; 10,656,997)	7,003,453 (4,199,871; 10,661,288)	7,031,240 (4,217,268; 10,703,258)	7,034,261 (4,219,815; 10,707,524)	7,037,270 (4,224,841; 10,711,770)	7,040,267 (4,232,136; 10,715,997)	7,043,252 (4,237,487 10,718,564
Guinea	1,554,851 (1,027,140; 2,182,560)	1,571,490 (1,039,407; 2,205,286)	1,588,303 (1,051,809; 2,228,245)	1,605,290 (1,064,361; 2,251,440)	1,622,454 (1,076,802; 2,274,874)	1,765,443 (1,171,884; 2,474,671)	1,784,308 (1,184,590; 2,500,422)	1,803,368 (1,197,432; 2,526,438)	1,822,627 (1,210,409; 2,552,722)	1,842,085 (1,223,525 2,579,275)
Guinea-Bissau	354,517 (227,938; 502,838)	358,337 (230,631; 508,102)	362,196 (233,503; 513,416)	366,096 (236,408; 518,784)	370,037 (239,346; 524,044)	392,347 (254,194; 555,273)	396,568 (257,348; 560,877)	400,832 (260,538; 566,538)	405,141 (263,764; 572,255)	406,982 (265,073; 574,484)
Haiti	928,138 (634,154; 1,296,708)	937,988 (640,859; 1,310,125)	947,941 (648,613; 1,323,678)	957,996 (656,451; 1,337,371)	968,155 (664,372; 1,351,203)	960,504 (660,068; 1,340,179)	970,685 (668,012; 1,354,037)	980,970 (676,044; 1,368,037)	991,363 (684,180; 1,382,179)	1,001,862 (692,404; 1,396,466
Honduras	3,490,567 (2,166,145; 5,166,256)	3,491,871 (2,167,045; 5,167,131)	3,493,170 (2,167,941; 5,167,993)	3,494,463 (2,168,834; 5,168,851)	3,495,752 (2,169,724; 5,169,705)	3,458,974 (2,146,987; 5,114,280)	3,460,238 (2,147,861; 5,115,117)	3,461,497 (2,148,733; 5,115,951)	3,462,752 (2,149,602; 5,116,782)	3,464,001 (2,150,467 5,117,609
India	189,504,458 (145,122,790; 243,473,223)	189,552,776 (145,193,823; 243,527,024)	189,600,902 (145,264,543; 243,580,663)	189,648,837 (145,334,952; 243,634,141)	189,696,582 (145,405,050; 243,687,458)	187,275,882 (143,582,760; 240,569,955)	187,322,633 (143,651,738; 240,622,263)	187,369,199 (143,720,410; 240,674,413)	187,415,580 (143,788,779; 240,726,407)	187,461,77[illegible] (143,856,84[illegible] 240,778,24[illegible]
Indonesia	111,657,379 (68,832,444; 167,353,734)	111,668,552 (68,846,122; 167,369,698)	111,679,681 (68,859,741; 167,385,605)	111,690,766 (68,873,301; 167,401,455)	111,701,807 (68,886,803; 167,417,249)	109,844,141 (67,747,724; 164,632,268)	109,854,911 (67,760,884; 164,647,686)	109,865,639 (67,773,987; 164,663,048)	109,876,324 (67,787,033; 164,678,355)	109,886,96[illegible] (67,800,02[illegible] 164,693,60[illegible]
Iraq	27,064,292 (16,500,631; 40,226,825)	27,321,194 (16,657,186; 40,604,197)	27,325,455 (16,659,714; 40,606,081)	27,329,700 (16,662,234; 40,607,959)	27,333,927 (16,664,747; 40,609,833)	28,873,178 (17,603,121; 42,892,056)	28,877,607 (17,605,760; 42,894,025)	28,882,019 (17,608,390; 42,895,990)	28,886,412 (17,611,012; 42,897,949)	28,890,789 (17,613,627 42,899,903
Jamaica	730,764 (409,133; 1,136,290)	731,275 (409,658; 1,136,618)	731,784 (410,182; 1,136,944)	732,291 (410,703; 1,137,269)	732,796 (411,226; 1,137,593)	682,843 (383,417; 1,059,619)	683,310 (383,905; 1,059,919)	683,774 (384,396; 1,060,218)	684,237 (384,886; 1,060,515)	684,698 (385,373; 1,060,812
Jordan	4,729,737 (2,827,209; 7,084,575)	4,730,736 (2,827,824; 7,085,016)	4,731,732 (2,828,437; 7,085,456)	4,732,723 (2,829,048; 7,085,895)	4,733,711 (2,829,658; 7,086,333)	4,604,676 (2,752,544; 6,892,161)	4,605,629 (2,753,134; 6,892,584)	4,606,578 (2,753,722; 6,893,007)	4,607,523 (2,754,309; 6,893,428)	4,608,465 (2,754,894 6,893,849
Kazakhstan	8,600,633 (5,281,154; 13,005,547)	8,602,776 (5,283,661; 13,007,036)	8,604,911 (5,286,159; 13,008,521)	8,607,037 (5,288,649; 13,010,002)	8,609,154 (5,291,129; 13,011,479)	8,136,025 (5,001,457; 12,294,791)	8,138,009 (5,003,784; 12,296,178)	8,139,986 (5,006,102; 12,297,561)	8,141,955 (5,008,412; 12,298,940)	8,143,916 (5,010,714 12,300,315

Kenya	11,106,787 (7,730,711; 15,365,893)	11,114,971 (7,735,802; 15,369,600)	11,123,122 (7,740,884; 15,373,301)	11,131,241 (7,745,957; 15,376,997)	11,139,327 (7,751,020; 15,380,687)	11,803,776 (8,212,777; 16,290,254)	11,812,271 (8,218,119; 16,294,149)	11,820,732 (8,223,452; 16,298,039)	11,829,159 (8,240,432; 16,301,924)	11,837,553 (8,257,563 16,305,802
Kiribati	62,161 (39,883; 89,114)	62,169 (39,890; 89,120)	62,176 (39,896; 89,125)	62,184 (39,902; 89,131)	62,191 (39,908; 89,136)	61,613 (39,538; 88,302)	61,620 (39,544; 88,307)	61,628 (39,549; 88,313)	61,635 (39,555; 88,318)	61,642 (39,561; 88,323)
Kyrgyz Republic	1,249,425 (937,791; 1,630,145)	1,249,697 (938,064; 1,630,345)	1,249,969 (938,338; 1,630,545)	1,250,239 (938,610; 1,630,744)	1,250,509 (938,882; 1,630,942)	1,205,896 (905,454; 1,572,611)	1,206,154 (905,714; 1,572,855)	1,206,411 (905,973; 1,573,140)	1,206,666 (906,236; 1,573,423)	1,206,921 (906,501; 1,573,705)
Lao PDR	1,213,224 (936,346; 1,562,968)	1,225,590 (945,959; 1,578,747)	1,238,082 (955,669; 1,594,684)	1,250,699 (965,479; 1,610,897)	1,263,445 (975,388; 1,627,305)	1,229,525 (949,270; 1,583,609)	1,240,041 (957,458; 1,597,145)	1,240,272 (957,705; 1,597,433)	1,240,502 (957,951; 1,597,721)	1,240,731 (958,197; 1,598,006)
Lesotho	358,976 (250,117; 496,585)	359,238 (250,280; 496,704)	359,500 (250,443; 496,823)	359,760 (250,606; 496,941)	360,020 (250,769; 497,060)	356,680 (248,425; 492,213)	356,935 (248,906; 492,330)	357,189 (249,422; 492,447)	357,442 (249,711; 492,563)	357,694 (249,958; 492,680)
Liberia	817,236 (547,766; 1,144,974)	825,945 (553,983; 1,156,867)	834,745 (560,266; 1,168,882)	843,637 (566,499; 1,181,020)	852,620 (572,609; 1,193,283)	925,541 (621,666; 1,295,003)	935,391 (628,368; 1,308,446)	945,343 (635,141; 1,322,028)	955,399 (641,985; 1,335,749)	965,558 (648,902; 1,349,610)
Madagascar	4,610,222 (3,092,629; 6,458,181)	4,659,341 (3,127,703; 6,525,250)	4,708,969 (3,163,154; 6,593,008)	4,759,111 (3,198,453; 6,661,461)	4,809,773 (3,232,934; 6,730,617)	5,248,010 (3,527,974; 7,341,967)	5,303,845 (3,565,992; 7,418,171)	5,360,259 (3,604,412; 7,495,157)	5,417,257 (3,643,237; 7,572,933)	5,474,845 (3,682,473 7,651,507)
Malawi	4,203,551 (2,799,847; 5,895,215)	4,206,384 (2,804,566; 5,897,551)	4,209,206 (2,808,448; 5,899,881)	4,212,016 (2,810,881; 5,902,204)	4,214,816 (2,813,157; 5,904,521)	4,603,358 (3,072,935; 6,447,087)	4,606,389 (3,075,409; 6,449,602)	4,609,408 (3,077,877; 6,452,111)	4,612,415 (3,080,341; 6,454,613)	4,615,411 (3,082,799 6,457,107)
Malaysia	13,632,814 (8,357,747; 20,627,827)	13,635,842 (8,360,673; 20,630,549)	13,638,858 (8,361,673; 20,633,262)	13,641,863 (8,362,668; 20,635,964)	13,644,855 (8,363,661; 20,638,657)	13,184,173 (8,080,478; 19,940,085)	13,187,041 (8,081,433; 19,942,668)	13,189,897 (8,082,387; 19,945,241)	13,192,742 (8,083,339; 19,947,804)	13,195,575 (8,084,289 19,950,358
Mali	4,344,343 (2,787,872; 6,170,301)	4,391,217 (2,819,659; 6,234,938)	4,438,580 (2,851,786; 6,300,239)	4,486,437 (2,884,257; 6,366,212)	4,534,793 (2,917,076; 6,432,862)	5,068,082 (3,262,782; 7,187,176)	5,122,670 (3,301,342; 7,262,364)	5,177,828 (3,340,358; 7,337,181)	5,233,561 (3,379,854; 7,411,239)	5,289,875 (3,421,539 7,486,042)
Mauritania	1,048,464 (733,504; 1,447,987)	1,059,703 (741,571; 1,463,280)	1,071,060 (749,724; 1,478,731)	1,082,535 (757,964; 1,494,340)	1,094,129 (766,292; 1,510,108)	1,184,903 (830,094; 1,634,728)	1,185,729 (830,899; 1,635,107)	1,186,551 (831,703; 1,635,485)	1,187,370 (832,504; 1,635,862)	1,188,186 (833,194; 1,636,239)
Mexico	27,606,991 (12,533,779; 45,854,960)	27,922,570 (12,684,690; 46,340,587)	28,241,541 (12,837,870; 46,831,248)	28,563,939 (12,992,779; 47,326,996)	28,705,125 (13,065,379; 47,522,148)	27,698,043 (12,632,659; 45,817,748)	27,736,245 (12,700,374; 45,844,009)	27,774,295 (12,767,740; 45,870,182)	27,812,194 (12,834,759; 45,896,266)	27,849,941 (12,901,431 45,946,754

Mongolia	1,415,273 (909,847; 2,024,572)	1,415,419 (909,978; 2,024,679)	1,415,565 (910,108; 2,024,785)	1,415,710 (910,237; 2,024,891)	1,415,855 (910,366; 2,024,997)	1,331,719 (856,302; 1,904,568)	1,331,854 (856,423; 1,904,667)	1,331,988 (856,544; 1,904,766)	1,332,122 (856,664; 1,904,864)	1,332,256 (856,784; 1,904,962)
Morocco	12,753,923 (7,968,323; 18,629,516)	12,757,879 (7,981,433; 18,631,759)	12,761,820 (7,992,605; 18,633,994)	12,765,745 (7,996,339; 18,636,221)	12,769,653 (8,000,060; 18,638,440)	12,310,749 (7,713,784; 17,967,023)	12,314,486 (7,717,344; 17,973,196)	12,318,209 (7,720,893; 17,979,341)	12,321,916 (7,724,428; 17,985,457)	12,325,609 (7,727,951 17,991,545
Mozambique	6,945,340 (4,540,308; 9,787,228)	6,950,379 (4,544,778; 9,788,093)	6,955,398 (4,551,093; 9,788,956)	6,960,397 (4,560,240; 9,789,817)	6,965,376 (4,569,352; 9,790,676)	7,616,158 (5,002,635; 10,698,747)	7,621,555 (5,012,517; 10,699,681)	7,626,931 (5,022,361; 10,700,612)	7,632,286 (5,026,891; 10,702,721)	7,637,619 (5,031,179 10,707,174
Myanmar	7,461,602 (5,819,758; 9,555,246)	7,462,587 (5,820,366; 9,557,259)	7,463,567 (5,820,973; 9,559,262)	7,464,543 (5,821,579; 9,561,254)	7,465,516 (5,822,183; 9,563,194)	7,408,301 (5,777,411; 9,489,807)	7,409,259 (5,778,008; 9,490,798)	7,410,212 (5,778,604; 9,491,784)	7,411,162 (5,779,198; 9,492,756)	7,412,108 (5,779,792 9,493,717)
Namibia	1,490,115 (814,475; 2,286,691)	1,490,929 (815,807; 2,287,288)	1,491,739 (817,132; 2,287,882)	1,492,547 (818,450; 2,288,473)	1,493,351 (819,761; 2,289,062)	1,508,720 (829,071; 2,311,972)	1,509,526 (830,381; 2,312,560)	1,510,328 (831,659; 2,313,146)	1,511,127 (832,747; 2,313,728)	1,511,923 (833,831; 2,314,308)
Nepal	4,606,505 (3,566,203; 5,891,222)	4,607,358 (3,567,468; 5,892,676)	4,608,208 (3,568,728; 5,894,123)	4,609,055 (3,569,983; 5,895,563)	4,609,898 (3,571,232; 5,896,997)	4,266,772 (3,305,964; 5,458,395)	4,267,546 (3,307,110; 5,459,710)	4,268,317 (3,307,754; 5,461,019)	4,269,085 (3,308,243; 5,462,322)	4,269,850 (3,308,730 5,463,620)
Nicaragua	998,771 (710,011; 1,321,117)	999,496 (710,951; 1,321,635)	1,000,218 (711,890; 1,322,151)	1,000,937 (712,826; 1,322,665)	1,001,654 (713,759; 1,323,177)	967,596 (689,963; 1,277,771)	968,282 (690,915; 1,278,262)	968,965 (691,661; 1,278,752)	969,646 (692,495; 1,279,239)	970,324 (693,430; 1,279,726)
Niger	6,594,239 (4,434,611; 9,233,129)	6,664,432 (4,484,802; 9,328,771)	6,735,352 (4,535,532; 9,425,389)	6,807,006 (4,586,616; 9,522,994)	6,879,403 (4,636,000; 9,621,596)	8,000,490 (5,392,200; 11,186,457)	8,005,479 (5,396,271; 11,190,346)	8,010,448 (5,400,334; 11,194,220)	8,015,398 (5,404,388; 11,198,079)	8,020,328 (5,408,509 11,201,923
Nigeria	34,803,931 (23,367,899; 48,600,374)	35,183,992 (23,633,435; 49,104,199)	35,568,044 (23,901,854; 49,613,209)	35,956,130 (24,173,187; 50,129,749)	36,348,291 (24,447,464; 50,666,284)	39,808,972 (26,786,695; 55,478,994)	40,242,804 (27,090,327; 56,072,316)	40,681,189 (27,397,252; 56,671,750)	41,124,173 (27,707,503; 57,277,359)	41,571,804 (28,018,714 57,889,204
Pakistan	44,693,843 (32,097,721; 59,156,554)	45,128,061 (32,432,023; 59,721,241)	45,145,029 (32,466,588; 59,733,704)	45,161,930 (32,507,193; 59,746,122)	45,178,763 (32,534,909; 59,758,495)	45,193,459 (32,566,908; 59,768,087)	45,210,157 (32,600,273; 59,780,369)	45,226,787 (32,633,515; 59,792,607)	45,243,352 (32,666,634; 59,804,801)	45,259,850 (32,699,630 59,816,950
Papua New Guinea	780,221 (593,409; 1,013,481)	788,242 (599,617; 1,023,753)	796,345 (605,888; 1,034,129)	804,530 (612,224; 1,044,609)	812,798 (618,624; 1,055,196)	859,569 (654,338; 1,115,757)	868,401 (661,185; 1,127,064)	877,323 (668,102; 1,138,484)	886,335 (675,091; 1,150,020)	895,439 (682,151; 1,161,672)
Paraguay	2,299,883 (1,366,659; 3,508,979)	2,300,971 (1,367,785; 3,510,521)	2,302,055 (1,368,908; 3,512,056)	2,303,134 (1,370,028; 3,513,584)	2,304,210 (1,371,144; 3,515,104)	2,254,787 (1,342,199; 3,439,591)	2,255,830 (1,343,284; 3,441,064)	2,256,869 (1,344,366; 3,442,530)	2,257,904 (1,345,444; 3,443,990)	2,258,935 (1,346,520 3,445,442)

Peru	8,670,669 (4,794,016; 13,573,733)	8,677,197 (4,797,938; 13,577,916)	8,683,699 (4,801,851; 13,582,085)	8,690,174 (4,805,755; 13,586,239)	8,696,624 (4,809,649; 13,590,378)	8,322,688 (4,605,531; 13,000,365)	8,328,807 (4,611,603; 13,004,296)	8,334,902 (4,617,659; 13,008,213)	8,340,972 (4,623,700; 13,012,116)	8,347,018 (4,629,725 13,016,006
Philippines	37,226,676 (23,893,876; 53,304,053)	37,603,151 (24,136,190; 53,840,167)	37,983,416 (24,380,948; 54,381,661)	38,367,509 (24,628,172; 54,928,591)	38,755,469 (24,877,892; 55,481,011)	39,524,937 (25,372,562; 56,579,511)	39,924,564 (25,629,856; 57,148,512)	40,328,213 (25,889,743; 57,723,224)	40,735,926 (26,152,251; 58,303,705)	41,147,742 (26,417,405 58,890,012
Rwanda	2,559,482 (1,646,616; 3,629,589)	2,561,440 (1,650,610; 3,631,249)	2,563,390 (1,654,606; 3,632,893)	2,565,333 (1,658,589; 3,634,036)	2,567,268 (1,662,569; 3,634,370)	2,658,173 (1,724,259; 3,760,582)	2,660,159 (1,728,356; 3,760,926)	2,662,138 (1,732,439; 3,761,268)	2,664,108 (1,735,845; 3,761,610)	2,666,070 (1,737,472 3,761,951)
Senegal	4,324,534 (3,014,684; 5,981,778)	4,327,685 (3,016,644; 5,983,445)	4,330,824 (3,018,601; 5,984,870)	4,333,951 (3,020,555; 5,986,293)	4,337,065 (3,022,505; 5,987,714)	4,660,904 (3,251,990; 6,431,729)	4,664,222 (3,258,360; 6,433,251)	4,667,526 (3,261,588; 6,434,770)	4,670,818 (3,264,807; 6,436,287)	4,674,096 (3,268,017 6,437,802)
Sierra Leone	1,517,996 (975,246; 2,154,275)	1,519,169 (976,583; 2,155,283)	1,520,338 (977,914; 2,156,287)	1,521,502 (979,431; 2,157,271)	1,522,662 (981,577; 2,158,249)	1,563,146 (1,009,344; 2,214,580)	1,564,326 (1,011,768; 2,214,784)	1,565,501 (1,014,186; 2,214,987)	1,566,672 (1,016,604; 2,215,191)	1,567,838 (1,019,015 2,215,393)
Solomon Islands	178,652 (134,277; 233,902)	178,717 (134,338; 233,950)	178,783 (134,400; 233,998)	178,848 (134,461; 234,046)	178,912 (134,522; 234,093)	190,157 (142,990; 248,767)	190,225 (143,055; 248,817)	190,293 (143,119; 248,866)	190,361 (143,183; 248,916)	190,428 (143,247; 248,965)
Somalia	2,067,829 (1,458,510; 2,873,819)	2,089,335 (1,473,926; 2,903,710)	2,111,062 (1,489,053; 2,933,906)	2,133,011 (1,505,270; 2,964,412)	2,155,184 (1,521,637; 2,995,228)	2,440,166 (1,724,284; 3,391,290)	2,465,523 (1,743,649; 3,426,532)	2,491,141 (1,763,218; 3,462,133)	2,517,020 (1,782,995; 3,498,035)	2,543,163 (1,802,733 3,534,116)
South Africa	22,300,493 (10,198,976; 35,976,278)	22,323,481 (10,213,860; 35,986,057)	22,346,378 (10,237,721; 35,995,810)	22,369,183 (10,251,101; 36,005,538)	22,391,898 (10,261,095; 36,015,240)	22,143,587 (10,171,860; 35,589,464)	22,165,850 (10,207,010; 35,598,998)	22,188,024 (10,232,289; 35,608,508)	22,210,110 (10,268,081; 35,617,992)	22,232,109 (10,310,937 35,627,452
Sri Lanka	6,376,509 (4,111,291; 9,196,241)	6,377,431 (4,113,131; 9,197,302)	6,378,349 (4,114,963; 9,198,359)	6,379,264 (4,116,784; 9,199,411)	6,380,175 (4,118,597; 9,200,459)	6,136,580 (3,962,520; 8,848,932)	6,137,449 (3,964,245; 8,849,931)	6,138,315 (3,965,961; 8,850,926)	6,139,177 (3,967,668; 8,851,916)	6,140,036 (3,969,366 8,852,903)
Sudan	8,603,477 (5,649,506; 12,152,515)	8,609,176 (5,652,707; 12,156,498)	8,614,852 (5,655,900; 12,160,463)	8,620,506 (5,659,167; 12,164,410)	8,626,136 (5,662,570; 12,168,339)	9,311,068 (6,123,184; 13,130,213)	9,317,093 (6,139,504; 13,134,414)	9,323,094 (6,154,927; 13,138,595)	9,329,072 (6,162,559; 13,142,758)	9,335,025 (6,170,023 13,146,902
Suriname	144,213 (80,385; 224,940)	145,760 (81,292; 227,257)	147,324 (82,210; 229,598)	148,904 (83,137; 231,962)	150,500 (84,075; 234,351)	148,477 (82,990; 231,104)	150,068 (83,926; 233,483)	151,675 (84,871; 235,887)	153,300 (85,826; 238,314)	154,940 (86,794; 240,767)
Tajikistan	1,915,333 (1,391,705; 2,598,478)	1,915,815 (1,392,181; 2,599,172)	1,916,295 (1,392,655; 2,599,862)	1,916,773 (1,393,128; 2,600,548)	1,917,250 (1,393,598; 2,601,230)	1,915,253 (1,392,271; 2,598,555)	1,915,725 (1,392,737; 2,599,229)	1,916,195 (1,393,202; 2,599,899)	1,916,663 (1,393,671; 2,600,565)	1,917,129 (1,394,137 2,601,228)

Tanzania	16,243,963<br>(11,567,705;<br>22,229,401)	16,254,111<br>(11,574,250;<br>22,240,345)	16,264,218<br>(11,587,994;<br>22,251,231)	16,274,286<br>(11,597,713;<br>22,262,059)	16,284,313<br>(11,607,406;<br>22,272,828)	18,089,007<br>(12,896,614;<br>24,737,920)	18,100,051<br>(12,907,315;<br>24,744,624)	18,111,051<br>(12,917,988;<br>24,749,974)	18,122,007<br>(12,928,631;<br>24,755,299)	18,132,919<br>(12,939,246<br>24,760,599
Thailand	16,921,382<br>(10,243,881;<br>25,423,378)	16,924,448<br>(10,249,584;<br>25,430,118)	16,927,503<br>(10,255,271;<br>25,436,817)	16,930,545<br>(10,260,942;<br>25,443,476)	16,933,574<br>(10,262,338;<br>25,450,094)	15,827,962<br>(9,591,397;<br>23,790,337)	15,830,771<br>(9,592,204;<br>23,796,448)	15,833,569<br>(9,593,011;<br>23,802,521)	15,836,356<br>(9,593,817;<br>23,808,557)	15,839,132<br>(9,594,622<br>23,814,556
Timor-Leste	709,231<br>(465,032;<br>1,018,133)	709,291<br>(465,189;<br>1,018,203)	709,351<br>(465,344;<br>1,018,273)	709,410<br>(465,499;<br>1,018,343)	709,469<br>(465,653;<br>1,018,412)	718,849<br>(471,925;<br>1,031,859)	718,908<br>(472,079;<br>1,031,929)	718,967<br>(472,233;<br>1,031,998)	719,027<br>(472,385;<br>1,032,067)	719,085<br>(472,537;<br>1,032,136)
Togo	1,590,476<br>(1,051,746;<br>2,232,316)	1,606,433<br>(1,063,596;<br>2,254,069)	1,607,540<br>(1,065,628;<br>2,254,982)	1,608,642<br>(1,067,664;<br>2,255,894)	1,609,740<br>(1,069,116;<br>2,256,803)	1,742,656<br>(1,157,570;<br>2,442,468)	1,743,834<br>(1,158,532;<br>2,443,446)	1,745,008<br>(1,159,492;<br>2,444,421)	1,746,177<br>(1,160,449;<br>2,445,394)	1,747,342<br>(1,161,405<br>2,446,364)
Tunisia	3,324,736<br>(2,022,133;<br>4,899,278)	3,326,030<br>(2,024,269;<br>4,900,228)	3,327,318<br>(2,026,400;<br>4,901,172)	3,328,602<br>(2,028,260;<br>4,902,113)	3,329,881<br>(2,030,165;<br>4,903,049)	3,018,306<br>(1,841,801;<br>4,443,400)	3,019,456<br>(1,843,674;<br>4,444,215)	3,020,600<br>(1,845,542;<br>4,445,027)	3,021,740<br>(1,847,407;<br>4,445,834)	3,022,876<br>(1,849,267<br>4,446,638)
Turkey	31,793,626<br>(19,107,831;<br>48,254,284)	31,803,954<br>(19,120,414;<br>48,259,094)	31,814,240<br>(19,132,939;<br>48,263,887)	31,824,485<br>(19,145,407;<br>48,277,801)	31,834,689<br>(19,157,818;<br>48,293,337)	30,457,092<br>(18,334,760;<br>46,203,553)	30,466,774<br>(18,346,522;<br>46,218,258)	30,476,417<br>(18,358,231;<br>46,232,886)	30,486,021<br>(18,369,892;<br>46,247,438)	30,495,587<br>(18,381,702<br>46,261,915
Uganda	10,147,675<br>(6,616,454;<br>14,343,427)	10,155,205<br>(6,631,932;<br>14,344,721)	10,162,706<br>(6,641,101;<br>14,346,010)	10,170,176<br>(6,647,726;<br>14,347,297)	10,177,617<br>(6,654,324;<br>14,348,580)	10,775,757<br>(7,047,226;<br>15,182,149)	10,783,567<br>(7,054,151;<br>15,183,500)	10,791,346<br>(7,061,421;<br>15,184,848)	10,799,094<br>(7,075,613;<br>15,186,192)	10,806,812<br>(7,087,452<br>15,187,533
Ukraine	6,546,351<br>(4,266,666;<br>9,632,058)	6,612,763<br>(4,310,071;<br>9,728,737)	6,679,844<br>(4,353,915;<br>9,826,386)	6,747,602<br>(4,398,202;<br>9,925,014)	6,816,043<br>(4,442,937;<br>10,024,630)	6,362,660<br>(4,147,522;<br>9,356,844)	6,416,780<br>(4,182,917;<br>9,435,449)	6,417,677<br>(4,183,618;<br>9,435,791)	6,418,571<br>(4,184,318;<br>9,436,549)	6,419,461<br>(4,185,015<br>9,437,416)
Uzbekistan	5,337,382<br>(3,935,197;<br>7,066,046)	5,339,270<br>(3,937,411;<br>7,067,101)	5,341,150<br>(3,939,617;<br>7,068,153)	5,343,022<br>(3,941,813;<br>7,069,197)	5,344,887<br>(3,944,000;<br>7,070,221)	4,994,571<br>(3,686,292;<br>6,605,481)	4,996,299<br>(3,688,385;<br>6,606,320)	4,998,020<br>(3,690,289;<br>6,606,852)	4,999,734<br>(3,692,094;<br>6,607,383)	5,001,442<br>(3,693,892<br>6,607,910)
Vanuatu	162,685<br>(102,912;<br>236,071)	162,722<br>(102,944;<br>236,099)	162,760<br>(102,974;<br>236,126)	162,797<br>(103,005;<br>236,154)	162,834<br>(103,035;<br>236,181)	172,767<br>(109,326;<br>250,558)	172,806<br>(109,358;<br>250,587)	172,845<br>(109,389;<br>250,616)	172,884<br>(109,421;<br>250,644)	172,922<br>(109,453;<br>250,672)
Vietnam	28,053,308<br>(18,092,436;<br>40,077,818)	28,055,885<br>(18,094,758;<br>40,079,700)	28,058,452<br>(18,097,075;<br>40,081,575)	28,061,009<br>(18,099,385;<br>40,083,444)	28,063,555<br>(18,101,690;<br>40,085,307)	26,543,879<br>(17,122,088;<br>37,912,967)	26,546,269<br>(17,124,256;<br>37,914,717)	26,548,649<br>(17,126,419;<br>37,916,461)	26,551,019<br>(17,128,577;<br>37,918,200)	26,553,380<br>(17,130,728<br>37,919,932
Yemen, Rep.	4,110,668<br>(2,395,732;<br>6,052,666)	4,155,829<br>(2,422,622;<br>6,115,940)	4,201,466<br>(2,449,804;<br>6,179,862)	4,247,584<br>(2,477,281;<br>6,244,438)	4,294,187<br>(2,505,056;<br>6,309,674)	4,373,117<br>(2,551,709;<br>6,422,334)	4,421,055<br>(2,585,038;<br>6,489,401)	4,469,498<br>(2,618,930;<br>6,557,154)	4,518,451<br>(2,653,267;<br>6,625,600)	4,567,918<br>(2,689,049<br>6,694,489)

Zambia	4,934,117<br>(3,503,874;<br>6,762,187)	4,937,268<br>(3,506,929;<br>6,765,586)	4,940,407<br>(3,509,687;<br>6,768,967)	4,943,534<br>(3,510,899;<br>6,772,330)	4,946,648<br>(3,516,283;<br>6,775,674)	5,483,489<br>(3,898,774;<br>7,509,990)	5,486,911<br>(3,902,091;<br>7,513,655)	5,490,320<br>(3,905,398;<br>7,517,301)	5,493,716<br>(3,908,697;<br>7,520,927)	5,497,098<br>(3,911,987<br>7,523,046)
Zimbabwe	3,084,389<br>(2,198,343;<br>4,218,985)	3,086,303<br>(2,200,322;<br>4,221,049)	3,088,209<br>(2,202,159;<br>4,223,101)	3,090,107<br>(2,203,992;<br>4,225,143)	3,091,998<br>(2,205,820;<br>4,227,174)	3,197,881<br>(2,281,851;<br>4,370,512)	3,199,820<br>(2,283,730;<br>4,371,456)	3,201,751<br>(2,285,604;<br>4,372,395)	3,203,675<br>(2,287,473;<br>4,373,330)	3,205,591<br>(2,289,337<br>4,374,261)

References

1 McGovern ME, Krishna A, Aguayo VM, Subramanian SV. A review of the evidence linking child stunting to economic outcomes. *Int J Epidemiol* 2017; **46**: 1171–91.

2 Victora CG, Adair L, Fall C, *et al.* Maternal and child undernutrition: consequences for adult health and human capital. *The Lancet* 2008; **371**: 340–57.

3 Phurichai Rungcharoenkitkul. Wealth effects and consumption in Thailand. Bangkok: Monetary Policy Group, Bank of Thailand, 2011.

4 International Monetary Fund. World Economic Outlook Databases (online). https://www.imf.org/en/Publications/SPROLLs/world-economic-outlook-databases (accessed Jan 13, 2021).

5 Jappelli T, Pistaferri L. Fiscal policy and MPC heterogeneity. *Am Econ J Macroecon* 2014; **6**: 107–36.

6 Glytsos NP. Dynamic effects of migrant remittances on growth: an econometric model with an application to Mediterranean countries. Centre of Planning and Economic Research Athens, Greece, 2002.

7 Reserve Bank of India. Exploring the slowdown. Macroeconomics of Growth: Structural Constraints in Indian Agriculture. Mumbai: Reserve Bank of India, 2002.

8 Khan K, Anwar S, Ahmed M, Kamal MA. Estimation of Consumption Functions: The Case of Bangladesh, India, Nepal, Pakistan and Sri Lanka. *Pak Bus Rev* 2015; **17**: 113–24.

9 Aitymbetov S. Emigrant remittances: Impact on economic development of Kyrgyzstan. *Kyrg Econ Policy Inst* 2006.

10 Bengtsson N. The marginal propensity to earn and consume out of unearned income: Evidence using an unusually large cash grant reform. *Scand J Econ* 2012; **114**: 1393–413.

11 Achoja FO. Multiplier effect of micro credit investment among small scale poultry agribusiness entrepreneurs in Delta state, Nigeria. *Trop Agric Res Ext* 2013; **15**.

12 Murugasu D, Wei AJ, Hwa TB. The Marginal Propensity to Consume across Household Income Groups. Kuala Lumpur: Bank Negara Malaysia, 2013.

13 Javed MS, Hussan S, Salik KM. Marginal propensity to consume: An application to small farmers of Punjab [Pakistan]. *J Agric Soc Sci Pak* 2005.

14 Graham JD. Valuing the future: OMB's refined position. *Univ Chic Law Rev* 2007; **74**: 51–7.

15 Hoddinott J, Alderman H, Behrman JR, Haddad L, Horton S. The economic rationale for investing in stunting reduction. *Matern Child Nutr* 2013; **9**: 69–82.

16 Alderman H, Behrman JR, Puett C. Big numbers about small children: Estimating the economic benefits of addressing undernutrition. *World Bank Res Obs* 2017; **32**: 107–25.

17 Galasso E, Wagstaff A. The aggregate income losses from childhood stunting and the returns to a nutrition intervention aimed at reducing stunting. *Econ Hum Biol* 2019; **34**: 225–38.

18 Fink G, Peet E, Danaei G, *et al.* Schooling and wage income losses due to early-childhood growth faltering in developing countries: national, regional, and global estimates. *Am J Clin Nutr* 2016; **104**: 104–12.

19 Bhutta ZA, Das JK, Rizvi A, *et al.* Evidence-based interventions for improvement of maternal and child nutrition: what can be done and at what cost? *The lancet* 2013; **382**: 452–77.

20 Anderson JD, Bagamian KH, Puett C, *et al.* Potential impact and cost-effectiveness of prospective Shigella vaccination in 102 low- and middle- income countries: a modelling study. *Forthcoming* 2022.

21 WHO. Market Information for Access to Vaccines (MI4A). Vaccine purchase database. 2020. https://www.who.int/teams/immunization-vaccines-and-biologicals/vaccine-access/mi4a/mi4a-vaccine-purchase-data (accessed Jan 20, 2022).

22 Prüss-Üstün A, Corvalán C. Preventing disease through healthy environments: towards an estimate of the environmental burden of disease. Geneva: World Health Organization, 2006.

23 Murray CJ, Lopez AD, Organization WH. The global burden of disease: a comprehensive assessment of mortality and disability from diseases, injuries, and risk factors in 1990 and projected to 2020: summary. Geneva: World Health Organization, 1996.
